# Supplementary material for: Nuclear Magnetic Resonance Fingerprints and Mini DNA Markers for the Authentication of Cinnamon Species Ingredients Used in Food and Natural Health Products
Source: Plants (Basel). 2024 Mar 14;13(6):841. doi: 10.3390/plants13060841 (PMC10975438; doi:10.3390/plants13060841)
Supplement: Supplementary file 1 [file plants-13-00841-s001.zip › plants-2821173-supplementary.pdf]

Supplementary Figure S1. Phylogenetic classification using complete chloroplast genomes for samples in this study and those of related taxa in Genbank

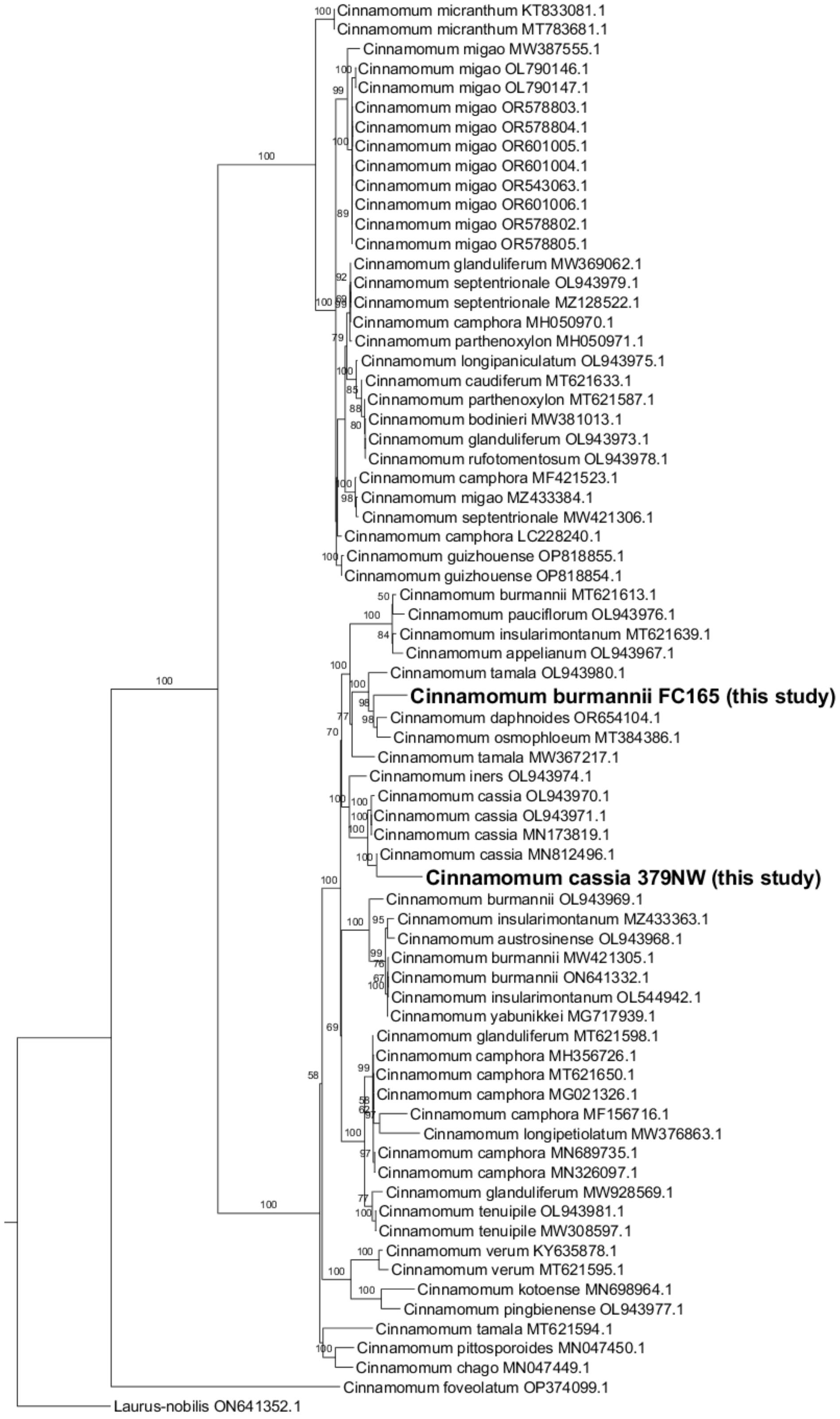

Supplementary Figure S2. Phylogenetic classification of samples using the Cincas_216 mini-DNA marker for samples in this study

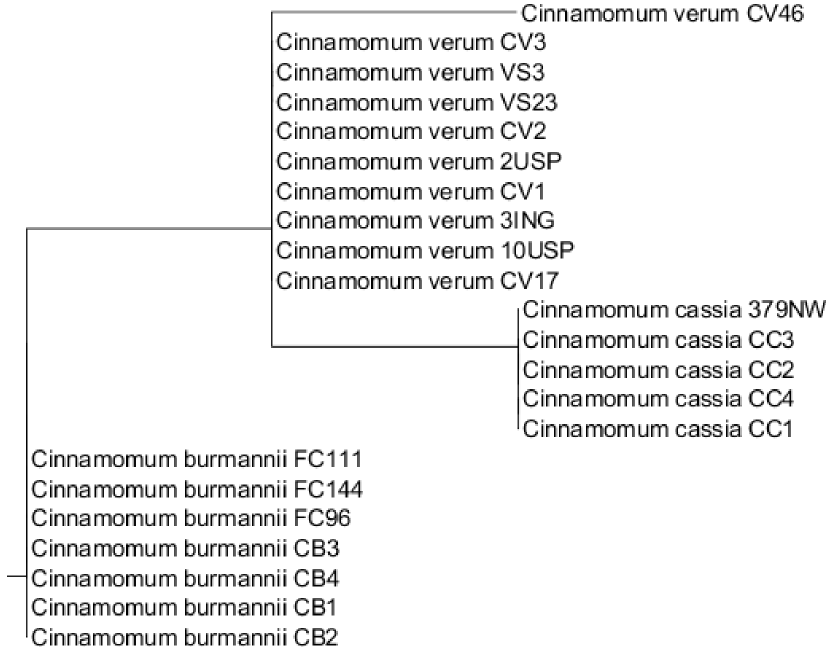

Supplementary Table S1. Specimen list and accession numbers

	**Species**	**Material**	**DNA Accession ID (NMR ID)**
1	*Cinnamomum burmannii* (Nees & T.Nees) Blume	leaf	BRM1531
2	*Cinnamomum burmannii* (Nees & T.Nees) Blume	leaf	BRM1532
3	*Cinnamomum burmannii* (Nees & T.Nees) Blume	leaf	BRM1533
4	*Cinnamomum burmannii* (Nees & T.Nees) Blume	leaf	BRM1534
5	*Cinnamomum burmannii* (Nees & T.Nees) Blume	bark powder	FC96 (*Cin_burm*_30)
6	*Cinnamomum burmannii* (Nees & T.Nees) Blume	bark powder	FC111 (*Cin_burm*_33)
7	*Cinnamomum burmannii* (Nees & T.Nees) Blume	bark powder	FC144 (*Cin_burm*_32)
8	*Cinnamomum cassia* (L.) J. Presl	leaf	BRM1535
9	*Cinnamomum cassia* (L.) J. Presl	leaf	BRM1536
10	*Cinnamomum cassia* (L.) J. Presl	leaf	BRM1537
11	*Cinnamomum cassia* (L.) J. Presl	leaf	BRM1538
12	*Cinnamomum cassia* (L.) J. Presl	bark powder	379NW (*Cin_cass*_17)
13	*Cinnamomum verum* J. Presl	leaf	BRM1547
14	*Cinnamomum verum* J. Presl	leaf	BRM1548
15	*Cinnamomum verum* J. Presl	leaf	BRM1549
16	*Cinnamomum verum* J. Presl	leaf	BRM1550
17	*Cinnamomum verum* J. Presl	leaf	BRM1551
18	*Cinnamomum verum* J. Presl	bark powder	VS3 (*Cin_veru*_36)
19	*Cinnamomum verum* J. Presl	bark	VS23
20	*Cinnamomum verum* J. Presl	bark powder	2USP (*Cin_veru*_29)
21	*Cinnamomum verum* J. Presl	bark	10USP
22	*Cinnamomum verum* J. Presl	bark powder	3ING (*Cin_veru*_47)

Supplementary Table S2. Specimen list and NMR ID

	**Species**	**Material**	**NMR ID**
1	*Cinnamomum verum* J. Presl	bark powder	*Cin_veru*_01
2	*Cinnamomum verum* J. Presl	extract	*Cin_veru*_02
3	*Cinnamomum verum* J. Presl	powder	*Cin_veru*_03
4	*Cinnamomum cassia* (L.) J. Presl	powder	*Cin_cass*_04
5	*Cinnamomum burmannii* (Nees & T.Nees) Blume	bark powder	*Cin_burm*_05
6	*Cinnamomum cassia* (L.) J. Presl	bark powder	*Cin_cass*_06
7	*Cinnamomum cassia* (L.) J. Presl	bark powder	*Cin_cass*_07
8	*Cinnamomum verum* J. Presl	powder	*Cin_veru*_08
9	*Cinnamomum burmannii* (Nees & T.Nees) Blume	powder	*Cin_burm*_09
10	*Cinnamomum verum* J. Presl	powder	*Cin_veru*_10
11	*Cinnamomum cassia* (L.) J. Presl	bark powder	*Cin_cass*_11
12	*Cinnamomum cassia* (L.) J. Presl	bark	*Cin_cass*_12
13	*Cinnamomum verum* J. Presl	powder	*Cin_veru*_13
14	*Cinnamomum verum* J. Presl	powder	*Cin_veru*_14
15	*Cinnamomum cassia* (L.) J. Presl	bark powder	*Cin_cass*_15
16	*Cinnamomum verum* J. Presl	extract	*Cin_veru*_16
17	*Cinnamomum cassia* (L.) J. Presl	bark powder	*Cin_cass*_17
18	*Cinnamomum cassia* (L.) J. Presl	powder	*Cin_cass*_18
19	*Cinnamomum cassia* (L.) J. Presl	powder	*Cin_cass*_19
20	*Cinnamomum cassia* (L.) J. Presl	bark	*Cin_cass*_20
21	*Cinnamomum cassia* (L.) J. Presl	bark	*Cin_cass*_21
22	*Cinnamomum verum* J. Presl	extract	*Cin_veru*_22
23	*Cinnamomum verum* J. Presl	extract	*Cin_veru*_23
24	*Cinnamomum verum* J. Presl	powder	*Cin_veru*_24
25	*Cinnamomum verum* J. Presl	powder	*Cin_veru*_25
26	*Cinnamomum verum* J. Presl	powder	*Cin_veru*_26
27	*Cinnamomum verum* J. Presl	powder	*Cin_veru*_27
28	*Cinnamomum verum* J. Presl	powder	*Cin_veru*_28
29	*Cinnamomum verum* J. Presl	bark powder	*Cin_veru*_29
30	*Cinnamomum burmannii* (Nees & T.Nees) Blume	bark powder	*Cin_burm*_30
31	*Cinnamomum burmannii* (Nees & T.Nees) Blume	bark powder	*Cin_burm*_31
32	*Cinnamomum burmannii* (Nees & T.Nees) Blume	bark powder	*Cin_burm*_32
33	*Cinnamomum burmannii* (Nees & T.Nees) Blume	bark powder	*Cin_burm*_33
34	*Cinnamomum burmannii* (Nees & T.Nees) Blume	bark powder	*Cin_burm*_34
35	*Cinnamomum verum* J. Presl	powder	*Cin_veru*_35
36	*Cinnamomum verum* J. Presl	bark powder	*Cin_veru*_36
37	*Cinnamomum verum* J. Presl	powder	*Cin_veru*_37
38	*Cinnamomum verum* J. Presl	extract	*Cin_veru*_38
39	*Cinnamomum verum* J. Presl	bark powder	*Cin_veru*_39
40	*Cinnamomum cassia* (L.) J. Presl	bark powder	*Cin_cass*_40
41	*Cinnamomum verum* J. Presl	powder	*Cin_veru*_41
42	*Cinnamomum verum* J. Presl	extract	*Cin_veru*_42
43	*Cinnamomum verum* J. Presl	extract	*Cin_veru*_43
44	*Cinnamomum verum* J. Presl	extract	*Cin_veru*_44
45	*Cinnamomum verum* J. Presl	powder	*Cin_veru*_45

46	*Cinnamomum verum* J. Presl	powder	*Cin_veru*_46
47	*Cinnamomum verum* J. Presl	bark powder	*Cin_veru*_47
48	*Cinnamomum verum* J. Presl	extract	*Cin_veru*_48

Supplementary Table S3. Genomic region locations for *C. burmannii* and *C. cassia*

	LSC (bp)	IRb (bp)	SSC (bp)	IRa (bp)
C. burmannii	1–93704	93705–113778	113779–132690	132691–152764
C. cassia	1–93684	93685–113750	113751–132658	132659–152724

Supplementary Table S4. FC165_C_burmannii_full length chloroplast genome

```
TTATAGGGATAGCGAATGCTGGGCGAACGACGGGAATTGAACCCGCGCATGGTGGATTCA
CAATCCACTGCCTTGATCCACTTGGCTACATCCGCCCCTCCTCTCTCAAAAGGATTCCAT
TTTCACCATTCATTATTTTTTGATTTAGTCTTTATTACTTCACTCTCCTTCCTGCTGAAA
TACAGATATTGTACATAAAACAAAATGTTGTACGTAAAAAAAAAAAAAAAAAATGCT
TTGATTTTTTCAAAAAATAAAATTATTTTGAAGAATAAGAGGATATAAAATGCAGGTTGG
TACAGAAGAAACTACGATATTCGATCATGAAATAACCAGCGGTTTTCATAAGTTGAATAA
AAGAAATGAAAATGAAAAACGATTATGTGAATAAAACACTACTGAACCAAATGGATCAAT
ACCAAACTTCTTGTTCTATTAAGAAGTTTGGTATTGATCCTTCAACGACTCGTATACACT
AATACCAAAGTATTATCCGTTTGTAGATGGAACTTCGACAGCAGCTAGGTCTAGAGGGAA
GTTGTGAGCATTACGTTCATGCATAACTTCCATACCAAGGTTAGCACGATTGATGATATC
AGCCCAAGTGTTAATAACACGACCTTGACTGTCAACTACAGATTGGTTGAAATTGAAACC
ATTTAGGTTGAAAGCCATGGTGCTGATACCTAAAGCAGTAAACCAGATACCTACTACAGG
CCAAGCTGCTAGGAAGAAATGTAAGGAACGGGAGTTGTTAAAACTAGCATATTGGAAGAT
CAATCGGCCAAAATAACCATGAGCAGCTACGATATTGTAAGTTTCTTCCTCTTGACCGAA
TCTGTAACCTGCATTAGCAGATTCATTTTCAGTGGTTTCCCTGATCAAACTAGAGGTTAC
CAAGGAACCATGCATAGCACTGAATAGGGAGCCGCCGAATACACCAGCTACGCCTAACAT
GTGAAATGGATGCATAAGGATATTGTGCTCTGCCTGGAATACAATCATGAAGTTGAAAGT
ACCAGATATTCCTAAAGGCATACCATCAGAAAAACTTCCTTGACCAATAGGGTAGATCAA
GAAAACAGCAGTAGCTGCTGCAACAGGAGCTGAATATGCAACAGCAATCCAAGGGCGCAT
ACCCAGACGGAAACTAAGTTCCCATTCACGACCCATGTAACAAGCTACACCAAGTAAGAA
GTGTAGAACAATTAGCTCATAAGGACCACCATTGTATAACCATTCATCAACGGATGCTGC
TTCCCATATTGGGTAAAAATGCAAACCTATAGCTGCAGAAGTGGGAATAATGGCACCGGA
AATAATATTGTTTCCATAAAGTAGAGACCCAGAAACAGGTTCACGAATACCATCAATATC
TACTGGGGGAGCAGCAATGAAGGCAATAATAAATACAGAAGTTGCGGTCAATAAGGTAGG
GATCATCAAAACACCGAACCATCCAATATAAAGACGGTTTTCAGTGCTGGTTATCCAGTT
ACAGAAGCGACCCCATAGGCTTGTGCTTTCGCGTCTCTCTAAAATTGCAGTCATGGTAAA
ATCTTGGTTTATTCAATTCTCAGGGACTCCCAAGCACACAGATTATCTATAAATAGAAAT
AGACAACGGAAGGCTTGTTATTCAACAGTATAACATGACTTATATGTCCGTGTCAACCAA
TAAGAGAGATATCTATCTGGATAGATCCATCCGAACGATTTGTAAATTAAATGAGTAGGG
ATTTATCCAATAACAAATATTTTTTCGTATTTTCCGTACGATTGGTAATGGGTTGCCCGG
GACTCGAACCCGGAACTAGTCGGATGGAGTAGATAATTTCCTTCTTGCAATATAATAGAT
ATAGAGTAAAAAGACCCCCCAAAAAAGCCGTGCTTGCATTTTTCAGTGCACAGGGCTTTA
CCTATGTATACATCCAAAACTAAGTTCCCTAAAAGGGGACCTAAGAAACTTGAAGACTCA
GTTGATTCAACCACTACTGTATGAACATTTCAGAATTCAAATGAATAAAATGATTTTGTG
ATTTTATCTCTTCATCATTTAGGGATCCTTTCTATTTACATGACCTCATGACCAATCATA
AATGACTGACTAGGTCATTGATACGGATAATATCCAAATACCAAATCCGTTCTCTATGTG
ACCTATGAGAAGGAGAAGAGGTTGTTGGGAAGATCAAAGAAAGAGCTTGTTCTTCCTCCG
TAAAGAATTCTTCCAAGAACTCCGAACCTAATCTTTTCAAAAAAGCACGTATCGTACTTT
TATGTTTACGAGACAAAGTTCTAGCACATGAAAGTCGAAGTATATACTTTATACGATACA
AACTCTGTTTTTTTGAGGATCCACTGTGATAATGAGAAAGATTTCTGCATATCCGCCCAA
ATCGATTGAGAATCTCAGAATCTGACAAATCGGCCCGAAACGGCTTACTAATGGGATGCC
```

CTGATACATTACAAAATTTTGCTTTAGCCAATGATCCAATCAGAGGAATAATTGGGACTA
CGGTCTCGAATTTCTTAATAGCAGTATCTATTCGAAACGAATTCTCTAGCATTTGACTCC
TTATCACCGAAGAGTTTAGTCGTACACTTGAAAGATAGCCCAGAAAATAGAAGGGATGAT
TATATAATTGCTTTATATGGATCCTGGCCGGTTGAGACCACAAGTCAAAATGACATTGCC
AAAAGTTGACAAGGTGAGATTTCCATTTCTTTATCAGAAGACGAGTCCCCCTTGAAGCCA
GAATCGATTTTCCTTGATATCTGACATAATGCATAAAAGGGTCTTTGAACAACCATAGGG
TTTTCTGAAAATCGTTACAAAGCACTACTACAAGATATTCTATTTTTGCATAGAAATGTG
TTCGCTCAAGAAAGGATCCAAAAGATTTTGATCGTAAATGAAAGGGTTGTTTACGGAGAA
AAATGAATATGAATTCACATTCATATACATGAGAATTAGAGAGGAACAAGAAGAATCTTT
GATTCTCTTTTGAAAAAAGGGAAATGGAATTTTTTGGAGTAATGAGACTATTTGAATTCC
AATACTCGTAGAGAGAGAATCGCAATAAATGCAACGAAGGAGTATCTTGTATCCAAGAGT
GAAGGGTTTGAACCAAGATTTCCAGATGGATGGGGTGGGGTATTAGTATATCTGACACAT
GATTTAAATGTGATAACTTGTCCTCGAAAAAGGGAAATATTGAATGAATAGATCGTAAAT
TATGAGATTTTGCTATTTCTTTTTCTTCTAGGGAAGATACCAATCGCAGCGAGAATGGAA
TTTCCACAACGACTGCAAAACCCTCCGATATCATTTGAGAATCAAAATGATTGTTGTGCC
CAACGAATCGATTTTGATTAGAATCATTAACCGAAATAATCAAACGATTCTGTTGATGCA
TTCGAGTAATTAAACGTTTCACAATTAGTGAACTGGATTTATTGTCATGATCTAAATTTT
CCACCGGTTCATAAAGAATCGATCCATTTAAAGCATGACCATGAGCAAGCGCGTAGATAT
ATTCCTGAAATAGAAACGGATATAGGAAGTATTGTTGCCGAAATCCATCCATTTCTAAAT
ATCCTTGTAGTTCCTCCATTTCCATTTGAAATTACACTTGAACCAAATGGGGGATTTCTT
GAGTTATCAAATAATACATAGTACGATACGGTCAGAACAAGGTATATAGTAAGAAAAGAA
TAGATACCCCGGAGCCAGAAAGGACAATCAACGGATCCTATTTCCATCCAATTTATTTAT
GTTCGTTATAGTTACAAGAGATGGTTAGAAATCCTTTATTTTTACAACCCGATCACTCTT
TTGACTTTGGAATAATGAATTTTGATCAGTATACCGGTTCTTCTACACATTCGTCTCCAC
TACATAATAGAGAACATAATAGAGAATAGTTAGGATTCATTAAAAAAAAGGAATTGATGA
TCCACTCACAAGAGAACCCTTTCCCACATCAGGCACTAATATATTTTTAACGTCTAATTA
GATCGGGTAATCATTCGAATTAAGAACAAACAGAAGCTCGTTGCTTTTGGTTTCCCTATA
ATTGGAGCTATAGGGCTCTATCCATTTATTCACTCGACCCAACTTGAATTGATTTGACCC
CTTTCCAAGAAAAGAATCAAAACAAGATTTTGTATCGATCCGTTAAGGATGAAGTATTCT
AAGAGTTCTCCATTGATACGACATGCTGTTTTTTCCTTTCATTCCCTTTCAGGATCAGTC
GTGGTCTTACAAACTCCACCAATGGTATGGACGAATCCGTTGCTTCATCAAAATGTGTAA
AAGACCATAGCCGCACTTAAAAGCCGAGTACTCTACCGTTGAGTTAGCAACCCATATAAA
TAGGGTGTGTAGATACGATCGGAATAAAAAATAAATAAAGAGATTCGATTGCCCGACCTC
GTCAAAACATTGAACTAGCAACAGATCAAAAAGAAAGATTTGATGATCAATTGTGAACAT
AAAAATGAACAGAGATCAGATGAAAATACAACAGATTCTGGGATAAATTATAGAGAAAAT
CTAAATAGATGTAAAAATTGATAGACTACCCATACTCTATTTTTTTTTTTTTTCATTATA
TTAACTAAGATACTTCTTGTGTCACAACGAAATTGACGAACCCATCGTTTGAGTGAAATA
AAGAAACAAACTTATGAAATGTGGATAAATAGATCTATTTATCCACGATCGAATTATATT
TGTTCGATACACCATTGTCAATATGAATTGAATGTTGAGAAAACCAATTCAATTTCAATA
AATAAAACAAGGACTTGTGTTGGAGTGACACTACAACATAGATAAGGGATGAAGTATGAG
TGGGGAAATAAATAAGGAATTCCGGTAGGAAAAAAATGTCGGGTTTATTCAATATTTATT
CAATAGAGGTACAATAAGCAAGATTGACCTTTTGTTTGTTGGTGGAGTCCCAACGAAAAC
CATCTGATTGATGGTAATCCCATAATTTCCCAGTTATTTCATCTATTCACTCAATCTTTT
TTCTATCTGTCTCATATCAAGAGAAGATATTTTTTTTTATAGTTCTATGATACGGGGTC
ATGTGAGAGCAACAATGAATAGAGAATAGAGAAAAAAAAATAAGGAATGGTGGAGAAACAA
TTAGACAAAGCTAGATACTGGCCCCCCCCTTTTTTTTTATTTCATTAATTTCATTTGATT
AATTCGAAATTCCTTAAATACCTCCGCCTTCTTTGAAATATCATGAACAGTTCCTGTAGG
TTGAGCGCCTTTTTCAAGGAAATATAGAATAGCGGAAACATTTGAATAAGTTTGGTTCTT
TATCGGATCGTAAAAACCCACTTTACGAAGATCTCTTCCCTCTCTTCGGGATCGAACATC
AATTGCAACGATTCGATAGATGACTCATTGGGATAGACATAAATGAACAACCCCCCCTAG
AAACGTATAAGAGGTTTTCTCCTCGTACGGCTCGAAAAAGAATGATTCGAATTTATGTAT
TGTAGTGGCAAATAGATCCACAAAATCATCAATTAGACTATGATTTGAGTCATTTTTTTT
TGTTCTTCCTTCCTGAAAAAAAAAAAAAAAAAGAAATCTCATTCGTACTCATAACTCAAG
TTGGGTAATTCTCAAAGAGCTCGAAGGGAAATCCTTAGACATTTATTGAGCCGTCTCTAA
CCTCTTTTGTTTGTCTCGCCTAGAATCGATTTGATTTCTTCCCCATTCTGATCTAGTTGT
TGAGACAATTGAAAACGGTGTTTCCTTGTTCCGGTATCCTTTATTTTATCTTTGCTTTGA
ATCCTTGGGTTTAGACATTACTTCGGTGATCCTTAATTGTTTCAAAATGGTAGCAACATA
CCTTTTGTTATTTCGTTCTATGGAAACGATTGATTCCCCTGTGATACACTTTTGATCGGA
ATTGGTAAAACTATTTCGACAAATTCATTTTACTTTTTTTTTTACTTGTTCGAACTTG
ATCCTTTCAATTTCTATACCGAAGATATACTTACGAAGTTGTTCCAACTTATTGATTGGC
ATTAACCCTAGATCCTTCTCTCTGCTAAATGAACCAATTCTTTATGCTCGAGCTCCATCA
TGTGCTATATTATAATTATATTATTTTATTACAACCCCAAAACTGGGGTCCTAGTGGAAT
AGAACAAACTATGTCGAGCCGAGAGCATCTTCTTTGATATATAAAATGGTGGGTACAAGA
ATCCACAGCCAATAATGTCCTTCAAGTCGCACGTTGCTTTCTACCACATCGTTTCAAACG
AAGTTTTACCATAACATTCCTCTAATTTTGGACCGGTATGGAATTGATTCAATATGGAAT
CATGAATAGTCATTGGCTCAATCGGTATATAGTATATGAAGTCCTCCATACTTTCATTTT

CATATATGGATCTGGAGAAGTCTCAGCAAGAATATAATTTAACCCCATATTTTATTAGAA
GAATGAAACACATTTATAAAAAACACGAAGAAATGCGTTGCTTAACACTTCTTTACATCT
TCAACAGATTGTTAGGGATGATGAATCATGAAAGATCCAATTCTTTCTCAAACAACTAAA
ACTAAAGAAGGGTCTAGATTACCGATACCGGAACAGAATGAGTCATTAACCAAATAAGCT
ATTCCAATGACCGGGAAAGATCGCAAGTGACTCGATAGGATAGATTCACCAATGTCACAC
TTCTTCGAGGAGAAAGAATTTATAAGGAATCATAAAAAACAATTTCAAGAATTTCCTACT
TCGACATCATTACTGGAAATAAGTTTTCCAGTAAAGACTGAATTGAATCTCTAAATCCAT
CAACAAGTCGGTACAACGAGGATCTAAAAATGTTTAGCAGATATCTGATTAATTAAATCA
GACGCGAATTTGATCATCATAAGAGACCTCTATGTTTCCTTTCCGATTGAATCATCAATA
ATTTAAACCAGATAAATGGGTCAAGAAACAAATCCAATTGTTTTGTTTTCTTGGGGATGG
ATAGAAGGGGCTCATGAAAGAAAAGATTAAGGTCGGTATTCTTTCAGGTATTCTTTCATC
TGATTGATAAAATCAGAATCGTAGGAGTCTGATTTTATCGTATTCATCAGATACACCAAA
CAAGTGTTACCGGGGGTAATCGTGGGTATTTAAGGGATCGCAGATCTTTTTCGCCAAAAC
TGAAACACCGGTGTCACTGATCACTGAAATAGAACAATAACAATATATATAGGCATGGTT
CATTTTCTACAGATTTTTCCTTACTTCAGATTCAGGAATCTTTCCATAAAGTAAAATGAG
TGTATGAATCTCCCCCCTCCCCCAATTGATCGCTACATTGATTTCCAATTATTCATTGGA
GCCAAATCAAATACAAAATAAAAGAAATTAGGTCCAAAGAAAATAATCCGTTCCGTATTG
AATTTTCTTGTTTGTTAATAAGATCCGGATGACAAGGGTCTTGAAATTGATACCTTCCTT
TCCTTTGCGGATTAACTCATATCGACACGAATTCCATGGGGATCATGAATCATACCCAAA
GCCAATTTAAAAGGATCTTCTTATGGGATGCTATCCTGTCTTGTATAAGTACAAAGCAAA
ATGGGTTCATATCAATTCGTTGTTACTATTTTGTTTGATTTTGTCCCACCCCAGTCTCAG
GAGCTTTAATTCCAGCGATGGAATTAATACCAAGAACCCCCCCGTTTTTTAGGATTCAGG
ATCCACATAGAATTAGTCATTTTGTCTACCCTATCTTTATTTATATTTACTTTAGGGAAA
GTAGAGACTTCTCTTTTATTTCGCATTTCGACTCAGAATGCATCATGTGAGAATCCAAAT
ATCATAGATATGGGGCATAAAGTTTATCCAAATGACTAACTAACCTTCAATATGAATATG
GGCGAGGAGGCGAACATACGCTGAATGGCCCACCCAGTTTTTTTTTTTTGAATTTCACTT
TGATCTTTGTTCCCATCCTACCTATATCAAAAAAGATATTTATTTCCATCCACATGTCAT
TGATACTCGATCCGTTTGTTTGTGAGAAACAAAATCGAAAGGGAAGATATGATTCTATAG
AAGAATCATTAGAAATCACAAAGAAAGATTGGATCACATTGTATCCAACATTACACCCTT
AAACAGAAGATTGTTAAAAAGAACAATCTTTGTTATGATAGAATTGGTCTGGGACGGAAG
GATTCGAACCTCCGAGTAACGGGACCAAAACCCGTTGCCTTACCACTTGGCCACGCCCCA
TTTTTATTTCTATTCGGCACCAATAAACACTAATATCGGTATTGGTTGTTCGTCAATTCC
AGCCCCAATATCTATTGAATTGGTTGTTGCTATGATTCTACACATGTAGATGTAGAATCA
AAATGAATTTATTGATCATTACATATAATTCAATTAAGATATTGTATGTAAGGTATGATT
CCTTCTATTCTCATTTGAGAATTGAAGGATTTTTGATTGAGGGAGTTCAAAGAAAAAGAA
AGATTTTGCGGCCTACTTTCCCTTCTTTCTTCATTTTCCCCTTATATCAATAACCCAATA
ATAATGAAATTTTCTCCAAGAACAAAATGTTTGTTATGCTTAATATCTTTAGTTTGATCT
GTCTTAATTCTGCCCTTCATTCGAGTAGCTTTTTCTTCGCCAAATTGCCCGAAGCTTATG
CTTTTTTCAATCCAATCGTAGATGTTATGCCAGTCATACCTGTGCTCTTTTTTCTCTTAG
CCCTTGTTTGGCAAGCTGCTGTAAGTTTTCGATGAGATCTTTAATACTGTCTTAGAAACA
TTCATGATTTATTCGATAAAAAAAAAATTCTATTCTTAAGAATTGATAAGATCAGATAATG
AACCCTCGACTCAAACATGGAAATTCTTTTGGATAACCGAGATGAATCGGAATCACCTCA
TTTCTTCATTCCTTCTGGGGGTCGAAGACCCTATGTATGGTCCCTACAATACCTAATTGT
AGGTATGAGAGATCATTTTGTTAACGAAAGAATCAGAATCTTATTACAAATGCATTCCTG
CAAATTCCTTATGTTTTCTAGAAACCGCTTCTTTTCTTGGTGTCAAAACGGAATATGTGG
TACAAAAATGGAGAATCTATTCCCCTATTTCCCCCAAAATGATCTTGGAGATTGTGTAAT
GCTTACTCTCAAACTCTTCGTTTACACAGTAGTGATATTCTTTGTTTCTCTCTTCATCTT
CGGATTCCTATCTAATGATCCAGGACGTAATCCTGGACGTGATGAATAAAAAAATCAGGG
GTTTTTCCTTGCTCGATTTCTGAATTTTCTTAGGATTTTCTTTCTCCATTCCATACATTT
AACTATGAGAAAGGGGGTTAGAGATTTTTTCGAATTCGAAAGGGAAATATCAAGTGATCA
GAAGAAACGGAGAGAGGGGGATTCGAACCCTCGGTACAAATAATTCGTACAACGGATTAG
CAATCCGCCGCTTTAGTCCACTCAGCCATCTCTCCCCATTTTAAATGGATAATTCATATG
TGACGCGTGAAGTAAAGGTTGATAAAAGTTTTTCCTTATCTTTCTTTATTCTATAAATAT
AGACGAATTTGATCAATCATCAATTCCCTTTAGATAATGATTCGAAACAGATATCTCCAA
TAGAAAGAGTACCTCTTTGATTTCGTCCGAAAAGTTCTTTCTTTTATTCCCCCGGCCTGG
CCAGTACCTAGCCAGGCCATTCCTTGTTCCAATGAATCATAGATCAAATGATTTATTTGA
TTTGAAAACGAAAATGCTTGTTATTGAAGCAGCAACAAGGCTATTTCCATTCCTATGATA
GGAGTGTCGTTTGTTATTATGTTTTTCCTTTTCTCGATTTACTTAAATGGAATAAAAAAA
ACATATTTTTTTCTATTATAATAGAACTCATATATTTCTTCAAAGAACATGTTTGAACCT
GAACCCTTGAGTCCACAACCAAAACAATAGGATTTTTCACTCGATCCAATCGACCCGAAT
TCATAAGATTTGGCAGTTGAATGAATAGGAAAAGGAGTAGCTTCGAAAAAGAAAAATGGA
GCTCTGGATTCTTGTACAACTCAACTCATTTTTATGTTCCGACTTCAATGGCTCTTTCGG
GCCGGGACTATCAGTAACGGCTCCCCGATAAAAGCTTGTTATTGAAATGAACCTCCTTCT
CCTATTTTATCAAGTCTCCCCGTCAGAGCACAACATGTCAGCACCCCAATTTTCATGATT
CTGATCCTATCTTGATTACGTTTCACGCCCTTGTTCGACAAATGGCCCGCTCGTATACAA
TAATTATATTGTAGCGGGTATAGTTTAGTGGTAAAAGTGTGATTCGTTCTATTAACAAGT

GAAATAGATAAGGGATCTTTCGTTTGATTCCTATTCTGATCAAAAACTTTATTTATTAAA
AGGGCATTAATCCCTTACCTCTCAATGCCACATTTGAGGAAGAATATACATTCTCGTGAT
TTGTATCCAAAAGTCAAGTCAATTAGAAATTGACTAACAAAATTGGATTATGGAATTGCG
AAGCATAATTTTTTTTTTTTGAAGTTGGATCAACCATTCCAATTGAATGAGTATAAGTAA
GGGATCCATGTATGAAGATACAAAAGTCTATTTCTAATCGTAACTAGATCTTCCATTTTT
TTTTTTTAGGGGGAGATTGAAGCCAAATAGCTATTAAACGATGACTTTGGTTTACTAGAG
CCATCGACATATTGTTTCAGCTCGGTGGAACAAAAAAATTCTTTTCTTCAGGATTCTTGC
AAGTACAAATAGGGAACGAAGTAACTAGAAAGATTTGTGAGAATCCTCCTCTTTCTAGAG
GGATCATCTAAAAAGCAAGTCATTTGGGGTGCATTCAGACGAAAAGGCTGACATAGATGT
TATGGGCCAAATTGATTTCTTTGAATTCAGATTTGCTATGACTCCCTTTTCCCATACATC
GTAAATTTTTTAGTTTTTTTTATGTCTTAGATTTGGGAATCCCATAAAGGAGCCGAATGA
AACCAAAATTTCATGTTCGGTTTTGAATTAGAGACGTTAAAAATGATGAATCGACGTCTA
CTATAACCCCTAGCCTTCCAAGCTAACGATGCGGGTTCGATTCCCGCTATCCGCTTCATA
TTAATTATTATAATACATGCTTTTGATATGTCCTTAAAATCTTTCTTTCACATACAATCC
TATTCCTTTTTTTTATAGGAGATAGGAAAGTCAGAACGTGAAAGAAATCGGAATGAAAAG
CGTCCATTGTCTAATGGATAGGACAGAGGTCTTCTAAACCTTTGGTATAGGTTCAAATCC
TATTGGACGCAATTTATTTCCATCTATTTTTGTAGATTGCTATGTCAAGAAACATATTTT
GAATGATTCGAATCGGGGCCATTTCTCAACGATTCGTCTTGTACTTAAGAGTGATCAATT
TCTTTATTTTTGTTCCTGAAGTAGAAACAGTTCTATCTGTTCCGGAATAGCTTCCTTCAA
AAGGGCTTCCGCTTGCGCGGTAAATGTCTTGGTAGAAGATATGATTTCTTGGACCTGAGG
TTTATTTGTTTTTAAGTAGGTACGTAACTGAACGAGAAATTTCTTTACCTGTCCAATTTC
TAACGGATCAAGATACCCATTCGCTCCAGTATAAATAGTAACTATCTGTTCTTCCACCGT
GAGAGGGGCTGATTGGGATTGTTTGAGCAACTCGCGCAATCGTTGACCTCTTGCCAATTG
ATTCTGAGTGGCTTTATCGAGATCAGAAGCGAATTGTGCAAAGGCTTCTAACTCTGCGAA
TTGAGCCAGTTCCAGTTTCGATTTGCCGGCTACTTGTTTCATGGCTTTAATTTGAGCTGC
AGATCCTACTCTCGAGACAGAAATACCCACATTAATGGCAGGACGGATTCCGGCATTGAA
TAGATCAGCGGATAAGAATATTTGTCCATCTGTAATGGAAATCACATTAGTAGGGATATA
AGCCGAAACGTCCCCAGATTGAGTCTCAACTATTGGTAAAGCGGTCATACTTCCTTCACC
TAAACGAGAACTTGATTTAGCGGCTCTTTCCAAAAGGCGTGAATGCAAATAAAAAACATC
TCCTGGATAAGCTTCGCGGCCGGGCGGTCTTCTTAATAGAAGAGACATTTGACGATAAGC
TTGTGCTTGTTTGGAGAGATCATCATAAATTATTGAAGTATGTCGTTCACGGTACATAAA
ATATTCAGCCAGAGCCGCTCCTGTATAAGGAGCGAGGTATTGTAATGTAGCAGGTGAATC
CGCCGTTTCGGCTACCACAATAGTGTATTCCATTGCCCCTCGTTCCTGGAAAGTGGTCAC
TACCTGGGCCACGGAGGATGCTTTTTGACCAATAGCTACATAAACACATATTACATTTTG
CCCCTTTTGGTTGAGAATAGTATCTGTGGCTACTGCTGTTTTTCCGGTCTGTCTGTCCCC
AATAATTAATTCTCGCTGACCGCGTCCTATAGGGATCATCGAATCAATAGCAATAAGTCC
CGTTTGAAGAGGCTCATATACGGAACGTCTCGAAATAATACCTGGAGCAGGAGATTCGAT
TAACCGAGATTCAGAAGCTGAAATTTCACCTCTCCCATCAATGGGTTTAGCCAGAGCATT
TATAACACGACCCAAATAAGCCTCACTAACGGGTATCTGAGCAATTCTTCCTGTTGCTTT
TACAGAACTTCCCTCTTGTATCATCAAACCGTCACCCATTAATACAACGCCAACATTATT
TGATTCCAAATTCAGAGCAATGCCTATTGTACCCTCTTCAAATTCTACTAATTCCCCTGC
CATTACTTCATCAAGACCATGAATACGAGCAATGCCGTCGCCTACCTGAAGTACTGTGCC
GGTATTCACAATCTTGACTTCTCTATTATATTGTTCAATACGTTCACGGATAATATTACT
AATTTCGTCGGCTCGAATGGTTACCATTAGTGTCTCTTAATTCTTTTTCGGAAACAAAGA
AAAAAAAAAAAAAAAAAATAATGCCTACAGTAGAAGGGCTAATCAGTTACTTCTTTCATG
GCCCCGAGCATGCCAATATTAGCACCGATGGTGCGTAAATGTAACTCGCTGTTTGAACAA
CTATTCAGAGTTCCTAGAGCTCCTTGTAAGGCTTGTTGGAAAACTCGTTGTCGCACCTGA
TTAATTGCTCTTTGTTGTTCAAAATGAATGGTTTCATTTTTGTAATTTTCTAATCGTTCC
AAATTCTCATAAGTGGCATTAATCAAATTCCATTTTTCTCGTTCTATCTCAGAGTATCCA
TTCACTCGAAACTCATCTGCTTCTATTTCCACTTTCCGTAAGCGAGCCCGGGCTTCTTCG
AGCTGCTCAATGGCTCCTTCACGTAGTTCTTCTGAATTTCGAATAGTACTCAAGATCCTC
TGTTTTCGATTATCTAATAAATCACTTAATGAAAGTAGATTATCTTCCCATTCATTTCAC
AACTTCATTTCCATGATCTCTTCCCGAACCAAACATGAATCTTTCGATTCATTTGGCTCT
CACACTCAGTTACTTAATGGGTCATTCCATCTATTTTAATGTAATGAGCCTACCCTCTCT
TCTCTGTTCGTATTCCAAGATATCAAAACTGATACGAGACCAGAATATTCGGAGGACTCT
TCCGACCCGACAAAAAATCTGTCATTGTCAGCAAAGTTGTTTCTTTTTTTTTGTTTTC
TTCAAATCCAAAAAATTCTTCTTATTTTAGACATAGGTCATCGATTCAACATTGGATAAA
AAAGGGCGAGACACCCATTTTTACAGTAAATGGTTCAAATCATTTTATCGATATGAGTGT
TCTATATCGGATAAATTGCCAACTATTCATTTTTTCGAAACCATCTCCGTACTAACGTAG
TGGTAGAAAGAGTACCATGTTGTGCCTGGACTTCAAACGGTTTCGCTTTAACCATGTTAA
AGGTCCCACATTATTGGCTGATAGAGAATCAAAGTTGATTTACCAATAAATTACGAAATG
CTATGGTTCTTACATATGATTTCTTAATTTATTCAGAAGTAATTCGTCGAGATCGTGCAC
CTTTCTTTCCTATTTATAACTTTCCCATTCAAAAAAAAAAAAAAAGGAAAGTGCAGCCGGT
TGGATCCAGCCTATTCTTGAAATACACAACTCGCACACACTCCCTTTCCAAAAAAGATCA
ATACACCAAGCACTACACTTAGATTTATTAGATTTGTTGCTAAAATATCGGTATTAAACC
CGAAACTCCCAGCGGATGGCCAGTGACCAAAGGAAACGAAAGAATCGGTTACATCTCTCA

TATGCTTTTCTCTTATAGATAGGACCAACAAAATGGAACAGAGTTCTTTTTGTATCACTT
CGCCCCCTTTGTTTTGGATTGATTTCTTTTTATTAATTTCCTTATTATAAATATGAATAG
ATTCATTTTAAGAAATATTTTTCTTTATTATTATTTCCATGGAAATTCTCAATAATCTAT
TTATTTAGTCCCAGGTTTCATGTCAATTACGAAATACCTCGTTTGTTGCAACACTTCCTA
AAAGTAAAAAAGAGTTTCCATTAAGAACGGAAGGGAAGAAAGCAAGTGGGTCTGCTATGC
TAATTCCTCATCCTCAAATCACTCCATCCCCGGGGGTATTGTCTCAACGAAGAAGTGATT
GTAGGAGTGAAGTTTGGATATAATTCGGCAAGGCAAGCCCGCGGCAATAAAATAGGAAAA
GAAAATAAGTATTTATTTTTCACATTTATAGGATTAAACAAAAGGATTCGCAAATAAAAG
CGCTAATGCCACGACCAGTCCGTAAATTGTTAAAGCTTCCATAAAAGCCAGACTAAGCAA
TAAAGTACCTCGTATTTTACCCTCTGCTTCTGGCTGTCTCGCGATACCTTCTACGGCTTG
GCCCGCAGCAGTACCTTGACCAACTCCAGGTCCAATAGAAGCAAGTCCCACAGCCAATCC
AGCAGCAATAACGGAAGCGGCAGAAATCAATGGATTCATATTAAGTTCCTCGCACCAAAA
AAAAGAAATGGTTAATGATACAATCAACCGATGAATTATTACTTCATTATTCCATCACTT
AAGATCTATCCGAAAAAAAAAAAAGAACTAAGAACTCTGAATTGAAATAATAATATTACTG
AATCATCAGAGCTACTTCGATATCTCGTTTTTAGTTCCTATCCGTGGAGTCTTTGTAAAT
CTATACGGTTCCAGTTCTTCCATTTCTTTGTTCCGAACCATTCCATTCTTTCAATTCTTC
GCTCTTTCTCTTCTATATGCATGCTTGACTTCATTTGTTTATTCATTCAATCCACAGTCA
CAAATAAAACGGAAGGGCTTGCATTGGAATCCGTCTAAATTCAGTGGGATGGGAAATAAT
ATATATGGATAGCCCATATATAACTAGTGAATATCTAATATCACATATACATTGTTTCTT
TAATAACGTAAACCATCCGTATCTTCTATTGAACCGGATTCTAGAATCATTCTTCGAAAC
ATATACAGGGATTGGCTTAGAGCCCTTACATATACCCAGCTCGACCCCCCTTACTTTTTT
TGAATTCTCTAATATCATACATTTTTTTTTTGCTCCTATTCTAGATCGTATATACCGGTA
TGAGTTGGGATAAGCTTTTTTTTAAGACCATTTCGGAAGCTAGTCAATGATGACCCTCCA
TGGATTCACCTATATAAGCTGCGGCTAAAGTTGCAAAAATAAGAGCCTGAATCCCGCTTG
TGAATAATCCAAGGAACATGACAGGTATAGGAACCACCGAAGGTACTAAAGAAACAAGAA
CAACAACTACTAATTCATCCGCTAATATATTCCCGAAAAGTCGAAAACTAAGTGATAAGG
GTTTTGTGAAATCTTCTAGAATGTTAATTGGTAAAAGTATTGGAGTTGGTTGAATGTATT
TACCGAAATAACCCAATCCTTTTTTGGTAAGACCCGCATAGAAATATGCCACTGACGTAG
GTAAAGCTAAAGCAACAGTAGTATTTATATCATTCGTGGGTGCAGCTAACTCTCCATGCG
GTAACTGTATGATTTTCCGGGGTAAAAGGGCACCTGACCAGTTAGAAACAAAAATAAATA
GGAACATAGTTCCAATAAAGGGAACCCAAGGACCATATTCTTCTCCAATCTGAGTTTTGC
TCAAGTCTCGAATGAATTCGAGGACATATTCGAAGAAATTCTGACCGTCGGTTGGAATGG
TTTGTGGATTCCGAACAGCTATAGTGGCTGAACCTAATAAGATAGCAATTACAACCCAAG
AAGTGATAAGTACTTGGGCATGGACTTGGAAACCCCCTATTTGCCAATAAAAATGTTGGC
CTACTTCCACATCGGATATATCGTATAACGCTTTTAGTGAGTTGATGGAACAGGGTAAAA
CATTCATATTGCCCTCTAACATAAATAGAACTTAAAAAGGAATTATTTTGATTCAGCCAT
CTCGTATCTCTTTCTCAACTCGTCTACTTTGAATCAATCGTATATTTCGGATCCCAAGTG
ATCACATAATATCCCCAGTGATTTTGATCTCTTTTTTGAGACTCAGGGATAGTAACCGAT
TCAATCAATTTATGGAGTTTCCAAAGTGATTGACTTATCCTAATCAACAATTTCTTATAT
AGCTAGAACTGCCCTCACAAATTGCGGATACTAATTTGTTAAGAATCCATCGGATTGAAG
CCATAGCGTCATCGTTCGCTGGAATCGAAATATTTGCGAGATCCGGGTCACAATTTGTAT
CGATTAAACAAATTGTTGGAATCCTCAAAGTGAGACATTCTCGAAGAGCCGTATATTCTT
CTTGCTGACCAACGATGATTACAATATCGGGTAACCCCGTCATATATTTGATCCCGCCCA
GATAGGTTTGCAAGTGAGATAATTGCCTCTTCAACATTGCTACATCTCTTTTCGGGAGAC
AGTTGAGTTTCCCCGTATTTTGTTCCGATCTCAAGTTTCTGAACCTATGAAGTCTCATTT
CTGTAGTGGACCAATTCGTTAACATACCACCGAGCCATTTTTTATTAACATAATGACACC
GAGCCCTTATTGCAGCTGATGCTACTAAATTGGCTGCTTTATTTTTGGTACCAACTATTA
AGAAGTGTTTTCCTATACTTGCTGCATCAAAAACTAAATCACAGGCTTCTGACAAAAAAC
GAGCAGTTCGAGTAAGATTTGTAATATGAATACCTTTACGCTTTGAAGAGATGTAAGGTG
CCATTCTAGGATTCCATTTCCTAGTACCATGGCCAAAATGAACTCCTGCTTTCACCATCT
CTTCAAAATTAATGTTCCAATATCTTCTTGTCATTTCTCCCCACATTTTCTCTCTTTTTT
TTTATTTAAGAGGTACCCCTGAAATAAATAATTGTTCCGACGGAACCTTCTACCGGAGAT
TGACCGTTAATACCCAGTCCAAGTCATTAATTCCTTTCTATTCGTTATTATCTTTATTAC
CAAATCAAATGACCAGGACCCTATAGTTAAAAGAAAAGAATGAATCTGTCATTAAATCCC
GTAAATGATCGTTCTGATGTATCAGGGAAATTATTTGGGATGCAAGAACCAAATAATTCT
CTATGGTGGAACAAAAGATCTCTCATTTCCTCCTCGAATGGATTCTTCTTTTTGATTTCC
AAAGGAATGTTGCTGTGTTGCCTTGAGCGGTGCACTAATCCTTTGAATCCGGTACCAACA
GGCATCATCCCCCCCAGAACAACGTTTTCTTTCAGGCCTTTCAACCAATCAATACGACCT
CGTAGAGCAGCTTTTGCTAAAACTCGAGCGGTTTCTTGAAAACTCGCTTCGGATATGAAA
CTTTGAGTATTCAGAGATGCCCTCGTTATTCCCAATAAGATGGCTCGGTAACAGATCGCT
TCTTCCAAAGCACGCCCTGTTCGTTCCGCTCGCAACAATCCGATTAGTTCTCCAGGTGAA
AAAACATTAGACATTCCATCTTCTGAAACCAACACTTTTGATGTTATTTGACGTACAATA
ATCTCTATATGCCTATTATGGATCTGCACCCCTTGGGATCGATAAACCTTTTGGATCTTA
TTAACCAAAGAGATACGACTTTGCGCTATGGTTAGTTCAGCGCCAATCAAGAATCCCCAA
GGAATTCCAAGGATTCTTGTTATACGTTCGTTCCAACCCTCAACCCTCTTTTCTAAGTTC
ATCGATATTGAATCAATCGAACGCGCTTCTAACACTTGTTCCACTTTTGGAAGACCTTGT

GTTATATCACCCGATCTCGATTTTTCATATATAAATGTAACTAATGTATCTCCTTCGTAA
AGGATTTCTCCACAATGGCCATGAACAGTTGCCCCTGGCGTAGCCAAATGGGGCTTAGCG
GATCTTATGACTAAGGAGTCAACATGAACAATTAGAACTTGACTCGATTTTATGTGTGGT
CCGCATTTGGATATACATACATTTTCACAAATAAACTGTCCAAGGCTAATTATTGTGGAT
GTCTCCTCACAATAATCGTGAGGGAGAAAGCACCAATTCAAATCGAATGGATTAAAAATG
ATGTTACTGCATGAATCGGGATTATAAATTCTCCCATTTTCATCCATTAAATAATATTTA
AGCCCTTGGAAAGTGTGTTTGAAATTGTCAAGTAGAAAATATTTCTTTAACAAGATCTGA
TTATGAGTTATTAAATAGAAATAGTAAAATGAATAAAAATTCGTAATTTGAGGTACAATC
CCTAAAGGACCCAACGAATTCCTAATGGGAATCGCGGGATCCTCTTTAATTAATTTAATT
AATTCTTTGTGAGATTTCGAACCATTGAATAGACCAATTCGAGAACAATCGGATGATAAC
AAAATTAGAAAAGATGGACATTCCTTATTTCTATTCAGCAACGTACGAATAGTCCCTTGA
TGTTGGGTAAATGATTGAATCCTCGCCTTGAAATAAAAAGGATTAATATTGGTGCGATCT
GATCCATTATTGGCAATCAATCCTGAACTTGCCATATCATTCCTTTTTCCGATATAAAAA
ATAGAGGACTTCACTAAATCAATTCTTATGAAATCTCGAATCAGATCATTTGCCCTTACT
TCAACAAAGGAAGCATGAACCTCTTCTATAGAACCATCTCGGTCTTGGTTCCAATTCAAT
ACTAAACAAGTCCGAACTAATTGAAGACTTGTGTGATAAATTCCCCGAATCGGTTTGCCA
TTTCCATAAAGGATATAATTGACAATTCGTAGTTGCACATTATCCCTTTCCTGCAACGGA
TCCTGGGGGAAAAGCGTTGCTAAATTTATCCCATCAGCTATTTCATATGTGACTACGGGT
CGAACTGAAACAAAATACTTTTTCTTGGTAGGTGTAATCCGTTGGACATAAATCCAATTT
TTCAATTTTTTTGATTCCTTAGAATTTTTTTTTCCCGTTCCTGGTGGTATCAAGATGCCG
CAGTGCCGGGATATCTTATCTGTCGCTCCAGGAAAATAGATATCCCCAGAAAAGATTTTG
AGTTCAATCCTTTTTTTTTTTCTCTCCACCCGGACCAATCCGCCTACTTGGCTTCTTGTA
TTTAAAGCGATTCGTGTATCTACTCCAATGATACTGTTGTTCCGTACCATTATGGGCGAA
GAACCGGGTAAGATATGCACTTCTTCGGGAATGAAAAAAAATCGATCTACTTTCATTTGG
TATTTTGGCCTAAATTCTTTTGCTCTTCGATACTCAATCAAATCCTCTTTTTTTACGATT
GAACCCACCCCTATAGTCCCATATTTAGTAATTCCTGAACTGCTTCTTCTGTATAGGGGA
TCGTCGAAATAAGCAAGAATACTATTTCTAGGTAAAACACCATTTATGGGTATTTCAATC
GAGATACCGGAACGGGGCATTAGTTCTTTTTCTCGTTCTTGATCAGATTGGAATGGGATG
ATAAATCTATTTCTTCGCCTCTTAGCCAATAAATCAGAATTCTCGTGGAGAATGGTAGGA
TATATGAAATTCCAATGACCGTTGGATATGATTCGATCAGGTCCTGAATAATCAAGAACC
CCCCCCTTTTTACCGTAAGGATTCGAACTAAACAATTTGTGTCTCACTTGATCATTAGTC
ACGGAGAGGTCAGAAATAGATCTCCGTTCAACAGAATGAACATTCATTTGATCTTGATCC
TTGTGGAGCGAAAAAGGCACTATACTGGATCTGCACAGAGCTCCTGATAATATCCATAAA
TGACTTGTTTTGGGTAAGAGATGAACATTACCATATTTATATTCAGGTGCATGGTACACA
TCGGTACTCCAGTGCATTTCTCCCTCTGAGTCAGAATAAATATGTTTTCGAACTTTCTCT
TTAACTTTATTAAAATTGAAAGTGGATGTTCCAGCGCGAATCTCAGCAATCACTTGTTCT
GATTCTACATATTGATCATTTTGAACTAAAAGAAAACTTTTGGGTGGAATATTCACATTA
TGTAGAATATCGTGACTCTCAATAGTTACATACAGGTCTATATAACATAGAAAAGCAGGA
TGCCCATGACGTGTACGTGTGGGATGAACCAAATCCTCATTGAATTTGATTTTTCCCTTA
AAAGGAGCTCGTACATGTTCTGCAGTACCACCTGTGAATACTCCACCGGTATGAAAAGTT
CTTAATGTTAGTTGAGTCCCCGGTTCGCCGATTGATTGACCCGCAATAATACCTACTGCT
TCTCCTAATTCGACCAGGTCGCCATGAGTGGGACTCTGACCATAACATAATCGACAGATC
CAAGATATACTCCTGCAAAGAAAGGGGGTTCGAATATATATTGGTTGTGTTCGAAAGGTT
ATGAATCGAGTGACAAGTCCAACCCCAATATCTTTATTTTGAGCGGCAATGCAACGTGGA
CCCATATATATATTGTATGCTAATACACGACCAATTAGTGTTTGGACCCAAATTCTTTCC
GTCATCCCATTTCTAGGACTCACGGAAATGCCTCGGGTAGTGCCACAATCTGTTCTACGT
ACAACAATGTGTTGAACTACTTCAACAAGTCTACGCGTGAGGTATCCAGCATCTGATGTT
CGTACAGCAGTATCCACAACTCCTTTGCGGGCTCCGTAGCAGGAAATGATATATTCCGTT
AAAGAAAGTCCTTCGCGTAAATTGCTTTGAATCGGTAAATCAATCATTTGTCCTTGGGGA
TCCGACATTAATCCTCTCATACCTACTAATTGGTGTACCTGAGATGCATTTCCTCTAGCT
CCCGAAAAGGACATTATATGGACTGAATTAGAAGGATCAGTCATCCTAAAATTAGGATGC
ATTTCTTGTCTCAAATATTCACTTGTAGCATACCATATCTCAATGGATTGGCGTAATTTT
TCTACTGTGTGTACATTCCCATAATGATGGTGCTTTTCTAAAATGAAACTTTGTTGTTCA
GCATCTTGGACTAACCACCCCTTAGAAGGTACTGTTAAAAGATCATCAATTCCTAATGAA
ATGGATGTAGCAGTGGCTTGCTGGAAACCCAGAGTCTTTACTTGATCCAGGGTGTGCGAT
GTATATGCCATTCCGAAGTGATCTATTAATCTGCTAATAAGTCGTTTCATGGCAGACCCA
TCTATCGCTTTATTGTAAAAGAACAGATCAGCCCATTCTGCCATAAGTACTTCTCTATTC
CGCTGAGTAGGATTCGCCAATGGGTTTGAGTCAGTGATTCGAAGACCTCCTTTACTGGAT
CTCGATTCATGTAGAAATTAAGGAATTATGATTCCAGTTGAACCGGAGAGATCCAAATTC
CCGCGGTATTACATAATTCCTTAGCTTAGGTACCGTATGAGTAGGCCTGACAAAACCCCT
GTATGGCTTCTTCTATTTCTCGAGAAAAAGAAATAGGACCAACAGTGGTTCGGGTGTATA
TACAAAGGGTTTGTTTTTTTATACGTCTTACTATTAGATAGTGCCCATAAATTTCATGAT
AGGTACCCAAAGATTCATATTGAACTTCGACAGGAACTTCTCTTGAGGCAATGACACGTT
GATCTAGTCGCCACCGAAGCCACAAAGGACTATCTAAATTGATTCGTTTCTGCTGATAAA
CTATAAGTACATCATAGGAACTACAAAAATAGGGCTCTTTCTCTTTCGTAGTATATTTAT
ACTTATAATCATTAACTGTTTCATTTTGATAGTTTATGCGATTCCATGGATTATACCTAT

TTGCACAAATACCTCGACGATTCCCGATCGTTAATACATAGAGTCCAATAAGCATATCTT
GGGTTGGTACCGAAATGGGATCTCCAATAGCCGGAGAAAAGAGATTCATATGAGAAAACA
TAAGTAAACGAGCCTCCGCTTGCGCTTCCAAAGATAAAGGTACATGAACAGCCATTTGAT
CCCCATCAAAGTCTGCATTGAATCCTTTACGAACTAATGGATGTAAACAAATAGCACGTC
CACCCCCTAAAATGGGCTGGAACGCCTGTATGCCTAATCTATGCAGGGTGGGCGCTCTAT
TCAACAATACAGGATGCCCCTGCATAACTTCTTGAAGTATTTCCCATACAATGGGTTCTT
TTTCCCGAATTTGACTTTTAGCAATTCCTATGTTAGAAGCGATATGTCGTCTGATTAAAC
CACGAATGACAAATGTCTGAAAAAGTTCTATTGCTATTTCTCGAGGTAATCCACATCGAT
GTAATGAAAGCGAAGGGCCCACGACAATGACGGAACGCCCCGAATAATCGACCCGTTTAC
CAAGTAGAGTCTCACGAAATCTTCCTTCTTTGCCTTCAATTACATCTGAAAATGACTTGT
AAACTTTATTATGACCGTCTCTCATTGGTTGTCCGCGGATCCCATTATCAAGAAGTGTAT
CCACGGCTTCTTGTACCAATTTCTCCTGACACATTACTAATTCCCCTGGCGTAGATCTAC
TTGTTGTTAATGAATCGGTAAGAGTATTGTTCCGATAGATAACTCTTCGGTAGAGTTCAT
TAATATCCGAACTCATTAGTTTACCCCCATCTATCTGAATGATTGGCCTCAACTCGGGAG
GAAGAACTGGTAATAGGCACAAAACCATCCGTTCTGGTTCTACATTTGTTCGAATAAAAT
GCTTAGCTAATTCCATGCGTCTAACCAAAAAATCCTTTCTTCTTCCAATTTTTCTATCTT
CCCATTCATTCCCAGTGAACCCTTCTTCCCCTAATTCCTTCCATTCTACCGATGAATGAT
CTATAATAATTCGCAAATCCGGATCGGCTAATTGTTCTCTGATAGCACCTGCTCCAGTAG
AGATTTCTCGATTTCGAAATGTATCGAAGCCTGGGGTAGTAAAAAAAAGTGGGATGCTGT
ATTTCCGGGATTGGATTTCAGATTCGAATGAACCTCGTAATCGCAAGAAAGTCGGTTTTT
TAGCTATGGGCCTGGCAAAAGAAAAATTGGGATAGGTTCCTATAGGATCCCCCCTTCAAA
ATCGGACGTGATGGTTTCCTCTCATCCGGCTCAAGTAGTTACACCAAATAAAGAAAGGGG
TTCTCCACTTTCAAATTTTGTTCTAGAAAATCCCAGAAAGATCTACTCCTTACTCAAGTT
CCCAGTGAGGACCAACAAAAATATTTCATGGATTCATCCTTCCTTTGACTTTTCTGAATT
ACTTATTCAATTACGATAAAAATGGAATGTCAAATTATTGAGTAGTCTACTTCCCTTCGA
ATGATAAATCCCCTTAAATGAAAGGAATACCTTGGAATTAATAAGGGATTTACTTGTCTA
TGTATTGTTTCATTCGATCTTTTAGGTCTCCACTTCACCTCGACGGTTATGCCATAATGT
CCCTTGAAGCATATATGCGATGGATGGGCTCCTGTAACCGTGTCATATTTGTTTATTTGA
ACAGAATCTCTTTCTAAAAGAAATAGAACGTCTAATTCCACGAAAGAAGTTTTTTTTTT
TCACGAGGTACAACTGTTATATGTTACGGAATCGACCATGGATCAATTCCCCTTTCATTT
GGAAGTATTGAATACAACCATAATTCTGAGCTTCATATTCCTCCTCCCAAGATACATGTC
AGAGTCAGGGGCATCCCCATCGAATTGAATGGGATGACAGTTTCTCATTCCGAATCTGTA
AAATCCTAATTTCGATCAAATCACACATCGCAGTATACTAGGCCTTCTAATTCCTTAAGG
GGTTTATCTAAAAGATTCGCGATATAACTAGGAAGACGTTTCAAATACCATACATGAGTC
GCTGGACATGCCAGTTTGATGTATCCCATTTGATATCTTCGTATCCGAGAATCAACAAAT
TCAACTCCGCATTGTTCACAAAATTTCGGATCTTCTTTTTCCTCTCCGATCACTCGATAA
TTTCCACAAGCACAAATTCCGCTTTTTATAGGTCCAAAAATTCTTTCACAAAACAATCCA
TCCTTTTCCGGTTTATTGCTTTTGTAATGAAAAGTATAGGGTTTTGTCACCTCTCCAACC
ATCTCTCCATTAGGTAGGATTTTGGTGGCCCAAGCACTTATTTGTTGAGGAGAAACTGAT
CCAACTCGAAGTTGTTGATGTTTATATCGGTCGATCATAGAAGAAAAATTCTGATTCATT
TCGATCAAACTTCCTTCCTGTTAATCTGGAAGTTCTTCTCAGATACAAGGAAATGATTCA
GTTCCAGAGCCAAAGATCGTAGTTCTCGAACGAGCAATCGAAAAGATTCTGGCGCATCCT
CGGGTTTAAGTATTGTTCCGCCAATGATTGTAGTTCCAAGTACTTCCTGACGAGCTCTAA
TATGATCCGATTTATAAGTAAGCATCTCTTGTGAAATATGAGCAACACCAAATCCCTCTA
GAGCCCAAACTTCCATTTCTCCTACTCGTTGTCCCCCTTGCTTGGCCCTTCCCCTAAGGG
GTTGTTGTGTAACAAGTGCATAATGCCCGCTGGAACGTCCATGGATTTTATCATCAACTT
GATGAATTAATTTCAGGATATAGGACTTTCCTATTATAACCGGTTGTTCAAAAGGATCTC
CTGTTCTTCCATCCAATATTCTGCTTTTTCCCGGATACTCGGGTTCAAATACCCACGGAT
TTGCTGTTTGCTTACTGGCTGAATATAATTCAGGAAACACTAGCTTTCTCGAAGCCCCCT
GCTCATATCTCTCATCAAAGGGTGCTATTCTATAATGTCTGTCCAACAGGTACCCCGCTA
AACCGAGCGAACATTCAAATATTTGTCCCACATTCATTCGCGAAGGTACTCCTAATGGGT
TGAAGACCATATCGACCGGTGTTCCAGCTTGCAAATAAGGCATATCCTGTCTAGGCAAAA
TTTTGGAAACGATACCCTTATTCCCATGTCTTCCAGCTATTTTATCACCTACTTTGATTT
CACGTTTCTGTAAAATATATACACGAATCGTTTCTGGATTATAACTGGAACCCCCCTTTT
TCTGGATCCATCTCACATCAATAACTCGACCCCTTCCGCCTATAGGTAGTTTTAGACAAG
TTTCCTTTGCGGTGGATACCTGAATGCCAAGTATGGCTCGTAATAATCTATCTTCCGGGG
CATACGATGATTCTTTCGCCGCCTGAGGCGTTAATTTACCCACTAAAATATCACCTCTTT
CTATCCAAGATCCCAGCATTACAATTCCATTTTTGTCTAAATTGCGGAGTAAATGGGCTT
CTAAATGCGGTATTTTATTAGTGATTCTTTCGGGGCCTTGGCTTGTCACATGAGTCTGAA
TTTCATATTTCCGTATGTGAAAAGACGTATAAATATCTCCATATACCAGACGTTCGCTAA
TGAGTACCGCGTCTTCAGAATTGTAGCCTTCCCATGACATATGAGCTACTAATACATTTT
TTCCCAAAGTAAGTTCACCGCCAACTGTAGCCGCGCCGTCCGCTAAAATTTGTCCCTTTT
TAATGCATTTACCCCGCGGAGCCTGAGGTTTTTGATGCATACAAGTATTTTTGTTGGAAC
GTTGATACATAACCAACGGAATGCTTATAGTGTCTCCATTACCCGATAAAACGATCTTGT
CCGTATCGGTATAAATGATCTTTCCCTCGTGTTCGGCTATAGCCGAAACCCCCGAATCTA
GAGCCGCTTGGCATTCCAACCCAGTTCCAACAATGCACTTCTCGGATCGAGAAAGCGGAA

CTGCTTGACGCTGCATATTAGAACTCATTAAAGCCCGATTTGCATCATTATGCTCGATAA
AGGGAATGAGGGAAGCTCCAATAGAAAAATATTGGAAGGGAAAAATGCTTCGAAGATGAA
TCTGTTCCCATGCAATAGTCAAGAATTCTTGACGGTATCCGGCTGGAACAACCTGTTCTT
CCTGAATACCCCGATTCAAAGCCAAAGAATTTCCTGCCGCTACCATATAGTATTCATCTC
TACTTGGTGATAAATAAACCATCTTTTTTGATCTCTCAGATATTTCATAAAACGGACTCT
CTATAGACCCCCAATGACCAATCCTCGCATGAATAGCTAAGGATCCAATAAGTCCAACAT
TGATTCCTTCGGACGTGTCAATTGGGCAAATACGTCCATAGTGACTAGGATGGATATCTC
GTATCCGAAAACTAGCAGTTCGCCCTGTTAATCCTCCAGGACCCAAATAACTCAATTTTC
GCCCATGAACTATTTGTGTCAATGGATTAGTTCGATCCAAAACTTGAGATAAAGGGTGTA
GGCCGAAAAACGATTCATAAGTGGTTGTTAATGGAGTTGGAGTTACCAAATTATGAGGAG
TCGGTATCAATTTATGCCTGATTGCTCCACATATAGTTCCTCGAACCGCATTTTCTAAAC
GAACCAGAGCCAATCCGAATTGATCCTGTAACAGATCTGCTACAGAACGAATACGTTTAT
TTTTCAAGTGATTCATGTCGTCAAGTGTGCCCATTCCAAATTTCATTCCGATCAAATGAT
CCACAGCAGCCAATACGTCTTGTGGTAACAAGAATGTATTGTTCGGAGGTATATCAAGAT
TCAGTCTCCGGTTCATATTTCGTCGACCAATCCTTCCTAATTCACATCTTTGTTGAAAAA
ATTTCTTTTGTAATTCCTTACATAAGGACTCAGAAAATACCGGATCCCCGCCTACACAAG
CAAATTGTTGATAAAACTCCAAAATGGCATTTTCCCTTGACCCAATCTTTTTTTTCTCCT
TATGATTCGGGAAAGACAAGAAAATTTCAGGGTAGCAAACATTATCTAGAATTTCTCTTA
GATTCGAACCCATAGCCGATGATGGAACTAGGATAGATATTTTTTGTTTCCTACTCACAC
GGGCCCATATCCTTGCTTTTCTATCAATCTCTAATTCTGATCTTCCCCCCCAATCTGATA
TTATGGTGCCGGTATAGACAGAAATTCCGCTATGGTCCAATTCTGAACGGTAATAAATAC
CGGGGCTTTGCAATATTTGATTGATTACAATTCTGTATATTCCACTTACTAGAGAGGTTC
CCAGGGAATTCATTAGAGGAATATTTCCAATAAATACGGTTTGTTCTTGCATATCTCTAC
CGGTTTTCCAAATTAATCCCGCGGATACATATAATTCAGAAGAGTATGTGAGTGATTCAT
ACACAGCATCTCTTTCTTTTATCAAGGGCTCTGCCAATTGATATGTTGCCACAAATAATT
GAAATTCAATTTCTTGGTCTGTATCTTCAATTTTTGGAAACTTATGAAGTTCTTCCATCA
AGCCTTGATCAATGAACCTACAAAATCCGTCAAATTGTATCTGACTAAACCCTGGTATTG
TATACATTCCCTCATTTCCATCCCGGAACATCTTAAGTTTTCCGTTTATCGAAAAAATCC
AACTATTGGCTCACTCTTCGTTGAACCATATAGATTGATCTAGCAACGATGGAATGTATA
TTTTGCTCATTTGAACAACATGAAATTTTATCCAACCCCATATACATATATACATGTACT
AAATACGTATGAACGGAGGAATAAAAAAAAATGTGACTCAAATTCGAATTTGCGACAGAT
ACAAATGGAAATGAATTGATAAAACATTCCTGGAAACAAAATTCTGCCACTTAGACTTAT
GGAGTCTTGTATAGAATATCAAAATAGATCCAATTTCTACCTTATGATATTACGATCAGA
TTGGGTACCATAAATGGATTCGGAATTGAATCTGTTCTCTATGAGTGAGATAAAGACAGA
ATAATCAGGAACCGTCTAGAGTTGACTTTGATTTAGCACTTAATTTATTTCATCGATTC
CGTTGTTCAAAAAATGATTCGCAGAGAGAAAAGATATTTCTACCCATTTGTTAATTAGTA
GAATACGATTGAAGTGCGTAAGAGAAGTCATATTTATTAAGTACATGCAGATATAACTAT
CTAGCTATCCGTATATCCCATCTTTTATCGAAGTTCCCTTGAGGCAACATAGGTCGTGCT
ATATCCAAATTTCTATTTTATATTCAATATATTCAATGAAAAATGCAAGCACGACGATTT
CCTAATAGGAATATGTAGATAAGATACCTGACTAGGCATTTGTGTAAGAATTTCTGTACT
GGGGTTTACATATACACATAATTGTTGTTATAATTGAAATTGAAAAGGATTAATTATGGA
AAAGAATTGGGACTGATTAGTCGTATATCAATTTGATCTCCTTATGTCATTAAGGAAACC
AAATTGGAGATCAAAATCCAAGAACCATTCATGAATTCACAGTCATTAATGCTTCCAATT
TGTTCTGAATTTTGGATTCTGTGACTGGAAATCCATTTTTCTCTTTCAATAGAAAAATAG
GGGAAAGCTTTTATTTAAGTGTTGTGTGTTTTGAAATACAATCAATCTAAGAGAACAACA
GGATCCAATCAAAAGAAAGAAATGGTTCAGCAATTCCCCAGAATATTTCCATCTATATCT
ATTTTGTATCGTTTTGGCGGCATGGCCGAGTGGTAAGGCGGGGGACTGCAAATCCTTTCT
CCCCAGTTCAAATCCGGGTGTCGCCTGATTAACAAAAGACTCGGAATTTCTTACCCTACT
AAACTAATAGAACTCACAAAATTCTTGCCTGGCAGAAGCAGAGGTAAGGGGCGGGGACTG
TCGATACCCAATTTTAAGAATCGGGGGGTTGACTTTCAATTATTTCTTCAAAAATCGGGG
TGTGACCCAAACCTGTACCATACCAATATGAATTACCAATATAAATAAAGAAATACTCAC
TTAATTACGGATTGCTGATGCGTTCAGGCCATTGATTTGATTTATCAATCGAATCAAATC
CATAGTAAGATTCAATTGTGAGATTCAGAACTACGAAAGTCAGGGACCGTAAATCCGTGT
ATCCAAAGGAAGGTTCCTAAGAGACTGTAAATCCTTGCTCCTAGGATCCAAGAAGGGGTT
CTAGGAAGGACTATAAATACTTCCTAATTACCTTACCCTACTAGTGTTTACTGACTGAGT
CTTGAAGTAGATTGGGTAGGCTGGTGGGGAATCCAATTAGGAGTTGTGGAAAGAACTGAA
GATACTTTGTATCCATACAAACTCATGAGAGTCTCTGAGTGCTCAGGTTTTCAATTAATA
TTGTATGGGTGATTGGCTTTTATAGAATAAAAGTGGAAAAAAGTGCTTTCGTTGGGGTAA
CCCCGCCAAGAATGTAATAGGGTGTCTTCCAATTGTTTCACATTTCACAGAAGTAGAGAC
AGTAAGGCTTAGATGGGAAATTAGGAGTATGTGATAGATAGTTATATATCTTGATGGTAT
ATTTTTTTTTTCTGCTTTTTGTTTATGAAAGGCAACAATAGGTCTTACTATTCCTACATA
TTCCATTAGTCACATTCCCTTGAGACTTCCAAGGGGCAACTGTGTATCTTGCTTGTACTT
AGTGCTTTCCGATTCCACCAGAAATCATATAGGGACTTGTTACGGGTGTATTCATTGGAT
TGGTTCATCAAAAACGTTAGGTCAAAATCCCATTTTGACTCTGCACCATTGATTCCACTA
TTATTAGTGATCAAGAATGGAATAATTCCTTCATATTCATAGAGATAGGGGACACGATTC
ACATGGATATAGTAAGTCTCGCTTGGGCTGCTTTAATGGTAGTCTTTACATTTTCCCTTT

CACTCGTAGTATGGGGAAGAAGTGGACTCTAGGGGTACTACTAATTGAGTTGAGTAATCG
AATTTATCAATTGTTTAATAGATCGTTCTGCAAAGCGCTTTTAAATCAAAATATCTCCA
CCTCATAAATTCTACTGGAATCCAATATGAATAAGAACCTTTCGATCAAACAAATATTTC
AACGACTTGATTCCCATATTCGTATTTCGAAACTCAAAGGGATACACATGATGGGAAATT
TTTTCCAACCGAATTCTTTCTAAATATTCTATTTCGACAAATCGGCCCTTACTAGAATTA
TGCATATTACAATGAGGAGCAACCAACCCCTATTTTTTTTTGATTTGTTTCCCTTTTCT
CTTTGCTGTTCAAAGAGGGAACCGTTCTTCTATTACGTACGTGGATATGTACTTTCTACT
GAGGCGACATAGACATAGTGGTTGTCCAAAGAGGTACTACGCAAAATAAGATCTTACTTT
CGTTGGGTATGCGTACTTACCTTTTTATACTCCTAGGAATCTTATTTATGCTTTATCGAC
TCGTCTCATGTCATGGTTCAAGCATGAAAAATCGGTGGGGTCTACTACATCCTTTTCAAA
ATCCGAAGAAGTTACTATAGAACTTTTTGGATCATCCGTTAACGGATCAATCAATTACTT
CTTCGTAATGCTAAAAAAAGGCTTGGTTTTCTTTTATATAATATATGCCCATACTAGTCT
TTCTCCATTGATTCTTTCAATGGATCCCCGGATCCATATTGAAAATAATCAGAAACCCAG
GAATTAGAAAAGTTGACGTTCGATTATTTCAGATTGATCGGGATCAATACAAATTGACGT
AACAAAGAAATAGAATTGGAGTGCTATTTACATGTACATATATAAATGTGGGTACATATT
GTGGATTGATCTATATCAAGCTCATACCTTTCTACAATAATAGATAGTGTGGTAGAAAGA
ACTATAATGAACCTTTCTACCATACTATCTATTATACTGCTGACTCCAACCCGACCATTT
AAGACTTGGAATTTGAATCCCTTTCTTTCATTTCTTCAATCGTTGATAAGAACTAATAAG
TCAAGTTTCAGTCAAATTAATCACTTTGACTGACTGTTTTTACGTAGATTATAAGTAAAA
AAGCAGTAGGAACTAGAATGAACAACGCAATAGCAATAAATGCTAGAATATTGACTTCCA
TAATCTCATCGTTTTTTTTTGCTTCGCAATAACTCGGGATCTAATCCCATAGAGATGATA
AGTCTTTCTCCTGTAAATTCAATAGGATAGATTGTATCCTGATGATACTTAATCGTATCA
ATATCATGAATAACAATATCTGATCTATCAAATCGATTCATCGTCGAGAATTGAATAGTA
TAACATAGGAAGATCTTTTATCCATACCGAATCCAAAATTGGATTCCTGGTCCAATCAAG
AATCCCATTGAATTTCTCATTTCCACTCTTTCTTTTTTATAACCTGCCGTCTTCCTTATA
CAATCATCTGGCCGGCGTTCCATTGGTCACAAACCCAAACGGTAGGGATGAAATGGAAAA
AGGAATGAGTTAAGTTCTAAACGAAGTTTTTGTGAAGATCTACTCTTTTTGGAAGACAGA
GAAGTGTGATAAAGATTGGTCCGGTAGAAAAGATCTAACAGAATATTCTATTCTGACAAA
TTCATTTATTTATTGATTTTGTTTTTTCTTCGATGGGGCCATTAAAATAGGAAGAAAAAA
AAAAGGGGGGGGTAGGTTTCATCTGAAAAGTACTCTGTCGCTGTCGGGGTAACTAGTA
ACTATACTATATTAATTAAAACTATATTAATTAAATTAATTGCGTATCATACAATAAACG
AATACAATTTGTGTATGTGCTCCCGGGAAACGTATGAGTACTCTATTACATGGACCAGGA
GCAATCGAAAAAGACAGGCCCGTAGGGTCTCTCTTGAAATCTGAATAGGGCGATACCGCC
GATCAATCTACATATGTCTCTCCCCATCAATCGGTACTAGTTGAAGTAATTGAAAGTCCC
ATATTTGTACGATGAGAAATGCGAAACGAAAAACACGAAAGAAATAAGGATCCCCGGGGG
ATTAAATCCTGCTCCTTGTCCCCCCTCTTCGCAGAAAATGGGGAGATGAGTTGATGGATT
CATCGGATTCTAGGTCGGGACTGACGGGGCTCGAACCCGCAGCTTCCGCCTTGACAGGGC
GGTGCTCTGACCGATTGAACTACAATCCCGGGAAATGGGGTGTACAGCATACATACATAT
TCTTATAATTTCATTCGAACCCTTTCTTTCTATTCTATATTAGATTGAAAATCGACATCT
TTCTGTTACAAGAAAGACGAGTGATATACTGATATACACATGGATATGGACTATAGTGGG
AGTGACACGGATTACTAGTAATCCTGTGGTTATTTTATTACCCAATCAATTGATAATCCA
TTTTTCAATGAAAAAAAAAAAAGGACTCTTTATTTCTATCTATCAGGCATTTCATTTAT
AGAGGACAAACTGGTTATATCATCCTCATGGATCGGCGAATTGTTGGGCCGAGCTGGATT
TGAACCAGCGTAGACATATCGCCAACGAATTTACAGTCCGTCCCCATTAACCGCTCGGGC
ATCGACCCAGGAAGAATCAATTCTAGGCTTATTGATAATCCATGATCAACCCCCTTTCGT
CTTACCCCCAGGGGAAGTCGAATCCCCGCTGCCTCCTTGAAAGAGAGATGTCCTGAACCA
CTAGACGATAGGGGCATACCCGCCCGATCGCCATCATACTATCTATGCTCATAGTATGAG
CAGTTTTTTGAAATTGTCAATATACAATATATATGACTAGATCCGAAGAATCTTTCTTGC
TTACAAGATTCCATAGAATGGAATTTTGGGATTGTTGATTCATGAACCATCCTATATATA
AGAGAGGATAGGATCCTTCAGGGAGTGATTTGTCCGACAGAAAAAGGGCAAACCCCATTC
CATTTCTTTCATTTTCACTCGTTGATTCGTTCGTCGTTAAGGTGAGATATGCCTATCTCA
CACTAACACTAAACTAAGCCAGGAAATTCAGAAACGATAGAATTTCTTTTTTTGAGGATC
GACGAATAATCGAAAAGATTCTTTTTTTTTTTTCTAATAATTTAATTTAGGGTACGAATC
GAATCCCTTCATCACATGATTCGATGAAATACCTTGGATCTATATCGGATTGGTACATGT
ATCAATCAACCAAGCGAATCTCGTCCGGATGAATCAATAAAAGCAAAGCAATTAGGCGCG
TCCCTGAAACAATTCATTGCATTGATATTTCTCAAATATCAATAACTAAAACTTCCTAGG
TAAATCAAATTTATTGTTCCTGAATGAGCCCCTATGTATACATGTACATTATATACATAT
ACATTGTAGTACATACATAGATATATGTAGTAGACTCTATAGTAGCTAGTGATTAATTCA
TTTTTTGAAGAAAATGGGCCCTTTTAACTCAGTGGTAGAGTAACGCCATGGTAAGGCGTA
AGTCATCGGTTCAAATCCGATAAAGGGCTTTTTCTACGAAGCTCCAGTCTTCGTCTTCAT
TTTTCATTGGAGAATAGAGATATTGTTGATATTTGTAATAAAAGTAACCCATAATGAGTT
ATCATTCTAATGAGTTATAGGTATAAAGTGAAACAGTTGTTTATTATGATTATGATAAGT
AATCGTACTTAGTAGGAGGACTACTATGTAATTCACTACAAGCTATACCCCTCCTCATAT
TATTCCTATTTTTGGTCCTGGGACATAGATATTCTAGATACCCAATCCAAATTGTGAATC
GCCAAACCAAAGTATTCCCATTTCTCTATTGTTCCAATCAAATCCCTCGGAAAAATTAGA
AATCAAGAAAATCAAAAAGTAAGTGGACCTGAGCCATTGAATCATGACTATATCAGCTAT

TCTGATATTCAAATTCGATAGAGATGAAATTGTAGAAGCGAACTTTTTCTTTCCTTGGAC
CACGCAATAATTTGTCGATATTTCAGATTGAATCTTCTTGTTCCTGGATGCTCCATAGGA
ATAAATTGCTCTTCCTTTCCTCCACAGAGATACGTTTATTCCAAGTCACAAGAGCAATCT
CTTTTTCAATACCTTTCTTTGATTCCAGAAAAAAAGAAGTTTCTATCTATATAGGATTTA
GATATAGATATCAAATCATGGCTTCAGGTACAAAATATTTCCATATTGATGCATCAGATA
TTTTTGTTCCTCCAATGCAACGGAAAACGAGTGCGATAAAGGAGGGATTTTGATTTCCAG
TCTCCCTATTTAATTTAGGGGGCAGGGACAAAAAATGGGGTCCTTTTTTTTCTGCCGGAT
ATAGGGTAATAAAAAAAAGTAAAGAGGGAAATATTCGAAGTTTATTTTTTTTGGTTCGAC
CCGCGAAAAGATATACTCTGGAATTTTAGATTCATTCGAAGGAAATATAACAAAGAAGAC
AATACAAAAAGCAATCAAAAAAGGAAGGAGTAAGAAATTATATATATATATAGGA
TACTGTAGTAGTTTAGTATACACATAAATTACGAGAATCCATAAAGATATTTATTGATCT
TTTCTCAATAAGATCCAAGAACAAGAATACGATTAGCTTATGGAATGGCGAGCTAGATCT
GGGGAGCAACTGATAACGAGAGAAAGGATCGCTTGTTTCCTCACAGTTATTTCAAAAAAT
GGATCTGATTGATGGGTCATAAGACAATTCAGGGTTCGGATGGTTATTAAGAATAAGAAG
GAATAAGGAATAGGAAGGAATAATTGAATCGAACTCATGGATTTACCTAGGTTGGTTTCT
GGCCCAATAGAAAGGAAGGATTTGTATCTTCGAAACCCATTGGATGGAAGGCGCAGTGGA
CGAGGAATCGTTCATAGATGACCGAACCATCGTATGCCCTGAGAATGATATGAGGTGTTC
GGAAATGGTTGAAGTAGTTGAATAGGAGGATCGATATGACTATAGCCCTTGGCAGATTTA
CCAAAGAAGAAAATGATTTATTTGATATTATGGATGACTGGTTAAGGAGGGACCGTTTCG
TTTTTGTAGGTTGGTCTGGTCTATTACTCTTTCCTTGTGCTTATTTCGCTTTAGGCGGTT
GGTTCACAGGTACAACCTTTGTAACTTCATGGTATACCCATGGATTGGCCAGTTCCTATT
TGGAAGGCTGCAATTTCTTAACCGCTGCAGTTTCTACTCCTGCTAATAGTTTAGCACATT
CTTTGTTGTTACTATGGGGACCTGAAGCACAAGGAGATTTTACTCGTTGGTGTCAATTAG
GCGGTCTGTGGACTTTTGTTGCTCTCCATGGAGCTTTCGGGCTAATAGGTTTCATGTTAC
GTCAATTTGAACTTGCTCGATCTGTTCAATTGCGACCTTATAATGCAATCGCATTTTCTG
CTCCAATTGCTGTTTTTGTTTCTGTATTCTTGATTTATCCACTAGGTCAGTCTGGTTGGT
TCTTCGCGCCTAGTTTTGGTGTAGCAGCTATATTTCGATTCATCCTCTTCTTCCAAGGGT
TTCATAATTGGACGTTGAACCCATTTCATATGATGGGGGTTGCTGGAGTATTGGGCGCTG
CCCTGCTATGCGCTATTCATGGTGCTACTGTAGAAAACACTTTATTCGAGGATGGTGACG
GTGCAAATACATTCCGTGCTTTTAACCCAACTCAAGCTGAAGAGACTTATTCGATGGTCA
CTGCTAACCGCTTTTGGTCTCAAATCTTTGGGGTTGCTTTTTCCAATAAACGTTGGTTAC
ATTTCTTTATGTTATTTGTACCAGTAACCGGTTTATGGATGAGTGCTATTGGAGTAGTCG
GTCTGGCCCTGAACCTACGCGCCTATGACTTCGTTTCCCAGGAAATCCGTGCAGCGGAAG
ATCCTGAATTTGAGACTTTCTACACCAAAAATATTCTCTTAAACGAAGGTATTCGTGCTT
GGATGGCGGCTCAGGATCAGCCTCATGAAAATCTTATATTCCCTGAGGAGGTTCTACCCC
GTGGAAACGCTCTTTAATGGAACTTTAGCTTTAGCCGGTCGTGACCAAGAAACCACTGGG
TTCGCTTGGTGGGCCGGGAATGCACGACTTATCAATTTGTCCGGTAAACTACTCGGGGCT
CACGTAGCCCATGCCGGATTAATTGTATTCTGGGCCGGAGCAATGAACCTATTCGAAGTG
GCTCATTTCGTACCAGAGAAACCTATGTATGAACAAGGATTGATTTTACTTCCCCATCTA
GCTACTCTAGGTTGGGGAGTAGGTCCGGGTGGGGAAGTTATAGACACCTTTCCATACTTT
GTATCTGGAGTACTTCACTTAATTTCCTCTGCAGTCTTAGGCTTTGGCGGCATTTATCAT
GCACTTCTAGGACCTGAGACTCTCGAAGAATCCTTTCCATTCTTCGGTTATGTATGGAAA
GATAGAAATAAAATGACTACAATTCTGGGTATTCACTTAATCTTGTTAGGTATAGGTGCT
TTTCTTCTAGTACTCAAGGCTCTTTATTTTGGGGGCGTATATGATACCTGGGCTCCCGGG
GGGGGAGATGTAAGAAAAATTAGCAACTTGACCCTTAGCCCGAGTGTTATATTTGGTTAT
TTACTAAAATCGCCTTTTGGGGGAGAAGGATGGATTGTTAGTGTGGACGATTTAG
AAGATATAATTGGAGGACACGTATGGTTAGGTTCCATTTGTATACTTGGTGGAATTTGGC
ATATCTTAACCAAACCCTTTGCATGGGCTCGCCGAGCATTTGTATGGTCTGGAGAG
GCCTACTTATCTTATAGTTTAGGTGCTTTATCTGTCTTTGGTTTCATTGCTTGTTGTTTT
GTTTGGTTCAATAATACCGCTTATCCTAGCGAGTTTTACGGGCCCACCGGGCCGGAAGCT
TCTCAAGCTCAAGCATTTACTTTTCTAGTTAGGGACCAACGTCTTGGGGCTAACGTGGGA
TCCGCTCAAGGACCCACTGGTTTAGGTAAATATCTAATGCGTTCCCCGACCGGAGAGGTC
ATTTTTGGAGGAGAAACTATGCGTTTTTGGGATCTCCGTGCTCCCTGGTTGGAACCTCTA
AGAGGTCCCAATGGTTTGGACTTGAGTAGGCTGAAAAAAGACATACAACCTTGGCAAGAA
CGACGTTCGGCAGAATATATGACTCATGCCCCTTTAGGTTCTTTAAATTCCGTGGGTGGC
GTAGCTACCGAGATCAATGCAGTTAATTATGTCTCTCCTAGAAGTTGGTTAGCTACCTCT
CATTTTGTTCTAGGATTCTTCCTATTCGTGGGTCATTTGTGGCATGCGGGAAGGGCCCGT
GCAGCTGCAGCAGGATTTGAAAAAGGAATCGATCGTGATTTTGAACCTGTTCTTTCCATG
ACCCCTCTTAATTGAGACAGGAGATCAAATGCATGAAGTAGGAATCCATTTGATTCCATT
ATACATATTAGGTTAAGATCAGGTCATATTTAAAAAGTATTCCTTGTTTTTTCCTTTTCA
TTCTATCTATTTTTTTCTGGCTCGGCTATCCCACCTAGCCGAGCCATTTCCCTTTATGAC
ACCGGGCCAGGCCATACCAATAAAGAAACAAATCGATTCAACGAGCAAAAGGAGAGAGAG
GGATTCGAACCCTCGATAGTTCTTTGTTCGGAACTATACCGGTTTTCAAGACCAGAGCTA
TCAACCACTCAGCCATCTCTCCAAGAGACAATCTCCATTTTATTCCTCCGAATAGAACAT
GGCCATATGGGTTGATACTCTAACTATCTGTAGAAACATCCCAAGTGCGAATCTATATTT
CGACATATCTATCTGTCTATAGATGCATGATCCAACATGCCCATTTGGGAAGTCAAAAAA

AAATTCCCTGATTCCATGTCCGAATAAAATAAAGTGGGACTAAGTTCGAAAGGATCAATA
AATTCATGGTCAAATCCCGTCATGATGCATTATTTCAATTTTGACTCTGAGAGAGGGATC
AAATGGTATAGTTCATTTGTTGGTAGCTTGGAGGATTACAAGCATGACTATTGCTTTCCA
ATTGGCTGTTTTTGCATTAATTGCTACTTCATCAATCTTACTGATTAGTGTACCCGTTGT
ATTTGCTTCTTCTGATGGTTGGTCAAGTAACAAAAATGTTGTATTTTCTGGTACATCGTT
ATGGATTGGATTAGTCTTTCTGGTAGCTATTCTTAATTCTCTCATCTCTTGAACCTATTC
GGTCTTTCCCGGATCAAAAAACTGACCCCTCCCCAAATTCTTTCGGATTGTAAGACACAT
TAAAATGAAATATGAGTCCAAAAATAAAAATAAAAAAATTGGAGGGAGGGGTCAAAAATC
ACTTCTTGAATAAAAAAAACGAAGAATCTAATAATAATTGGAATCTTCCTAAGTATCTGA
CCCTGTCTGTACAAATGGGATCCAGACACATATATGATATATCATATATGTGTGGACATA
TACGTGTGTATCAGGAACGAAGAAAGTGCGGATATGGTCGAATGGTAAAATTTCTCTTTG
CCAAGGAGAAGATGCGGGTTCGATCCCCGCTATCCGCCCATGGTAAAGTAAGGTAATATG
ATAAATGATTTAGGTATAGTTGACCACGATAGGGGAGTGGTTCTATTCTTCCCATCCCAA
AACCAAAATAGCCATTGGTTACTAGGTAACGGAATCGCACCTAAAAATGTTTTTTTGAAA
AAAAAAAAAAAAAGAGATGTTGCGGAGACAGGATTTGAACCCGTGACCTCAAGGTTATGA
GCCTTGCGAGCTACCAAACTGCTCTACCCCGCGCTGAAAACTAATGGACGAACAAGAATT
GGATGTGCCCCCATACCATATTCTATATAAATAGAATAGCCCATTTATACAGAATGGTAA
AGGGGGCCCCTCTATGATCATAGCTCATAGAGATAAATAGAAATAGGAAGAAGGGGTATT
TTTATCCTTACCAACTTGATCTTGTGGCCCCCGGCAACAAACATGCCTGAACCTTTTCGT
GAAGTATGTGTCCGGATAGCCGAAAGTCTCGATAGCTAGCTCTAGGTCTTCCGGTCAAAA
AACAACGTCGATGAAGACGTGTAGGTGTACTATTACGTGGTGGGGATTGCAATTTTCCAT
GAATTTCCCATTTGTCACTCAACGATGAAACTTTGCCTATTTCTTTTTTTGAGGATCGAC
GAATCAAATGATATTTCTGTTCCAATTTCTGCTTCTTTCTCTCCCTCTGAATCAAACTTT
TCCTTGCCATAAAGGTTCAGTTCCTATTATTATCAATGATACGGGTCGGATCCTAGATGT
AGAAATAGAAGAAGGTGGATTCTCCCTTCTCCATCGAATCAAATGAAATTGTCGATGATA
CAGCACATTAAAAAAAAAAAATTAACCAAATTTGCCTGATGTAGAGGCAATCAAGAAAGCT
GCATAAGTGAATATATAACCTACAGAAAAGTGGGCTAATCCAACCAATCTTGCTTGCACA
ATGGAAAGAGCCACTGGTTTATCCCTCCATCGAATCAAATTAGCCAAAGGCGTGCGTTCA
TGAGCCCATGCTAAGGTTTCAATCAATTCCTGCCAATATCCACGCCAGGAAATTAAGAAC
ATAAATCCAATAGCCCAAACAAGATGCCCAAATAGGAACATCCATGCCCAGACCGATAAA
CTATTCATACCAAAGGGGTTATAGCCATTGATAAGTTGTGAAGAGTTTAACCATAGATAA
TCTCTTAACCATCCCATCAAATAAGTGGAGGATTCGTTAAATTGTGAAACGTTACCCTGC
CATAATGTGATGTGCTTCCAATGCCAATAAAAAGTAACCCATCCGATAGTATTTAACATC
CAGAAAACCGCCAAATAAAATGCGTCCCAGGCCGAAATATCACAAGTACCGCCTCGTCCC
GGACCGTCGCAAGGAAAACTATAACCGAAATCTTTTTTATCTGGCATTAACTTGGAACCA
CGTGCATCTAAAGCACCTTTTACTAAGATCAATGTAGTTGTATGCAAACCTAGAGCAATA
GCATGATGAACCAAGAAGTCTCCAGGACCTATTGTTAAGAATAGTGAATTACTATTCTCA
TTAACGGCATTCAACCAGCCCGGTAACCATATACTTCGACCAGCATTGAATGCCGGGCCA
TTCGTTGAAGATAAAAGTACATCGAACCCATATGAAGTCTTACCGTGAGCGGATTGTATC
CATTGGGCAAATATGGGTTCGATCAAGATTTGTTTCTCCGGAGTACCAAAAGCGAGCATG
ACGTCGTTATGAACATAGAGTCCTAAGGTATGGAACCCTAGAAAGAGACTGGCCCAACTT
AAATGAGATTTGATAGCTTCTTTATGGTCTAACATTCTTGCCAATACATTATCCTCATTC
TGTTCCGGATTGTAATCTCGAATGAAGAATATAGCTCCATGAGCAAAGGCCCCTGTCATG
ATGAATCCTGCGATGTATTGGTGATGAGTATATAACGCAGCTTGAGTAGTAAAGTCTTGT
GCTATGAATGCATAAGCAGGTAAAGAGTACATGTGTTGAGCTACCAAGGAAGTAATAACC
CCTAAAGAGGCTAGAGCAAGGCCTAATTGAAAATGAAGCGAATTATTGATTGTGTCATAA
AGACCCTTATGCCCACGTCCCAATCGACCCCCCGGAGGAATATGTGCTTCTAAAAGATCT
TTCATACTGTGCCCAATCCCGAAGTTAGTTCTATACATATGACCAGCAACGAGAAAAATA
AATGCAATAGCTAAATGATGATGAGCCATATCGGTCAGCCATAAACTTTGCGTTTGTGGA
TGGAATCCCCCGAGAAGGGTTAGAATGGCAGTTCCCGCCCCTTGGGAGGTACCAAATAAA
TGACGACTGGAATCGGGGTTTTGGGCATAAAGATTCCACTGACCTGTAAAAAGTGGGCCC
AACCCTTGAGGATGGGGTAATACATCTAAGAAATTATTCCATCTGACGTACTGCCCCCTT
GATCCTGGAATAGCGACATGAACTAAATGCCCTGTCCAAGCCAAGGAACTGACTCCGAAG
AGTCCTGACAAATGATGATTGAGACGAGATTCGGCATTTTTGAACCACGAAACGCTTGGT
TTCCATTTGGGTTGTAGATGTAACCAACCCGCAATTAAAGATATGGCAGAAAGAAATAAT
AGAAAAAGAGCTCCAGTATAAAGATCTTCATTGGTGCGTAAGCCAATTGTGTACCACCAC
TGATAAACACCGGAGTAAGCGATATTCACTGGGCCGAGAGCCCCTCCTCGAGTAAAGGCT
TCTACAGCCGGTTGACCAAAATGGGGATCCCAAATTGCATGAGCAATAGGTCTTACATGT
AAAGGGTCCTGTACCCATGACTCAAAATTTCCTTGCCAAGCTACATGAAACAGATTTCCG
GAAGTCCACAGAAAGATTATTGCTAACTGCCCGAAGTGAGAAGCAAAAATGTTCTGATAA
AGACGTTCCTCAGTAATATCATCATGACTCTCAAAATCATGTGCGGTAGCAATACCAAAC
CAAATACGACGAGTAGTGGGGTCCTGAGCTAAGCCTTGGCTAAACTTTGGAAATCTTAAT
GCCATAATGCCTTTCAAATCCTCCTAGCCATTATCCTACTGCAATAATTCTTGCTAAGAA
GAATGCCCATGTTGTGGCAATTCCACCCAGAAGGTAATGGGTTACTCCTACAGCGCGTCC
TTGTACAATGCTCAAGGCTCTAGGCTGAGTAGCAGGAGCAACTTTTAATTTGTTATGAGC
CCAAACGATGGATTCAATGAGTTCTTGCCAATAACCACGGCCGCTGAATAGAAACATTAA

ACTGAAAGCCCAGACAAAATGAGCACCTAGGAAAAAAAGGCCATATGCCGATAATGAAGA
ACCATAAGACTGAATTACCTGAGATGCCTGTGCCCATAAAAAATCCCGGAGCCACCCATT
AATAGTAATGGAACTCTGTGCAAAGTTTCCTCCCGTAATATGAGTTACCACCCCTTGATC
ACTTATACTACCCCAAACATCTGACTGCATTTTCCAACTGAAATGGAATATTACTACCGA
AATAGCATTGTACATCCAGAATAGACCTAAGAAGACATGATCCCAGGCAGATACTTGACA
TGTCCCCCCTCTTCCAGGTCCATCACAAGGGAAACGAAAACCAAGATTTGCTTTATCAGG
TATCAAACGTGAGCTACGAGCAAATAGAACACCTTTCAGTAGTATCAATACGGTCACATG
GATCGTAAATGCATGAATGTGATGTACCAAAAAATCTGCGGTTCCTAATGGAATAGGTAA
CAAAGCGACTTTGCCGCCTACTGCTACTAAATCACCACCCCCCCAAGTCAAGCTGGTGCT
TGTTGTTGCACCAGGAGCTGTTGCACCAGGTGCTAAAGCGTGGGTGTTTTGTACCCATTG
AGCAAAGATGGGTTGTAATTGTATAGCGGTATCTGAAAACATATCTCGGGGACGCCCTAA
AGCGCTCATGGTATCATTATGAATATACAAGCCAAAACTGTGAAAGCCTAGAAATATACA
TACCCAGTTGAGATGTGATATGATTGCATCTCGGTGTCTAAGGACACGATCTAATAGATC
GTTGTATCGAGTAGTTGGATCATAGTCTCTTACCATAAAAATGGCTGCATGTGCAGCAGC
ACCAACTATGAGAAATCCACCGATCCACATGTGATGTGTGAACAACGAAAGTTGTGTACC
ATAGTCAATAGCTAGGTATGGATAGGGGGGCATGGAATACATATGGTGAGCTACAACAAT
GGTTGAAGAGCCTAACATAGCTAGATTAAGAGATAATTGAGCATGCCATGACGTTGTTAG
GATCTCATAGAGGCCTTTATGACCCTGGCCCGTAAATGGGCCTTTATGCGCCTCTAAAAT
ATCTTTCAGGCCATGACCAATGCCCCAGTTGGTCCTATACATGTGACCAGCTACCAGGAA
AAGAATTGCAATAGCTAAATGATGGTGTGCAATATCTGTCAGCCATAGACCCCCTGTTAC
TGGATCTAATCCTCCACGAAAACTAAGAAATTCCGCGTATTTTGACCAATTCAAGGTGAA
AAATGGGGTTGCTCCCTCGGCAAAACTGGGATAAAGTTGAGCCAAAAGATCCCGATTCAA
GATAAATTCATGAGGAAGTGGTATCTCTTTAGGATCCACTCCAGCGTCTAGAAATTGGTT
AATCGGTAAAGATACATGTACTTGGTGTCCCGCCCAAGAAAGAGACCCAAGCCCTAGTAA
CCCCGCTAAGTGGTGATTCAACATGGATTCCACATCTTGGAACCAAGCCAATTTTGGGGC
AGCTTTGTGATAATGGAACCAACCGGCAAAAAGCATTAAGGCTGCAAAGACCAATGCACC
GATTGCGGTACAATAGAGTTGTAATTCATTAGTTATTCCAGATGCTCGCCAAAGCTGAAA
AAAACCAGAGGTTATTTGTATTCCTCGGAAACCCCCGCCCACATCACCATTCAATATTTC
TTGACCTACTATTGGCCAAACAACCTGGGCGCTGGGTCCAATGTGAGTAGGATCACTTAG
CCATGCTTCATAATTAGAAAAACGGGCGCCATGGAAATACATACCACTCAGCCAAAGAAA
AATGATGGAGAGTTGACCGAAATGAGCACTAAATACTTTTCGAGAGATCTCCTCCAAATC
ACTGGTATGGCTATCGAAATCGTGAGCATCAGCATGAAGGTTCCAGATCCAAGTGGTAGT
ATCAGGGCCCTTAGCTATTGTTCTTGAGAAATGGCCGGGTCTGGCCCATTCCTCGAAAGA
AGTTTTTATGGGATCCCTATCCACCAAAATCTTCACTTCTGGTTCCGGCGAACGAATAAT
CATTGAGTCCTCCTCTTTCCGGACAACACATACAAAGAAACCCGCCAACAGTCAAGTAAT
TAGTGAACGATGGGTATTTATGATTAGTTCCTTATCTTTCCTATCCCCCATCTATCTTT
TTTTTTTTAGTTATTTACTAGAGCAATTATGATATGGAAGTCGATCCGGGGCAAGTGTTC
GGATCTATTATGACATAACCATGGGGCGCTCAACGGACCTTTATAATATTTTATAACCCC
CTCCGGCGTGACACAAAAACGGATTTTTTGATACAAGCTAGTTAGTGTATTCATATCTCA
ATGTATAGTATCTAGATGGATCTACTTCATATCTTACACGGAACATATTACTTACAATAC
AAATCAAAGGATCATTCATTAGTCATTAATAAGAGACATCTTGATATCTATATTTAGTCA
TTCGAGGTCTGTCTTTTTTACTGGCTTAGCTTTATTAGCATAGCAGAAGGGAATATTTTC
TGTACTGTATCCGTATCGTTTATCCCTATGACAGACGAAATAGAACAATCTTAGACTTAG
AAGGGATATAATGAAATTCCCTGATTGGCTCTTCCTAGAGGAACGATCTATTTTATTTGA
TTGATGGATCCCATATTATAATGAATTCAAAAAGAGAATGTTCTTATTCAAACCTCCTTG
CGATCTTCAACCAATTATGTGCTTCAATATAATTCCCCGGAGTAAGCGCTATAGCTTGTT
TCCAATATTCAGCAGCTTGATCGGACCAAGCCTCTGCAATTTCAGAATCTCCTTGTCGAA
TGGCCTGTTCTCCCCGGTCGGAATAGGTGGGTCAATTCCTTCCCTTAGAACCGTACTTGA
GAGTTTCCTACCTCATACGGCTCGACATTCTTTTGGTGTTCCATCTTAATCTACCATATC
TAACTGAATGAGATTTCTCATAAATCTATCCCATTTTTTTTTTTTTCGGGTTAACCAGAAG
AGGTTAATTACACGAGTTTCAAACTCTAATTTTGATCAATAATCAGTTTTCTCTTTTCTC
CCACCTTCAGAAGAACCAAGCATAGGTATTTTCCTCTATCGTTCGAATTTTCTGAAAGGT
AACTATCTCGGTTTCATATAGAAATTCATATAGAATCTTTGAAAAAGACTTTCCTCCATA
AGAAAGAAAGGACTTACTATCTTTGGGATCTGATGCTACACCGCTGCTCAATACCTTAGT
AGATCGACTCTATTACATAAGTTGATTCCTAACTTTTATCTCATATCATGACATTAAGTA
AGCAGTCCTTATTGTATCGGTCCCCGAACCTCACTAATTGATCTTTACGGTGCTTCCTCT
ATCAATTAGATCCTTTATCCATAGAATAAAGTATATAGGCCATACCTATTTCTTCATATT
TCGGCTCTTATGAAGTCTCTTTCTTTGCTACAGCTGATAAAAATCGTTGCTTTGGACGAT
GCATATGTAGAAAGCCTATTTTGTTTCTAGTATTGACTAGCGGATTTGGTCTTTCCTTCC
TTCTTTCTATAGTGTAGATAGTCGCACGTAATGACAGATCACGGCCATATTATTAAAAGC
TTGTGGTAAGAATGGATTTCGTTCTATTGCCCGGAAATAATATTCCAAAGCCTTCGTGTG
TTCTCCGTTACTTGTGTGGATAAGGCCTATGTTATAGAGTATATAACTTCGATCATAGGG
ATCAATTTCTAGTCGCGTAGCTTCATAATAATTTTGTAAAGCTTCCGCATAATTTCCTTC
GGATTGAGCTGACATCCGTTACGGTCGTTCATTCTATTCAAAGAATCTCCGTTCCAGAAC
CGTACGTGAGATTTTCATCTCATACGGCTCCTCCCTTCTGTGCATAGTAATAAGGGGAAT
AATCCATGGAATCAAAAAAGATTGAAATATTCTCATTATGAACTGACAGGGGCTGGTGTT

TTTACAAGAAATCTCTAGCCAGCCTTCCTGCAAGAGGTCTGTCTTTTCTTAACACCAAGC
GCGTTTGTGCTAGATAGAAATGGTAACTCCAACAATTTCTTTGTCCTCAACGCCCCCTGT
TTCCAGGAATTAGTCACTTCAACGACCTTTGATGGTTATACGGGTATCCAAAGTACGAAC
GAGATGGATGTTTGTTGTCCCAACCATTCTTGTTAGTTCCGATCCCGATAAGGAAAAGGG
TTAATTTATAACAAAGTTTTCGTGTTGTTGATTTCTAGATGTAGTGCTTCTTCCCCTATG
CGGCCTATTGGTACTAGTGGAGTAGGATTGACCCGCAATACAGAACCTATAGGTGTAACC
TTTCGCTCAATACTAGAATCGACAGTTGAAGCATCTAAGGCTGCATCAATCGGGGATACA
CGACAGAAGGAATTGTTCTATCTCCAAACTTCACCTTCATCAAGCGTAGGTTTATTTCAA
GAATCTTTTTTCTTTGTATCCCGAATCATGTCTCTTTCTCGTAAGACTGAGGGCGGTAAA
TAAATAAATTCAAAAAAAAAGCAAATCGCACCATCTCTGTAATAGGTAAATGCCTCTTT
TTCTCCTGAGGTTGTCGGAATTATTCGTAATAAGATATTGGCTACAATTGAAGAGGTCTT
ATCAATAAAATTTCCATTTATCCGAGATCTAGGCATAGTTAACAATCCATTCTAGAATTC
TTCTCATTACCCCTCAGGGGAAAATGATTCCACAAACAAAGGAATTGTACAGTACGAAAT
CACATAAAAACAGACTCATTCTAAAAAAAAAAATGTGGACCTTCCACTCAAATTATCCCT
TTTTGAGAGGTATAAATAGGAAATATTTGAATCGGATTGGATTTCATTGAAATTGAGTAG
TATACCAATGAATGGAACTCTTTTATTTTATCGAAGTTAAGAAATCCAGGAATTTTTACT
ACCGATTCTTATAATTTAATTCGAGAAATTTGGATTTGATTATGATCCAAAGAGGAAAAA
GAATCAAATAATCATTCCATGATGAAAATAGAATAACCATCCATTTTGTGTGCATAGTGT
GGATACACCATCCAATCGAAAGATAAAAATCTATAGAACGATTCATGAATTTGTAATAGA
TCTATGGAGTAGCTCATGAGAGGAGTTGTTGTTGAGAAATCTGAAACTGGAAGGGGGGAA
TTTTGTAATTCCTATGGAATCGTAGTTTAAATATAAACATAGTCTAAAATAGGGTCAGTT
GACTCGTTCCAATTCATTGGCTTAATCCGAAATATTAGAATAAGATAGGATCGTCGTATT
GACAAACAAGACATTTTTGTTTTTAACAAGAAAAAAGTGTGTTTTTTTTATCCCTCGAGC
CTCGAAGGAAAATCGTTCTTTAGCGAAAAGTTTTCTATTTCTAATAGATTGGTCGTACCT
GTATTGCAATAATATGAATGACTCGCTATTTACTCGGTTTCTGGGGCATAATAATAAGAT
TATGTAGGAGAGATGGCCGAGTGGTTCAAGGCGTAGCATTGGAACTGCTATGTAGACTTT
TGTTTACCGAGGGTTCGAATCCCTCTCTTTCCGTACCTTCATCTAATTCACCAACCGACC
ACAATGTATCAAATCAAATAACAATTGATACCATTATTCCAACAGTAAGACCCTTATTTG
ATAGAGATTCTCTATTCCTAATTACTGCAGTACGGAAAATACCGGAATAGAGTGGAAAGG
AATGAAAATCTCACTGCTGATCCATTTGTGATACGTGAATGGGAGAAAAATCCGGATCAA
ACCCCTTCTTCGGTGAAAAAAAAAAAGAGGGGGGGGGCAAAATGGTCCGAAGCTTTGTT
ATTTTAGTTAGGTTCAAGTCTGACGGGAATAATATTCTACGACTAGAAACTCATTGATTT
TCAAACCGATCCATTTAATATCTATTATTTGATTTACTAATCCTTTATATTGGGATGAGT
CAAAAGTCAAATGTTTTGCCAAATCCTCGCGGGGCGATGAATCAAGATAATTTTGAATCA
GAGCTCTGGATCTTTGTTCATCCCTTGCAGTAATAATATCTCGGGGTTTGCAGCGATAAC
TTGGGATATCTACTACACGACCATTAACTAAAATATGTCTATGGTTAACTAATTGCCTGG
CTCCAGGAATGGTCGAAGCCATACCTAATCGAAAAAGGATGTTATCCAAACGCATCTCAA
GTAGTTGTAGTAAAACCTGACCTGTTGACCCTTTGGCTTTTCCGGCAATACGAACATATC
TAAGCAATTGTCGCTCTGTCAGACCATAATGAAAACGCAATTTTTGTTTTTCTTCTAGAC
GAATACGATATTGAGATCTTTTCCCGAAACGTGATTGGTTTCTAAGATCACTTCCGGATC
TAGGTCTTTTACTAGTTAGTCCCGGTAAAGCCCCCAGACAGCGTATTTTTTTGAAACGAG
GCCCTCGGTAACGAGACATAAAGACTCCTTGTTAAAATTGTATTTTACAGAATAAACTTA
AATTAAGACTGAACTAAACGATAAACGAAACTAAATCTATTGAAGTACTACAAAAGAAGA
CTACAAAAGAAGAATGAGATGAATTGTATCAATATCCGGATTATTTTGTATATATAGGAA
GTGAAGGACCCCTTTCTTGATTTGTTCTGTAGTGTAGAGATTTAACTGCTCCAATCAAAT
AAGTTTTTTATTCATAGTTGGAAGTTGCTACGACATAATAGATCGGTGACCCGACATTTT
TAAAAGAAAAAAAGAGAGGAGTCTTTTCAATATTCCTTGAGATCAAGGAATATTGAAAAG
CCGGCTATCGGAATCGAACCGATGACCATCGCATTACAAATGCGATGCTCTAACCTCTGA
GCTAAGCGGGCTCACATAACAGAAATAAGTGCAATAGAACTAACTAACTATATCTATATA
GAATGTTTTTTTTAATTCTTAATTTATATATTATACTTAATTTATAGCAGTTAGTTATAG
CAGATTAGATTAATCATATTAGAGCAGATCGGTACTAAGGAAAGGATAAGATAAGGATGC
AATCCAGATCATAATGAGACATTTCGCCGGTTTCATTCAGAAAGGGGGGAGGTAGAACGA
AAAAAAAAAATGAATATCGACCGTTCCAGTATTAAAAATCGCGCGGGAAAAATGAGAGGG
GGGGAGGGTATGTATATGTGGGATATCTCTATCCATATTGAATTGCAGATACATCAATGA
TAGAATCATTTCTGATGGGACCAAATACGGGTCTTCCGATAGAGAATATGGACAAGAAAT
CAAAATAAAATAAATAAAATAATAAAATAGGAGTAGACTTTTTTTCGATATTAGGAATCA
GTATCTAATGAATTCAACGGTTCCGACATAAATAAATGAAAGAGGGGGATGGGATCACAA
TGAGATCTCGGTCTCATAAGGGGATATGGCGAAATTGGTAGACGCTACGGACTTGGTTGG
ATTGAGCCTTGGTATGGAAACCTACTAAGTGATAACTTCCAAATTCAGAGAAACCCTGGA
ATTAAAAATGGGCAATCCTGAGCCAAATCCTGTTTTCAGAAAACAAGGGTTCAGAAAGCG
AGAACCAAAAAAAGGATAGGTGCAGAGACTCAAAGGAAGCTGTTCTAACGAATGGAGTTG
ATTAACATTGGTATAGGAATCCTTCTATCGAAATTCCAGAAAGGATGACCCTATCCTATA
TACGTACTGAAATATCAAACAATTAATCACGATCCGATTCCGTATTTTTTTTATATGAAA
AATGGAAGAATTCTTGTGAATCGATTCCAAATTGAAGGAAGAATCGAATATTCAGTGATC
AAATCATTCACTCCTCGGATAGATCTTTTGAAGAACTGATTAATCGGACGAGAATAAAGA
TAGAGTCCATTCTACATGTCAATACCGACAACAATGAAATTTATAGTAAGGGGAAAATCC

GTCGACTTTAGAAATCGTGAGGGTTCAAGTCCCTCTATCCCCAATAAAAAGAAAAGAGCC
CGTTTTACTACCTAACCTCTTTATTTCGTCATCGGTTCCAAATTAGTTATGTTTCTTATT
CACCCTACTCTTTCACAAACGGATCCGGACAGAAACCTTTCTCTCTTATCACAAGTCTAT
AGATACGATATACTTACAAATGAACATATATAGGCAAGGAATTTCCATTATTAAATAATT
CACAGTCCATATCATTACTCTTACACTGACAAAGTCTTCTTTTTGAAGATCCAAGAAACT
CCAAGGCCTAGGTAAGATTTTGTAAGACTTTTTGGGTTTCTTTAATTGACATAGACCCCA
GTCCTCTAATAGGGCGATGCATCCGGAATGGTCGGGATAGCTCAGCTGGTAGAGCAGAGG
ACTGAAAATCCTCGTGTCACCAGTTCAAATCTGGTTCCTGACACGCGGTTAATGTATCGA
ATGGATACTCATCCAAATGAATGGGTAAAGGAAAGAAGTAGATTTTGTTCCTTTTTTTTA
TACTGTACCCCCTCTCGCTCAAAAAGAATGTTAATACTTCATACATATCCAAAGTTAGGT
GGCTGAAACCAAAAAGTCTAGCCTAGGGGAGTTGAAGGATAGGAATAGACAGGATTCATT
TCAGATACAGTACAAAGAAAATACGATCCCTTTTCATTTCTGAATTTCATATTTTCTTGC
GTATTCTATTTCCTCACTCCCTCTTACGCGACTTCCAGGAGCCCATCCAAGTGATATGCG
CGGTACAAAGTTCATGGTACAGAACTCTTTTGATTCATACTATTGGCTTTACTCATCCGA
AATAGATATATTTAAAATTGGGGAATATCAACGAAGCCTATTTATTAGCTCATCCATAAT
ACGAATTAGAGCCCAGTTACTCTGTTTCATCTAGAACGTAAAAAGATTCCTTGAATATCT
GGAGTCGTAGAAGTGAAGATTAGTTTCTTATCATTCAATGAGCATCTTGTATTTCATAGA
AATTGGGGGCAATATAATCCTTACGTAGGGGCCATCCCACCCAACTTTCGGGCATCAAGA
TACGTTTCAGGCGTGGATGATTTTCATAAGAGATCCCCAACATATCATAAGATTCCCGTT
CTTGAAAATCAGCACTTTTCCAAATCCAGAAAACAGACGGGATTCTAGGATTCCTCCTTG
GAACAAATACTTTTATGCACACCTCTTCGGGTTGATCCACCCCATACTGTATTCTCGTAA
GATGATACACACTAGCTAAAAATCCGCCAGGTGCTACATCATAGGCACACTGGAAACGTA
GATAATTGTAACCATATACATATGAAATAACAGCAATGGAGTACCAATCCTCGGGCTTTA
TTTGTAAAGTCTCTCTTCCTTGGTAATCGAAGCCCAAAGATCTATGAACTAGCTCATGCT
TGACTAGCCAAGCAGATAAACGACCCTGCATCTTCTTGATCTCTCCCACATTTGTATGAA
TATTTTACATTTACGATGAATTTTATGAAAATTGACTCGCCGTTTGTTATTCCGCACAAA
AACACCCTGCCTAATTCATTAATTTGGGGGAAGATACTGAACTTTTGTATTTGAAAAATG
TTTCAGAAGGTATCTCTGAAGTAGATGGAGATTGGTAGAGTAATCCTTGATCGTAATTTC
CAGTATGAGTACTGTGTCCAACATGAAACTTGTGATTGGTAGTAAAATATCGATTTTCCT
GTTGAGACCCAATTCTATCTTCATAGATTTCTCGAGAGACTTTCTTACGAAGTTTCGTTA
TAGCATCTATAACTGCCTCTGGTTTAGGGGGGCAGCCCGGCAAATAGACATCCACAGGAA
TTAGCTTATCGACTCCCCGAACAGTACTATAAGAATCGGTACTGAACATCCCTCCTGTAA
TAGTACAGGCTCCCATAGCAATGACATATTTTGGTTCGGGCATTTGTTCATATAATCTCA
CTAACGAAGGAGCCATTTTCATTGTTACTGTGCCGGCTGTTAAAATTAGGTCCGCTTGCC
TAGGACTTGATCTTGGTACCAGTCCATAACGATCAAAATCGAATCGCGAGCCTATTAATG
AAGCAAACTCAATGAAGCAACAACTGGTACCATAAAGAAGCGGCCATAAACTAGAGAGTC
TTGACCAATTCGAAAGATCATTCGATGTAGTTGAAATAACTGAATTTTGGGCGGTTCGGT
CAAGTAACGGAAACTCAATAGAATTCATAACTGTTTCAATGTAATCTTTTCCTTCTTTTT
GATTTTGATTGTCTGAATATTCAGGAGCTAAGACCATTCCAATGCTCCTTTTCGCCATGC
ATAAACTGAACCAACAATTGGGATAAGCACGAAAATTAAAGCTTCTATAAACACAGATAC
ACCCAATACATCGAAACTCATTGCCCATGGATAAAGAAAGACTGTTTCAACATCAAAAAC
AACAAAAACTAGAGCAAACATGTAATAGCGGATTCGGAATTGTAACCAAGCATCCCCCAT
CGGTTCTATGCCCGATTCATAACTAGAGAGCTTCTCTGGTCCTTCACTAATCGGGGCCAA
AACTCCGGAAATTAGAAATGCCAAAATAGGAATAACACTTGATATTATTAGAAATGCCCA
GAAAATATCATATTCGTGAAGCAGAAACATAGAAGCACTCCTATTAATGTGGAATATACC
GAATTAGTTGATTCAAATTGGAATTCTCAATTCATCCATAACTGCATTAGTCGAAACAAC
AATTTTGATCAAACCACATAGTTTCGTTTGTTTACTTGTTGTGGGTCATGTATCGTCTCA
AGATTCATCCAACGGAATCCCACTTACACTTACTTCGATTCTATTTAGATATGGTGTAGA
CATATAATGCTATTATACAAATCAAACTCTCTCCTACCTTGCCTCGGGTTTTCTATCAAA
CAAAAAAAGGAATTAAGGAATTTTTTTTAAAGAATATTTTAAATAATATGAATTGAAATT
GAAATAATATTCAAACAATATTATTCAAATAAGATTAAATGAAATATTAAACATAAATAA
TTTCAATATTCTATTAATATAATAGAGCCAAAGAGGAGGTCTGGCCCATTTTTTCTCTCT
CTCTCTTTTTTTTTTTTTTCTTAGTGATTTAGAATATAGTCAGTAGTCAGATGTAATAGA
ATTTCTAGGAATTTCCATCTCGGGATTTATGGTATATTCTACGTGGCTGTGTTGGTGTAT
TCTTTTCATTAAGATCCGGATAGAGGATTATTGTTTCTATTGATTTGATACGGATACGAA
AACGGAATGCATCGACCGATTCGATTCTCTCTCCCTGTCCCAGATTTATACTTAATTGAT
TTGATTCAATCCATTGAATTGTGAGGAACCCTTACATATAAAAACTCATGGGTTCCTATA
CCCAAATTAGGAAACTTTGGACCTGTACTACCCCGGCCCCGGCTTTACCCCCGAGTTAGA
AGTCTTGAAAGAATCATTTCAGACCCATTTCTAGGACTAAAGATCGTGATTTGGAATGAC
TCGAAATACTTATTTATTTAATGTAATAATAACACCTAGCAGGACCAACCAACGAGTTAG
GTTTCGTGACAACAAAAAACGTTTCTTTTGAAGCAAACCTACAAAATGGGGCATAGTTTA
ATGGTAGAGTCGGCTGAATCGTAAATGATTTACAGAAGATACTTCGAATGGAATCATGCG
TTGTCGAACGATTCGATAGACAAAATCTCCCCATCCCAAAACCAACTCATAGAACATAGA
AATAGAAGAGGGTGCGTTGATACATTTAGGACCAATTCATTGTCTCTAAAACAATGAATT
GGGATTGTTGTTCTGCCAAAAGGCGATACGTCGGGGGATTCCTAACTCATCCAAGTTCAA
ATGGACCCTAATCTTTTTAGATAAAGTCTGCATTGGTAGAATTGGAATGATGAACAAATT

GGATTGGGTAGATTGGAATAAAAAAAAGAAGTTATTAAGTATTGTACAGAAAAATGACTA
CTTGCTTTGCTAAGCCGGTTATACGAAGAAAAGCCTATTGTACAATGAAACTTACCAAAG
AGCTTCGTTTTTGAAACTCTGGCTTTTCTACAAATACAAGAACAAGAATAGGTTCTAGAT
AATGTGACTTACTATTAGATTGAATTTGGATTTGATTTGGTTGGGTCAGGTTGGAGTTTT
TCTTGAGCCAGGCTCATGTTATGATTTTGACTTCATAAATTGGCTTGGGTATACCAAAGC
AAAGGTGTATCACTAAATCTTGGATCATGGACAAATAAAAGAGGAAAAAGGCCGTATGTC
ATTCACAGACGAAGATTAATGAAGAAGAATGGGTTTGTTTATCCGAGATTTGAAAATACC
GATCCGATTGGATCCATTGGAATAAATTATTGTTTTCAAGCCCCGAGGGATCTCCGTGAT
CCTGTGGGAATGATTCCATTTCTATGGAACAATCAACCGGCCGGTCACACGCACTAATTA
GGAAATGAATACAAAAATGTATAGGGCTATACGGACTCGAACCGTAGACCTTCTCGGTAA
AACAGATCAAACTTATTATTATCGAAATGATTCGAACTGTTTCAAAGACCCAACATGCG
TTTTTTTTTTGCATTGGGCTCTTTCATTAACTGATAAAAAGATCGGCTAGTCCACCATAT
TTTTTCTTGACAGGAAGATAACGAGATGGCTCCATGCGCTCGGATTCATTATTTGAATTC
TGATCCGGGAGCAATACCAAAGTGTTTCAAAGAAGGGTTACCCTGACGTAGGTCTGCCTC
CGGCCTAGATCAACCTAAGTTAAATGGAGTCTCTATCAATCCGCCCCAAGAGTCAAATAT
GATACTTAATACACCTTAAAGTTCATAGGACGAAAAGAGGTTATTTTGAGGTCCTTATCC
TCATTATGCCTAGCATTGAAGGGACTGGGTATTCACCTTATCAATGATCAAACCAATGAT
GGGTTCTATTTGGTACCTGAATTGGCACCTGAATCGGACCGAACAAAATATTTGTCAGGC
TATTGTTCTCTTGTTCCCTCGAATCCATGGAGTAAGACATCGATTTCTCAATAAGATCAA
TTCTGTTGATTGCATGATGGACTCCTCTGAAAAAGCATTGGCGCGCGTGTAAACGAGGTG
CTCTACCTAACTGAGCTATAGCCCTTGTCATAGACATATTAACATCTAGATAATTTCTTG
TCAAGATGGATATTCCATAATCCCACATGATAACTCTCCGATCCGTTTCCTGCCAAGGAT
TGGTATTGCTGAGAAGTAATATTCCGTCTATAATCCCCGATGTGATGGGTCCCATTTTTT
CTTTCTCTTTGTGATGATAAATGACCTACTTAACCCAGTGGTTAGAGTATTGCTTTCATA
CGGCGGGAGTCATTGGTTCAAATCCAATAGTAGGTAGAACTTATTAGATACCGGAGTCGA
TGGTATCTAATAAGTTTTTCTACCCACCTTCTTCTCTTTTTTTTTTTATGGATTTTGTACC
CTTTCCCTATTATACCCCCACTACTCATATTTGTATTTGTTTTTTTTTTGTTCGTTACAT
CAGATTACAATTGATTGTATCCAATTGGCGGAATCCAAATATGGTGTATAAACAGAACTT
CTTTTGATTATTCTGATACATTGACTAGTACGAAATAACATTGATAGCCTCTACTCGTGT
CCTAGCTCGTCTAAGAGCTAGATTCGCCTCAATTGCTTGTCTCTTGCCTTCAGCTCTACT
CAAGTTAGCTTCAGCTATTTCAAGAGTTCGCTGAGCTTCTTGTGGATCAATGTCACTACC
CTTCTCTGCATCATTTACTAAAATGGTGATCTCATTATTGCCTATTCTAGCGAAACCGCC
CATCACAGCCATCGTTAACCATTGGTCGTTGAGGCGTATTCTCAAAATACCTATATCTAC
GGCTGTGGCAATAGGGGCGTGATTTGGTAATACGCCAATTTGGCCACTATTAGTAGATAA
AATGATTTCTTTCACTTCCGAATCCCAAATAATTCGATTCGGAGTCAGTACACAAAGATT
TAAGGTCATTTCTTCAATTTGCTCTCCACTTCTAAGTTCATAGCCTTCGCAGTAGCTTCA
TCAATGTTACCTACCAAATAAAAGGCCTGCTCGGGAAGACCATCTAATTCTCCGGAAAGG
ATCAGTTGAAACCCCCTAATTGTTTCTGTAAGACCAACATATTTCCCTGGAGAACCAGTA
AATACTTCTGCTACGAAGAAGGGTTGTGATAAGAAACGTTCAATTTTTCGTGCTCTTGCT
ACGGTTAAACGATCCTCTTCAGATAATTCGTCCAACCCAAGGATAGCTATAATGTCCTGA
AGTTCTTTGTAACGTTGTGAAGTTTGCTTAACTCTTTGCGCAGTTTCATAATGTTCCTCA
CCAACGATCCTAGGTTGTAGCATAGTTGACGTTGAATCTAACGGATCTACTGCTGGATAG
ATACCTTTGGCAGCTAATCCTCTTGATAGTACGGTAGTAGCATCTAAGTGCGCAAATGTC
GTAGCAGGAGCAGGGTCGGTCAAATCGTCCGCAGGTACATAAACTGCTTGAATGGAAGTT
ATAGACCCCTCTTTGGTAGAAGTAATTCTTTCTTGCAAAGAACCCATTTCTGTACTAAGG
GTAGGTTGATAACCCACAGCGGAAGGCATTCTACCTAATAAGGCGGATACTTCTGATCCT
GCTTGGACGAAACGGAAAATATTGTCGATAAATAGAAGTACGTCTTGTTCATTAACATCC
CGAAAATATTCCGCCATGGTTAGGGCAGTCAAACCAACTCTCATACGAGCTCCCGGCGGT
TCATTCATCTGTCCATGGACTAGAGCTACTTTGGATTCTGCAATATTTTGTTCATTAATC
ACTCCGGATTCTTTCATTTCCATGTAAAGATCATTTCCTTCACGAGTACGTTCGCCTACT
CCGCCAAATACAGATACACCTCCATGAGCTTTGGCAATGTTGTTGATCAATTCCATGATG
AGTACTGTTTTACCCACCCCAGCTCCCCCGAATAGTCCGATTTTTCCTCCACGACGATAA
GGGGCTAAAAGATCTACCACTTTAATCCCTGTTTCAAAGATTGATAATTTGGTATCTAAC
TGGATAAAAGCAGGCGCAGATCTATGAATAGGAGATGTTGTGCGAGTATCTACAGGACCT
AAATTATCAACAGGCTCTCCAAGAACGTTGAAAATTCGTCCTAGAGTAGCTCCACCCACT
GGAACGCTTAGAGGAGCTCCCGTGTCAATCACCTCCATTCCTCTCATCAGACCATCTGTA
GCACTCATAGCTACAGCTCTAACTCGATTATTTCCTAATAATTGCTGGACCTCACAAGTC
ACATTAATTTGCTGACCGACAGTATCTCGACCCTTAACTACCAAAGCGTTGTAAATATTA
GGCATCTTGCCGGGGGGAAAAGCTACATCCAGTACCGGACCAATGATTTGAGCAATACGC
CCCAGGTTTTTTTCTTCAAGTGTGGAAACCCCAGGCCCAGAATTAGTAGGATTGATTCTC
ATAATAATGAAAGTGAAATATGTCAAAATTTTTTGCGAATATTACCGAATCGAAAATAAA
TGTCCGATAGCAAGTTGATCGGTTAATTCAATAAATAAGAAATGGGAGTTAGCGCTTGAT
TTCGTTGGTACCATTCAACTGAATCCAACTCAATCGTTTACTCATTCACTAAATGAATTT
TCAAGTTCAACCAACCCTTTTTCAAAATATCTATCAAATCAAGTAGATGAATAAGAATCA
TGGGGAAGTCTTTCATTTTTCTATCATTATAGACAATCCCATCCATATTATCTATGGAAC
TCGAACCTGAACTTTATTTATGATTCAGAATTTCTATCTTATTGGCCGTTGTTCCTTATT

TCAGCATATTAGTTTCCGCCTATTCTTGTTTTTATTTTTTATACCCTTTCATGGATGAAT
TCTGCCTATTTTCACATCTAGGATTTACATATACAACATATATCACTGTCAAGGGTGAAT
TTCTTATTATTTAGATTCAAAAAAAAAGAAGGAGATCCAAACTTGCAAAACAAGGATTGG
GTTGCGCCATACATATGAAAGGGTATACAATAATGATGTATTTGGATGTATTTGGCAAAT
CAAATACCATGATAACGAACCATTCTAATTAGTTGATAATATTAGTTGAGAATTTTGTGA
AAGATTCCTGTGAAAGGTTTCATTCATTACTAATCCATGTCGAGTAGACCTTGTTGTTGT
GAGAATTCTTAATTCATGAGTTGTAGGGAGGGACTTATGTCACCAAAAACAGAGACTAAA
GCAAGTGTTGGATTCAAAGCTGGTGTTAAAGATTACAAATTGACTTATTATACTCCTGAA
TATGAAACCAAAGATACTGATATTTTGGCAGCATTTCGAGTAACTCCTCAACCCGGAGTT
CCACCTGAGGAAGCAGGGGCTGCGGTAGCTGCCGAATCTTCTACTGGTACATGGACAACT
GTGTGGACCGATGGACTTACCAGCCTTGATCGTTACAAAGGACGATGCTACCACATCGAG
CCCGTTCCTGGGGAGGAAACTCAATTTATTGCCTATGTAGCTTACCCTTTAGACCTTTTT
GAAGAAGGTTCTGTTACGAACATGTTTACTTCTATTGTGGGTAATGTATTTGGGTTCAAA
GCTCTACGAGCTCTACGTCTGGAGGATCTGCGAATTCCTCCTGCTTATTCCAAAACTTTC
CAAGGCCCGCCCCATGGCATCCAAGTTGAGAGAGATAAATTGAACAAGTATGGTCGTCCC
CTATTGGGATGTACTATTAAACCAAAATTGGGGTTATCCGCCAAGAACTACGGTAGAGCG
GTTTATGAATGTCTCCGTGGTGGACTTGATTTTACCAAGGATGATGAGAACGTGAACTCC
CAACCATTTATGCGTTGGAGAGACCGTTTCGTATTTTGTGCCGAAGCAATTTATAAAGCG
CAGGCCGAAACAGGTGAAATCAAAGGACATTACTTGAATGCTACTGCAGGTACATGCGAA
GAAATGATCAAAAGGGCCGTATTTGCCAGAGAATTGGGAGTTCCTATCGTAATGCATGAC
TATTTAACGGGGGGATTCACTGCAAATACTACCTTGGCTCATTATTGCCGGGACAACGGC
CTACTTCTTCACATCCATCGCGCAATGCATGCAGTTATTGATAGACAGAAGAATCATGGT
ATGCACTTTCGCGTACTGGCTAAAGCGTTACGTATGTCTGGTGGAGATCATGTTCACGCT
GGTACCGTAGTAGGTAAACTAGAAGGGGAACGGGACATCACTTTGGGTTTTGTTGATTTA
CTACGTGATGATTTTATTGAAAAAGACCGAAGTCGCGGTATTTATTTCACTCAAGATTGG
GTCTCTATGCCAGGTGTTCTGCCCGTGGCTTCAGGGGGTATTCACGTTTGGCATATGCCT
GCCCTGACCGAGATCTTTGGGGATGATTCCGTACTACAGTTCGGTGGAGGAACTTTAGGA
CACCCTTGGGGAAACGCACCTGGTGCAGTAGCTAATCGGGTGGCTGTAGAAGCGTGTGTA
CAAGCTCGTAATGAGGGACGTGATCTTGCTCGTGAAGGTAATGAAATTATCCGTGAAGCT
GCCAAATGGAGCCCTGAGCTAGCTGCCGCTTGTGAGGTATGGAAGGAGATCAAATTCGAA
TTCGCCGCAATGGATACCTTGTAATCCAGTAATTCCCGTTCGTTCCCCTAATTGTAATTA
AACTCGGCCCAATCTTTTACTAAAAGGATTGAGCCGAATTAAAGAATGAGGATCCTATGT
ATATGGATAGATATAGATCTTGTATCTATCAATATGTGCCTACTTTACCTAGATATACAA
GATCTAAATACAAGATAAGATCTAAGACTAAACAACTCAATGCTTCTATTGTTGGATCCA
TAATTAATCCTATGGATCCTTAGGATTGGTGGATCCTTTTCTATCCCGTTGTTTCGGACC
ATAGATCGAGCCAAGGGTCACAACTTCTTCTACTCATCCTGTATATTGTCCTTTTCATTC
CGTGTTGCATTAGAAACTTATTATTATACGAGATTATACGAAAATGAATCCTTCCTAGGA
GGGAACAAATATTTCTCTTTTCGATGAGAGTTTGTACACAACATGGGAGAAACCTATCTT
CTATTTATAATAATTGAAGAAAAGGTTCCATCATATCATATATAGTGAATTGATACTCCC
GATTCCCACAAAATCATTTCTTTCGTTCAATAGTTACTCGTTATTAGTTAATAATCCTAG
TGATTGGATCTATATGCGTATTCCGATAGGAAATGAAATAGTAAAATGATTTTTCGTCGA
ATGACTATTCATTTATTGTATTTTCAAATAGGGGGCAGGAAGGATCTATGGGAAAATGGT
GGTTCAATTCAATGTTGTCTAACGAGGAGTTAGAACACAGGTGTGGGCTAGGTAAATCAA
TGGACAGTCTTGGTCGTCCTGTTGGAAATACCAGTGGAAGTGAAGATCCCATTCTAAATG
ATACGAATAAAAACAATCATAATCATGGTTGGCGCGAAAGTAATAGTTGCAGTAATGTTG
ATCATTTTTTCGGTGTCAGAGACATTTGGAGTTTCATCTCTGATGACACTTTTTTAGTTA
GGGATAGTAATGGTAACAGTTATTCCGTATATTTTGATATTGAAAATCGGGTTTTTGAGA
TTGACAATGATAGTTCTTTTCTGAGTGAACTAGAAACTGCTTTTTCTAGTTATCTGAATA
GCGGGTCTAAGAGTGACAATCGCTACTATGATCATTATATGTATGATACTACGTATAGTT
GGAATAATCACATTAATAGTTGCATTGATAGTTATCTTCGTTCTGAAATCAGTATTAATA
AGTACATTTCGAGTGGTAGCGACAATCCCATTTACAGTTATATTTATAGTTACATTTGTA
GTGGTGAAAGTGTAAGTGATAGTGACAGGGGGAGTTCTAGTATAAGAACTGGCGGTAATG
GCAGTGATTTCAATATAAGAGGAAGATCTAATGATTTCGATGGAAATAAAAAATACAGAC
ATTTATGGGTTCAATGCGAAAATTGTTATGGATTAAATTATAAGAAATTTTTTAGGTCAA
AAATGAATATTTGTGAACAATGTGGATATCATTTGAAAATGGGTAGTTCAGATAGAATCG
AACTTTCGGTTGATTCGGGCACTTGGGATCCTATGGACGAAGACATGGTCTCTATTGACC
CCATTGAATTTCACTCGGAAGAGGAACCTTATAGAGATCGTATCAATTCGTATCAAAGAA
AGACAGGTTTAACTGAGGCTGTTCAAACAGGCATAGGTCAACTAAATGGGATTCCCATAG
CCATTGGGGTTATGGATTTTCAGTTCATGGGGGGTAGTATGGGATCCGTAGTAGGCGAGA
AAATCACCCGGTTGATCGAATATGCTGCTAATAGATCTCTACCTGTTATTATGGTGTGTG
CTTCTGGAGGAGCACGCATGCAAGAAGGAAGTTTGAGTTTGATGCAAATGGCTAAAATAT
CTTCCGCTTTATATGATTATCAATTCAATAAAAAGTTATTCTATGTATCAATCCTTACAT
CTCCTACAACCGGCGGAGTAACGGCCAGTTTTGGTATGTTGGGAGATATTATTATTGCTG
AACCCAATGCCTACATTGCATTTGCGGGGAAAAGAGTAATTGAACAAACATTGAATAAGA
CAGTACCTGACGGTTCACAAGCAGCTGAGTATTTATTCCATAAGGGCTTATTTGATCCAA
TCGTACCACGTAATCCTTTAAAAGGTGTTCTGAGTGAATTATTTCAGCTACACGGTTTCT

TTCCCTTGAATCAAAATTCAAGTAGAGCGCTAGGCTCAGTTATTTGTAGCGAACTTTAGT
TCATCGGAATCAAAGTCAAAATAAGAAGAGTGGAGTTTTCTTTGGTAACATAACTTCTAT
AGGAAGTTTCGGATAATTACTTTTTTTGATGCAGATTTTTTATCCTACCCCTATTCATGA
TTAGTAATCAGGAACCCCCTATCAGGAGGAAAAGAGTGAATTCTTCCTTCCGCGGAATGG
AATTGGGAAAAAAAATCAAAAGAATTTCATGTTCCCTTCTTTCATATTAATATATATCGT
ATTAATATATATAGAATAATTCAAATCTATAAGGGAAGTGTTGCATATTTTTATATCTCC
CGAGGACCTACACTTTTGACTATGAATTCCTGTTGGATCGGATTCTAACAAATCCTTAGG
AAACTCGTAAGAAACTCTTTATTAGAAGATAAGGGCGGTAGAACAAGAATAATAAAGCGG
ATTATCATCCATCTATTTCTTGTAGAAAGGTGAATAGATACACTTATTTAGCTCTACATT
CCTTGCACTTATTATATACTTAATAATACTTATAATAGATATACTTATCATAAGATAAGA
TATCTTTATAACAGGTACAAATATTAAATCGAGGCACCCATTCTATGACAGATTTCAATT
TACCCTCTATTTTTGTGCCTTTAGTGGGCTTAGTGTTTCCAGCAATTGCAATGGCTTCTT
TATCTCTTCATGTTCAAAAAAACAAGATTGTTTAGACCTGATAGGACAAAATTTCATCAA
TTTATTTCAACACTTGGACTTGGATCATAATAGGGATATCCATTTAGTGGAATATGATAC
GACATGTGGCCCCCTCCGGGCACAAATAAAAAAGTGATTATACATGCGGATACATTATAT
ATGGATAAATGCATGTATATGGGGGGATAGCTGTTTTAAAATGGATCAGAGTGGATATCT
GAAATAGAAAGTTAACGTATCTATATTATGTAGATATACATAGTGGTGGTATTATACGAA
GGGGATGTTATTATTTTATATCTAACCAATTCGATGAATTACTCCTAATAGTTCGCGTCA
TAATAGTGCTAGTTGATGAGAGTTACTTCGGGAGCAAAATAAAAAAAGTAAAATCAAATT
CATTTGGCTTATTCTCTTCTCTCAATTCCAATAGGATGCAACTGAATCTAGTATGAACTA
TCGATCAGAACGTATATGGATAGAACTTATAACGGGGTCTCGAAAAACCAGTAATTTCTG
CTGGGCCTGTATCCTTTTTTTAGGTTCACTAGGATTCTTATTGGTTGGAACTTCCAGTTA
TCTTGGTAGGAATCTGATATCTTTATTCCCGTCTCAGCAAATCATTTTTTTTCCCCAAGG
AATCGTGATGTCTTTCTATGGGATCGCAGGTCTGTTCATTAGTTCCTATTTGTGGTGCAC
AATTTCGTGGAATGTAGGTAGCGGTTATGATCGATTCGATAGAAAAGAAGGAATAGTGTG
TATTTTTCGTTGGGGATTTCCTGGAATAAATCGTCGCATCTTCCTTCGATTCCTTATGAG
AGAGATTCAATCGATCAGAATGGAAGTTAAAGAGGGTCTTTATCCTCGACGTGTCCTTTA
TATGGAAATCAGAGGCCAGGGCGCCATTCCCTTGACCCGTACTGACGAAAATTTGACTCC
ACGAGAAATTGAACAAAAAGCTGCTGAATTGGCCTATTTCTTGCGCGTGCCAATTGAAGT
ATTTTGAAATGAAGGGAATGAATGCTTTCTCAGCATGAGGGAAGGGACCCAGGAACCCCC
TTTTAAATATAACTGAAGCTTCTTCGGAACGTTCATTCGAGCAAAACATGTTAGATTCTA
TTTCCCCCGTTCCGTTGGTAATCCTTCTGTGGCCCATAGAATAAAGCAGGCGGACGTATA
CGGAACAACCATAATAAGAAGCAATTTTGATCGACCAAAACTCCTTTTTCTGCATATAGA
ACTCAATTCTACTAGTAACAAGTCTAATAAGTATGTATTCATCACACATATCAGAGCATT
TCGGAATACATAATTTATCTTTTTAGGACCAATACTTTGGATTAATACATTAGATACAGA
TGTATCATATCCCGTTAATTATCTTTCTTTTGTCAATCGATGTTTCTTTTTTGATCCTTT
CTTTAGCTCCTGGATAACCAAACGTTTAGATCTCTCATAACTATCCAATTTCTCTCTTGT
TTTGTCACCTATTTCGGATTTCATCATTAATATTTTTCAGAAATATCCCCTCAATTATTC
CCGGGTCGTGGGTAGCCAGTGAAAATTTCGAAAAAATAATTGAGGGGAGTTCTTTCGTCT
CGAAATCAAAATAATATTCATTATTTCAAGCGGTTCTTTTGGGCATTCATCGAAAGAACA
AATGAAGATAGAATTGGTTCGAATTTGACCAACTGAGATATCTGGGAAAAGTATTTGATT
ATTTCTTCATTCGAAACGGGCCCTTATTCTATTTCTATATTCTTTCTAGATCCA
AGGACTAAACAATTCAAAAAAAAAAGGAATAGATCCATAGGTTCCATACCTTGTTATAGA
ACTCATGCTTCATAGAAATATCGGATCAGATAGAGTCGGCGAATGAAGCGGGTTCATTAA
CAATTCACAGATGAAAAGTGTCAAAAAAGAAAGCATTGACTCCCCTCCCGTATCTTGCAT
CTATAGTCTTTTTGCCCTGGTGGATCTCTCTCTCATTTAATAAAAGTCTGGAACCTTGGG
TTACTAATTGGTGGAATACCGGACAATCCGAAACTTTTTTGAATGATATTCAAGAAAAGA
ACGTTCTAGAAAGATTCGTAGAATTAGAACAACTATTCCTGTTGGATGAAATGATAAAAG
AGTACCCGGAGACACAGATACAAAAGCTTCGTATAGGAATCCACAAAGAGACGATGCAAT
TGGTCAAAATGCACAACGAAGATCATATCCATATCATTTTGGATTTCTCGACAAATATAA
TCTGTTTTGCTATTCTAAGTGGTTATTCTATTCTGGGTAATGAAGAACTTGTCATTCTGA
ATTCTTGGGTTCAGGAATTCCTCTATAACTTAAGCGACACAATAAAGGCTTTTTCTATTC
TTTTATTAACTGATTTATGTATCGGATTCCACTCACCCCGGGGGTGGGAACTAATGATTG
GTTCGGTCTACAAAGATTTTGGATTTGCTCATAACGATCAAATTATATCTGGTCTTGTTT
CCACTTTTCCAGTCATTCTAGATACAATTTTGAAATATTGGATCTTCCATTATTTAAATC
GTGTATCTCCTTCACTTGTAGTGATTTATCATTCAATGAATGAATGAAGAACTCATTTGA
TCTGCTGATATCAATCAAATCATGATGCTACTTCGTACATAAACAAACTGTTTTGAAGCT
TACTCACTCTTTATACTTCTACCCGCCCAGGGGGTTCCTACTATACTTCAGTACAATTAT
TCCAGTACAATGGCAGAATCATGGATAGGGAACTATGCTAGCTACCTACCTAATTTATTG
TAGAAATTTCCGGGATCAATTATTGGACCATGCAAAATAGAAATACCTTTTCCTGGGTAA
AGAAAGAGATGACTCGATTCATTTCCGTATTGATCATGATATATGTAATAACTCGGACAT
CTATTTCAAATGCATATCCTATTTTTGCGCAGCAGGGTTATGAAAATCCACGAGAAGCAA
CCGGTCGTATTGTATGTGCCAATTGCCATTTAGCTAATAAGCCCGTGGATATTGAGGTTC
CACAAGCTGTGCTTCCTGATACTGTATTTGAAGCAGTTGTTAGAATCCCTTATGATATGC
AAATGAAACAAGTTCTTGCTAATGGTAAAAAGGGGGCTTTGAATGTGGGGGCTGTCCTTA
TTTTACCCGAGGGATTCGAATTAGCTCCCCCCGATCGTATTTCTCCCGAGCTGAAAGAAA

AGATGGGCAATCTGTCTTTTCAGAGCTATCGCCCCACTAAAAGAAATATTCTTGTAGTGG
GTCCTGTTCCTGGTCAGAAATATAGTGAAATCGTCTTTCCCATTCTTTCTCCGGACCCTT
CTACTAAGAAAGACGTTCACTTCTTAAAATATCCCATATACGTAGGCGGGAACAGGGGAA
GGGGTCAGATTTATCCCGACGGGAGCAAAAGTAACAATACAGTCTATAATGCTACAGCAG
CAGGTATAGTAAGCAGAATAGTACGTAAAGAAAAAGGGGGATATGAAATAAGCATAGCCG
ATGCATCGGATGGACACCAAGTGGTTGATATTATACCTCCAGGACCAGAACTTCTTGTTT
CAGAGGGTGAATCCATCAAGCTTGATCAGCCATTAACGAGTAATCCCAATGTGGGTGGAT
TTGGTCAGGGAGATGCAGAAATAGTACTTCAAGATCCATTACGTGTCCAAGGTTTGTTGT
TCTTCTTGGCATCTGTTATCCTAGCACAAATCTTTTTGGTTCTCAAAAAGAAACAGTTTG
AGAAGGTTCAATTGTCCGAAATGAATTTCTAGATCCACGGATTCATCAAGTTGATAAAAA
GGGCCGAATTATTGTTGATCAATGCAATTATGTATGATCCAAAAAAATATGGAAAGCCCC
TTGTCTTGCTTTGTTTATCCTGCTTTTCTGCGAGATGCCGGGAATTGCTTGTATCCCATT
CCCAGTAATAGTATGTATATTGCGAAGAAGACTACTTGACCCCCCCCTTTTTTTTTT
CAATCCAAATTGGAGCGGTGTGACGCTTCTTATTGCCAGATTGTAAATGCCATGGAATGC
ATCAATAGTTTTTTCTATCTAATAGAATCGAATTCTAATAGACAATAGGCGGCATAACAT
TAAGTAGGAAGAGAATACGCGGCAAGGGATAAATGAAAGAATGATTCTGGGAGGGATTAC
TTGTCTTCCTAATTTTCGACACAAGAAAAGGAATTTTCCGCCCTTTTCTTGTGTCGAAAT
AATAATGATTCTTGATCTTGTTCGTCAAAGATTACTGTTTTCTTTTCCAGGTCTATCGGA
ACCTCTTTCTTTAGATTCATAAGAAGTGGCGGACAAACAAAAAAGGGGGATGGCTTAGTA
AACAAATAGAACTTCTTCAACGAACTTATAAAATTTCAAGTAAAAAAAAATAAAATTTT
AAGATGAGATAATAAATAAGGATTTGCATATGTGCAAAAATCCAAGATTTTCCCCTTTCA
ACCGGAACATTAAGAGTCTTTTTTTACTTGATGAAATTTTGTTTTTATAATTTTTATAGA
ATAGAGTAGAGTAAGGTTCAATTAAATTAGTATAGAAATGGTTTGCAGGATGTCTCATCT
GTAGAAATCCTGTGTCATCCAAAAAATAAATTGATTCCTTCTTTCTTCTTGTTTCGGAAG
GGGCCCTCATACTATGGCGGAACAGATACTATGAATCAATCAAGGGATTCCATTTTTCAA
AAACATCATCAGAAACAAAGCATCCTTTTATCATTTCTATGAATCTAATATTATGATTAT
GTTGACTGGATGAATTTCCAACTTTTTTTATGTTATGGAATAGATCAAACAAAACCTTAC
CCGAAGAGTAAGAACTCAATAGGACCTTACCCCTCTTTGTCTGATTCGGGGGGTAAGGTC
CTATTGAGTTCTTACTTTTTCATGTCTACAATCCGGCTCATCCGATTACTATAGGGATGA
TCCCAATCCGGAATATGAGCCGTAAAAGAAAATACCTATTGAACCGATCACAGGAATACC
AGTTACAGTACCTATAAGCCAAAGAGGAATCCTTCCAGTAGTATCGGCCATTTACCCACT
TCCCTCCACATTTCATCAAGTGGTCGTGCTAGAGACATAAACAGTCATGGATAATTATGA
GGATGATATCCTTCCGAATGGGATAAGAGAATTCCTACTCTTTTTCTTTATTTTTTCTCA
ATTGAAGAAATAATTGGAAAATAAAACAGCAAGTACAAAAATGAGTAATAACCCCCAGTA
GAGACTGGTACGATTCAATTCAACATTTTGTTCGTTCGGGTTTGATTGTGTCGTAGCTCT
ATAATTCGGATTAGGTTTATCGTTGGATGAACTGCATTGCTGATATTGACCCCAAAAAAG
AAACAGTAGGTACAGCTAGTCCGTGAACAGCCAACCATCGCACTGTAAAAATTGGATAGG
TTCGATCTATGGTCATTGAGGCCTCCTAAAAGGATCTACTAAATTCATCGAGTTGTGCCA
AAGGATCAAAACGACCAGTTATTAATGGAATTCCTTGCCGGCTCTCTGTGAAATATTCGT
TTGGCCGAGGGCTTCCAAACACATCGTAAGCTAAACCTGTGCTGACGAATAACCAACCCG
CAATGAATAGGGAAGGTATAGTAATACTATGAATGACCCAGTATCGAATACTGGTAATAA
TATCAGCAAAAGAACGTTCTCCCGTGCTTCCAGACATGCTGAGCTCCACATATTCTTGTA
CAGTCAAACAGAGGATCGATTCCGTGAAAGATGGGATCAGTAAATGGAAAACTACTGATA
TTTCATCCTTGTGAGATCGTCAATAGTGTACCGAAGGTGTATTTAGAGTATACCGAATCA
GTATAGCTATCCTTCCTCTGACACAGCAATGCATTTTCAATCAGTATCGAAAAGAAATGG
AATTCCTTTCTTCCTTTCTTGTTCCTTGTCTATGCAAAACCGCGTGTCATTCAATAGAAA
ATTCTTAAATACCTGTAATATAGGGTTTCCTTTACTGGCTTCGGAATAGAAACTGAAGAT
CTTGGTAAAGTATGAGTCGACGGGTTCTAATAATTCATGATTATTATATTTCCACAATTC
AATTAGATGCAAAATTTAGAAACCCCTTTTCATGGTTGTCGAAAAGGTATTTTTTTTTC
TAATCCTTTCATTTAAAGTAATTGGTTGGTCGTACAGTAGTAGACAGTAGTAGGTAGTAG
TAGATGGAAAAAACAGAACAAACAGTAGTTGGAACAATTATCAATATTTGTGATGCAACC
ATTGCTGCATTAGACCCAAAGGTTCCTTCTTAACCCAGCTACAAGGATGGGACTGAACTT
TATGATATGGAAAGACAGAGTGCGAAACCTAAACTAAAAGGATAATAGCAATTGCTAGTT
TTAGAATGAAGTTGGGCTCGAAATAAAGGTTTTATTTCTTCGAGAAATCATGGGATACTT
TTCATTTTTCTTCTCGTTCGAAATATTATGTGCAATTAACCAACCTACTACTGAATACAA
TTAAGTTAAAAAGTCAAGTAAAAGCCGTTATCCGGCTGTTTGTTCCAAAATGGATTAGAT
AAATTGAAAAAGAAACGAAATGATCAAAAAAAATGGAATTTTCAAATCCAATTCTTTATAT
TCTGATAGTTACTCAAAGAGAATTTCATTTTTGAATTGAATGAAGTTACAAGACACAGTT
CTTATTATTAGTACTTTACTCACGGGTTGCTCACTGAATCTGTTGATTCGGAATCATGGG
ATCTGTAGATGTTACAGGCGACGAATCCATCTTTTTTTTCTACCCCTCTTACTCTCTCTT
TGTTAGTGCCGTCTATAATGGATGATGAATCAAGAGCTTTCAATTGGAACTGATTCTGTC
AATTGGTATTTTTTCTTGTCATTGTATCTCGCAAAAATGGAAACTTAGGTAAGTGCTTTA
GAACCCTATGTATGAAAAAGAAGATATCTCATTTAGCTCCTCCATGACTACTATAACTAG
TTATTTCGGTTTTCTACTGGCTGCTTCAACTATAACCCCAGCTCTATTGATTAGTCTGAG
CAAGATACGACTTATTTGAAATTAATTGAATGAACAATTCATAAAAATGAATATTTCTGT
GAGATTCCCGGTATTCTATAGTTCCTTCCCGCGTTAATTGCCAATTCTTGGTTATTGAGA

TTCATGGGCGATTCGGATTAATATTTAGGGACAGATATTACCTCTCTTTTTCTATTCTTT
CAAAGAAATTGAAATGATTGAAGTTTTTCTATTTGGAATTGTGTTAGGTCTAATTCCCAT
TACTTTGGCCGGATTATTCGTAACTGCATATTTACAATACAGACGCGGCGATCAGTTGGA
CCTTTAATTGAGTAACATCTCTTTTTTTTAATTGACCTCCTCCTTAATCTCCAGGAGGTC
AAATTCAGGTTGCAGTTCAAGTTAGTGAAGTTATTTTATTGTGATTCAACATTAAAAGAA
CAGAATCACGCTCTGTAGGATTTGAACCTACGACATCGGGTTTTGGAGACCCACGTTCTA
CCGAACTGAACTAAGAGCGCTTTATTATGATGGGAGATACGGATGTCAAGAAAAGGATTC
TTTTTGTACCCCCAATACATCTTGTATGTATAGTATCACAAAATGGTAGATTGTGTCCAA
TTTTAATCGATCTCAATTGACCCCTCGTTACTGTCCATAGGAGAAGTAATAGGTAGGGAT
GACAGGATTTGAACCCGTGACATTTTGTACCCAAAACAAACGCGCTACCAAGCTGCGCTA
CATCCCTTTCATGATTGTTGTACAGTGCCATTGTAGGGAATCCATGTTTTGTTTTCCACA
TCATAATTTCCTCTATCTAAATAGAATTTCTTTTGCCATTTCTTCTTTTTGGTTTTTTGG
TTTCATTCTCAATAAAGAATTATATACATACCTAACGTATAAACGTATAAAGGAATGAAT
ATTTATCAGTAGTGCTCAGGAAGGAGGGTTCATCTTTTTCTGTTTTAGGGACAGGTAGAT
TTCATCTACCGGATCGTTGTATATATCCATTTTGGTTAGAGATTCCCCGTACAAATGATC
TTTAACTACATATGCATCTGATCATATATGTATTACAATATACAATAAAGTCAATAAAGT
TAACTTTAAAGAAGGAGGATTTTCAATGCGAGATATAAAAACATATCTCTCCACGGCACC
TGTGCTAACTACTCTATGGTTCGGGTCTTTGGCGGGTCTATTGATAGAGATCAATCGTTT
ATTCCCAGATGCGTTGACATTCCCCTTTTTTTCATTCTAGTTATTGACATGGGAAGGGAT
CAAGAAGATTAGAGATACAATAAATTCTCTGTGACTAACCCCCCCCCTTTTTCAGTTCTT
TAGGATAGGAAAGAAAGAGTAAAGAATAAAAGTGGATTGAATCTCATCGAAACTCGGGTT
CGGGTTAATATAGGGAGAACAGAAATGGAAATGTGGGTCGAGGGCAGGCTGTTCAAGATC
ATACAAGATACTAAATGAAATACTGGGATTGGGAATAATTGATAGTTAGAAATATTTGTA
TTACTTAATAATTTGATTACTCTATTGATTGCAACGAAATCTTTCATAATTGAATTTGAT
TTCGAGTTAGCAACTTCTCGTCTATTTATTTTTCATTCCTTTCTTCTTCGCTTCGGTTCG
AATCGAAAATAGAAGAATTGAGTGAATTCAAAATCCAAAGGAGGTTCATGGCTAAGGGTA
AAGATGTCAGAGTAGTAGTTATTTTGGAATGTACCAGTTGTGTCCGAAATGGTTTGAATA
AAGAATCGCGGGGCATTTCCAGATATATTACTCAAAAGAATCGACACAATACACCTAGTC
AATTGGACTTGAAAAAATTCTGCCCTTATTGTTACAAACATACGATTCATGGGGAGATAA
AGAAATAAATCGAACGGAACGCGTGTGCCACTCTTCCAAGGAAGAGGAAGAAATTACATA
TATATATATATAATATATACAAATCCAGTCCTATTTTGGTCGGATCCGAGATGAATGAA
GAAATAGGATTTTAGAAATAAGAAATAAACCATGGATAAATCCAAGCGGCCCTTTCATAA
ATCCAAGCGATCTTTTCATAGGCGTTTGCCCCCAATTGGATCGGGGGATCGAATTGATTA
TAGAAACATGAGTTTAATTAATCAATTTATTAGTGAACAAGGAAAAATATTATCTAGACG
AGTGAATAGATTAACCTTGAAACAACAACGATTAATTACTATTGCTATAAAACAAGCTCG
TATTTTATCTTCGTTACCTTTTCTTAATAATGAGAAACAGTTTGAGAGAACCGGGTCGAT
CCCTAGAACTACTGGTCCTAGAACCAGAAATAAATAAGCCTATTCCTCTCAATCGAATCA
AAACTCTAATTCGAACTCAGATTGAAGTTTTGTTCGAAAAAGCCGAGAGATTGTCGCGCC
GTAATGTAATAATAAAAAAAAGAATGGGGGAAGAATAAATCTTTTTTTTTTTTATTGAAAC
GTGTTCGTTCATTCCTACTACTTATCTTATCATACGAATTTCTACTATACCCTCCCGGAG
TTCATTCTCCGGGGAACTCCGTTTAAAGTATTCCAGTGAATTCCTTCCAATCTCCTTATT
TGATGATCGCATTGGAAATCGTGTCAAGACAATTCCTATTTGATATGGCTATTTGTGCAG
GTATTTTACGATTAAGAAGCAACTGCCTCTTGTACAGATCAAGTATTAATCGACTATAAC
TATGGGATCCCCTATTCTCACGAGTTACTGCATTTATCCGAGTGATCCACAAACGACGAA
AACTTCTCTTTCGCCTGCCTCTATCCCGATGAGTGGAAACCAAAGCTCTCATTTTCTGTT
GAGTAGTAGTTCGAGTAAGTCTTGAATGAGCCCCTCGAAAGGTTGCTGCAAATAAACGAA
TTTTTGTTCTACGTCTCCGAGCTATATATCTTCGTCTAACTCTGGTCATTGAATCAAAAG
AAACTTTGATGAATAACTAATTGATTTCTTTTCTTTCAGTCATTCTTTTCCCCTCTCCTG
GTTTATTAATAACAAAACGGATTCTTCCGATGTATAAAATTAAAATAAAAATTCCAATGG
CTTTTGCTACTATAACCTTCCCAACCACGATTTTTTTATTTCTTCCAGGCATTTCACCTC
AAAAAAAAAAAAAAAAGAAATTGTACCGATATTAGGTATAAAATAAATCGTAAATGGACA
AATAGTGGCTTCCATCGTTTCTATGGTTACTTCTTAAACGGCGAGGTCCTCTCTATACAC
CGGAGCCCCTTTCCTCATTTAATCAATGTTATTGGTAACTTGTACAGTTCACGCTCTTTG
GCTCTACCGATGAATTATCGAGTAATAGGTCTTTTTTCAATGGGATCTATCCATACAGTG
ACGGCATTTAATTATGAAGGTTGAATAGGTAGCTGACCCTGTTAGTCCGTTCTTGCAAGA
GTAGGAGCATAATCTTTCTGCTTCTTAAATATCATTCCCCCGCTTAATGGATAACCATTT
GCTACCAATGGGAATTGCTTTTCATCTCAAATCGAGGTGATTGGATTTGCACCAATGGAA
ACCATAAATTCCATACAAATAGAGGTATACGAGAGATCTTTATTTTTCGATAGTGAATGG
AGTTCTTCCATTCTATTCATTGGTACGAGTCATTGATACTGTAAAAGTCGTCTCATTTGT
TCTAGCTCATGATCTGAACGAGTCGCACATACACCCTAGTACATGTTCCTCGACGCTGAG
GACATCCTCGAAGAGCGGGGGATTTCGTGACATTTCTGATTGGCTGTCTTGTGTTTCTAA
TAAGTTGTTTAATAGTTGGCATGCTGAATCATATACAGAATGGGCTGGTTTAGATCGATC
CTAACCGGATGATTATGAATTACTTCTTTACCCAGGTAAGAAGATAAAAGATCAAATAAG
GGTTCATTCAAATTATGATTCGAAATGGAATCAAAGATTTATGCGAAATCCCCGGTATTT
TCGATCGCTACAAGATCAACAATGCCATAATCTTGGGCTTCTGTTGCTGACATAAAAACA
TCCCTTTCCATGTCTTCGGATACAACCCATAAAGGATTGCCCGTTCTTTGTACATAAACC

CTTGTGAGGGTTTCGCGGAGTTTCAGTAGTTCTTCCGCTTCCAGGATAAATTCTCCTGTG
GGTGCCTCATAAAAAGAACTAGCAGGTTGATGGATCATAACCCTGATGATATAACATAAT
GAACGATTCCTCTATCTCGCATGATTGGGCGAAGGGAAGGGATAAAGAATAACAAGCAGG
GAGAAAAAGATAGAATTGAACAACCGTACAGGCATCTTTTGTGCATACGGCTCTGTAATG
GAATTTTTTTTTCTCTTTTTTCATCGAAGAAAGAGACAAGTCGAATCTATCAGACCCAGA
TCGTTGAATGATCCATTTACCATCCTTCCTTTCGGAGTAATCAAAAAATACTATGATGGT
TCCGTTGCTTTATATATTTATCTCGTCTGTGATTCAGCAATCCCAAAGTTTCTTTCTGAT
CCGATCAAATAAAAATAAGTAAAAAATGATCTTTTTTTTTTTTTTTATTTTCACACTCTT
TCATAACATAAATATTGGAAAGAGACTTCTGATGTGGAAGCAAAAAGGTTTGTGACGCTG
AAATGGACCCCGATACATAAGATCAAGTCGGAAATAACCTTTCTTTCATACTACTATCTC
GATACATAATCTCATATTATGAAAAAATAATAATAGTTTGCTCATATCGAACTTGAAATG
CCATGCTATTATTACTTAATATTATTATTATTTTATTCATATTCCATATGACGAAGGCAT
AGTCTTTTTTTCTCTCAAATAAAAAAACTCATTGGCGCCAAGCGTGAGGGAATGCTAGAC
GTTTGGTAATTTCTCCTCCAACCAGGATGAAAGATCCCATTGAAGCGGCTAATCCCATGC
ATATTGTATGCACATCTGGTGGCACAAATTGCATAGTATCATAAATAGCTATTCCTGGGA
TTACCCATCCGCCGGGAGAATTTATAAACAAATACAGATCCCTGGTATCATCCTCTATAC
TGAGATATACCATGAGACCAACAAGTTGATTCGAGATCTCGCTATCAACTTCTTGGCCTA
AAAAAAGTAATCTTTCTCGATGAAGTCGGTTGATTAGGACAAAATTCTATTCCTTAGGAA
CCGTACACGCACCTTTGGGTGCATACGGTTCAAAAAATCAAAATTGAGAAAAAAAAAAAA
AGAAATTGTCGATTCCAGCCCTATTTCTTTTTTCGTAGCGGGCTTTTTCTTCCATTTTTT
AAGAAACATGAGTTTTGACTTGCTTCCCTATAAAATCAAAAAAGAATTCACTGAACTTAT
CGAGCTAACCCCTCATTGATGTATTGTTTCATCGAGATCTAAATCACGATGTAATTTTCT
TGTTCCCGAATGGGCCTCTTCCACTCTTTTAGGTTTATGCTCTACTCCGGGTAAAGATCT
GCCCGAATTCGATTTGCACATATAGGACAAATGATCCCAGTACCACTTCTTTTTGCTATG
ACTTCTTTTTTTTTTTTCAATTTGTTTCATTTTCATGCCTTCCACAAAATATTCGATGT
ATTCATCATATTATTCCATTAATTGGCAATTTGGGATCACTCATATGGTATAAAGGAATC
ATTTCTGATAGGGTGGTAATCATACATGGATTACCTTGGTATTTTCTGAACGGAGCCTGT
ATACTTCATTTTATTGGTCCAAGCCAACCATAAATTCTTTTAATTGAGAATATTGATCCT
CCAACCAAATAAATTGATCTAATTGCACTTCACGCTTCGAATTATTGATGGTTCAATCAA
TCTTTCTTGGGCGAAACAGAGGATATCTCGATCGGGGGAGAGAACGGGGAAATCCCATAT
GACCCAATATGTCTGACAAGTCACACTATACGTCAACCCAAACTGCATCTTCCTCTCCAG
GACTCCGAAAAGGTACTTTTGGAACACCAATGGGCATTAAATGAAAGAAAAATGAAGTAT
TCTATTTCACTTTGATGTGGAAACGTAACAAACAATGGTTTATTGTCTTCATAATATTGT
CGTTTATCGTATTTTATCGATAGATTGGAAGATTCATAGAGGAAGACGGAATAAAGGAAA
ATTCTTACGAACGGATCGTTCGAATGAGAAACAAGTATCTATACATTCGCTCACAAAAAA
TAGGATTAATCCCCCCATTGCGTATTGGTACTTATTGGGTATAGAATAGATCTGCTTCTC
TTTGTTCCTACGAACAGAATTGTTCAATTATTACTAACGGAACAGAATAAATATTAACCC
TTGTTTCGAGATAATCCAATGAAAAGGTGAGGTCCATAGCATAGTTATTTCCAATGTGAT
AAAGTTACATAGTATCTATTTTATCTTTGAGAAAGGGGTATTTCCATGGGTTTGCCTTGG
TATCGTGTTCATACCGTCGTATTGAATGATCCCGGTCGGCTGCTTTCTGTCCATATAATG
CATACAGCTCTAGTTTCCGGTTGGGCCGGTTCGATGGCTCTATACGAATTAGCAGTTTTT
GATCCTTCTGACCCCGTTCTTGATCCAATGTGGAGACAGGGTATGTTCGTTATACCCTTC
ATGACTCGTTTAGGAATAAACAATTCATGGGGCGGTTGGAGTATTACAGGAGGAACTATA
ACGAATCCGGGTATTTGGAGTTACGAAGGTGTGGCCGGGGCACATATTGTGTTTTCTGGC
TTGTGCTTCTTAGCAGCTATCTGGCATTGGGTGTATTGGGACCTAGAAATATTCTGTGAT
GAACGTACGGGAAAACCCTCCTTGGATTTGCCCAAGATTTTTGGAATTCATTTATTTCTC
TCAGGGGTGGCTTGCTTTGGGTTTGGCGCATTTCATGTAACAGGCTTGTATGGTCCTGGA
ATATGGGTATCCGATCCTTATGGCCTAACCGGAAAAGTACAATCTGTAAATCCAGCGTGG
GGTGCGGAAGGTTTTGATCCCTTTGTTCCGGGAGGAATAGCCTCTCATCATATTGCAGCA
GGTACATTGGGTATATTAGCAGGTCTATTCCATCTTAGTGTCCGCCCACCCCAACGTCTA
TACAAAGGATTACGTATGGGCAATATTGAAACTGTCCTTTCCAGTAGTATCGCTGCTGTC
TTTTTTGCAGCTTTCGTTGTTGCTGGAACTATGTGGTATGGTTCAGCAACTACCCCGATC
GAATTATTTGGTCCCACTCGTTATCAGTGGGATCAGGGATACTTCCAGCAAGAAATATAT
CGAAGAGTTGGCGCCAGTCTAGCCGAAAATCTGAGTTTATCGGAAGCTTGGTCTAAAATT
CCCGAAAAATTAGCTTTTTATGATTACATCGGTAATAATCCGGCGAAAGGTGGATTATTC
CGGGCAGGCTCAATGGACAACGGGGATGGGATAGCTGTTGGATGGTTAGGACACCCTATC
TTTAGAGATAAAGAAGGGCATGAACTTTTTGTACGCCGTATGCCTACTTTTTTTGAAACA
TTTCCAGTAGTTTTGGTGGACGGAGACGGAATTGTGAGAGCCGATGTTCCTTTTAGAAGG
GCAGAATCGAAGTATAGTGTCGAACAAGTGGGTGTAACTGTTGAGTTCTATGGTGGCGAA
CTCAATGGAGTCAGCTATAGCGATCCTGCTACTGTGAAAAAATATGCTAGACGTGCCCAA
TTGGGTGAAATTTTTGAATTAGATCGTGCTACTTTGAAATCCGATGGTGTTTTTCGGAGC
AGTCCAAGGGGTTGGTTCACTTTTGGACATGCTACGTTTGCTTTGCTCTTCTTTTTCGGA
CACATTTGGCATGGCGCTCGAACCTTGTTCAGAGATGTTTTTGCTGGGATTGACCCAGAT
TTGGATGCTCAAGTGGAATTTGGAACATTCCAAAAACTTGGAGATCCAACTACAAGGAGA
CAGGTAGTCTGATACAACATTGCTCCGGTATCTTTCGCCTCTATATTTGATTTTTTTGAT
TTGACATAAGGTACCGTAGAAATATTGATTTGAATCATCGCCTTTCTTTGCTCTTGTCCT

TTCTTTATCTGGGAAATAATCCTAAATGAACAGGTGTGGAAGCTATAATTGTAAACAACG
ATCGAATCTATGGAAGCATTGGTTTATACATTCCTCTTAGTCTCGACTTTAGGGATAATC
TTTTTCGCTATATTTTTTCGAGACCCGCCTAAGGTCCCGACTAAAAAGACGAAATGATTT
TTCATTATCTTAATTGAAGTAATGAGTCCCCCATATGGGGGACTCATTACTTCAATTAGT
CTCCGTGTTCCTCGAATGGATCTCTTAGTTGTTGAGAGGGTTGCCCAAAAGCGGTATATA
AGGCGTACCCTGTAAAGCTTACAAGTGAACCAGATATGGAGATGGCGACTAAGGTTGCTG
TTTCCATTATTAGAGAATTTCAAGACCACGATGGATCTATGCTACGATAAGATCGTTTAT
TTACAACGGAATAGTATACAAAGTCAACAGATCTCAACCAATGCAATAGTATTTATGGCT
ACACAAACCGTTGAGGGTAGTGCTAGATCTGGGCCAAGACGAACTATTACAGGGGATTTA
TTGAAACCATTGAATTCAGAATATGGTAAAGTGGCTCCTGGATGGGGAACCACCCCATTT
ATGGGTGTCGCAATGGCTCTATTTGCGATATTCCTATCTATTATTTTAGAGATTTATAAT
TCTTCCGTTTTACTGGATGGAATTTCAATGAATTAGGTCCATAAGAACCAGAAGCCCTAG
CTTTTCAATCAAAAATGAATCACTTAGGACTCAGATTTATAGTCCATTCTGGTAGTTTGA
CCGTGGAATTCCGTTGTTTCGGTATTTCCGGAATATGAGTGTGCGACTTGTTATAATTGA
TCCTATTGATAGTACAGAGAATGGGTCTGTCATCTCGACAGAGATGGTTCTGCCTCGTCG
GATATTCATCCTAGTATCTGGAGCACGGAATATATGGAATAGATCAAGAAATATTTGAAC
TATGATTCATACCTACTATTCAGACCTCGTGACTGGACTTCAAAAAATTTTCAAACAAAG
AGGTATTTGATAAATTGAACGATTTTTCTTCCTTTAGAATCATGCTTATTTTGACCGAAG
GACAAATCTTTCTCTGGATTTTTAGTCATTACATCTATGAATAAGTGATGATCAAATAGT
TCTTACTCATAGAACCCTTGGTCTTAGTTTTTGGGTTTTATTGAATCATCGTGGTTCTAG
TATGAATCTGAGGTTTCAATCGATTCATAGGGTCTCAACAAGAGAATTCCTATCAAAAAA
AAAATAGTAAACAATAGTCAATCTGCATTACGCACAAACAAAAACAACAAATCAAATAAC
AAATAAATAGGGGAATAGAAGATTCAAGAGGCCTGTAACGATCAACATAAAGACAGATGA
GCTAACTTGATATTTTGGCATTCTCATCACAACAAAGAAGAGAGTTCGGATTTTGGTTCC
TTCGTATCTTCAGAGACGATTGAATCAAGTGGATAAATAAGAAATTTCAAATTTTCTATT
ACATATCCATTGTAATCAGTATTTGGGTGTTTCTGCTTGAGCCGTACGAGATGAAATTCT
CATATACGGTTCTCAGAGGGGGAGTCCCCTTGGTTTACCTATCTCAATAAAGTATATGAT
TGGTTCGAGGAGCGTCTCGAGATTCAGGCGATTGCAGATGATATAACTAGTAAATATGTT
CCTCCTCATGTCAATATATTTTATTGTCTAGGAGGGATCACACTTACTTGTTTTTTAGTA
CAAGTAGCTACGGGTTTTGCTATGACTTTTTACTATCGTCCGACCGTTACGGAGGCTTTT
GCCTCTGTTCAATACATAATGACTGAAGCCAACTTTGGTTGGTTAATCCGATCAGTTCAT
CGATGGTCAGCAAGTATGATGGTCCTAATGATGATCCTACACGTATTTCGTGTGTATCTC
ACGGGCGGATTTAAAAAACCTCGCGAATTGACTTGGGTTACGGGTGTGGTTCTGGCTGTA
TTGACTGCATCGTTTGGTGTAACTGGTTATTCCTTACCCCGGGACCAAATCGGTTATTGG
GCAGTAAAAATCGTGACAGGCGTACCTGAAGCTATTCCCGTAATAGGATCACCTTTAGTA
GAGTTATTGCGTGGAAGTGCTAGTGTGGGTCAATCTACTTTGACCCGTTTTTATAGTTTA
CACACTTTTGTATTACCTCTTCTTACTGCCGTATTTATGTTAATGCATTTTCCAATGATA
CGTAAGCAAGGTATTTCAGGTCCTTTATAGAGAAGACAGATCATAGATATTTGTAATCGA
TCATATATAATTTCGGGGAGGAACAATAGTGTTTTATTGCTACAAATATGGATTATTGAA
AAGAATAAGACATCTTTTTGGATATTTCTCTTCAACTAACTACGAAGTATTGTATTCTTT
ATTTGATACGAATAGTTGAAGTACATTCTCCGAAGAGAAGATGGATTATGGGAGTGTGTG
ACTTGAACTATTGATTGGGCCGTGCAGATATATGATTTTATCCGCCACATTGGAATTCAC
AACCAAATGTGTCTCTGTTCCAACCACCGCGTAGGTCCCCCTACAGAGGATAGGCTGGTT
CGCTTTAGGAGAATCTTTTCTATGATCAGACCAAATCATGTTATGTTGTGCATGAACGGG
CTCCGTAAGATCCAATAGAATAAAATAAAATGAAGTAATGTGGCATGATCCAGATTATGT
TTTATCTATTTACTTAAAGTATGGAAATGCATTCATTTCCTCTGCATCGATCCCAATCTA
TGATACTATCGGAGTGAAACAAGGGATCTAAGGAAGAACATAGGCTAGACTTTATTAGTA
ACAAGGAAATCCTTTGTATTAAGAAGACTCGAGATATTGTGGGGATAAACACTAATCACA
AAGCATGAGACCATCCAAAAAGCATTTGATCATGATCAAATTTGGAAGCCTACTTGGGTA
TTGAGCATTTACTTGTAAGAACTGAATTCCTTGCAATGGGTAGTTGCAACCCCGTAAAAT
TGAATCCGGTAAATCTTTTCTTACATAGAGTCATATATGTGTGGATGATATATCTATTTT
ATATGGACCCGTTTTATTCTTTTGATTCTTGCTCGAGCCGGATGATAAAAAATTATCATG
TCCGGTTCCTTCGGGGGATGGATCTATAAGAAAGAATTCACCTATCCCAATAACAAAGAA
ACCTGACTTGAATGATCCTGTATTAAGAGCTAAATTGGCTAAAGGGATGGGGCATAATTA
TTACGGAGAACCCGCATGGCCCAATGATCTTTTATATATTTTTCCAGTAGTCATTCTAGG
CACTATTGCGTGTAACGTAGGTCTAGCGGTTCTAGAACCGTCAATGATTGGTGAACCAGC
GGATCCATTTGCAACTCCTTTGGAAATATTACCCGAATGGTACTTCTTTCCCGTATTTCA
AATCCTCCGTACAGTACCCAATAAGTTATTGGGTGTTCTCTTAATGGTTTTAGTACCAAC
GGGATTATTGACAGTACCGTTTTTGGAGAATGTTAATAAATTCCAAAATCCATTTCGTCG
TCCAGTAGCTACAACAGTTTTTTTGATCGGTACCGCGGCAGCCCTTTGGTTAGGTATTGG
GGCAACATTACCTATTGATAAATCTCTAACTTTAGGCCTTTTTTAAGTTGATTTAACCGT
GAAATACTACGCGTATGTATCTAGGGAATAGTCACTTCTAAAGTGAATTCTCCCTAGATA
CATCTCTTAAATTTCATTATCAATCCATTCTGGATATAGAGATGATACTAAGGATTCAAA
AGCCATTTTCTTCTTTTCTTTCTTTCAAAAAAGATGAAATAATACCAATGGATTTAAAAC
TTATTCTTAGGTAAATAAATTGCAAAATGCTTCTGTAGAATGTCCAATATCTGTTTTACA
TCTTCTATGCGAAGATGTTCAATTCTCATAAGATCTTCTTGACTGTTATTCAAAAGGTCC

AATAATGTATGTATATTGGACCTTTTGAGACAATTATAGGTCCTGGAGGGCAATTCTGAT
TGGTCAATAAAAATACATTTCAGTGCAATTCCTTTTTTGTTTTTCCTTATATTAGCCAAT
CTATCATGAAAAGTAAAAAACGATACAGTAAACCTGTTTTGATTGTCTTCTAAATAAAAA
TGAATGTCCTCTTCCTCCGCATGTAGAAAAGGAATAAATAAGTCAATCAAAGTACGGGAA
GCTTCGCGAAGTGCTTCTTTAGGAGTTAAACTTCCATTCGTCCATATTTCAAGAAAAAGT
ATCTCTTGTTTCTCATTCTCACTCCCATAAGAATGAATACTATGATTCGCATTTCGAACA
GGCATGGATACAGCATCTATAGGATAACTTCCATCTTGATAGTTATTGGGGGATTTCATA
CGATATCCGCGATCCCTCTCGATTTTGAATTCAATACACAAATGAATTGGCTCCGTCAGA
TTAGCTATATGCTGTGTAGTATCGACTAGTTCCACAGAAGGCGGTGAGATGATATCTTGA
GCAGTTACGTATTTAGGACCCCTGACGCAAATGGATGCGTCACGAGTTCCATACAGATTA
CTTCTCAATACAATTTCTTTCAAATTCATTAAAATTTCATGTACTGATTCTTCAATACCA
ACTATCGTAGAATATTCATGTGATACCTTCTCAGATTTTGCACGTGTGATACATGTTCCC
TCTATTTCTCCGAGTAAAGCCTTTCGCATCGCGATACCTATCGTATCTGCTTGACCTTTC
ATAAGCGGGGACAGAACGAAACGGCCATAATAAAGACGCTTACTGTCTGTTCTTGATTCA
ACACACTTCCACTGTAGTGTTCGAGTGGATACTGCTACTTCTTCTCGAACCATACTAATA
TTATTGTTTGATCAGATCATTGAATCATTTATTTCTATTGCAATCCATTCAATTTTTATT
TCTACACACGTCTTTTTTTAGGAGGCCTACATCCATTATGTGGCATAGGGGTTACGTCAC
GTACGAAACTTAATAGTATACCACTTCTACGAATGGCTCGTAATGCTGCATCTCTTCCGA
GACCAGGGCCTTTTATCATGACTTCTGCTCGTTGCAGACCCTGATCCACTGCCTTACGAA
TAGCATTTCCTGCTGCGGTTTGAGCAGCAAATGGTGTCCCTCTTCTTGTGCCTCTGAATC
CACAAGTACCAGCGGAGGACCAAGAAACCACCCGACCTATTACATCTGTAACAGTCACAA
TGGTATTGTTGAAACTCGCTTGAACATGAATAACTCCTTTTTGTATTCTACGTCCATTCT
TACGTGAACCAATTCTTGGTATAGCTTTTGTCATATTTTATCATCTCATAAATATGAGTC
AGAGATATACGGATATATCCATTTCATGTCAAAACAGATCCTTTATTTGTACATCGGACC
GTTTAGAAAGTCCCTTGTTAGAAAGATTACCCCTGTCTCTGTTTATGTTTCGGATTGGAA
CAAATTACTATAATTCGTCCCCGCCTACGGATCAGTCGACATTTTTCACAAATTTTACGA
ATGGAAGCCCTTATTTTCATATTTGTTATTCCTTAATTCCAAATATACTCCTTGGAAGAA
AATAAGTCTCTTGAAATTTTGAACCTCGAATTGTATTCCCATGAAAGGAATGTTTAAATT
CAAATAAAAAGCCGCCTAATCATTCGACTCTTTGTTGCGAAGTCTATAAATTATACGTCC
CCTAGTTGAATCATAACGACTTACTTCGATTTTGACTCTATCTCCTGGTAGTATCCGGAT
AAAACTCCGTCGGATCCTCCCCGAAACATAACCTAGAATCAGATCTTCATTGTCTAAACG
AACTCGGAACATACCATTGGGAAGTGATTCAGTAATTAAACCTTCGTGAATCAATTTTTG
TTCTTTCATTCCAGGTAACCCCCTTGAAGTATCAACTAATGGAGGAGGAGTAATAGTAGA
CAATTCGTCTTTCCTCTCTTTTTCCCAAATAGCAAGTTACGGATCAAATTCGGATACCAG
AAGGATCACCAGATATAATACAAAATTTCTCCCCCAATTCTTTCTAGTCGAGCTTCTCGA
TCTGTCATTATACCTCGAGAAGTAGAAAGAATTACGACCCCCATTCCACCTAAAATCCTA
GGAATTCGTTGATGGTTGGAATAGATTCGTAGACCGGGACGACTGATACGCTTTAAAATA
TTTCTATATGTTCCTTTCCTATTCCTTCTATGTCGCAGGGTTGAAACCAAGAAATATTTG
TTGTTTTCCCTATGTTTCCTAACATTTTCAATAAACCCTTCTTGTAGAAGTATTTTAACA
ATGTTTTCGGCGATATTAGTAGATGCTACTCGAACCGTTCCTTTTTTATCCATGTTAGCA
TTTCTTATAGAAGTTATTATATCGGCAATAGTGTCCCTACCCATGACGAACTAAATTTAT
GGGTGCCTTCCAGTTTTGATATAATCAACATGTTCCTTTTTTTTCATTTTTTCTTAT
TTATTTATGAATTATTAAAGGTATATGCGTGAGACACAATCTACTAACGTAATCTATTTC
AGAGACCTGACTATACTCTATCACGGTCTCATCTACTAGTATTTATAAGACTTCAGGAGC
TAATGAGACTATTTTAGTGAAATTCAACTGTCTCAATTCCCGCGCGATCGCTCCAAAAAC
TCGAGTTCCTTTTGGATTTCCTTCTTGATCAATGACAACTGCTGCATTGTCATCATATCG
TATTATCATACCGTTGTCGCGTTTGAGTTCTTTACATGTACGTACAATTACAGCTCTGAT
CACTTCTGATCTTTCGAGAGGCATATTGGGCACTGCTTCTTTGATTACAGCAACAATAAC
GTCACCAATATGAGCATATCGTTGATTACTAGCTCCTATGATTCGAATACACATCAATTC
TCGAGCCCCGCTGTTGTCCGCTACATTCAAATGAGTCTGAGGTTGAATCATATCATTTTT
TTTTTTTTTTGAATCTGCTCTTTCAATGCAAAAGGCAAAGGAAAAAGAGAGAAATATTGT
CTGCCCAGAAATCCAAAAATCTGCGATTGTATTTTTCATCACAAATACCCTTCACATACC
TATCACGCGATAATGAATTGAGTTCGTATAGGCATTTTGCACGCAGCTATTGAAATAGCT
GCTCTGGCTACAGTTTCTGATACTCCGCCCATTTCATAAAGTATTCGACCGGGTTTAACG
ACAGATACCCAATATTCGGGAGATCCTTTCCCCGAACCCATACGTGTTTCGGTAGGTCTT
ACTGTAACGGGTTTGTCGGGAAATATACGTACCCATATTTTTCCACCACGGCGTGCATAT
CGTGTCATTGCTCGTCGTCCTGCTTCTATTTGTCTAGATGTGATCCAAGCGGGTTCAAGT
GCCTGAAGAGCGTATCTGCCGAAACAAATATGATTGCCTCGATAAGATATTCCCTTCATT
CTTCCTCTATGTTGTTTACGGAATCTGGTTCTTTTGGGGTTATAGTTGATGGTTGTTTCT
CAATCCCATCTCTACTGCAGAACCGGACATGAGAGTTTCTTCTCATCCAGCTCCTCGCGA
ATGAAACGATTCAATAAGATTACGTATATGTATTTATTGAATGAATAATACACTGAATCA
TGGAATTTCTTGATATTTAATCTGTCACACGGGAAGCCGTATAGTATATAGTATATACGG
CTAGACGGATATTTCTATTTTATTTATATGGGATAATGCCTTTCTTTTGAAAATGAATCC
TTGACCTTTACCGAATCTGTCAAAATACTACAATCCAATAATGGTTTCGCGGGCGAATAT
TGACTCTTTCCATATTTGCTTCATTCGTAGGGTGAACCCATGACCTATCAGAAGAAATTA
ATTGGTTCCTGGTTGATTCCGCCATCCCACCCAATGAATCATTAGGATTCGTTTTCAATA

GAATCTTCCGCAGTCACAGGTTTCGTCGTTCCCATAGCTTTTCCATTAATGGCTAGGCCT
GAACTATGCAATGGAGCTCCTAATTAAATTCGTTCCCGAGCCAATCTCCTCAGTCTCTAT
TGACTCGGGGCTCTTTATTATTTGTATTTTTCTTATGAACCGTATTCATCTAATTATGGA
CGAATCAGTATTGATGCTTTATCACACTGCCTTTTATGATATGATGTGATTGATAGACCA
TACATATTGGAATCATATATCATGGAGATTCTCCTTCTCTCTTTCTCTCGCCCTTCCAGT
TACCCACATCCCTCTATTTTTCTTTCCAACCTATAAATGGATTTTTCCTTTATGGAAAAA
AAAAGATTTCAGTTGCTACAACTATATGATCGATACATCATATGGCGACTGCTTCCTTGG
ATCTCGATAATACAAAGCAATGAGTTGGTTACTAGTTCTTATAGTTATTAGTTAGGGGCT
GGTCTGTTTTTTGAATCCCAACTTTAAATAAAAAACCAACGAGTCACACACTAAGCATAG
CAGTTCCACCAAAAGGTCAATCGAATTTTTATTCAACCTTATAGAATTAGAATTGCTCAT
TTTTCATTTTTTTTTTTTTATTGAAGTGAAAAGGAATAGTTTGTAGTTTTTGTTCTATCAC
TGAATAGAATGGCAAGCAAAGGAAGGGTCCATTATTGCTCGTCTACAAATATCCAAATTT
TGATGCCCAATGCCCCATAGGCAGTTCGAACTGTATAGGAACAATGATCAATTTTAGCTC
GAATGGTTTGGAGGGGAACCCTACCTTCTCTGATCCATTCGACACGTGCAATTTCTTTTC
CGTCGATACGCCCTGCAATTTCCACTTGAATTCCTTTTGTGTCTGCTTGTTCAGTTAATT
CAATAGCTTTTTTCATTGCCTTTCGAAAAGAAACCCTATTCTTTAATTGTAGAGCTATAT
ATTCTGCAAGAATATTAGGTTGTCCATAAGGTTTTGCAACTCTTGTAATAGCAATGTTAA
GTCTCCGATTCACAGAATGAAGCCCCTTTTGTACATTGATCTGCAATTCTTCGATTCCTC
GTGTTTGGCCTTCTATTAACAAATTTGGGAATCCAATATAGATTATGACCTGGATCAAGT
CCATTTTTTTTTGAATCTCTATATGTGCAATTCCAATTCCTTCGAAACTTGAAGATACTC
TTATATTTTTTTGTACATATATCTTGATCCAATCCCGTATTTTTTCATCTTCCTGGAGAC
CTATGTAATAACTTTTTGGTTGTGCGAACCAAAGGGAACGATGACTTTGGTTTTCGCCAA
GGCGGAAACCAAGTGGATTTATTTTTTGACCCATCTTTTTTTTCTCTCTCTCTCTCCTTC
TCTATATATCTTTCTATTATAGGATCTCTCCATACATTTTTTGTTTCTAAAAATATATCC
TGATCTGTTTTCTTTTTAGAGCTATCCTTCAATACAATAATTATATGACAGGCGGGTCTT
TCTATCGGATAACTACGTCCTCTAGCCCTGGGTTTTAACTTTTTCACGATAGTACCCCCA
TTGACTTCGGCTTTACTAATGACCGAATCAGCTTCGTTGAAACTTTTATTGTGACTAGCA
TTTGCTGCTGCAGAATAAACCAGTTTAAAAATGGGATAAAATGCTCGATAAGGCATGAGT
TCCAGTAACATAAGTGTTTTCTCATAGGAATGCCCACGAATCTGATCAATGACTCTTCGT
GCTTTGTGAGCAGACATACATATACGTTGAGCTAAAGCTTGTACTTGTGTACTCGAGCTC
CTCTTCATCTTCTGCTTTTTCAAGGTCTTCTTCCACTTTCTCCACTTTGACATAAGATAA
GGTTCGCCTCCCGCCAATGAACGATGAGCACCTATTTCACTTTATTTTATTAACGGAGAG
ATCTAGTATCGTTTCTCGCGTGTCCCTGGAAAGTAAGAGTAGGTGCAAATTCTCCTAATT
TTTGACCCACCATACGATCCGTTATATAAATAGGTAAATGCGCCTTTCCATTATGAATGG
CAATTGTATTGCCGATCATTGTGGGTATAATGGTAGATGCCCGGGACCAAGTTACTATTA
TCTCTTTTTCCTCTCTCATGTTAAGCTTCTTTATTTTTTCCAATAAATGATTAGCTACAA
AAGGATTTTTTTTTAGTGAACGTGTCACGGCCTATTATTCCCCCCCCCTTTTTTTTTTTT
GAAAAGACGAGTAAAAAACAATATTTATTTGATTTTGAATATTCCTATTTACGGCGACGA
ATAATAAAACTATTACTATATTTATTCCTTTTCCTACTTTTTCTTCCAAGCGCAGGATAA
CCCCAAGGGGTTGTGGGTTTTTTTCTACCAATCGGGGCCCTCCCTTCACCACCCCCGTGG
GGATGGTCTACAGGGTTCATAACTACCCCTCTTACTACAGGACGCCTACCTAGCCAACAT
TTAGATCCGGCTCTACCCAAACTTTTCTGGTTCGCCCCAACATTACCCACTTGTCCGACT
GTTGCTGAGCAGTTTTTGGATATCAAACGGACCTCCCCAGATGGAAATCTTAATGTGACC
GATTTACCCTCTTTTGCAATCAGTTTCGCTACAGCACCTGCTGCTCTAGTTAATTGTCCA
CCCTTTCCAAGTGTGATTTCTATGTTATGTACGGCCGTGCCTAAGGGCATATGGGTTGAA
GTAGATTTTTCTTTTTGATCAATAAAAACCCCTTCCCAAACCGTACAAGCTTCTTCCAAA
GCATACGGCTTTCCGGATGTATATATATATTCTATGATGATATCTAGACAGATGGATTTT
ATATGAATCGTGTGATGAAGTACCACATGAGTGGATATATAGGAATACAAATCTGCCAAA
TCACTCATGTTATGATCTTATACATCCTAGGTCTCTCCGTTTCGTCATCTGGCTTATGTT
CTTCATGTAGCATTCAGACCGAATGACTCTATGAAATTACGTCGCTACTTCCACATATTA
CGGGTAACGTAGGAGACATCTCTATTTTTCCCCGGGGGAATTTTTAGAATTACCACCACT
TAGCTTTCAATTCACCTCTGACCATCAAATGAAATGTGAATAACCCATCCTCTTCTCTTT
GAAACAAGGGTCGCTTCCGCTTCTGTCCGTGCTTCAAACAATTTTGTCTTCTCCATATTA
CCATATCTGGAGTGTCAATAGTTTTCTATGAGGAACTACTGAACTCAATCACTTGCTGCC
GTTACTCTTCAGTTTTCTGTTGAGGTCTATCCCGTAGAGGTACTCAAATTGGATCAGTGA
TCAATTTCTAGGTTTCGTCGTAAACCTAATTGGTTACTTCCAATTACGTAAATCCATAGT
TCAAACCGCACTCAAAGGTAGGGCATTTCCCATTGATATAGGAACTTCTGTACCGGAAAC
AATGGTATCTCCAATTATAGCCCCTCTGGGATGTAAAATATATCTTTTCTCACCATCTCC
ATAGTGTATGAGACAAATGTATGCATTTCGATTAGGGTCATATTCTATGGTTACGATCCT
AGCAGATATGTCTTTTTCATTCCGTCGAAAATCGATTTTACGGTATAGACGCTTATGGCC
TCCTCCTCTATGCCCTGCGGTAATGATTCCCCTGGAATTACGACCTTTACCGCAGCGATG
CTGTCCATAGATCAAATTATTTCGCGGATTGGATTTCGCTTGACTGTCTATGGATCCTTT
GCGTATGCTCGGGGTAGAAGTTTTGTATAAATGTATCGCCGTGTTATTTAGTATTTTTTT
TTTCTTTTTTTTTTTTTACTTAAATTCTTTTCTCTTCTCTATAAGAGGTAAAATAGAATAA
CCCGGTTGAAGCGTAATGATCATACGTCTGTAATGCATTGTATGTCCCATAATGGGTCCC
GTTCTTCTACCCTTTCCCGGGAGTTGATGACTATTTATAGCTATTACTTTGACGCCAAAG

AAGAGTTCGACCCAATGCTTTATTTCTGTCCTAGTTGATCCTGATTCGACTTTCTTCCCC
ACGAGTTCCAGTATCGATAAGAATTCTAGTTCTTACTCTTCATATGTTATGGTTGGTATG
AATATACCATACCAATTCGTTATGTATGGATGATGAGATTCCATTGATACGGAGCCAGTG
GAATTAGTCTTATTGAATGTCCCCGTTGGCCTGCATCCAGCAGGAATTGAACCTACGAAT
TCGCCAATTATGAGTTGGGTGCTTTAACCATTCAGCCATGGATGCTTCACTGGGGTCATT
GTACATCGCGAGTGACCCAAATTCAATTCACTTAGATTCTTTTGGATTCTTTAGGAGGAA
TCAATGAAATGAGAGGACATCAATTCAAATCCTGGATCTTCGAATTGAGAGAAATCAAGA
ATTCTCACGATTTCTTGGATTCATGGATCCAACCCGATTCGGTGAAATCTTTCACTTCCT
TTTTTTTCCACCAAGAGCGCTTTATGAAACTTTTTGATTCCCGAATTTGGAGTGTCCTAA
TTTCACGTGATTCACAGGGTTCAATTCGTCGACATTGCATGATCAAAGGTGTAGTACTGC
TTGTACTTGTAGTAGCGGTCCTTATATACAATCGAAATAGGGTCGAAAGAAAAAATATCT
ATTTGATGGGGCTTCTTCCTAAACCTCTGCGTTCCATTGGACCCCCCAATTATACATTGA
AAGAATCCTTTTGGTCTTCCAATCTCAATAGGTTGATTGTTTCGCTCCTGTATCTTCCAA
AAGGGAAAAATATCTATGAGAGTTGTTTCATGGATCCGAAAGAAAGTACTTGGGTTCTTC
CAATAACTAAAAAGTGTATCATGTCTGAATCTAACTGGGGTTCGCAGCGATGGAGGAATG
CGATCGTAAAAAAGAGGAATTCCAGCTGTAAGATATCGAATGAAATTGCAGCTGGAATTG
AGATCTCATTCAAAGAGAAAGATATCAAATATCTGGAGTTTTTTTTTGTATCCTATACGA
ATGATCCGATCCGCAAGGACCATGATTGGAAATTATTTGACCGCCTTTCTCCGAGTAAGA
AGCGAAACATAATCAACTTGAATTCGGGACAGCTATTCGAAATTTTAGTGAAACATTTGA
TTTGTTATCTCATGTCTGCTTTTCGTGAAAAAAGACCAATTGATGAGGGGGGTTTCTTCA
AACAACAAGGAGCTGAGGCGACTATTCAATCAAACGAGATTGAACATGTTTCCCATCTCC
TCTCGAGAAACAAGGGGGGTATTTTTTTGAAAAATTGCGCTCAATTTCATATGTGGCAAT
TCCGCCAAGATCTCTTCGTTATTGGGGGGAAGAATCGGCACAAATCGGATTTTTTGAGGA
ACGTCTCGAGAGAGAATTTGATTTGGTTAGACAATGCGTGGTTGGTAAACAGGAATCGGG
TTTTTAGCAAGGTACGGAATGTATCGTCAAATATTCAATATGATTCCATAAGATCCATTT
TCTTTCAAGTAACGGATTCTAGCCAATCGAAAGGATTTTCTGATCAATCCATAGATCCTT
TCAATTCCATTAGTAATGAGGGTTCGGAATATCACACATTGATCAATCAAACGGAGATTC
AGCAACTAAAAAAAAGATCAATTCTTTTAGATACTTCCTTTCTTCAAACGGAACGAACAG
AGATAAAATCAGATCGATTCTCAAAATACCTTTCCGGATATTCCTCAATGGCTCGGCTAT
TCCCGGAACGTGAGAAGCAGATGAATAATCATCTGCTTCCAGAAGAAATAGAAGAATTTC
TTGGGAATCCTACAAGATCAATTCGTTCTTTTTTCTCTGATAGATGGTCAGAACTTCATC
TGGGTTTGAATCCTACCGAGAGGTCGACTATAGATCAGAAATTGTTGAAGAAACAACAAG
GTGTTTCTTTTGTCCCTTCGAGGCGATCGGAAAATAAAGAAATAGTTGATATATTCAAGA
TAATTACGTATTTACAAAATACCTCCTCAGTTCATTCGATTGCAGCAGATCCGGGATGGG
ATATGGTTCCGAAGGATGAACCGGATATGGACAGTTCCAATAAGATTTCATTCTTGAACG
AAAATGCATTTTTTGATTTATTTCATCTATTCCATGATCGGAACAAGGGGGGATACAGGT
TGCACCACGAGTTTGAATTAGAAGAGACATTTCAAGAAATGGCAGATCTATTCACTCTAT
CAATAACCGAGCCGGGTTTAGCCTATCATAATAAGGAATTTGGCTTGTCTATTGATTCCT
ACGGAAAATTATTGAATGAGGTATTCAACTCCGGGGATGAATCGAAAAAGAAATCTTTAT
TGGTTCTACCTTCCATTTTTTATGATTTATTTTTATTGGTTCTATCTTCTATTTTTTATG
ATTTATTTTTATTGGTTCTACTTTCTATTTTTTATGATTTATTTTTATTGGTTCTACTTT
CTATTTTTTATGAAGAGAATGAATCTTTTTATCGAAAGATAAAAAAAAAATCGGTCCGGA
TCTCCTGCGGGAATGATTTGGAAGATCCAAAACCAAAAATAGCGGTATTTGCTCACAACA
ACATAATGGAGGCGATCCATCAATATAGATTGATACGAAATCAGATTCAAATCCAATATA
GTACCTATGGGTACATAAGAAATGTATTGAATCGATTCTTTTTAATGAATCGACCCGATT
GCAACTTCGCATATGGAATTCAAAAGCATCCCATAGGAATTCAAAAGCACCCAATAGGAA
ATGATATTCTGAATCATCTAACTATAATAATAGATAAGATCAACCAACATTTATCCAATT
TGAAAAAGATTAAGAAGAAGTGGTTCGATCCTCTGATTTCTCGAACCGAGAGATCCACGA
ATCTGGATCCTAATGTATATAGATACAAATGTTCCAATGGAAGCAAGAATTTCCAGGAAC
ATTTGGAGCATTTCGTTTCTGAGCAGAAACACCGTTTTCAAGTAATGTTCGATCGATTAC
GTATTAATCAATATTCGATTGATTGGTCCGAGGTTATCGACAAACAAGATTTGTCCAAGT
CACTTCGTTTCTTTTTGTCCAAGTCACTTCTCCTTTTGTCCAAGTCGCTTCTCTTTTTAT
CTAAGTCACTTCCTTTTTTCGTTGTGAGTCTCGGGAATATCTCCATTCATAGGGCCGAAA
TCCACATCTATGAATTGAAAGGTCTGAATGATCAACCCGGCAATCAGTTGTTAGAATCAA
TAGGTGTTCAAATCGTTTATTTGAATAAATTGAAACCCTTCTTATTGTATGATCATGATA
CTTCCCAAAGATCGAAATTTTTAATCAATACAGGAACAATATTACCTTTTTTGTTCAACA
AGATACAAAAGTGCATGATTGACTCATTCCGTACTAGAAAAAATCGCAAGAAATCCTTTG
AGAACACGGATTCCTATTTCTCAATGATATCCCACGATCGAAACAATTGGTTGAATCCTC
AGAAAAGTTCATTGATATCTTCTTTTTATAGAGCAAACAGACTTCAATTCTTGAATCATC
CCCATTGCTTCTGGTTCTATTGTAACAAAGGATTCCATTTTTATGGGGAAAAGACCCGTA
TCCATAATTATGATTTTACATATGCACAATTCCCCAATATCTTGTGCATTCGCAACAAAA
AATTTTCTTTGTGTTTCGGTAAAAAAAAACATGTTTTGGGAGAGAGAGAGACTATTTCAC
CAATTGAGTCACAGGTATCTGGCATATTCATACCTAACAATGTTTCACAAAGTGGTAACA
AAACGTATAACTTGTACAAATCTTTCCATTTTTCAATTCGATCTGATCCATCCGTTCCTA
TTTACTCGATTGCAGACATTTCGGGAACACCTGTAATAGAGGAACAAATAGTCAATTTTG
AAAGAACTTATTGTCAGCTTCTTTCAGATATGAATCTATCTGATTCAGAAGGAAAAACT

TGCATCACTATCTCCGTTTCAATTCAAACATGGGTTTGATTCACACTCCATGTTTTGAGA
AATATGTGCCGTCCGGAAAGAGGAAAGAACTGAGTCTATGTCTAAAGAAAAACGTTGAGA
AGGGGGAAGTAGGTAGAACCCTTCAACGAGATAGTGCTTTTTCAAATCTCTCAAAATGGA
ATTTGTTCCAAACCTATATGCCATGGTTCCTTACTTGGACGGGGTGTAAATATCTTTATT
TCACCTTAAAAAACAATATTTATTTGATATTGAATATTCCCTTTCAATATTCCCTAAGTG
ACAGTCAAAATTTTGTGTCCGTTTTTCATGATATTATGCATGGATCAGATATATCATGGC
CAATTCCTCAGAAAAAGTGGTGGTCGATTCTTCCACAACGGAATCTGATAAGTGAGAGTT
CGAGTAAGTGTTTACAGAATCTTCTTCTGTCCGAAGAAATGATTCATCGAAATAATGAGT
CACCCATTCCATTGATATGGACACATCTGAGATCACCAAATGCTTGGGAGTTCCTCTATT
CAATTCTTTTCCTTCTTCTTGTTGCTGGATATCTCGTTCGTACACATCTTCTCTTTGTTT
TCCGAGCCTCTAGTGAGTTACAGACAGAGTTAGAAAAGATCAAATCTTTGATGATTCCAT
CATACATGATTGAGTTGCGAAAACTTCTGGATAGGTATCCTACATCTGAACTGAATTCTT
TCTGGTTAAAGAATCTCTTTCTAGTTGCTCTGGAACAATTAGGAGATTCTCTGGAAGAAA
TACGGGATTCTGCTTCTGGCGGCAACATGCTATTGGGTGGTGGTCCCGCTTATGGGGTCA
AATCAATACGTTCTAAGAAGAAATATTTGAATATCAATCTCATCGATCTCATCAGTATCA
TACCAAATCCCATCAATCGAATCACTTTTTCGAGAAATACGAGACATCTAAGTCGTACAA
GTAAAGAGATCTATTCATTGATAAGAAAAAGAAAAAACGTGAACGGTGATTGGATTGATG
ATAAAATAGAATCCTGGGTCGCGAACAGTGATTCGATTGATGATGAAGAAAGAGAATTCT
TGGTTCAGTTCTCCACCTTAACGACGGAAAAAAGGATTGATCAAATTCTATTGAGTCTGA
CTCATAGTGATCGTTTATCAAAGAATGACTCTGGTTATCAAATGATTGAACAACCGGGAT
CCATTTACTTACGATACTTAGTTGACATTCATAAAAAGTATCTAATGAATTATGAGTTCA
ATAGATCCTGTTTAGCAGAAAGACGGATATTCCTTGCTCATTATCAGACAATCACTTATT
CACAAACCTCGTGTGGGGCTAATAGTTCTCATTTCCCATCTCATGGAAAACCCTTTTCGC
TCCGCTTAGCCCTATCCCCTTCTAGGGGTATTTTAGTGATAGGTTCTATAGGAACTGGAC
GATCCTATTTGGTCAAATACCTAGCGACAAACTCCTATGTTCCTTTCATTACGGTATTTC
CGAACAAGTTCCTGGATGACAAGCCTAAAGGTTATCTTATTGACGATATCGATATTGATG
ATAGTGACGATATCGATATTGATGATAGTGACGATATTGATGATGACCTTGATACGGAGC
TGCTAACTATGACGAATGTGCTAACTATGTATATGACGCCGAAAATAGACCGATTTGATA
CCACCCTTCAATTAGAATTAGCAAAAGCAATGTCCCCTTGCATAATATGGATTCCAAACA
TTCATGATCTGTATGTGAATGAGTCGAATTACTTATCCCTCGGTCTATTAGTGAACTATC
TCTCCAGAGATAGTGAAAGATGTTCCACTAGAAATATTCTTGTTATTGCTTCGACTCATA
TTCCCCAAAAAGTGGATCCCACTCTAATAGCTCCGAATAAATTAAATACATGCATTAAGA
TACGAAGGCTTCTTATTCCACAACAACGAAAGCACTTTTTCATTCTTTCATATACTAGGG
GATTTCACTTGGAAAAGAAAATGTTCCATACTAACAGTAACAGATTCGGGTCCATAACCA
TGGGTTCCAATGCACGAGATCTTGTAGCACTTACCAATGAGGCCCTATCAATTAGTATTA
CACAGAAGAAATCAATTATAGACACTAATACAATTAGATCAGCTCTTCATAGACAAACTT
GGGATTTGCGATCCCAGGTAAGATCGGTTCAGGATCATGGGATCCTTTTCTATCAGATAG
GAAGGGCTGTTGCACAAAATGTACTTCTAAGTAATTGCCCCATAGATCCTATATCTATCT
ATATGAAGAAGAAATCATGTAAGGAAGGGGATTCTTATTTGTACAAATGGTACTTCGAAC
TTGGAACGAGCATGAAGAAATTAACGATACTTCTTTATCTTTTGAGTTGTTCTGCCGGAT
CGGTCGCTCAAGATCTTTGGTCTCCACCCGGACCCGATGAAAAAAATTGGATCACTTCTT
ATGGATTCGTTGAGAATGATTCTGATCTAGTTCATGGCCTATTAGAAGTCGAAGGCGCTC
TGTTGGGATCCTCACGGACAGAAAAAGATTGCAGTCAGTTTGATAATGATCGAGTGACAT
TGCTTCTTCGGTCCGAACCAAGGAATCAGTTAGATATGATGCAAAACGGATCTTGTTCTA
TCGTTGATCAGAGATTTCTATATGAAAAATACGAATCGGAGTTTGAAGAAGGGGAAAGAG
AAGGAGCCCTCGACCCGCAACAGATAGAGGAGGATTTATTCAATCACATAGTTTGGGCTC
CTAGAATATGGCGCCCTTGTGGCAATCTATTTGATTGTATCGAAAGGACCAATGAATTGG
GATTTCCCTATTGGGCCAGGTCATTTCGGGGCAAGCGGATCATTTATCATAAAGAGGATG
AGCTTCAAGAGAATGATTCGGAGTTCTTGCAGAGTGGAACCATGCAGTACCAGACACGAG
ATAGATCTTCCAAAGAACAAGGCTTTTTTCGAATAAGCCAATTCATTTGGGACCCTGCAG
ATCCATTCTTTTTCCTATTCAAAGATCAGCCCTTTGTCTCTGTGTTTTCACGCCGAGAAT
TCTTTGCAGATGAAGAGATGTCAAAAGGGCTTATTACTTCCCAAACAAATCCTCCTACAT
CTATATATAAACGCTGGTTCATCAAGAATACGCAAGAAAAGCACTTCGAATTGTTGATTC
ATCGCCAGAGATGGCTTCGAACCAATAGTTCATTATCTAATGGATCTTTCCGTTCTAATA
CTCCATCCGAGAGTTATCAGTATTTATCAAATCTGTTCCTATCTAACGGAACGCTATTGG
ATCAAATGACAAAGGCATTGTTGAGAAAGAGATGGCTTTTCCCGGATGAAATGAAACATT
TGATTCATGTAACAGGCTAAAACGGACTATGTACTTTATCTGTTGGGTTACGGGCGGGCA
TTTTACCAGAGGTTTCTATTGTATCAATTTACCCTTGTGTGATTCCTGTTGAAGCATATA
CTCGGGGGGTGGGTGCAGGGCGGACGATTTCAAAACGGACTCCTCATTCATTAGATAGAG
AAGATCGCCAAGATTTCGTGATCCGCTGCCGAACCTATTCCAATTCCAACAGCCCGGACT
CGGATCGTGGGGATCGATGGAATACTTCGTATCAACAGATACTTGGTATATGTATATCAA
TATTGATTAGATCCGAGATCTGTTATTGAATTGCTCATTCAATGAGCATTTCAATATTAT
GCCTTGAAGAGGACTCGAACCTCCACGCTCTTTAGCACGAGATTTTGAGTCTCGCGTGTC
TACCATTTCACCATCAAGGCATCTTGAAAGTGAATCGTATTCCATGAATATGATATCTAT
CTAGTGTGATATATGGAATATATGACAAAGGTGGAGTTTTGGAGTATTTCTATCGATCGG
TCATGTCATATAGGCCCGAGTCGGACATCAAATTGCTTCGATTTGAATTATCCGGAGGAT

ACCTTATATATATCAAAAAGATGTACAATCAAACCTATTTCTCGATTCAATCGAAGCCCA
AAGAAGTTAATATGGTACCCAAATAACGATAGATATGTAAAAAGCAGGTCCGATTACGCC
TATTCCTAATCCTAAATGGAATGTAACGACGTAGGGATCCATATGTAAACATAGTCTCTA
TTTACATATGCTCGAATGACCCCTTCTCATAATGAGAATGTACATAACCCTATTCCGGTC
TGGTCCGGTATGGAATGAACTTATAATCTGATGATCGAGTCGATTCCATGATTATAAGTT
CATAACCCCAGCCCATTCCCATTTTGGGCGGAACAGATCTACTAATTCTTTTATTCCAGT
TAGTAAAAGGGATCTTGAACTAAGAAATAGACCCTAGAAGCTAAAAGAGGGTATCCTGAG
CAATTGCAATAATTGGATTCATTGATATTCCTGGTATAGTAGATGCTATCACACATACAA
TCATACTCAATTCGATGGAATTGTTTGATCTTAAGGGGGATCTTCTATAATTTCGCACGT
GAGGGGTTATTTCTTGGTTTCGTCCAGTCATTAATAACTTGATTATTTTTAGATAATAGT
AGATAGAAACAACGCTCGTAAGGAGTCCTATTGAAACCAAGAAATATAGGCCTGCCTGCC
ATCCACACCAGAATAGATGGAGTTTTCCGAAAAAACCTGCTAGTGGAGGAAGACCTCCTA
GGGATAAGAGACATAGGGCTGAAGAGAGAGCCAAAAAAGGATCTTTCGTGTATAATCCTG
CATAATCTCGAATGTTATCAGTTCCGGTACGTAGACCAAATGAGACAATGCGAGCAAAAG
TTCCTAGATTCATGGAGATATAGAACAGCATATAAGTTATCATGCTTGCATATCCATCAT
TTGAGTCTCCAACAATTATTCCAATAATTACATATCCGATTTGACCTATGGACGAATATG
CAAGCATACGTTTCATGCTTGTTTGAGTAATAGCAATGAGATTCCCCAATATCATGCTAA
GAATAGCTAGGATTTCCAGAAGAAGATGCCATTCGTTTGATGAGAAATAAAAAGGAATAT
CGAAAATTCGAGTGGCTGAAGCTGAAGCAGCTACTTTCGAAGTAACAGAAAGAAAAGCAA
CGACTGGAGTGGGAGAGTCAGAGTCGAAAAGAGGATTCCTCACTTCTTTCTCTCATTCAA
AACCGTGCATGAGACTTTCATCTCGCACGGCTCCTAAGTGATAAAAGAAAGAAGAACTCA
CCTTCTTTCTTTTTTGATTACCTTCCTCGCGTATGTATAAGACCGAATCCATTCGATTTC
TAAAAAGGATTACTAATCCTTAACTTTTCGAGGAATCCTTCATCAGTGGTTGTGAATGAC
TGACTTTTTCAATCTTTTCGACCCCGGTTCCGTAGGAGCACAAGTCAGAAAGATTGAGAA
ATAGAACCATCTGATTTGATTCGTTCTCAATAGCCATGAGATGATCATCTTAGGGTGATC
CTTTTGTCGACGGATGCTCCTATTACACTCGTAGTCTCTGAAGGATGAGAACCAACTATG
TAGCATCTACATCGATAATTCAAGTATTGTATACGTCATTAGTCCGATCCTTTGTAGGAA
CTACCCGTAATAACGAACTTGCAAAATGGATCTGTTTATCATAAAGAGATTCGTTGTTCC
TGACCCTGCTTCACCTTAATTGTTATTTGAACAAAAAGATCACAATAAACTTTTGGTAAA
AGTTATGTCTTGGTCCGAGTGGGGATAGCATTTCTCTTCTGCATGTCCATGGAGTTTTGA
AAAATCCAAACATCTCAGAGATAGATATAGAGGTAGGAATTTGTCGAACGAACCGCACTC
CTTCGTATACGTCAGGAGTCCATTGATGAGAAGGGGCTGGGGAAAGCTTGAACCCAATTC
CTACAGTGATGGATATAAGCGCAATGGAAATTCCTGGGGAGTTATACATTTGTGTATTGA
TAAGACCATTCACTATTTCTTGAAGCTCGATCTCTCCCCCGGATGAACCATATAGCCAAG
AGAAACCATGAACCAGAATAGAAGAGCTTGCCCCACCCATGAGTAAATATTTCGTAGTAG
CCTCATTAGACCGTACATCTCTCTTGGTATATCCAGATAATAGGTAGGAGCATAAACTGA
AAGATTCTGGAGCTACAAAGATAGTTATTAAATCGTTAGCACCACATAAAAACATTCCTC
CTAGAGTAGCTGTTAATACGAATAACAAAAACTCTGTTATAGCCATTTCTGTACATTCAA
TGTACTCTACGGATAGAGGAATACATAGAGTTGAACATAGTAAAATAAGAAATTGAAAGA
TTTCGTTGAAATTGTTCGTTTGGAAATTTCCCGAAAAGCTAATCATAGGTTCTTCTCTCC
ATCGGAACAATAGGGCCGTTATGCTCATTACTAAACTTGTTGAAGAGATGAAATATAACC
AAGGTATATCTTTTTGATCAGAGGTTGAATCGATCATCAGAAGAAGAATTAGGCCAAAAA
TGAGGATACATTCTGGGAAAATGAAACTTCCATGGAAGAGAAGCAAATGAAACGCTTTCA
TAAAAATTCTCGTAGAATCGAGAATGAAGTTTTCATTCTGTACATGCCAGATCCTGAATT
AGTAACTGCATCCAATCTCCGAAAAAGTCCCAATTGTTTCGAACTTTCGATTTTTGGAAT
GGGATATTTACGGAATCCCCATGAACAGGATCAAACCTTATTCCATGGTATTTCCATGAG
ATTCCTCCTTCTTATTCTTAAGAAAGCCCCCGAGAGGGCTTAGTTGATCCATGATTTATG
TTTCATCTTTCTTTTCCTTTTCGTTTGTTTCGAGAAAGATATCGATCCATTCCAATTCTT
TCTTTTTCTATTGATTCTTTTCCGATCGAGATGTATGGATCCACGGATCTATGTGTCTAT
ATAGATCCTGTTCATGGATTAACGAAAATGTGCAAAAGCTCTATTTGCCTCTGCCATTCT
ATGAGTCTCTTCCTTTTTGCGTATGGCATCGCCACTCCCTTTGGCAGCATCCACTAATTC
GGAACTTAATTTGAAAGCCATATTTCGACCCGGACGTTTTCGGGATGCCCCTAATAACCA
ACGAATGGCAAGTGCTTTTCCTTGTGTAGATTCTATTTCAATAGGAACTTGATGAGTCGA
TCCACCTACACGTCTTGCTTTGACTGCTATATCGGGAGTTACTCCACGTATTGCTTGACG
TAAAACAGATAGTGGATTTGTTTCTGTCTTTTGTTGAATCTTTTTCACGGCTTGATAGAT
AATTTGATAAGCCAATGATTTTTTTCCGTGTTTCAGAATACGGTTAACCAACATGTTAAC
TAATCGATTACGATAAATTGGATCGGATTTTGCAGTTTTTTCTTCTGCAGTACCTCGACG
TGACATGAGCGTGAAAGAGGTTCAAGAATCCGTTTTCTTTTTATACGGGCTAAAAACGAA
TCACTTATTTTGGCTTTTTGACCCCATATTGTAGGGTGGATCTCGAAAGATATGAAAGAT
CTCCCTCCAAGCCGTACATACGACTTTCATCGAATACGGCTTTCCACAGAATTTGATATG
TATCTATGAAATCGAGTATGGAATTCTGTTTACTCACTTTAAATTGAGTAGCCGTTTCCC
TCCTTTTCCTGCTAGGATTGGAAATCCTGTATTTTACATATCCATACGATCGAGTCCTTG
GGTTTCCGAAATAGTGTAAAAAGAAGTGCTTCGAATCATTGCTATTTGACTCGGACCTGT
TCTGAAAAAGTCGAGGTATTTCGAATTGTTTGTTGACACGGACAAAGTAAGGGAAAACCT
CTGAAATGATTTCAATATTGAACCTTGGACATATAATAGTTCCGAATCGAATCTCTTTAG
AAAGAAGATCTTTTGTCTCATGGTAGCCTGCTCCAGTCCCCTTACGAAACTTTCGTGATT

GGGTTAGCCATACACTTCACATGTTTCTAGCGATTCACATGGCATCATCCAATGATACAA
GTCTTGGATAAGAATCTACAACGCACTAGAACGCCCTTGTTGACGATCCTTTACTCCGAC
AGCATCTAAGGTTCCTCGAACAATGTGATATCTCACACCGGGTAAATCCTTAACCCTTCC
TCCTCTTACTAAGACTACAGAATGTTCTTGTGAATTATGGCCAATACCAGGTATATAAGC
AGTGATTTCAAATCCAGAGGTTAATCGTACTCTGGCAACTTTACGTAAGGCAGAGTTTGG
TTTTTTGGGGGTGATAGTGGAAAAGTTGACAGATAAGTCACCCTTACTGTCACTCTACAG
AACCGTACATGAGATTTTCACCTCATACGGCTCCTCGTTCAATTCTTTCGAAGTAATTGG
GTCCTTTTCCTCGTTCGAGAATCTCCTCCCTTCTTCCACTCCGTCCCGAAGAGTAACTAG
GACCAATTCAGTCACGTTCTCATGTTCCAATTGAACACTTTCCATTTTTGATTATGATCA
AAGGAGAAGATTATTCTTTTTACCAAAATATGCGGATCAAATCACGATCTTATAATAAGA
ACAAGAGATCTTTCTCGATCAATCCCTTTGCCCTCATTCTTCGAGAATCAGAAAGATCCT
TTTTGAGTTTGAATTTGTTCATTTGGAATCTTTATTTATTTTTGTATTTTTTATTTATTT
ATTTTCTTTGATTCTTTATTTCGATTTTCTTTCCCTCTCTTTTCTTTTTATTCCCTTCCA
TCATTCCTTAAGTCCCATAGGTTTGATCCTGTAGAATCTGACCCATTTTCTCATCGAACG
AGGGGTACGAAATAAATCCGATTGATTTTTCGATCAAAAGTACTATGTGAAATCTTCGGT
TTTTTCCTCTTCCTCTATCCCATAGGTACAGCGTTTGAATCAATAGAGAACCTTTTCTTC
TGTATGAATCGATATTATTACATTCCAATTCCTTCCCGATACCTCCCAAGGAAAATCCCG
AATTGGATCCCAAATTGACGGGTTGGTGTGAGCTTATCCATGCGGTTATGCACTCTTCGA
ATAGGAATCCATTTTCTGAAAGATCCTGGCTTTCGTGCTTTGGCGGGTCGTCCGAGATCC
TTTCGATGACCTATGTTGTGTTGAAGGGATATCTATATGATCCGATCGATTGCGTAAGGC
CCGCGGTAGCAACGGAGCCGGGAAAGTATACAGAAAAGACAGTTCTTTTCTATTATATTA
GTATTAGTTAGTGATCCCGGCTCGGTGAGTCCTTTCTTCCGTGATGAACTGTTGGCACCA
GTCCTACATTTTGTCTCTGTGGACCGAGGAGAAAGGGGGGCTCAGCGGGAAGAGGATTGT
ACCATGAGAGAAGCAAGGAGGTCAACCTGTTTCAAATATACAAATGGATTCTGGCAATGC
AATGTAGTTGGACCCTCATGTCGATCCGAATGAATCAGTCTTTCCACGGAGGTCAATCTT
TGCCTGCTAGGCAAGAGGATAGCAAGTTACAAATTCTGTCTCGGTAGGACATGTATTTCT
ATTACTATTAAATTCAGAAATGAAGTAGTTAATGTTGGGGTTACCATTATCCTTTTTGTA
GTGACGAATCTTGTATGTGTTCCTAAGAACCTAAGAAAAGGAATTTGTCCCTTTTTCGAG
GTCTCAAAAGGGCGTGGAAACACATAAGAACTCTTGAATGGAAATTGAAAAGAGATGTAG
CTCCAGTTCCTTCGGAAATGGTAAGATCTTTGGCGCAAGAAGAAGGGGTTGATCCGTATC
ATCTTGACTTGGTTCTGCTTCCTCTCTTTTTTTAACAATACCGAGTCGGGTTCTTCTCCT
ACCAGTATCGAATAGAACATGCTGAACAAAATCTTCTTCCTGTAAAACCTGCTCGATTTA
GATCGGGAAAATCGTACGGATTTTATGAAACCATGTGCTATGGCTCGAATCCGTAGTCAA
TCCTATTTCCGATAGGAGCAGTTGACAATTGAATCCAATTTTTCCATTCTTTTCGTATCC
GTAATAGTGCGAAAAGAAGTCCCGGCTCCGAGTTGTTCAGGAAGAGTGGCGTTGAGTTTC
TCGACCCTTTGCCTTAGGATTAGTCAGTTCTATTTCTCGATGGGGGCAGGGAAGGGATAT
AACTCAGCGGTAGAGTGTCACCTTGACGTGGTGGAAGTCATCAGTTCGAGCCTGATTATC
CCTAAACCCAACGCAATGTGAGTTTTTCTATTTTGACTTGCTCCCCCGCCGTGATCGAAT
GAGAATGGATAAGAGGCTCGTGGGATTGACGTGAGAGGGTAGGGATGGCTATATTGCTGG
GAGCGAACTCCAGGCTAATATGAAGCGCATGGATACAAGCCTTGGAATGAAAGACAATTC
CGAATCAGCTTTGTCTACGAACCGGAAGATTGGTAAGTAATGCAACTATGAATCTCATGG
AGAGTTCGATCCCGGCTCAGGATGAACGCTGGCGGCATGCCTAACACATGCAAGTCGGAC
GGGAAGTGGTGTTTCCAGTGGCGGACGGGTGAGTAACGCGTAAGAACCTGCCCTTGGGAG
GGGAACAACAACTGGAAACGGCTGCTAATACCCCGTAGGCTGAGGAGCAAAAGGAGGAAT
CTGCCCGAGGAGGGGCTTGCGTCTGATTAGCTAGTTGGTGAGGTAATAGCTTACCAAGGC
GATGATCAGTAGCTGGTCTGAGAGGATGATCAGCCACACTGGGACTGAGACACGGCCCAG
ACTCCTACGGGAGGCAGCAGTGGGGAATTTTGCGCAATGGGCGAAAGCCTGACGCAGCAA
TGCCGCGTGGAGGAAGAAGGCCCACGGGTCGTGAACTTCTTTTCCCGGAGAAGAAGCAAT
GACGGTATCTGAGGAATAAGCATCGGCTAACTCTGTGCCAGCAGCCGCGGTAAGACAGAG
GATGCAAGCGTTATCTGGAATGATTGGGCGTAAGGCGTCTGTAGGTGGCTTTTCAAGTCC
GCCGTCCAATCCCAGGGCTCAACCCTGGACAGGCGGTGGAAACTACCAAGCTGGAGTACG
GTAGGGGCAGAGGGAATTTCCGGTGGAGCGGTGAAATGCGTAGAGATCGGAAAGAACACC
AACGGCGAAAGCACCCTGCTGGGCCGACACTGACACTGAGAGACGAAAGCTAGGGGAGCG
AATGGGATTAGATACCCCAGTAGTCCTAGCCGTAAACGATGGATACTAGGCGCTGTGCGT
ATCGACCCGTGCAGTGCTGTAGCTAACGCGTTAAGTACCCCGCCTGGGGAGTACGGTCGC
AAGACTGAAACTCAAAGGAATTGACGGGGGCCCGCACAAGCGGTGGAGCATGTGGTTTAA
TTCGATGCAACGCGAAGAACCTTACCAGGGCTTGACATGCCGTGAATCCTCTTGAAAGAG
AGGGGTGCCTTCGGGAACGCGGACACAGGTGGTGCATGGCTGTCGTCAGCTCGTGCCGTA
AGGTGTTGGGTTAAGTCCCGCAACGAGCGCAACCCTCGTGTTTAGTTGCCACCATTGAGT
TTGGAACCCTGAACAGACCGCCGGTGATAAGCCGGAGGAAGGTGAGGATGACGTCAAGTC
ATCATGCCCCTTATGCCCTGGGCGACACACGTGTTACAATGGCCGGGACAAAGGGTCGCG
ATCCCGCCAGGGTGAGCTAACTCCAAAAACCCGTCCTAAGTTCGGATTGCAGGCTGCAAC
TCGCCTGCATGAAGCCGGAATCGCTAGTAATCGCCGGTCAGCCATACGGCGGTGAATACG
TTCCCGGGCCTTGTACACACCGCCCGTCACACTGTGGGAGCTGGCTATGCCCGAAGTCGT
TACCTTAACCGCAAGGAGGGGGATGCCGAAGGCGGGGCTAGTGACTGGAGTGAAGTCGTA
ACAAGGTAGCCGTACTGGAAGGTGCGGCTGGATCACCTCCTTTTCAGGGAGAGCTAAAGA

AGCGAGCTACGTCTGAGCTAAGCTTGGAGATGGAAGTCTTCTTTCGTTTCTCGACGGTGA
AGTAAGACAAGCTCATGAGCTTATTATCCTAGGTCGGAACAAGTTGATAGGATCCCCTTT
TTTACGTCCCCATGCCCCTCCCGTGTGGCGACATGGGGGCGCAAAAAGGAAAGAGAGGGA
TGGGCTTTCTCTCGCTTTTGGCATAGCGGGCCTCCCCGTGGGGGGCCCGCAAGGGCTATT
AGCTCAGTGGTAGAGCGCGCCCCTGATAATTGCGTCGTTGTGCCTGGGCTGTGAGGGCTC
TCAGCCACATGGATAGTTCAATGTGCTCATCAGCGCCTGACCCGGGGATGTGGATCATCC
AAGGCACATTAGCATGGCGTACTCCTCCTGTTCGAACCGGAGTTTGAAACCAAACTTCTC
CTCAGGAGGATAGATGGGGCGATTCAGGTGAGATCCAATGTAGATCCAACTTTCTATTCA
CTCGTGGGATCCGGGCGGTCCGGGGGGGACCACCATGGCTCCTCTCTTCTCGAGAATCCA
TACATCCCTTATCAGTGTATGGACAGCTATCTCTCGAGCACAGGTTTAGGTTCGGCCTCA
ATGGGAAAATGGAGCACCTAACAACGCATCTTCACAGACCAAGAACTACGAGATCACCCC
TTTCATTCTGGGGTGACGGAGGGATCGTACCATTCGAGCCTTTTTTTTTCATGCTTTTCC
CGCGGAGGTCTGGAGAAAGCAGCAATCAATAGGATTTCCCTAATCCTCCCTTCCCGAAAG
GAAGAACGTGAAATTCTTTTTCCTTTCCGCAGGGACCAGGAGATTGGATCTAGCCATAAG
AAGAATGCTTGGTATAAATAACTCACTTCTTGGTCTTCGACCCCCTCAGTCACTACGAAC
GCCCCCCGATCAGTGCAATGGGATGTGTCTATTTATCTATCTCTTGACTCAAAATGGGAG
CAGGTTTGAAAAAGGATCTTAGAGTGTCTAGGGTTGGGCCAGGAGGGTCTCTTAACGCCT
TCTTTTTCTTCTCATCGGAGTTATTTCACAAATACTTGCCATGGTAAGGAAGAAGGTGGG
AACAAGCACACTTGGAGAGCGCAGTACAACGGAGAGTTGTATGCTGCGTTCGGGAAGGAT
GAATCGCCCCCGAAAAAGAATCTATTGATTCTCTCCCAATTGGTTGGATCGTAGGTGCGA
TGATTTACTTCACGGGCGAGGTCTCTGGTTCAAGTCCAGGATGGCCCAGCTGCGCCAGGG
AAAAGAATCGAAGAAGCATCTGACTCCTTCATGCATGCTCCACTGGGCTCGGGGGGATAT
AGCTCAGTTGGTAGAGCTCCGCTCTTGCAATTGGGTCGTTGCGATTACGGGTTGGATGTC
TAATTGTCCAGGCGGTAATGATAGTATCTTGTACCTGAACCGGTGGCTCACTTTTTCTAA
GTAATGGGGAAGAGGACCGAAACATGCCACTGAAAGACTCTACTGAGACAAAGATGGGCT
GTCAAGAACGTAGAGGAGGTAGGATGGGCAGTTGGTCAGATCTAGTATGGATCGTACATG
GACGATAGTTGGAGTCGGCGGCTCTCCTAGGGTTCCCTCATCTGGATCCCTGGGGAAGAG
GATCAAGTTGGCCCTTGCGAACAGCTTGATGCACTATCTCCCTTCAACCCTTTGAGCGAA
ATGTGGCAAAAGGAAGGAAAATCCATGGACCGACCCCATCGTCTCCACCCCGTAGGAACT
ACGAGATCACCCCAAGGACGCCTTCGGCATCCAGGGGTCACAGACCGACCATAGACCCTG
TTCAATAAGTGGAACGCATTAGCTGTCCGCTCTCCGGTTGGGCAGTAAGGGTCGGAGAAG
GGCAATCACTCATTCTTAAAACCAGCATTCTTAAGACCAAAGAGTCGGGCGGAAAAAGGG
GAAGAGCTCCCCGTTCCTGGTTCTCGTGTAGCTGGATCCTCCGGAACCACAAGAATCCTT
AGAATGGGATTCCAACTCAGCACCCTTTGAGATTTTGAGAAGAGTTGCTCTTTGGAGAGC
ACAGTACGATGAAAGTTGTAAGCTGTGTTCGGGGGGGAGTTATTGTCTATCGTTGGCCTC
TATGATAGAATCAGTCGGGGAGGCCCGAGAGGCGGTGGTTTACCCTGTGGCGGATGTCAG
CGGTTCGAGTCCGCTTATCTCCAGCTCGTGACCTTAGCCGATGCAAAGGTATATGATAGC
ACCCAATTTTTCCGATTCGGCAGTTCGATCTATGATTTCTCATTCATGGACGTTGATAAG
ATCCTTCCATTTAGCAGCACCTTAGGATGGCATAGCCAACACATTAATGGCGAGGTTCAA
ACGAGGAAAGGCTTACGGTGGATACCTAGGCACCCAGAGAGGAGGAAGGGCGTAGTAAGC
GACGAAATGCTTCGGGAAGTTGAAAATAAGCATAGATCCGGAGATTCCCGAATAGGTCAA
CCTTTCGAACTGCTGCTGAATCCATGGGCAGGCAAGAGACAACCTGGCGAACTGAAACAT
CTTAGTAGCCAGAGGAAAAGAAAGCAAAAGCGATTTCCGTAGTAGCGGCGAGCGAAATGG
GAGCAGCCTAAACCGTGAAAACGAGGTTGTGGGAGAGCAATACAAGCGTCGTGCTGCTAG
GCGAAGCGGTGGAGTGCTGCACCCTAGATGGATAGAGTCCAGTAGCCGAAAGCATCACTA
GCTTACGCTCTGACCCGAGTAGCATGGGGCACGTGGAATCCCGTGTGAATCAGCAAGGAC
CCCCTTGCAAGGCTAAATACTCCTGGGTGACCGATAGCGAAGTAGTACCGTGAGGGAAAG
GTGAAAAGAACCCCCATCGGGGAGTGAAATAGAACATGAAACCGTGAGCTCCCAAGCAGT
GGGAGGAGAAATCTCTGACCGCGTGCCTGTTGAAGAATGAGCCGGCGACTCTTAGGCAGT
GGCTTGGTTAAGGGAACCCACCGGAGCCGTAGCGAAAGCGCGTCTTCATAGGGTAATTGT
CACTGCTTATGGACCCGAACCTGGGTGATCTATCCATGACCAGGATGAAGCTTGGGTGAA
ACTAAGTGGAGGTCCGAACCGACTGATGTTGAAGAATCAGCGGATGAGTTGTGGTTAGGG
GTGAAATGCCACTCGAACCCAGAGCTAGCTGGTTCTCCCCGAAATGCGTTGAGGCGCAGC
AGTTGACTGGACATCTAGGGGTAAAGCGCTGTTTCGGTGCGGGCCGCGAGAGCGGTACCA
AATCGAGGCAAACTCTGAATACTAGATATGACCCCAAAATAACAGGGGTCAAGTTCGGAC
AGTGAGACGATGGGGGATAAGCTTCATCGTCGAGAGGGAAACAGCCCGGATCACCAGCTA
AGGCCCCTAAATGACCGCTCAGTAATAAAGGAGGTAGGGGTGCAGAGACAGCCAGGAGGT
TTGCCTAGAAGCAGCCACCCTTGAAAGAGTGCGTAATAGCTCACTGATCGAGTGCTCTTG
CGCCGAAGATGAACGGGGCTAAGCGATCTGCCGAAGCTGTGGGATGTAAAAATGCATCGG
TAGGGGAGCGTTCCGCTTAGAGGGAAGCTCCCGCGCGAGCAGGTGTGGACGAAGCGGAAG
CGAGAATGTCGGCTTGAGTAACGCAAACATTGGTGAGAATCCAATGCCCCGAAAACCTAA
GGGTTCCTCCGCAAGGTTCGTCCACGGAGGGTGAGTCAGGGCCTAAGATCAGGCCGAAAG
GCGTAGTCGATGGACAACAGGTGAATATTCCTGTACTACCCCTTGTTGGTCCCGAGGTAC
GGAGGAGGCTAGGTTAGCCGAAAGATGGTTATCGGTTCAAGGACGCAAGGTCACCTTGCT
TTTTTAGGGCAGGGTAAGAAGGGGTAGAGGAAATGCCCCGAGCCAATGTCCGAGTACCAG
GCGCTACGGCGCTGAAGTAACTCATGCCATACTCCCAGGAAAAGCTCGAAGGACCTTCAA

CAAAAGGGTACCTGTACCCGAAACCGACACAGGTGGGTAGGTAGAGAATACCTAGGGGCA
CGAGACAACTCTCTCTAAGGAACTCGGCAAAATAGCCCCGTAACTTCGGGAGAAGGGGTG
CCTCCTCACAAAGGAGGTCGCAGTGACCAGGCCCGGGCGACTGTTTACCAAAAACACAGG
TCTCCGCAAAGTCGTAAGACCATGTATGGGGGCTGACGCCTGCCCAGTGCCGGAAGGTTA
AGGAAGTTGGTGACCTGATGACAGGGGAGCCGGCGACCGAAGCCCCGGTGAACGGCGGCC
GTAACTATAACGGTCCTAAGGTAGCGAAATTCCTTGTCGGGTAAGTTCCGACCCGCACGA
AAGGCGTAACGATCTGGGCACTGTCTCGGAGAGAGGCTCGGTGAAATAGACATGTCTGTG
AAGATGCGGACTACCTGCACCTGGACAGAAAGACCCTATGAAGCTTTACTGTTCCCTGGG
ATTGGCTTTGGGCCTTTCCTGCGCAGCTTAGGTGGAGGGCGAAGAAGGCCTCCTTCCGGG
GGGGCCCGAGCCATCAGTGAGATACCACTCTGGAAGAGCTAGAATTCTAACCTTGTGTCA
GGACCTACGGGCCAAGGGACAGTCTCAGGTAGACAGTTTCTATGGGGCGTAGGCCTCCCA
AAAGGTAACGGAGGCGTGCAAAAGTTTCCTCGGGCCGGACGGAGATTGGCCCTCGAGTGC
AAAGGCAGAAGGGAGCTTGACTGCAAGACCCGCCCGTCGAGCAGGGACGAAAGTCGGCCT
TAGTGATCCGACGGTGTCGAGTGGAAGGGCCGTCGCTCAACGGATAAAAGTTACTCTAGG
GATAACAGGCTGATCTTCCCCAAGAGTTCACATCGACGGGAAGGTTTGGCACCTCGATGT
CGGCTCATCGCATCCTGGGGCTGTAGTAGGTCCCAAGGGTTGGGCTGTTCGCCCATTAAA
GCGGTACGTGAGCTGGGTTCAGAACGTCGTGAGACAGTTCGGTCCATATCCGGTGTGGGC
GTTAGGGCATTGAGAGGACCTTTCCCTAGTACGAGAGGACCGGGAAGGACGCACCTCTGG
TGTACCAGTTATCGTGCCCACGGTAAACGCTGGGTAGCCAAGTGCGGAGCGGATAACTGC
TGAAAGCATCTAAGTAGTAAGCCCACCCCAAGATGAGTGCTCTCCTATTCCGACTTCCCC
AGAGCCTCCGGTAGCACAGCCGAGACAGCGACGGGTTCTCTGCCCCTGCGGGGATGGAGC
GACAGAAGTCTTGAGAATCCAAGATAAGGTCACGGCGAGACGAGCCGTTTATCATTACGA
TAGGTGTCAAGCGGAAGTGCAGTGATGTATGCAGCTGAGGCATCCTAACAGACCGAGAGA
TTTGAACCTTGTTCCTACATGACCCGATCAATTCGATCAGGCACTCGCCATCTATTTTCA
TTGTTCAACTGTTTGACAACATGAAAAAACCAAAAGCTCTGCCCTCCCTCTCTATCTATC
CAAGGGATGGAAGGGCAGAGGCCTTTGGTGTCCCTTCCAGTCAAGAATTTGGGCTTCACA
ATCACTAGCCAATATTTCTCTCATACCTTTCTTCGTTCATGGTTCGATATTCTGGTGTCC
TAGGCGTAGAGGAACCACACCAATCCATCCCGGAACTTGGTGGTTAAACTCTACTGCGGT
GACGATACTGTAGGGGAGGTCCTGCGGAAAAATAGCTCGACGCCAGGATGATAAAAAGCT
TAACACCTCTTATTCTTATTACTCAAAAAGAAAAAAATGAAAAGGTCGTCTTATTCAAAA
CCCAATTATGACATCCCTTCTCTCCCACTTCACACCTCGGAACGCGCTGTTCTTATAGAG
AGAAAGGCGCTTTCACGTCTTCTTAACCCGAAATGGCTGAGGGGAGAAAAGGTTCCTTTT
TGAGGGTACTCCAGGGAACAGATCCAGTGGAGACGGGGTGGGGCCTGTAGCTCAGAGGAT
TAGAGCACGTGGCTACGAACCACGGTGTCGGGGGTTCGAATCCCTCCTCGCCCACAACCG
GCCCAAAAGGGAAGGACCTTTCCTTTACCTCTGGGGGTAGGAAAATCATGATCGGGATAG
CGGACGCAAAGCTATGGAACTGGGGTGTGGGTCTTTTGTCGAAATGGCCTTATTCTTTTT
ATTTATCGTGAAGGAAAAAATCGATACATATAGTATGCCTGGCCCGAATCAGCATATTTG
TGTTTTACTCCCCGTAACTCTTCCTCAGCCAGGCTTGGGAAGAATAGCAGAGCAAATACA
AGTATTAGTAGCATAGCAAAAATGCGTTCCTCGTCATTAATATGTTTGCTCGCGGTAATT
GTGGCCTCTCGGGAGAATCGATGACTGCATCTTTGATGCACTGCTAGTACATCATCTGAG
AATTATGAATTGGCTAGTTGTAAATAGCCCCAGGGCTATGGAACAAAGGATTATCCCGGA
TCTACACCGAGGTATTGACGGTGATTCTCAAATATCGCAGAACAGAATGCGATGAGATAG
AGTGCAATAGAAACAAAGACAGGGAACGGGTTACCTACTCCTAACGGTCAAAGCGAGCCC
TTTAATTCTGTAATTCTGAATTCTTTAATTAAGAATTCATCAAATCTCCCCAAGTAGGAT
TCGAACCTACGACCAGTCAGTTAACAGCCAACCGCTCTACCACTGAGCTACTGAGGAATA
ACGGGAGATTCGATCTCATAGAGTTCAACTCCCGCTCTCAACCCATGACCAATATGAGCC
CGAAGCTTCCTTCGTAACTCCCGGAACTTCTTCGTAGTGGCTCCGTTCCATGCCTCATTT
CATAGGGAACCTCAAAGTGGCTCTATTTCATTATATTCCATCCATATCCCAATATTCCAT
CCATATCCCAATTCCATTCATTTAATATCCCTTTGGTGTCATTGACATAAGAGATGTCAT
TCAATCGAAGAAGAGGGTATCATTGACATAAAAGATGTCATTTCTAGTCTATCTGTTTCT
ATCTATGGAAAGTGAAGAAATCATCATATAGTAATCGAGAAATTGCAATAGAAAAGAAAA
AAGGGAGGTTTGTGATGATTTTGAAATCTTTTCTACTAGGTAATCTATTATCCTTATGCA
TGAAGATAATAAATTCGGTCGTTGTGGTCGGACTCTATTATGGATTTCTGACCACATTCT
CCATAGGGCCCTCTTATCTTTTCCTTCTCCGAGCTAGGATTATGGAAGAAGGAACCGAGA
AGGAGGTATCAGCAACAACTGGTTTTATTACGGGACAGCTCATGATGTTCATATCGATCT
ATTATGCGCCTCTGCATCTAGCATTGGGTAGACCTCATACAATAACTGTCCTAGTTCTAC
CGTATCTTTTGTTTCATTTCTTCTGGAACAATCACAAACACTTTTTGGATTATGGATCTA
CTACCAGAAATTCAATGCGTAATCTCAGCATTCAATGTGTATTCCTGAATAATCTCATTT
TTCAATTATTCAACCATTTCATTTTACCAAGTTCAACGTTAGTCAGATTAGTCAACATTT
ATATGTTTCGATGCAACAACAAGATGTTATTTGTAACAAGTAGTTTTGTTGGTTGGTTAA
TTGGTCACATTTTCTTCATGAAATGGGTTGGATTGGTATTATTCTGGATACGGCAAAATC
ATTCTATTAGATCGAATGTACTTATTCGATCTAATAAGTACCTTGTGTCAGAATTGAGAA
ATTCTATGGCTCGAATCTTTACTATTCTCTTATTTATCACCTGTGTCTACTATTTAGGCA
GAATACCGTCGCCTATTGTCACTAAGAAACTGAAAGAAACCTCAAAAACGGAAGAAAGGG
GGGAAAGTGAGGAAGAAACAGATGTAGAAATAGAAAAAACTTCCGAAACGAAGGGGACTA
AACAGGAACAAGAGGGATCCACCGAAGAAGACCCTTCCCTTTGTTCGGAAGAAAGGGAGG

ATCCAAAAAAACTACATGAAAAAAAAAAAGAGGCAAGAAATTTTGAAGTTAGAAATACTTA
AAGAGAAAGAAGATAAAGACCTCTTCTGGTTTGAAAAACCTCTTGTGAATCTTCTTTTCG
ACTATAAACGATGTAATCGTCCATTGAGATATATAAAAAAAAATTTATTTCAAAATGCTG
TAAGAAATGAAATGTCACAATATTTTTTTCACGTATGTCCAGTTGATGGAAAACAAATAA
TATCTTTTACATATCCACCCAGTTTATCGATTTTTTTGGAAATGATGCAAAGAAAGATGT
CTTTGTGTACGACCGAAAAACTATCCCCCGAAGATCTGTATAATCATTGGGTTTATACCA
ATGAACAAAAAAGGTACAGCTTGAGCAATGAATTCATAAACCGAATAGAAGTTCTAAACA
AGGGATCTCTTACTATGGATGTGCTTGAAAAAAGGACCAGATTGTATAATGATAAAAATA
ACCAAGTCTAGTCACTTCTTATTCTATTCAAAACAAAAAGAATAGATAATAGATATAGTA
AATTAATAAAAAGCCGGATACATATGATAAATATACAAAGAGATAAGAAGAGATTCGCCC
TCCCCCCACATATTTGATCCCTTCTCCTATAAAGAAACTTGCAACACCAACACCATTGGT
AATTCCGTCAATTACCCGTCTATCTAAAAAATGAGTTACTTCAGCTAATCCTCTTATACT
TGTAGTTAAGCATCTTGCATAAAAAAGATCTATGTAACCACGATTATATGACCAATTGTA
TATCACATTTATTATTCGGTCCAATAAAATTTTCTTAGAGCCTGTTTTTTTAACAAATGA
ATTGATTAAGTCCAAATTCTGAAAAGATGAATTAACAGACCCATATAAAATAGACGCTAT
GAATATTCCAAAACAGGCTATACTGACTGAATAAATTGCATTTGTCACAAATTCATACCA
ATCCACAGAATAGTTCGAATTTTGATGTAAAAGGTTTATTGATGGAGTTAACCATTTTGA
TAATATATCAAAATCGATTACTCCTTGATCGAAAGGAATTCCTATGGATCCAACGAACAA
AGTAAATAGGACCAATATAAGTAGAGGCAAAAGCATAGTATTGTCTGATTCATGGGGATA
CGTTGAAGTGTCTTTATTTTCAAAATAAATCCTACAGGAGCGTATCAGATTTCTTACATT
TCCGTTCATTTTGTATATCTTCTTCGAAAAAAAGGAAACCTTTTCATTATTATTCATTGT
TGATAAAAACAAATTTCTGTTAACTGGTTTGGTTCCTTCTTTCCCCCATATAGATATTGA
ATAGAACGAGCTATTTTGAGTGCCACTGTAATTTTGAAAATGAGCATGTAAATGACCATC
AAAAGTAAGTAAATACATCCGAAACATATAAAATGCAGTTAACCCCGCCGAGAAACAAGC
TATTATCGCGAAAATAGGTGAATACAACCAACTATCATTAAGAATTTCATCTTTAGACCA
AAAACAAGCAAGAGGTGGAATTCCACAAAGAGAAAGTGTACCTAATAAAAAAGTATTTTT
TGTAATTGGCACATATTTTGTTAAACCACCCATAAGAACCATGTTCTGACTTTTATCTGG
AGAATATCCAACAATGGGTTCCATTGAATGAATAATTGATCCGGATCCTAAAAACAATAA
TGCTTTCGAATAGGCATGAGTGATCAAATGGAATAAAGCAGCTCGATAAGAGCCTATCCC
TGGGGCTAACATAATATAACCCAATTGAGACATTGTAGAATAGGCTAAACTTCTCTTAAT
GTCTCTTTGAGCAAGAGCTAAAGTAGCTCCTAATAGTACCGTTATTACACCTATCAAAGA
AATGAGATTCATTATGTAAGGTATGACTGTGAAAAGCGGAAGAAATCGAGCGACAAGAAA
AATGCCTGCTGCTACCATAGTAGCAGCATGGATAAGAGCTGAAATAGGAGTAGGCCCCTC
CATGGCATCAGGTAACCATACATGAAGGGGAAATTGTGCGGATTTAGCAACTGCACCGAC
GAATAATAGGGAGGCACACAGAGTAGCAAATAAAGAGTTGACCCCATTATTACGGATCAG
GTTATTGAAGATTTCGAACAAATCTCGAAATTCGAAACTCCCTGTTATCCAATAAAAACC
TAAGATTCCTAATAATAACCCAAAATCCCCTACACGATTAGTTACAAACGCTTTTTGACA
AGCATTTGCGGCAGCCGGTCGTGTGAACCAAAAACCTATTAATAAATACGAACACATTCC
CACTAGTTCCCAAAAAATATGAATTTGTATCAAATTGGAACTAGTAACTAATCCCAACAT
GGAAGTATTGGAAAAACTCATATAAGCAAAAAATCTCAAATATCCTTGATCATGAGACAT
ATAATTGTCACTATAAATAAGAACCATGATTCCAACCGTAGTGATTAGTATTGACATAAT
AGAAGTAAGTGGATCGATCAAGTAGCCGAACTCTAAGGAAAAATCGGTATTGATGGTCCA
AGACCATAGATGTTGATAGATAGAACTGCCATTTATCTGTTGAATAGACAGATCGGACGA
AAAAACCATAACTATACTTAGCAATGAAACACTAGGAAAAGTCCACATACGACGCAGATT
TTTTGTTGCGGTCGGAACAAGCAGAAGTCCCAACCCTATTGACATAGTAACTGGAAGTAG
AGCGAAAGGTATGATCCATGCATATTGATATGTATGTTCCATAAGAAAATAAAACTTTCT
TTTTTTATTCTTATTAATTGTTTCCCATTCACCAGCCCTTATTTCTTCCGGAAGGAGCAA
TAATCAAATAAATAAAAAAAAAATCAAGATATACAAGATATAAAAGAACTCAAATATGAT
TTTTCATTCTTAATTATTCTGATTCTTTCCAAACTATTGAAAAAAAAAAACAAAAA
ATTCAAATAATCAAGTTACTAGTTAAAAGCTACTACCTAGTTATTAACGAAGATATTTAT
TAAGATAAAAAATACGAGATTTTCAATTATTCTACTATATACATACGATACGGTGATTTC
TATCCATATTTATATCAATATAGTAAGGAAAAAGAATGATACCCAAATTTCAAGTACTTT
ATTCTGATATGTATCGTAGGATCTGCCAATAACTCGATCCAATAAACCTAGTTTTTTTTT
AGTTTCTTCGGAGTCATTAACTAAAACAAATTCTGGAATTGATTGGCTTCTAGTCCAATA
AAACAAACTTATGTTTATGTGTTTATGAAAAATGCTAGAATACTTGTCACTTCGCATAGA
ACTAAAAAGAGAAATTACTCAAATTCCCTTTATTTTATCAAGTAATGTATTGTTTAACGG
ATTAACTACTTTAGATTTAATTTCAATTTAAATTATGTGGACTCTATCTCCGAGCTTGAT
CAATTAATAGAAAAAGGTTACATTCATTATTGGTTTCCTAAATTAGGAAAGGGTTCTTAA
GCTTTTCGATTTATTAAAAATAACATTTATAATATATAGATATATTATCTAAATAGACTG
AGATATGAATTGGACCCTTTTTAATTCTTATCAATGGCATCCGATTCAACGGTAGTAGAT
CCCGCCCGTAGACATAGATTGAATAAAGATATGAAGCCCCCAATCAGTACAAATTATTCT
TTCTTCGAACATTGGATGTAATGGAAATGTGAAAAAAAAAAAAAAATTCCCTTGAAAATCA
CATCGTTTTTTCTTTTTTCTTCTATTTCATTTCTTTTTTTATCCTTAATGATACCATATG
CGATGCTGATCTATCTGTATCCATACTCTTCTATGTTATGAAGTAAGCATAAGTATCGGC
TATGGAATAAAGATAGATAATGAATAGTTAATAGAAAGAACAATCTATTTTGAATTGAAC
AATAAATGTCTTTCACGTCCAACTATAAAAATGAGTGACCCTTTTATTTTGAATGGCGG

TTCCAAAGAAACGTACTTCTCTGTCAAAAAAGCATATTCGTAGAAATATTTGGAAAGGAA
GGGGATATCAGGCCGCGGCAAAAGCTTTATCTTTAGCTAAATCTATTTCTACCGGGCATT
CAAAAAGTTTTTTTGTGCGACAAACAAGTAATAAAGCCTTGGAATGATCTGAATCTGCAT
CAGCATGACTCGATGAATTGGATGATTAAATTTATAAGATGAAGAATTCACATTAACTAA
TGTTTTGAACTCATCAATATGAGATAGAACTAGCTGTTGTGGTATGTAGAATATGAAGTA
TTCTGTATACTAGGTTCTAAAAATGATTTCTTTTTTTGTTTGAAAAAAAAAAAAAAGAAA
GAAAAAGAAGTAGTTAGACACAAAATACAAGGTTTCACCTTTCATTGATTGGTTTTTATA
ATTCGCGAGATGGGGATTGTAACTTTCCCCATCGATTCATTTTTCACAATAACAAAACTC
TTACTTTTTTTCTTAGGAAGGGATTGCCTTATTGGATTATGTATCTCCAATAGTTATACA
TCATTAAGATTTTTGGAAAATCTGAAACAAGTATGAACGAGGTTAGAGCCCCCTTTTTTG
TTCCAAAGTAAGGCGGGGGTAAGATTCATAGTCAAAGTCAATGGATTATTGAAACTAATT
GATTAAAATATACATTTTAAAACTTTGATTTGTAGCTCTCTGAATCACTACATATCTACA
AGGACTCCCTCTTGTTTCGAACAGATAAAAAAAAAAAACACAAAATAATAAAACTGCCAG
GATCCCATTTAGATCGATATTTATGTACTAAATAATGAGTCAATCTTACGTTACGTAATC
CAACCCCAATGGATCCTATCCCATGTTTGAGCTGGAGGATCGATCAATCGATATATCTAG
ATCCAATATCTAAATTATTTTATCTATTAATATTAGATTTCAAATCTATATATAAAGAGA
TCTTATCAGTTTAAATTTGATTTTCTAAGTTTTCTAAGTTAAAGTACATACAGTAGAATT
CCGATAAAGATTGAAACTCTTTTGTTATACGATATGCCCGGTCCAATACCTAATGGGTTT
GGTAAATCCTCACACAAATTATGTTCTGTATACAATGAAGATCCCAATCATTAATACATC
TTTGATACCAAACTATCCAAATTTTCATAGAAATCCTTTGGATGTAGTGATGAAGTTGTG
ATATATAAAAAATCTTGTTTTATTTTGTTCATTGAAAAATGAATCTTTTTCATTTCGGAT
CTATCAAATCAGATAAATGAGAAATGAATCGTCAAAAAATGAGAAAAAAAATTCTTAGTTC
TATACCTGGACTCAGGGCATAACCGTCTAGTTCCAACTATTCCAGAAGAACTTATAATAC
GGAAAAGCCCGCTGTTTAAAAGGACCCCCTGCCACGATACTAAGGATTATTGAGCGTTTA
AATATTTATTTGAACGGGTCTTTATTCATCGATTCTAACATTTCCTAAAATAGATTGCAT
TAAATCCCTCAATCTCGACGATTGAACATCATATGGACTATGATTATTTCGATGATGCCG
CCATGGTGAAATTGGTAGACACGCTGCTCTTAGGAAGCAGTGCTAGAGCATCTCGGTTCG
AGTCCGAGTGGCGGCATCCTCTAAAAAAGGCACAATAGATCCTATAATGAATTCAATTCC
GGATTTCCATTCCGTAATTGGGGATACCCCCCTAAAAAAAATTATGATATTTGCCACTTT
AGAACATATATTAACTCACATCTCTTTTTCTATCATTTCAATTGTTATTACGATTCATTT
GATGACCTTATTAATCCATGAAACCGTAGTACTATTTGATTTGTCAGAAAAAGCCATGAT
GGCTACCTTTTTCTGTATAACAGGATTATTAGTTACTCGTTGGATTTATTCGAGACATTT
GCCGTTAAGCGATTTATATGAATCTTTACTGTTTCTTTCATGGAGTTTCTCCATTATTCA
TATGTTTCCTAAAAGACGGAACCAGAAAAGTTATTTAAGCGCAATAACCGCGCCAAGTGC
TATTTTTACCCAAGGCTTTGCCACTTCGGGTCTTTCAACCGAAATGCATCAATCTGCAAT
ATTAGTACCTGCTCTACAATCCCAGTGGTTAATGATGCACGTAAGTATGATGTTATTGAG
TTATGCAGCTCTTTTATGTGGATCGTTATTATCCATAGCTCTTTTAGTCATTACATTTCG
AAAAAACCTAGATATTCCTCGCAAAAGCAATCATTTATTAAATGGGTCATTTTCCTTTGT
GAATGAAAAAAGAAGCGTTTTACAAAACACTTCTTTTCTTTCATTTAGAAATTATCACAG
GTATCAATTAACTCAACAATTAGATCAGTGTAGTTATCGTGTCATTAGTCTAGGGTTTAC
TTTTTTAACCATAGGTATTCTTTCGGGAGCAGTATGGGCTAATGAGGCATGGGGGTCTTA
TTGGAATTGGGACCCCAAGGAAACTTGGGCATTTATTACTTGGACCATATTCGCGATTTA
TTTACACAGTAGAACAAATCAGAGCTTTCAGGGTGTGGATTCGGCAATTGTGGCTTCTAT
AGGATTTCTTATAATTTGGATATGCTATTTTGGGGTCAATCTATTAGGAATAGGACTACA
TAGTTATGGTTCATTCACATTAACAACTAATTGAAGAAAGGGCCTGAAGAATACATAGGA
ACATCGTCCCGTATATTATATAAAGAAGGTTTATGCAAGTTTTTTCGAACCATTTGAATC
AAGTAGTGATTCAAATGGTTCGAAAAAACTTTAGATGTATCTGATTATAATTCACTTCAT
TTTTTTTTTCTTTCATTGAACGCTTTTAAAAATCACACAGTTCTTTTACATTAATGTAAT
GTCTTATTGATGAAAACTATTTATGAAAATAAATAGATAGAATAGCTTCTATCCTGTCAA
TTGATAGCGAGAGAACGAAATCAGGATAAATACCAATACCTATTACAGGTAGAAGGATAC
AGACCGAAACAAATAGTTCTCGTGGTCCAGAATCAAAAAAATAAGAGTTTGGAACGTTGA
ATAGCTTGTAGCCATAGAACATCCGACGTGACATAGATAATGAATAAATAGGAGTTAATA
TCATTCCAATTGCCATTACGAAAGTTATTAGTATTTTTGGCATTAAAAGATATTTTGGGC
TAGTAATTATTCCAAAAAATACTACTGATTCCGCAACAAAACCACTCATTCCTGGCAATG
CAAGAGAAGCCATCGAGAAGCTACTGAACATGGTAAATATTTTTGGCATTGGGATAGCTA
TTCCTCCCATTTCGTCGAGATAAACAAGACGTATTCTATCGTAGCTCGTTCCTGCCAAGA
AAAAAAGTGCAGCACCAATAAATCCATGAGAGATTATTTGTAAAATGGCTCCATTGATTC
CCGTATCGGTTATGGAACCAATTCCTATAAGTGTGAAACCCATATGAGATACGGAAGAAT
AGGCTATTCTCTTTTTTAAATTGCGTTGACCGAAAGAAGTTGAAGCTGCATAGATTATTT
GAATCGCTCCTACTATCATCAACCAGGGAGAAAATATAGAATGAGCATGGGGTAATAATT
CCATATTGATCCGAACCAATCCATACGCTCCCATTTTTAATAAGATTCCCGCTAGAAGCA
TACATGTACTGTAATGTGCTTCTCCATGGGTATCTGGTAACCATGTATGTAGGGGTATAA
TCGGCGATTTGACAGCATAAGCAATAAGGAAGCCAATATAGAATATTATTTCCAATCCCA
AAGGATACGATTGATTAGCTAATGTTTCAAAATTTAATGTTGGCTCATTGGAGCCATATA
AACCCATACCCGGAACTCCCATTAAGAGAAAAATAGAACCCCCCGCTGTGTACAAAATAA
ACTTTGTAGCTGAGTACAGACGTTTCTTCCCTCCCCATATGGATACAAGTAGGTAAACAG

GAATTAATTCTAATTCCCACATGAGGAAAAAAAGTAAAAGGTCTCGAGAGGAAAATGATC
CTATTTGACCACTATACATTGCTAACATCAGGAAATGGAACAATCGGGAATCTCTAGTAA
CTGGCCGAGCCGCTAAAGTAGCTAAAGTAGTGATGAATCCCGTCAGTAAAATGGGTCCTA
TGGAAAGTCCATCGATTCCCGGTCTCCAGTGAAAATCAAAAGTATTTATCCATTTATAAG
CCTCTTCTAATTGGATTAATGGATCGTCCAATTGGAAATGATAACAGAACGCATAGGTCG
TTAGAAGGAGTTCTAATAAGCATATACAAATAGTATACCACCGAACCACCTTATTTTTAT
TCCCTCTACGAGGGAGAAAGAAAATTGACGAACCCGCGGATATCGGCAAAACAACAATTA
TTGTTAACCAAGGAAAATAACTCGTGGTAAAGACAAGATAGACTTTGACCAGAAAAACCC
GCGCTCGGAATAATTTCTCGAGTACGGGTTTTTGTCGGTAAAGAGGAATCAAATGGATTC
AAGTGGATTTTTCTAGAACGTATCAATAAGCTAGACCCATGCTGCGAGTTGTCTCATGCC
ATAAGTAAACCCGAACACTCAAGAAATCCGTTGGACAAGCGGATTCACATCTCTTACAAC
CTACACAGTCCTCTGTTCTTGGAGCAGAAGCAATTTGTTTAGCTTTACATCCGTCCCAAG
GTATCATTTCCAATACATCTGTGGGGCAGGCTCGTACACATTGAGTACACCCTATACATG
TATCATAAATCTTTACTGAATGCGACATTGGATCTATAAATTTTTTGAACTTTATGAATT
TTCGATCTGGCTTCATTAGTAATTGAATTATATATATTATGTTGTAGACGCCAGACGAAT
CAGTGGTTTACCAGAACTTTTTTGATAATCAATCTATTTCTGGATCTGTTCATGAGCAAG
GGCCAAGATACTTTGATTTCTTACGTTTCAACAAGCATGGTCATACGAATCATACATGCT
AGTTCTAAATTAGTGAATATTTTGTGGATATCTGTCTTTATTTATATCATTAATACTATT
TATTCAACAAATTCGATTGATTGATACGAGTTGATTTTCTGTTACGATGGATCGATGAAA
CAATAGCCGGCCCAATAGCTGCTTCAGCGGCTGCAATAGCTATAACAAAAATCGAGAAAA
TATCTCCTTTTAATTGACGACTATCAAACAAATCAGAAAATGTTACGAGATTTATATTAA
CCGCATTCAGTATAAGTTCAAGACACATAAGTGCTCTAACCATGTTTCGACTTGTGATCA
ATCCATAAATACCGATAGAAAATAAATAGGCACTCAAAATAAGTACATGTTCGGTCATCA
TTGACCAACCCCTCATCAATTTTGATTCATTTCAATATGAACAACAATTTCACCGATTTT
ATTAGAATATAAATGAAGTACGAAACAAAGTAAGGTATTAGTAATGGATCGAACTAAACC
CCTTTCAATTTCATATATGAACAATAGAATATGAAAATGGAACTGAAGGAAAATGGATGT
GATAACATAGAACAAAACAAGACTTTATTTATTTTATTTGATTTTGGATCTAAGTATTTA
TTACTTAATTACTTTACTGCCGGGCCATAGCAATTGCACCTATCAAAGCAACTAAAAGAA
TTATAGAAATGAGTTCAAATGGAAGGTAAAAATCTGTTGATAAATGAATCCCAATTTGTT
GAACGTTACTTGTTAGGTCCTGTTCTATAATCTGATTTGATCTTGTAGTCCAAACAATCC
CGTACCACGACGTATCCGAGATAGTAGTAATTAGTGAAAAAAGAATACTTGTACAAACCA
GTGAAGTGACCCCATCCCCAACGGTCCAAAGATAGAAATCTTTGTAATATTCTGAACCAT
TCATGAACATCACAGCAAATAGGATTAAAACATTTACAGCTCCTACGTAAATAAGGAGCT
GTGCAGCAGCTACAAAATAGGAGTTAGATGGAATATGGAATAAGGATATACAAACAAGAA
CCAGTCCCAATGAAAAAGCAGAATAAATTGGGTTGGTAAGTAAGACCACCCCCAGACCCC
CTAATATAAGACCTGATCCCAGAAATACTAAAAGAATATCATGTATTGGTCCAGGTAAAT
CCATTATGTGTAAAAAAAGAGATAAATAAATCGAAATATTTCATAAACTTACTAACCGAC
CCAAGAAAAAAGAGGTTACCTTATTTTTTTCTTGTATATGATACGTTCCTAATTGAATCC
TAAAGGGGTGTAGATTTATATAGATACAGTTATTGGATCAATCCCGCTACTGTATCTGAA
AGAGTAGTTCGAAATTGTATATTTTGAAATCATCACAGATCTATGAATCCATTCTAAATC
AAAATCAAGCCTTGATTCTCACCAATCAATAGTTATTTACTGACCTAGCAAAATGAAAGA
AGCCTCACTCCTTTACTTTAAATCAAAACGGAATCTTAGTAATTGGTAATCGTTTTTGAA
TCAAGAGGTTTACCCCTGATTATTTTGATTGGAGTCGAATTCGTAATTGTTCGAATTGTG
TAATCTCCAATTACTGACATTGGTAACCGGCCCAAAGCAATTTGATTATAATTCAATTCG
TGACGATCATAAGTAGAAAGTTCATATTCTTCAGTCATTGATAAACAGTTTGTTGGACAA
TACTCGACACAATTACCACAAAATATACAGATTCCAAAATCAATACTATAATTAAGCAAT
CGTTTCTTTCTAATATCCGTTTCCAATCTCCAATGAACAACGGGTAGATCTATGGGGCAT
ACCCGAACACATACTTCACAAGCAATGCATTTATCAAATTCAAAATGGATTCGACCACGA
AAACGCTCCGATGTGATCGACTTTTCATAAGGATATTGAATAGTCACAGGTAAACGATTC
ACGTGAGATAAGGTAATCATGAAACTTTGACCAATATACCTTGCAGCTCGTATTGTCTGT
TGACCATAATTCATGAACCCAGTCACCATAGGGAACATATCGTGGATATCCATGAATAGT
TTGATGTTTCTTTCTCTTGCTCTAGATAGGTTATGAATCTAGAATATCATATTTTGTTAT
AGCGAAACGAGTTGGGAAGAAGTTGTTAATAATAGATTACCTAGAGAAATAGGTAAAAGA
AATTTCCACCCAAGGTTTAATAGCTGGTCCATTCTCATCCTAGGTAAAGTCCATCTTGTT
GTGATAGGAATGAACAGGAACAAATAAGCTTTAGCTAATGTAATAAGGATACCAATTGTC
ATTCCAAAGACTCCACCTGTTTTATTTATTCCAAAAGGTTCAGAAATGAATATGTACGGA
ATAGAGAAATTCCACCCGCCCAAGTAAAGAACTGTTACAAATAATGAAGAAACTAGTAGA
TTTAGGTAAGAAGCAACGTAAAATAAACCAGATTTAATACCTGAATATTCGGTTTGATAA
CCTGCTACTAATTCCTCCTCTGCTTCTGGTAAATCAAAAGGTAATCTTTCACATTCCGCT
AGGGAAGAAATTAGAAAAACTATAAACCCTATAGGTTGACGCCACAGATTCCACCCCCAA
AACCCATATTTTGACTGTGCCTCAACTATATCAACTGTACTTGAACTGTTAGATAATCAT
AGTCGATGATAACATCACTATTCCCATCGCTATTCCAGAACCGCACATGAGACCTCAGCT
TCATACGGCTCCTCGATGGCCACAAATAAATCTAAGGACTGGTTCATTATTACCTCGGCA
TGTTTGTAGTGTAGATTAGAGTAAAGATGTATCGAAGAGTCCCGAATTAGACCAATGGAA
TTCCGTCCGCCATAATAAAAAAAAAAAAAAAGCGCTTCCGAATTGATCTCATCCTTTA
TTTTCTAATTAGACTTAGAATTCTTTTAGTTCAGTAATAACTTAATCCTTCAATAAAATA

CTCGTTGTTTCAATAGATATTTCGACCCATACTTTTCGTACGAAAAAGAATTAGACACTA
ATTCATGAGTTATAGTTGATAAATCATTATAAGAATTCTATCCGAATAAATATGGATAGG
ATTGAGGAAAGAAAGAATAAACAAAGATCTCTTATTATTCAGTTTTCCTATTGCATTCCA
TTTCTTTCGTTCCTGTTCTTCTTTCTCACTCAGAAGGGGGTTTCTAAAAAATCAAATCAA
AGGATTACTTCGTTCTCGACAGTCATTTATTTAATCAGTGGATAGAAGCATACTCTGGAT
CGGAATCGTGAGGAAGTACTGCTTGATCATTTCTACGAACTTAAAGCCCTCATTATGATT
CCTTTTATGTTATGCAGAAATATCCCTTTGGATACCTTACATTATCTTCATCACTAATCC
TTTGTGTACCTTTGTGTTCCTAACCGTCCACTCATTTTTGATTGATCCCCGGTATGGTAA
TACATACAACATACACAACATACAACAACATACTATACAATAGTGGAAACTCCATACAGT
TGATCTTTTGCACCCGCTTCAAGTCATGATGACTAATCAACCAATCTTGGGGTAAACAGT
TTCGACCGCTTATGTTTACTTCCACTTTACTCTCGTACATAGGGAATGAGAATCAATCTT
CTTACTGCAAATTCATAAGCTGTTTTGTTTCACTCATATAACTATCTGGTTTAACTCATC
GACCCGAATGATGAATAAAAAGAGAAAGGTATCTATTCAACACATCCAACCCCTTTCCTG
GAAATAAAGGAAAGGGGTAAAGGTTCATGTTCCAACGAATCACACGTAGAGATATTGATA
ACACACACGGAGTTAATGGTATTTCATAACTAATAGATTGAGCAGCAGCTCGTAGACCAC
CTGAAAAGGAATATTTATTATTCGATCCATATCCTGACATAAGAAGTCCAATAGGAGCAA
TACTGGAAATAGCGATCCATAAAAAAACGCCTATACTGAGATCGGCTAGAACAAGGCGAT
AGCCAAAAGGAATTACTAAATAGCTTAGTAGAATTGATATGACCGCTATAGATGGCCCCA
TACTGAATAAACGAACATCTCCTCTAGATGGGAGAAGATCCTCTTTCAAAAGTAGTTTGG
TCCCATCTGCTAGAGCTTGAAGAATTCCCAAGGGGCCGGCATATTCAGGCCCGATACGTT
GTTGTATCCCTGCAGATATTTCTCTTTCTAACCACACAATCACGAGTACGCCTATTGTGA
TTCCCGATACAGGAGTAAAAATAGGGACAAGCAGCCATAAGAGGTCATAGACCTCTTTTA
AGGATTCCGATCTGGAAAAAGAATTGATAGCTTGTACTTCTGTCGTATCAATTATCATTT
CAACGATCAACTTCTCCCATAATGATATCTATACTACCTAGTATCGTCATGATATCCGCC
AATTTCATTCTTTTAACTAGCTGAGGAAGAATTTGCAAATTGATGAAACCAGGTGGACGA
ATTTTCCATCTCCAGGGAAAAACACTATTATCCCCTATCAGAAAAATTCCCAATTCTCCC
TTTGGGGCTTCGACTCTCACATAAAGTTCTTGTTTCGACAATTCAAAAGTGGGAGAAGGC
TTTTTACTAATGAATCGATATTCAAAACCATTCCATTCGGAATCACTTGCTCTATCAAAG
CGTCGAACTTCTAAGTTCTCATAGGGCCCCCCCGGAATTCCTTCTAGAGCCTGTTGAATA
ATTTTTATGGATTCCGTCATTTCATTGATTCGTACTAAATAACGAGCTAATGAGTCTCCT
TCTTTTTGCCACTGGACTTCCCAATCGAATTCATCGTAACACTCATAATGATCAACTTTA
CGAAGATCCCATTGGATTCCGGAAGCTCGTAGCATTGGTCCCGATAAACCCCAATTTATT
GCTTCCTCCCCACCAATAATGCCCACCCCTTCAACTCGTTCCAAAAAAATGGGATTTTGC
GTAATAAGCTTTTGATATTCAACAATTCCTGTTAAAGAATAATCGCAGAAATCCAAACAT
TTATCTATCCAGCCATGAGGTAGATCAGCAGCGACTCCCCCGATACGGAAATAATTATGC
ATCATTCGCATACCTGTGGCAGCTTCGAATAGGTCATATAGCAATTCCCTTTCTCTGAAA
ATATAGAAGAAGGGAGTCTGTGAACCGATATCTGCCATAAAAGGTCCAAGCCATAATAAA
TGAGAAGCTATACGACTCAGCTCCAGCATAATTACTCTGATATAGCTGGCTCTTTTGGGT
ACTTGAATATTTCCTAATTGTTCTGGTGCATTTACCGTTATTGCCTCTGTGAACATAGTA
GCTAAATAATCCCAACGTGTTACATAAGGAAGATATTGTATAATTGTTCGGTTTTCCGCA
ATTTTTTCCATCCCTCTGTGTAAATAACCCAATACGGGTTCGCAGTCAATAACATCTTCA
CCATCTAGAGTAACGATAAGTCGAAGAACACCATGCATTGATGGGTGGTGAGGACCCATA
TTAACTATCATGAGGTCTTTTCTTGTAGCCGGTACATTCATATGTTTTCTTCTCCGATCC
ATTATTCTATGAATTGCCGAAAAGGAAATAAATGAGGTTCATCAAAATTCAAGATCTAAT
AAACCAAAGAATTCAAATAATCTTCAAATTAACGAGTTTTTGGTTCCCGAATATCTAATT
GATCGATTAATTTTTTATAACGCACTCTATTTTTCTTTGACAAATAAGCCAGCAGTCGTT
GACGTTTTCCCAGAATTTTCCGCAAACCTATCTGAGATAAATAATCTTTTCTGTGCAATT
CAAAATGTGAAGTAAGTCTCTGTATCTTATTGGTGAAACTGATTACTTGAAATTCAACAG
ACCCTTTGTTTTTTTCTTCTTTCGGAATAACTGAGATGAATGAATTTTTTACCATAACCA
TAAAAATAAAATTTCTCTCTTTTTTTTTTTTTTCCACAGATATGGATTTTACCAATC
AGGAATAATAATAATGCCAGTTATTTTGATCTGATACACCCAATAATCTGGATTTATTCA
TATATTACTTTATTCTATATTCTATCAAATCAAATCAAAATTAAATTCAAATGAATTGTA
AGTACAATTTGTAATTTGATTCGATAGAAGGGCAATTTTTGTACCCACAAAAGAAAATTA
TTTTGACCTAAGAAGATTCTGCCAAATCATTTCTAGATTCAATCCAAATCCGACACCTGA
TATATAGGAAATGTACCAACCATCAACTAATTCCGAATCGTGGATACATATGTATCCTTG
ACATACTGAAACGGCTGCCATTATTGGTATCAAACCAGTAGCGATTCATACAAGCTAAAT
CCTCTAATCGATAATTGGGCCAAAGAAACAATTTGAATTGAATGAATTTATTTATATCTG
TATCAATATGCTTGTCCTCATCCAAAAATTGTCCCCAGTTCCTTACATTGTTGTCATTGA
AAAATACCGGATTTCTATCCATAGCATTCCTATTTCCGGAATTGAAACAAATTAGAATTC
TCAATTCTCTACGACGCCTAGGGGATAGAATATTTTCAGGAACAAGCAAATCATAATGAT
TTTCGTCTCTATTCACAAGCATCTTTTTATGTTGTACAATGGATCCATTGAAATAATTCT
CATCGACATATCTTTTTTCTAGATATCTTCCATTAGTTTGGCATTTATTATTATGGACCA
GTGAGATACCTATGGTTTGATACATAATAAATTGTCTATCCCATTTTATAGACAGACGTA
CTGGTTCAAGAATCAATATTCCCTTTTTTATCAATTCTGGAAGAGTTGGATTCGTCTGAA
TTAACATTACATCCAGGTGCATTTCTCCTCCTTGAATAGAGGATATAGCAATTTCCTTTG
CATTTATTAGTCTAAGCAGGAAACAATATACCTTAACATTATTGAAAATTCTGTTATTCA

AAAGATCATCCCATCTCAATTGAAAAAGCAAATATTTTTTCAGGAATAACTCTTGTTTTG
CTTTCTTGTTACTCTCGGGTTGTCCTTTCTTTTCACGTTTTTGAATGTCCGATTCCGTGT
AATCCTTTTCAAAATCTTTTTGTCGTTTTCGTGCGTCTGAGCCAATATTTCCGTGGCCGA
GCTGTTCTTTTTCTTCTTGATTTCGATTCTTTAATTCAAGATATTCTTTTTGATTAGATG
ATATACGAAGATCCTTTTTTTGATTTTCATTAATGTTTTTGTTTTCACTAATGTTTTCAT
TTCCATTAAAAATCAAAAGAAGTAATTTTATTGGTATAATCCACGGTTTGATCTTATATG
CATCATAAAGTGGCACAAATTCTGAGAAGAACCAAGATTTCGTATTTAATATGGGGCGAT
ATAGCATTTCTTTATTCATTCCCATCAAAAAAAACTTTTTTTTTTGATTGGGTGGGTTGA
TTTCTTGATGAATCGTGAGATAGAAAAGATCTTTCTTATCAATCATTTGATAATAATCAG
TCTCGGTCTTAGTCATTTTATTAATGTTGGTCCCGGTATCCGTATCGGTCCAGGACTCAA
TATCGATATTCTTTCTAAGAAATAAATCGAAAATTTTCCACTCCAAATATTTTCTATCCG
TACTTTTACCCGTACCAATAATAGACTCTTCTCCTAGATAATCACTAATACATATATCCC
CCAGTACATAAAATGGTTCAATTTTAGGTGTGTTGTAATTATATGGAATCTCTCGGTCCT
CGTTTACTTGTAATGAGGATGGATAAATATATGAGTCTTTCCTATCCCCATAATTAATAT
ATTTATATGAAAAAAGATCATATCTGTAGTTTTTTTTTAATTTTGCTTTTTTTATCATCA
ATGAATTCACTTCATAATCATTTTTTTTCTCGTAATGAATGAATTGGGCTTTTTCATATG
AATTCTTTTTTGAGTCTTTATTTTGAATCGTACGACGTCGATTGACCCTAGTTCGCCATT
TTTGCGGTACTAATTTAGACCACCTAGCCTGAGATAAATTGTATTGATAATGACCCCTTA
ACCAGTTTTTCCACTCATTCATTCCAGAATTCGGAAGTTTTTTATGCCTTGATTTGGAAT
CTAATATTCTTCGTGTTCCAAAAAAATTCCGGATTCTATCCTTAAGAATAAGATATGCTT
CGCGATATTGAAGTAGAGATCTCAAATGATACTTCTTATTAATAGCTTGGATTTGTGATA
ATTTGTAAAATACAGATGCTTGTGAAAAGGAATATAGGTCACCAGAAATCTTTGATTTAT
TTTTACTAATATTAGAAATAGACTTTTTTATAGTCGAAATAAAGTGCATTTTTTTTTGAT
TTATTTCATCAATCCCTTCTTTGTGAATGTATTTATCGATAATCTTTTTTGTTGATTTAA
GGAAAAGTTGTACGTTGACTCTAGAAACATTAACAGTATATAGAAAGATATGTATGTATA
TTCTTTCGTCGAGAAATGTCAAAAAATAGTGCCATTTGCGTATGAATATGAATCTGTTAC
TTTTTCCTTTTGATATCTGCCAAATATGTTTCTGCGATTCTGATCTTTTATCACCACAAC
TTGTATCGTTAGGACTAATATTTATATCCGGAGTTAGAAATATTTTTCTTTTGTCTTTTG
CACCCCTTTCTATTTGATTTCTGATTAGGGTTGTTCTATCGGACAGATCTTTCTTTTTTT
TTTCTGTCAGTGAATAATTTGCCCAATCCATGAATCTAATTCGAATGGTCGATGTCGATT
TAGGAACAATTTTATTACTGCTTATGATTATGGAATCTTTTCCATTTTCATTCGGTTCAT
ATACTTTCTTCAATACAAATAAGAAAATTGGATTTACGTTTGCAAACTCTTTTATTTTTC
TTTTTAAAAATAGAATCGTTTTGATAATCCATCTTGTTTTTTCTTTTGAGACCTTTCTAA
ACTGTTTTGTTTTTTTTTTGAAAACTCTTAGAACTAGAAAACATTTTTTTTTCACTTTTA
TCTTTTTTTTTTCGAATTCATTATAAATAGGTTCAAAAAAGGAAGGTTGTTGTCGGGCAG
AACCAAAAGGTAGTTCGGTTTCCTTTCCCCAGATTGTTAAAAAACAAAAATTTTCTGTTT
TCCCCTTCTTTTGGATTGGATCTCTATGATGGGATCGTAGTTTGGATTTGCGCCAGGGTT
TCAGACAGAAAGGAAATAGGATTTTTATCTGAATACCATCTGTTAACCAGTTTTTCGGAA
ATTCTGTTTCTGATAATTGAACACCATTATAGGTGCATTTAACATGCATTTCTCTATTCC
ACTCCTTCAAATCCTCGTACCACTCGGGGAATTGAAATAAGAGCATACGGCCGAGGTTTT
TAGCTATTATCAATGAAGGCAATATGATGTATTTTCTAAGAATCGATTGGGTTACTAACA
TAGTACCTCTTATTGCTTGAGCAAATATAAAAGTATCCCATGCTTCTGCTATTGCTATCC
GTTCATTCTCGTTTTTTTTATCTTCAATTTTTTTTGCCTCTTCTTTTGCCTCTTCCTCCT
CAGAATCCGAAGTTTTGAGTTTCGGTCCTGTATCTATCCAATTTCTAAAAATTAGATTCA
TTGTTCGGGAGATATCAAAAGAAAAAAAAAAAGTTTTGTCTATTCTGTCCAAAAAAAGCA
GGGAATGTGCATTCGCTTGAAACATTTCCCAAATAACCATTTTACGTCTTTGAGCGCGTA
TGGATCCTTTTACTATATCCCGACGAAAATCTGATTGTTGCGAGTAACGTACCAAAGCCA
ACTCTTCTGCTTGATCACTATTAGTACTGGTACTAGTATGAGTATTGGTATTCTGATCCG
GATCCGCCTTATCAGTATAAATTACCACACGTTTGGCTTTTCTTGAACGAATCCCATGAT
TTTCTGTTGATTCTTCTGATTCTTCTTCATTTTCCTCCTCCCGCTCTTCAAAAGAATTGA
TCAATTTGTATGATCGTCGAGGAATCTTTTTACCGATTTCTTCTATTCCAATAGATTTAT
TTTGAATTGTTTGATTATTTTGATCAGTTGTAATTACATCGAATAGAAATTTCAAACATT
TTTTTTTATTTTCTGAATCAAATCTTCTTTGTTCGGATATTAAAACAAGCTTTTTGAAAT
TCAGACTAAAACCAGTTGTTGATTTCCTAGCTAATTCACTGATAGAGGTCAAGAAAGGAC
CAATGTCTGTCGATAATGGTTCTTCATTAAATGTATCCGTTTTATGTTCAAGTTTTTGGT
AATCAGTAGGAAGCCTATCATGAATCTTATTTATCCAAACCATTCCTATAGAATCTTCTG
TCCCTATAGAATCTTCTGTCCCTATAGAATCTTCTGTCGAAGTGATTGAGTCATCCACCA
TTGAACGTGAATACACTTTTTTTATTGTTCCACGATATGGTCCGTTTAAAAAAGGATCAT
ACACTCTAGGCAAGCATTCTTGGTTATTTTTATCTTCTTGGTTATTCTTATCTTCTTGGT
TATTCTTATCTTCTTGGTTATTCTTATCCTCTTGGTTATTTTTATCATTATACAATCTGG
TCCTTTTTTCAAGCACATCCATAGTAAGAGATCCCTTGTTTAGAACTTCTATTCGGTTTA
TGAATTCATTGCTCAAGCTGTACCTTTTTTGTTCATTGGTATAAACCCAATGATTATACA
GATCTTCGGGGGATAGTTTTTCGGTCGTACACAAAGACATCTTTCTTTGCATCATTTCCA
AAAAAATCGATAAACTGGGTGGATATGTAAAAGATATTATTTGTTTTCCATCAACTGGAC
ATACGTGAAAAAAATATTGTGACATTTCATTTCTTACAGCATTTTGAAATAAATTTTTTT
TTATATATCTCAATGGACGATTACATCGTTTATAGTCGAAAAGAAGATTCACAAGAGGTT

TTTCAAACCAGAAGAGGTCTTTATCTTCTTTCTCTTTAAGTATTTCTAACTTCAAAATTT
CTTGCCTCTTTTTTTTTTCATGTAGTTTTTTTGGATCCTCCCTTTCTTCCGAACAAAGGG
AAGGGTCTTCTTCGGTGGATCCCTCTTGTTCCTGTTTAGTCCCCTTCGTTTCGGAAGTTT
TTTCTATTTCTACATCTGTTTCTTCCTCACTTTCCCCCCTTTCTTCCGTTTTTGAGGTTT
CTTTCAGTTTCTTAGTGACAATAGGCGACGGTATTCTGCCTAAATAGTAGACACAGGTGA
TAAATAAGAGAATAGTAAAGATTCGAGCCATAGAATTTCTCAATTCTGACACAAGGTACT
TATTAGATCGAATAAGTACATTCGATCTAATAGAATGATTTTGCCGTATCCAGAATAATA
CCAATCCAACCCATTTCATGAAGAAAATGTGACCAATTAACCAACCAACAAAACTACTTG
TTACAAATAACATCTTGTTGTTGCATCGAAACATATAAATGTTGACTAATCTGACTAACG
TTGAACTTGGTAAAATGAAATGGTTGAATAATTGAAAAATGAGATTATTCAGGAATACAC
ATTGAATGCTGAGATTACGCATTGAATTTCTGGTAGTAGATCCATAATCCAAAAAGTGTT
TGTGATTGTTCCAGAAGAAATGAAACAAAAGATACGGTAGAACTAGGACAGTTATTGTAT
GAGGTCTACCCAATGCTAGATGCAGAGGCGCATAATAGATCGATATGAACATCATGAGCT
GTCCCGTAATAAAACCAGTTGTTGCTGATACCTCCTTCTCGGTTCCTTCTTCCATAATCC
TAGCTCGGAGAAGGAAAAGATAAGAGGGCCCTATGGAGAATGTGGTCAGAAATCCATAAT
AGAGTCCGACCACAACGACCGAATTTATTATCTTCATGCATAAGGATAATAGATTACCTA
GTAGAAAAGATTTCAAAATCATCACAAACCTCCCTTTTTTCTTTTCTATTGCAATTTCTC
GATTACTATATGATGATTTCTTCACTTTCCATAGATAGAAACAGATAGACTAGAAATGAC
ATCTTTTATGTCAATGATACCCTCTTCTTCGATTGAATGACATCTCTTATGTCAATGACA
CCAAAGGGATATTAAATGAATGGAATTGGGATATGGATGGAATATTGGGATATGGATGGA
ATATAATGAAATAGAGCCACTTTGAGGTTCCCTATGAAATGAGGCATGGAACGGAGCCAC
TACGAAGAAGTTCCGGGAGTTACGAAGGAAGCTTCGGGCTCATATTGGTCATGGGTTGAG
AGCGGGAGTTGAACTCTATGAGATCGAATCTCCCGTTATTCCTCAGTAGCTCAGTGGTAG
AGCGGTTGGCTGTTAACTGACTGGTCGTAGGTTCGAATCCTACTTGGGGAGATTTGATGA
ATTCTTAATTAAAGAATTCAGAATTACAGAATTAAAGGGCTCGCTTTGACCGTTAGGAGT
AGGTAACCCGTTCCCTGTCTTTGTTTCTATTGCACTCTATCTCATCGCATTCTGTTCTGC
GATATTTGAGAATCACCGTCAATACCTCGGTGTAGATCCGGGATAATCCTTTGTTCCATA
GCCCTGGGGCTATTTACAACTAGCCAATTCATAATTCTCAGATGATGTACTAGCAGTGCA
TCAAAGATGCAGTCATCGATTCTCCCGAGAGGCCACAATTACCGCGAGCAAACATATTAA
TGACGAGGAACGCATTTTTGCTATGCTACTAATACTTGTATTTGCTCTGCTATTCTTCCC
AAGCCTGGCTGAGGAAGAGTTACGGGGAGTAAAACACAAATATGCTGATTCGGGCCAGGC
ATACTATATGTATCGATTTTTTCCTTCACGATAAATAAAAAGAATAAGGCCATTTCGACA
AAAGACCCACACCCCAGTTCCATAGCTTTGCGTCCGCTATCCCGATCATGATTTTCCTAC
CCCCAGAGGTAAAGGAAAGGTCCTTCCCTTTTGGGCCGGTTGTGGGCGAGGAGGGATTCG
AACCCCCGACACCGTGGTTCGTAGCCACGTGCTCTAATCCTCTGAGCTACAGGCCCCACC
CCGTCTCCACTGGATCTGTTCCCTGGAGTACCCTCAAAAAGGAACCTTTTCTCCCCTCAG
CCATTTCGGGTTAAGAAGACGTGAAAGCGCCTTTCTCTCTATAAGAACAGCGCGTTCCGA
GGTGTGAAGTGGGAGAGAAGGGATGTCATAATTGGGTTTTGAATAAGACGACCTTTTCAT
TTTTTTCTTTTTGAGTAATAAGAATAAGAGGTGTTAAGCTTTTTATCATCCTGGCGTCGA
GCTATTTTTCCGCAGGACCTCCCCTACAGTATCGTCACCGCAGTAGAGTTTAACCACCAA
GTTCCGGGATGGATTGGTGTGGTTCCTCTACGCCTAGGACACCAGAATATCGAACCATGA
ACGAAGAAAGGTATGAGAGAAATATTGGCTAGTGATTGTGAAGCCCAAATTCTTGACTGG
AAGGGACACCAAAGGCCTCTGCCCTTCCATCCCTTGGATAGATAGAGAGGGAGGGCAGAG
CTTTTGGTTTTTTCATGTTGTCAAACAGTTGAACAATGAAAATAGATGGCGAGTGCCTGA
TCGAATTGATCGGGTCATGTAGGAACAAGGTTCAAATCTCTCGGTCTGTTAGGATGCCTC
AGCTGCATACATCACTGCACTTCCGCTTGACACCTATCGTAATGATAAACGGCTCGTCTC
GCCGTGACCTTATCTTGGATTCTCAAGACTTCTGTCGCTCCATCCCCGCAGGGGCAGAGA
ACCCGTCGCTGTCTCGGCTGTGCTACCGGAGGCTCTGGGGAAGTCGGAATAGGAGAGCAC
TCATCTTGGGGTGGGCTTACTACTTAGATGCTTTCAGCAGTTATCCGCTCCGCACTTGGC
TACCCAGCGTTTACCGTGGGCACGATAACTGGTACACCAGAGGTGCGTCCATCCCGGTCC
TCTCGTACTAGGGACAGGTCCTCTCAATGCCCTAACGCCCACACCGGATATGGACCGAAC
TGTCTCACGACGTTCTGAACCCAGCTCACGTACCGCTTTAATGGGCGAACAGCCCAACCC
TTGGAACATACTACAGCCCCAGGTGGCGAAGAGCCGACATCGAGGTGCCAAACCTTCCCG
TCGATGTGAACTCTTGGGGAAGATCAGCCTGTTATCCCTAGAGTAACTTTTATCCGTTGA
GCGACGGCCCTTCCACTCGACACCGTCGGATCACTAAGGCCGACTTTCGTCCCTGCTCGA
CGGGCGGGTCTTGCAGTCAAGCTCCCTTCTGCCTTTGCACTCGAGGGCCAATCTCCGTCC
GGCCCGAGGAAACTTTTGCACGCCTCCGTTACCTTTTGGGAGGCCTACGCCCCATAGAAA
CTGTCTACCTGAGACTGTCCCTTGGCCCGTAGGTCCTGACACAAGGTTAGAATTCTAGCT
CTTCCAGAGTGGTATCTCACTGATGGCTCGGGCCCCCCCGGAAGGAGGCCTTCTTCGCCC
TCCACCTAAGCTGCGCAGGAAAGGCCCAAAGCCAATCCCAGGGAACAGTAAAGCTTCATA
GGGTCTTTCTGTCCAGGTGCAGGTAGTCCGCATCTTCACAGACATGTCTATTTCACCGAG
CCTCTCTCCGAGACAGTGCCCAGATCGTTACGCCTTTCGTGCGGGTCGGAACTTACCCGA
CAAGGAATTTCGCTACCTTAGGACCGTTATAGTTACGGCCGCCGTTCACCGGGGCTTCGG
TCGCCGGCTCCCCTGTCATCAGGTCACCAACTTCCTTAACCTTCCGGCACTGGGCAGGCG
TCAGCCCCCATACATGGTCTTACGACTTTGCGGAGACCTGTGTTTTTGGTAAACAGTCGC
CCGGGCCTGGTCACTGCGACCTCCTTTGTGAGGAGGCACCCCTTCTCCCGAAGTTACGGG

GCTATTTTGCCGAGTTCCTTAGAGAGAGTTGTCTCGTGCCCCTAGGTATTCTCTACCTAC
CCACCTGTGTCGGTTTCGGGTACAGGTACCCTTTTGTTGAAGGTCCTTCGAGCTTTTCCT
GGGAGTATGGCATGAGTTACTTCAGCGCCGTAGCGCCTGGTACTCGGACATTGGCTCGGG
GCATTTCCTCTACCCCTTCTTACCCTGCCCTAAAAAAGCAAGGTGACCTTGCGTCCTTGA
ACCGATAACCATCTTTCGGCTAACCTAGCCTCCTCCGTACCTCGGGACCAACAAGGGGTA
GTACAGGAATATTCACCTGTTGTCCATCGACTACGCCTTTCGGCCTGATCTTAGGCCCTG
ACTCACCCTCCGTGGACGAACCTTGCGGAGGAACCCTTAGGTTTTCGGGGCATTGGATTC
TCACCAATGTTTGCGTTACTCAAGCCGACATTCTCGCTTCCGCTTCGTCCACACCTGCTC
GCGCGGGAGCTTCCCTCTAAGCGGAACGCTCCCCTACCGATGCATTTTTACATCCCACAG
CTTCGGCAGATCGCTTAGCCCCGTTCATCTTCGGCGCAAGAGCACTCGATCAGTGAGCTA
TTACGCACTCTTTCAAGGGTGGCTGCTTCTAGGCAAACCTCCTGGCTGTCTCTGCACCCC
TACCTCCTTTATTACTGAGCGGTCATTTAGGGGCCTTAGCTGGTGATCCGGGCTGTTTCC
CTCTCGACGATGAAGCTTATCCCCCATCGTCTCACTGTCCGAACTTGACCCCTGTTATTT
TGGGGTCATATCTAGTATTCAGAGTTTGCCTCGATTTGGTACCGCTCTCGCGGCCCGCAC
CGAAACAGCGCTTTACCCCTAGATGTCCAGTCAACTGCTGCGCCTCAACGCATTTCGGGG
AGAACCAGCTAGCTCTGGGTTCGAGTGGCATTTCACCCCTAACCACAACTCATCCGCTGA
TTCTTCAACATCAGTCGGTTCGGACCTCCACTTAGTTTCACCCAAGCTTCATCCTGGTCA
TGGATAGATCACCCAGGTTCGGGTCCATAAGCAGTGACAATTACCCTATGAAGACGCGCT
TTCGCTACGGCTCCGGTGGGTTCCCTTAACCAAGCCACTGCCTAAGAGTCGCCGGCTCAT
TCTTCAACAGGCACGCGGTCAGAGATTTCTCCTCCCACTGCTTGGGAGCTCACGGTTTCA
TGTTCTATTTCACTCCCCGATGGGGGTTCTTTTCACCTTTCCCTCACGGTACTACTTCGC
TATCGGTCACCCAGGAGTATTTAGCCTTGCAAGGGGGTCCTTGCTGATTCACACGGGATT
CCACGTGCCCCATGCTACTCGGGTCAGAGCGTAAGCTAGTGATGCTTTCGGCTACTGGAC
TCTATCCATCTAGGGTGCAGCACTCCACCGCTTCGCCTAGCAGCACGACGCTTGTATTGC
TCTCCCACAACCTCGTTTTCACGGTTTAGGCTGCTCCCATTTCGCTCGCCGCTACTACGG
AAATCGCTTTTGCTTTCTTTTCCTCTGGCTACTAAGATGTTTCAGTTCGCCAGGTTGTCT
CTTGCCTGCCCATGGATTCAGCAGCAGTTCGAAAGGTTGACCTATTCGGGAATCTCCGGA
TCTATGCTTATTTTCAACTTCCCGAAGCATTTCGTCGCTTACTACGCCCTTCCTCCTCTC
TGGGTGCCTAGGTATCCACCGTAAGCCTTTCCTCGTTTGAACCTCGCCATTAATGTGTTG
GCTATGCCATCCTAAGGTGCTGCTAAATGGAAGGATCTTATCAACGTCCATGAATGAGAA
ATCATAGATCGAACTGCCGAATCGGAAAAATTGGGTGCTATCATATACCTTTGCATCGGC
TAAGGTCACGAGCTGGAGATAAGCGGACTCGAACCGCTGACATCCGCCACAGGGTAAACC
ACCGCCTCTCGGGCCTCCCCGACTGATTCTATCATAGAGGCCAACGATAGACAATAACTC
CCCCCCGAACACAGCTTACAACTTTCATCGTACTGTGCTCTCCAAAGAGCAACTCTTCTC
AAAATCTCAAAGGGTGCTGAGTTGGAATCCCATTCTAAGGATTCTTGTGGTTCCGGAGGA
TCCAGCTACACGAGAACCAGGAACGGGGAGCTCTTCCCCTTTTTCCGCCCGACTCTTTGG
TCTTAAGAATGCTGGTTTTAAGAATGAGTGATTGCCCTTCTCCGACCCTTACTGCCCAAC
CGGAGAGCGGACAGCTAATGCGTTCCACTTATTGAACAGGGTCTATGGTCGGTCTGTGAC
CCCTGGATGCCGAAGGCGTCCTTGGGGTGATCTCGTAGTTCCTACGGGGTGGAGACGATG
GGGTCGGTCCATGGATTTTCCTTCCTTTTGCCACATTTCGCTCAAAGGGTTGAAGGGAGA
TAGTGCATCAAGCTGTTCGCAAGGGCCAACTTGATCCTCTTCCCCAGGGATCCAGATGAG
GGAACCCTAGGAGAGCCGCCGACTCCAACTATCGTCCATGTACGATCCATACTAGATCTG
ACCAACTGCCCATCCTACCTCCTCTACGTTCTTGACAGCCCATCTTTGTCTCAGTAGAGT
CTTTCAGTGGCATGTTTCGGTCCTCTTCCCCATTACTTAGAAAAAGTGAGCCACCGGTTC
AGGTACAAGATACTATCATTACCGCCTGGACAATTAGACATCCAACCCGTAATCGCAACG
ACCCAATTGCAAGAGCGGAGCTCTACCAACTGAGCTATATCCCCCCGAGCCCAGTGGAGC
ATGCATGAAGGAGTCAGATGCTTCTTCGATTCTTTTCCCTGGCGCAGCTGGGCCATCCTG
GACTTGAACCAGAGACCTCGCCCGTGAAGTAAATCATCGCACCTACGATCCAACCAATTG
GGAGAGAATCAATAGATTCTTTTTCGGGGGCGATTCATCCTTCCCGAACGCAGCATACAA
CTCTCCGTTGTACTGCGCTCTCCAAGTGTGCTTGTTCCCACCTTCTTCCTTACCATGGCA
AGTATTTGTGAAATAACTCCGATGAGAAGAAAAAGAAGGCGTTAAGAGACCCTCCTGGCC
CAACCCTAGACACTCTAAGATCCTTTTTCAAACCTGCTCCCATTTTGAGTCAAGAGATAG
ATAAATAGACACATCCCATTGCACTGATCGGGGGGCGTTCGTAGTGACTGAGGGGGTCGA
AGACCAAGAAGTGAGTTATTTATACCAAGCATTCTTCTTATGGCTAGATCCAATCTCCTG
GTCCCTGCGGAAAGGAAAAAGAATTTCACGTTCTTCCTTTCGGGAAGGGAGGATTAGGGA
AATCCTATTGATTGCTGCTTTCTCCAGACCTCCGCGGGAAAAGCATGAAAAAAAAAGGCT
CGAATGGTACGATCCCTCCGTCACCCCAGAATGAAAGGGGTGATCTCGTAGTTCTTGGTC
TGTGAAGATGCGTTGTTAGGTGCTCCATTTTCCCATTGAGGCCGAACCTAAACCTGTGCT
CGAGAGATAGCTGTCCATACACTGATAAGGGATGTATGGATTCTCGAGAAGAGAGGAGCC
ATGGTGGTCCCCCCCGGACCGCCCGGATCCCACGAGTGAATAGAAAGTTGGATCTACATT
GGATCTCACCTGAATCGCCCCATCTATCCTCCTGAGGAGAAGTTTGGTTTCAAACTCCGG
TTCGAACAGGAGGAGTACGCCATGCTAATGTGCCTTGGATGATCCACATCCCCGGGTCAG
GCGCTGATGAGCACATTGAACTATCCATGTGGCTGAGAGCCCTCACAGCCCAGGCACAAC
GACGCAATTATCAGGGGCGCGCTCTACCACTGAGCTAATAGCCCTTGCGGGCCCCCCACG
GGGAGGCCCGCTATGCCAAAAGCGAGAGAAAGCCCATCCCTCTCTTTCCTTTTTGCGCCC
CCATGTCGCCACACGGGAGGGGCATGGGGACGTAAAAAAGGGGATCCTATCAACTTGTTC

CGACCTAGGATAATAAGCTCATGAGCTTGTCTTACTTCACCGTCGAGAAACGAAAGAAGA
CTTCCATCTCCAAGCTTAGCTCAGACGTAGCTCGCTTCTTTAGCTCTCCCTGAAAAGGAG
GTGATCCAGCCGCACCTTCCAGTACGGCTACCTTGTTACGACTTCACTCCAGTCACTAGC
CCCGCCTTCGGCATCCCCCTCCTTGCGGTTAAGGTAACGACTTCGGGCATAGCCAGCTCC
CACAGTGTGACGGGCGGTGTGTACAAGGCCCGGGAACGAATTCACCGCCGTATGGCTGAC
CGGCGATTACTAGCGATTCCGGCTTCATGCAGGCGAGTTGCAGCCTGCAATCCGAACTTA
GGACGGGTTTTTGGAGTTAGCTCACCCTGGCGGGATCGCGACCCTTTGTCCCGGCCATTG
TAGCACGTGTGTCGCCCAGGGCATAAGGGGCATGATGACTTGACGTCATCCCCACCTTCC
TCCGGCTTATCACCGGCGGTCTGTTCAGGGTTCCAAACTCAATGGTGGCAACTAAACACG
AGGGTTGCGCTCGTTGCGGGACTTAACCCAACATCTCACGACACGAGCTGACGACAGCCA
TGCACCACCTGTGTCCGCGTTCCCGAAGGCACCCCTCTCTTTCAAGAGGATTCACGGCAT
GTCAAGCCCTGGTAAGGTTCTTCGCGTTGCATCGAATTAAACCACATGCTCCACCGCTTG
TGCGGGCCCCCGTCAATTCCTTTGAGTTTCATTCTTGCGACCGTACTCCCCAGGCGGGAT
ACTTAACGCGTTAGCTACAGCACTGCACGGGTCGATACGCACAGCGCCTAGTATCCATCG
TTTACGGCTTGGACTACCAGGGTATCTAATCCCATTCGCTCCCCTAGCTTTCGTCTCTCA
GTGTCAGTGTCGGCCCAGCAGGGTGCTTTCGCCGTTGGTGTTCTTTCCGATCTCTACGCA
TTTCACCGCTCCACCGGAAATTCCCTCTGCCCCTACCGTACTCCAGCTTGGTAGTTTCCA
CCGCCTGTCCAGGGTTGAGCCCTGGGATTGGACGGCGGACTTGAAAAGCCACCTACAGAC
GCCTTACGCCCAATCATTCCAGATAACGCTTGCATCCTCTGTCTTACCGCGGCTGCTGGC
ACAGAGTTAGCCGATGCTTATTCCTCAGATACCGTCATTGCTTCTTCTCCGGGAAAAGAA
GTTCACGACCCGTGGGCCTTCTACCTCCACGCGGCATTGCTCCGTCAGGCTTTCGCCCAT
TGCGGAAAATTCCCCACTGCTGCCTCCCGTAGGAGTCTGGGCCGTGTCTCAGTCCCAGTG
TGGCTGATCATCCTCTCGGACCAGCTACTGATCATCGCCTTGGTAAGCTATTGCCTCACC
AACTAGCTAATCAGACGCAAGCCCCTCCTCGGGCAGATTCCTCCTTTTGCTCCTCAGCCT
ACGGGGTATTAGCAGCCGTTTCCAGTTGTTGTTCCCCTCCCAAGGGCAGGTTCTTACGCG
TTACTCACCCGTCCGCCACTGGAAACACCACTTCCCGTCCGACTTGCATGTGTTAGGCAT
GCCGCCAGCGTTCATCCTGAGCCGGGATCGAACTCTCCATGAGATTCATAGTTGCATTAC
TTACCAATCTTCCGGTTCGTAGACAAAGCTGATTCGGAATTGTCTTTCATTCCAAGGCTT
GTATCCATGCGCTTCATATTAGCCTGGAGTTCGCTCCCAGCAATATAGCCATCCCTACCC
TCTCACGTCAATCCCACGAGCCTCTTATCCATTCTCATTCGATCACGGCGGGGGAGCAAG
TCAAAATAGAAAAACTCACATTGCGTTGGGTTTAGGGATAATCAGGCTCGAACTGATGAC
TTCCACCACGTCAAGGTGACACTCTACCGCTGAGTTATATCCCTTCCCTGCCCCCATCGA
GAAATAGAACTGACTAATCCTAAGGCAAAGGGTCGAGAAACTCAACGCCACTCTTCCTGA
ACAACTCGGAGCCGGGACTTCTTTTCGCACTATTACGGATACGAAAAGAATGGAAAAATT
GGATTCAATTGTCAACTGCTCCTATCGGAAATAGGATTGACTACGGATTCGAGCCATAGC
ACATGGTTTCATAAAATCCGTACGATTTTCCCGATCTAAATCGAGCAGGTTTTACAGGAA
GAAGATTTTGTTCAGCATGTTCTATTCGATACTGGTAGGAGAAGAACCCGACTCGGTATT
GTTAAAAAAAGAGAGGAAGCAGAACCAAGTCAAGATGATACGGATCAACCCCTTCTTCTT
GCGCCAAAGATCTTACCATTTCCGAAGGAACTGGAGCTACATCTCTTTTCAATTTCCATT
CAAGAGTTCTTATGTGTTTCCACGCCCTTTTGAGACCTCGAAAAAGGGACAAATTCCTTT
TCTTAGGTTCTTAGGAACACATACAAGATTCGTCACTACAAAAAGGATAATGGTAACCCC
AACATTAACTACTTCATTTCTGAATTTAATAGTAATAGAAATACATGTCCTACCGAGACA
GAATTTGTAACTTGCTATCCTCTTGCCTAGCAGGCAAAGATTGACCTCCGTGGAAAGACT
GATTCATTCGGATCGACATGAGGGTCCAACTACATTGCATTGCCAGAATCCATTTGTATA
TTTGAAACAGGTTGACCTCCTTGCTTCTCTCATGGTACAATCCTCTTCCCGCTGAGCCCC
CCTTTCTCCTCGGTCCACAGAGACAAAATGTAGGACTGGTGCCAACAGTTCATCACGGAA
GAAAGGACTCACCGAGCCGGGATCACTAACTAATACTAATATAATAGAAAAGAACTGTCT
TTTCTGTATACTTTCCCGGCTCCGTTGCTACCGCGGGCCTTACGCAATCGATCGGATCAT
ATAGATATCCCTTCAACACAACATAGGTCATCGAAAGGATCTCGGACGACCCGCCAAAGC
ACGAAAGCCAGGATCTTTCAGAAAATGGATTCCTATTCGAAGAGTGCATAACCGCATGGA
TAAGCTCACACCAACCCGTCAATTTGGGATCCAATTCGGGATTTTCCTTGGGAGGTATCG
GGAAGGAATTGGAATGTAATAATATCGATTCATACAGAAGAAAAGGTTCTCTATTGATTC
AAACGCTGTACCTATGGGATAGAGGAAGAGGAAAAAAACCGAAGATTTCACATAGTACTTT
TGATCGAAAAATCAATCGGATTTATTTCGTACCCCTCGTTCGATGAGAAAATGGGTCAGA
TTCTACAGGATCAAACCTATGGGACTTAAGGAATGATGGAAGGGAATAAAAAGAAAAGAG
AGGGAAAGAAAATCGAAATAAAGAATCAAAGAAAATAAATAAATAAAAAAATACAAAAATA
AATAAAGATTCCAAATGAACAAATTCAAACTCAAAAAGGATCTTTCTGATTCTCGAAGAA
TGAGGGCAAAGGGATTGATCGAGAAAGATCTCTTGTTCTTATTATAAGATCGTGATTTGA
TCCGCATATTTTGGTAAAAAGAATAATCTTCTCCTTTGATCATAATCAAAAATGGAAAGT
GTTCAATTGGAACATGAGAACGTGACTGAATTGGTCCTAGTTACTCTTCGGGACGGAGTG
GAAGAAGGGAGGAGATTCTCGAACGAGGAAAAGGACCCAATTACTTCGAAAGAATTGAAC
GAGGAGCCGTATGAGGTGAAAATCTCATGTACGGTTCTGTAGAGTGACAGTAAGGGTGAC
TTATCTGTCAACTTTTCCACTATCACCCCCAAAAAACCAAACTCTGCCTTACGTAAAGTT
GCCAGAGTACGATTAACCTCTGGATTTGAAATCACTGCTTATATACCTGGTATTGGCCAT
AATTCACAAGAACATTCTGTAGTCTTAGTAAGAGGAGGAAGGGTTAAGGATTTACCCGGT
GTGAGATATCACATTGTTCGAGGAACCTTAGATGCTGTCGGAGTAAAGGATCGTCAACAA

GGGCGTTCTAGTGCGTTGTAGATTCTTATCCAAGACTTGTATCATTGGATGATGCCATGT
GAATCGCTAGAAACATGTGAAGTGTATGGCTAACCCAATCACGAAAGTTTCGTAAGGGGA
CTGGAGCAGGCTACCATGAGACAAAAGATCTTCTTTCTAAAGAGATTCGATTCGGAACTA
TTATATGTCCAAGGTTCAATATTGAAATCATTTCAGAGGTTTTCCCTTACTTTGTCCGTG
TCAACAAACAATTCGAAATACCTCGACTTTTTCAGAACAGGTCCGAGTCAAATAGCAATG
ATTCGAAGCACTTCTTTTTACACTATTTCGGAAACCCAAGGACTCGATCGTATGGATATG
TAAAATACAGGATTTCCAATCCTAGCAGGAAAAGGAGGGAAACGGCTACTCAATTTAAAG
TGAGTAAACAGAATTCCATACTCGATTTCATAGATACATATCAAATTCTGTGGAAAGCCG
TATTCGATGAAAGTCGTATGTACGGCTTGGAGGGAGATCTTTCATATCTTTCGAGATCCA
CCCTACAATATGGGGTCAAAAAGCCAAAATAAGTGATTCGTTTTTAGCCCGTATAAAAAG
AAAACGGATTCTTGAACCTCTTTCACGCTCATGTCACGTCGAGGTACTGCAGAAGAAAAA
ACTGCAAAATCCGATCCAATTTATCGTAATCGATTAGTTAACATGTTGGTTAACCGTATT
CTGAAACACGGAAAAAAATCATTGGCTTATCAAATTATCTATCAAGCCGTGAAAAAGATT
CAACAAAAGACAGAAACAAATCCACTATCTGTTTTACGTCAAGCAATACGTGGAGTAACT
CCCGATATAGCAGTCAAAGCAAGACGTGTAGGTGGATCGACTCATCAAGTTCCTATTGAA
ATAGAATCTACACAAGGAAAAGCACTTGCCATTCGTTGGTTATTAGGGGCATCCCGAAAA
CGTCCGGGTCGAAATATGGCTTTCAAATTAAGTTCCGAATTAGTGGATGCTGCCAAAGGG
AGTGGCGATGCCATACGCAAAAAGGAAGAGACTCATAGAATGGCAGAGGCAAATAGAGCT
TTTGCACATTTTCGTTAATCCATGAACAGGATCTATATAGACACATAGATCCGTGGATCC
ATACATCTCGATCGGAAAAGAATCAATAGAAAAAGAAAGAATTGGAATGGATCGATATCT
TTCTCGAAACAAACGAAAAGGAAAAGAAAGATGAAACATAAATCATGGATCAACTAAGCC
CTCTCGGGGGCTTTCTTAAGAATAAGAAGGAGGAATCTCATGGAAATACCATGGAATAAG
GTTTGATCCTGTTCATGGGGATTCCGTAAATATCCCATTCCAAAAATCGAAAGTTCGAAA
CAATTGGGACTTTTTCGGAGATTGGATGCAGTTACTAATTCAGGATCTGGCATGTACAGA
ATGAAAACTTCATTCTCGATTCTACGAGAATTTTTATGAAAGCGTTTCATTTGCTTCTCT
TCCATGGAAGTTTCATTTTCCCAGAATGTATCCTCATTTTTGGCCTAATTCTTCTTCTGA
TGATCGATTCAACCTCTGATCAAAAAGATATACCTTGGTTATATTTCATCTCTTCAACAA
GTTTAGTAATGAGCATAACGGCCCTATTGTTCCGATGGAGAGAAGAACCTATGATTAGCT
TTTCGGGAAATTTCCAAACGAACAATTTCAACGAAATCTTTCAATTTCTTATTTTACTAT
GTTCAACTCTATGTATTCCTCTATCCGTAGAGTACATTGAATGTACAGAAATGGCTATAA
CAGAGTTTTTGTTATTCGTATTAACAGCTACTCTAGGAGGAATGTTTTTATGTGGTGCTA
ACGATTTAATAACTATCTTTGTAGCTCCAGAATCTTTCAGTTTATGCTCCTACCTATTAT
CTGGATATACCAAGAGAGATGTACGGTCTAATGAGGCTACTACGAAATATTTACTCATGG
GTGGGGCAAGCTCTTCTATTCTGGTTCATGGTTTCTCTTGGCTATATGGTTCATCCGGGG
GAGAGATCGAGCTTCAAGAAATAGTGAATGGTCTTATCAATACACAAATGTATAACTCCC
CAGGAATTTCCATTGCGCTTATATCCATCACTGTAGGAATTGGGTTCAAGCTTTCCCCAG
CCCCTTCTCATCAATGGACTCCTGACGTATACGAAGGAGTGCGGTTCGTTCGACAAATTC
CTACCTCTATATCTATCTCTGAGATGTTTGGATTTTTCAAAACTCCATGGACATGCAGAA
GAGAAATGCTATCCCCACTCGGACCAAGACATAACTTTTACCAAAAGTTTATTGTGATCT
TTTTGTTCAAATAACAATTAAGGTGAAGCAGGGTCAGGAACAACGAATCTCTTTATGATA
AACAGATCCATTTTGCAAGTTCGTTATTACGGGTAGTTCCTACAAAGGATCGGACTAATG
ACGTATACAATACTTGAATTATCGATGTAGATGCTACATAGTTGGTTCTCATCCTTCAGA
GACTACGAGTGTAATAGGAGCATCCGTCGACAAAAGGATCACCCTAAGATGATCATCTCA
TGGCTATTGAGAACGAATCAAATCAGATGGTTCTATTTCTCAATCTTTCTGACTTGTGCT
CCTACGGAACCGGGGTCGAAAAGATTGAAAAAGTCAGTCATTCACAACCACTGATGAAGG
ATTCCTCGAAAAGTTAAGGATTAGTAATCCTTTTTAGAAATCGAATGGATTCGGTCTTAT
ACATACGCGAGGAAGGTAATCAAAAAAGAAAGAAGGTGAGTTCTTCTTTCTTTTATCACT
TAGGAGCCGTGCGAGATGAAAGTCTCATGCACGGTTTTGAATGAGAGAAAGAAGTGAGGA
ATCCTCTTTTCGACTCTGACTCTCCCACTCCAGTCGTTGCTTTTCTTTCTGTTACTTCGA
AAGTAGCTGCTTCAGCTTCAGCCACTCGAATTTTCGATATTCCTTTTTATTTCTCATCAA
ACGAATGGCATCTTCTTCTGGAAATCCTAGCTATTCTTAGCATGATATTGGGGAATCTCA
TTGCTATTACTCAAACAAGCATGAAACGTATGCTTGCATATTCGTCCATAGGTCAAATCG
GATATGTAATTATTGGAATAATTGTTGGAGACTCAAATGATGGATATGCAAGCATGATAA
CTTATATGCTGTTCTATATCTCCATGAATCTAGGAACTTTTGCTCGCATTGTCTCATTTG
GTCTACGTACCGGAACTGATAACATTCGAGATTATGCAGGATTATACACGAAAGATCCTT
TTTTGGCTCTCTCTTCAGCCCTATGTCTCTTATCCCTAGGAGGTCTTCCTCCACTAGCAG
GTTTTTTCGGAAAACTCCATCTATTCTGGTGTGGATGGCAGGCAGGCCTATATTTCTTGG
TTTCAATAGGACTCCTTACGAGCGTTGTTTCTATCTACTATTATCTAAAAATAATCAAGT
TATTAATGACTGGACGAAACCAAGAAATAACCCCTCACGTGCGAAATTATAGAAGATCCC
CCTTAAGATCAAACAATTCCATCGAATTGAGTATGATTGTATGTGTGATAGCATCTACTA
TACCAGGAATATCAATGAATCCAATTATTGCAATTGCTCAGGATACCCTCTTTTAGCTTC
TAGGGTCTATTTCTTAGTTCAAGATCCCTTTTACTAACTGGAATAAAAGAATTAGTAGAT
CTGTTCCGCCCAAAATGGGAATGGGCTGGGGTTATGAACTTATAATCATGGAATCGACTC
GATCATCAGATTATAAGTTCATTCCATACCGGACCAGACCGGAATAGGGTTATGTACATT
CTCATTATGAGAAGGGGTCATTCGAGCATATGTAAATAGAGACTATGTTTACATATGGAT
CCCTACGTCGTTACATTCCATTTAGGATTAGGAATAGGCGTAATCGGACCTGCTTTTTAC

ATATCTATCGTTATTTGGGTACCATATTAACTTCTTTGGGCTTCGATTGAATCGAGAAAT
AGGTTTGATTGTACATCTTTTTGATATATATAAGGTATCCTCCGGATAATTCAAATCGAA
GCAATTTGATGTCCGACTCGGGCCTATATGACATGACCGATCGATAGAAATACTCCAAAA
CTCCACCTTTGTCATATATTCCATATATCACACTAGATAGATATCATATTCATGGAATAC
GATTCACTTTCAAGATGCCTTGATGGTGAAATGGTAGACACGCGAGACTCAAAATCTCGT
GCTAAAGAGCGTGGAGGTTCGAGTCCTCTTCAAGGCATAATATTGAAATGCTCATTGAAT
GAGCAATTCAATAACAGATCTCGGATCTAATCAATATTGATATACATATACCAAGTATCT
GTTGATACGAAGTATTCCATCGATCCCCACGATCCGAGTCCGGGCTGTTGGAATTGGAAT
AGGTTCGGCAGCGGATCACGAAATCTTGGCGATCTTCTCTATCTAATGAATGAGGAGTCC
GTTTTGAAATCGTCCGCCCTGCACCCACCCCCCGAGTATATGCTTCAACAGGAATCACAC
AAGGGTAAATTGATACAATAGAAACCTCTGGTAAAATGCCCGCCCGTAACCCAACAGATA
AAGTACATAGTCCGTTTTAGCCTGTTACATGAATCAAATGTTTCATTTCATCCGGGAAAA
GCCATCTCTTTCTCAACAATGCCTTTGTCATTTGATCCAATAGCGTTCCGTTAGATAGGA
ACAGATTTGATAAATACTGATAACTCTCGGATGGAGTATTAGAACGGAAAGATCCATTAG
ATAATGAACTATTGGTTCGAAGCCATCTCTGGCGATGAATCAACAATTCGAAGTGCTTTT
CTTGCGTATTCTTGATGAACCAGCGTTTATATATAGATGTAGGAGGATTTGTTTGGGAAG
TAATAAGCCCTTTTGACATCTCTTCATCTGCAAAGAATTCTCGGCGTGAAAACACAGAGA
CAAAGGGCTGATCTTTGAATAGGAAAAAGAATGGATCTGCAGGGTCCCAAATGAATTGGC
TTATTCGAAAAAAGCCTTGTTCTTTGGAAGATCTATCTCGTGTCTGGTACTGCATGGTTC
CACTCTGCAAGAACTCCGAATCATTCTCTTGAAGCTCATCCTCTTTATGATAAATGATCC
GCTTGCCCCGAAATGACCTGGCCCAATAGGGAAATCCCAATTCATTGGTCCTTTCGATAC
AATCAAATAGATTGCCACAAGGGCGCCATATTCTAGGAGCCCAAACTATGTGATTGAATA
AATCCTCCTCTATCTGTTGCGGGTCGAGGGCTCCTTCTCTTTCCCCTTCTTCAAACTCCG
ATTCGTATTTTTCATATAGAAATCTCTGATCAACGATAGAACAAGATCCGTTTTGCATCA
TATCTAACTGATTCCTTGGTTCGGACCGAAGAAGCAATGTCACTCGATCATTATCAAACT
GACTGCAATCTTTTTCTGTCCGTGAGGATCCCAACAGAGCGCCTTCGACTTCTAATAGGC
CATGAACTAGATCAGAATCATTCTCAACGAATCCATAAGAAGTGATCCAATTTTTTTCAT
CGGGTCCGGGTGGAGACCAAAGATCTTGAGCGACCGATCCGGCAGAACAACTCAAAAGAT
AAAGAAGTATCGTTAATTTCTTCATGCTCGTTCCAAGTTCGAAGTACCATTTGTACAAAT
AAGAATCCCCTTCCTTACATGATTTCTTCTTCATATAGATAGATATAGGATCTATGGGGC
AATTACTTAGAAGTACATTTTGTGCAACAGCCCTTCCTATCTGATAGAAAAGGATCCCAT
GATCCTGAACCGATCTTACCTGGGATCGCAAATCCCAAGTTTGTCTATGAAGAGCTGATC
TAATTGTATTAGTGTCTATAATTGATTTCTTCTGTGTAATACTAATTGATAGGGCCTCAT
TGGTAAGTGCTACAAGATCTCGTGCATTGGAACCCATGGTTATGGACCCGAATCTGTTAC
TGTTAGTATGGAACATTTTCTTTTCCAAGTGAAATCCCCTAGTATATGAAAGAATGAAAA
AGTGCTTTCGTTGTTGTGGAATAAGAAGCCTTCGTATCTTAATGCATGTATTTAATTTAT
TCGGAGCTATTAGAGTGGGATCCACTTTTTGGGGAATATGAGTCGAAGCAATAACAAGAA
TATTTCTAGTGGAACATCTTTCACTATCTCTGGAGAGATAGTTCACTAATAGACCGAGGG
ATAAGTAATTCGACTCATTCACATACAGATCATGAATGTTTGGAATCCATATTATGCAAG
GGGACATTGCTTTTGCTAATTCTAATTGAAGGGTGGTATCAAATCGGTCTATTTTCGGCG
TCATATACATAGTTAGCACATTCGTCATAGTTAGCAGCTCCGTATCAAGGTCATCATCAA
TATCGTCACTATCATCAATATCGATATCGTCACTATCATCAATATCGATATCGTCAATAA
GATAACCTTTAGGCTTGTCATCCAGGAACTTGTTCGGAAATACCGTAATGAAAGGAACAT
AGGAGTTTGTCGCTAGGTATTTGACCAAATAGGATCGTCCAGTTCCTATAGAACCTATCA
CTAAAATACCCCTAGAAGGGGATAGGGCTAAGCGGAGCGAAAAGGGTTTTCCATGAGATG
GGAAATGAGAACTATTAGCCCCACACGAGGTTTGTGAATAAGTGATTGTCTGATAATGAG
CAAGGAATATCCGTCTTTCTGCTAAACAGGATCTATTGAACTCATAATTCATTAGATACT
TTTTATGAATGTCAACTAAGTATCGTAAGTAAATGGATCCCGGTTGTTCAATCATTTGAT
AACCAGAGTCATTCTTTGATAAACGATCACTATGAGTCAGACTCAATAGAATTTGATCAA
TCCTTTTTTCCGTCGTTAAGGTGGAGAACTGAACCAAGAATTCTCTTTCTTCATCATCAA
TCGAATCACTGTTCGCGACCCAGGATTCTATTTTATCATCAATCCAATCACCGTTCACGT
TTTTTCTTTTTCTTATCAATGAATAGATCTCTTTACTTGTACGACTTAGATGTCTCGTAT
TTCTCGAAAAAGTGATTCGATTGATGGGATTTGGTATGATACTGATGAGATCGATGAGAT
TGATATTCAAATATTTCTTCTTAGAACGTATTGATTTGACCCCATAAGCGGGACCACCAC
CCAATAGCATGTTGCCGCCAGAAGCAGAATCCCGTATTTCTTCCAGAGAATCTCCTAATT
GTTCCAGAGCAACTAGAAAGAGATTCTTTAACCAGAAAGAATTCAGTTCAGATGTAGGAT
ACCTATCCAGAAGTTTTCGCAACTCAATCATGTATGATGGAATCATCAAAGATTTGATCT
TTTCTAACTCTGTCTGTAACTCACTAGAGGCTCGGAAAACAAAGAGAAGATGTGTACGAA
CGAGATATCCAGCAACAAGAAGAAGGAAAAGAATTGAATAGAGGAACTCCCAAGCATTTG
GTGATCTCAGATGTGTCCATATCAATGGAATGGGTGACTCATTATTTCGATGAATCATTT
CTTCGGACAGAAGAAGATTCTGTAAACACTTACTCGAACTCTCACTTATCAGATTCCGTT
GTGGAAGAATCGACCACCACTTTTTCTGAGGAATTGGCCATGATATATCTGATCCATGCA
TAATATCATGAAAAACGGACACAAAATTTTGACTGTCACTTAGGGAATATTGAAAGGGAA
TATTCAATATCAAATAAATATTGTTTTTTAAGGTGAAATAAAGATATTTACACCCCGTCC
AAGT

Supplementary Table S5. 379NW_C_cassia_full length chloroplast genome

```
TTATAGGGATAGCGAATGCTGGGCGAACGACGGGAATTGAACCCGCGCATGGTGGATTCA
CAATCCACTGCCTTGATCCACTTGGCTACATCCGCCCCTCCTCTCTCAAAAGGATTCCAT
TTTCACCATTCATTATTTTTTGATTTAGTCTTTATTACTTCACTCTCCTTCCTGCTGAAA
TACAGATATTGTACATAAAACAAAATGTTGTACGTAAAAAAAAAAAAAAAAGAAAAATGCT
TTGATTTTTTCAAAAAATCAAATTATTTTGAAGAATAAGAGTATATAAAATGCAGGTTGG
TACAGAAGAAACTACGATATTCGATCATGAAATAACCAGCGGTTTTCATAAGTTGAATAA
AAGAAATGAAAATGAAAAACGATTATGTGAATAAAACACTACTGAACCAAATAGATCAAT
ACCAAACTTCTTGTTCTATTAAGAAGTTTGGTATTGATCCTTCAACGACTCGTATACACT
AATACCAAAGTATTATCCGTTTGTAGATGGAACTTCGACAGCAGCTAGGTCTAGAGGGAA
GTTGTGAGCATTACGTTCATGCATAACTTCCATACCAAGGTTAGCACGATTGATGATATC
AGCCCAAGTGTTAATAACACGACCTTGACTGTCAACTACAGATTGGTTGAAATTGAAACC
ATTTAGGTTGAAAGCCATGGTGCTGATACCTAAAGCAGTAAACCAGATACCTACTACAGG
CCAAGCTGCTAGGAAGAAATGTAAGGAACGGGAGTTGTTAAAACTAGCATATTGGAAGAT
CAATCGGCCAAAATAACCATGAGCAGCTACGATATTGTAAGTTTCTTCCTCTTGACCGAA
TCTGTAACCTGCATTAGCAGATTCATTTTCAGTGGTTTCCCTGATCAAACTAGAGGTTAC
CAAGGAACCATGCATAGCACTGAATAGGGAGCCGCCGAATACACCAGCTACGCCTAACAT
GTGAAATGGATGCATAAGGATATTGTGCTCTGCCTGGAATACAATCATGAAGTTGAAAGT
ACCAGATATTCCTAAAGGCATACCATCAGAAAAACTTCCTTGACCAATAGGGTAGATCAA
GAAAACAGCAGTAGCTGCTGCAACAGGAGCTGAATATGCAACAGCAATCCAAGGGCGCAT
ACCCAGACGGAAACTAAGTTCCCATTCACGACCCATGTAACAAGCTACACCAAGTAAGAA
GTGTAGAACAATTAGCTCATAAGGACCACCATTGTATAACCATTCATCAACGGATGCTGC
TTCCCATATTGGGTAAAAATGCAAACCTATAGCTGCAGAAGTGGGAATAATGGCACCGGA
AATAATATTGTTTCCATAAAGTAGAGACCCAGAAACAGGTTCACGAATACCATCAATATC
TACTGGGGGAGCAGCAATGAAGGCAATAATAAATACAGAAGTTGCGGTCAATAAGGTAGG
GATCATCAAAACACCGAACCATCCAATATAAAGACGGTTTTCAGTGCTGGTTATCCAGTT
ACAGAAGCGACCCCATAGGCTTGTGCTTTCGCGTCTCTCTAAAATTGCAGTCATGGTAAA
ATCTTGGTTTATTCAATTCTCAGGGACTCCCAAGCACACAGATTATCTATAAATAGAAAT
AGACAACGGAAGGCTTGTTATTCAACAGTATAACATGACTTATATGTCCGTGTCAACCAA
TAAGAGAGATATCTATCTGGATAGATCCATCCGAACGATTTGTAAATTAAATGAGTAGGG
ATTTATCCAATAACAAATATTTTTTCGTATTTTCCGTACGATTGGTAATGGGTTGCCCGG
GACTCGAACCCGGAACTAGTCGGATGGAGTAGATAATTTCCTTCTTGCAATATAATAGAT
ATAGAGTAAAAAGACCCCCCAAAAAAGCCGTGCTTGCATTTTTCAGTGCACAGGGCTTTA
CCTATGTATACATCCAAAACTAAGTTCCCTAAAAGGGGACCTAAGAAACTTGAAGACTCA
GTTGATTCAACCACTACTGTATGAACATTTCAGAATTCAAATGAATAAAATGATTTTGTG
ATTTTATCTCTTCATCATTTAGGGATCCTTTCTATTTACATGACCTCATGACCAATCATA
AATGACTGACTAGGTCATTGATACGGATAATATCCAAATACCAAATCCGTTCTCTATGTG
ACCTATGAGAAGGAGAAGAGGTTGTTGGGAAGATCAAAGAAAGAGCTTGTTCTTCCTCCG
TAAAGAATTCTTCCAAGAACTCCGAACCTAATCTTTTCAAAAAAGCACGTATCGTACTTT
TATGTTTACGAGACAAAGTTCTAGCACATGAAAGTCGAAGTATATACTTTATACGATACA
AACTCTGTTTTTTTGAGGATCCACTGTGATAATGAGAAAGATTTCTGCATATCCGCCCAA
ATCGATTGAGAATCTCAGAATCTGACAAATCGGCCCGAAACGGCTTACTAATGGGATGCC
CTGATACATTACAAAATTTTGCTTTAGCCAATGATCCAATCAGAGGAATAATTGGGACTA
CGGTCTCGAATTTCTTAATAGCAGTATCTATTCGAAACGAATTCTCTAGCATTTGACTCC
TTATCACCGAAGAGTTTAGTCGTACACTTGAAAGATAGCCCAGAAAATAGAAGGGATGAT
TATATAATTGCTTTATATGGATCCTGGCCGGTTGAGACCACAAGTCAAAATGACATTGCC
AAAAGTTGACAAGGTGAGATTTCCATTTCTTTATCAGAAGACGAGCCCCCCTTGAAGCCA
GAATCGATTTTCCTTGATATCTGACATAATGCATAAAAGGGTCTTTGAACAACCATAGGG
TTTTCTGAAAATCGTTACAAAGCACTACTACAAGATATTCTATTTTTGCATAGAAATGTG
TTCGCTCAAGAAAGGATCCAAAAGATTTTGATCGTAAATGAAAGGGTTGTTTACGGAGAA
AAATGAATATGAATTCACATTCATATACATGAGAATTAGAGAGGAACAAGAAGAATCTTT
GATTCTCTTTTGAAAAAAGGGAAATGGAATTTTTTGGAGTAATGAGACTATTTGAATTCC
AATACTCGTAGAGAGAGAATCGCAATAAATGCAACGAAGGAGTATCTTGTATCCAAGAGT
GAAGGGTTTGAACCAAGATTTCCAGATGGATGGGGTGGGGTATTAGTATATCTGACACAT
GATTTAAATGTGATAACTTGTCCTCGAAAAAGGGAAATATTGAATGAATAGATCGTAAAT
TATGAGATTTTGCTATTTCTTTTTCTTCTAGGGAAGATACCAATCGCAGCGAGAATGGAA
TTTCCACAACGACTGCAAAACCCTCCGATATCATTTGAGAATCAAAATGATTGTTGTGCC
CAACGAATCGATTTTGATTAGAATCATTAACCGAAATAATCAAACGATTCTGTTGATGCA
TTCGAGTAATTAAACGTTTCACAATTAGTGAACTGGATTTATTGTCATGATCTAAATTTT
CCACCGGTTCATAAAGAATCGATCCATTTAAAGCATGACCATGAGCAAGCGCGTAGATAT
ATTCCTGAAATAGAAACGGATATAGGAAGTATTGTTGCCGAAATCCATCCATTTCTAAAT
ATCCTTGTAGTTCCTCCATTTCCATTTGAAATTACACTTGAACCAAATGGGGGATTTCTT
GAGTTATCAAATAATACATAGTACGATACGGTCAGAACAAGGTATATAGTAAGAAAAGAA
TAGATACCCCGGAGCCAGAAAGGACAATCAACGGATCCTATTTCCATCCAATTTATTTAT
```

GTTCGTTATAGTTACAAGAGATGGTTAGAAATCCTTTATTTTTACAACCCGATCACTCTT
TTGACTTTGGAATAATGAATTTTGATCAGTATACCGTTTCTTCTACACATTCGTCTCCAC
TACATAATAGAGAACATAATAGAGAATAGTTAGGATTCATTAAAAAAAAGGAATTGATGA
TCCACTCACAAGAGAACCCTTTCCCACATCAGGCACTAATATATTTTTAACGTCTAATTA
GATCGGGTAATCATTCGAATTAAGAACAAACAGAAGCTCGTTGCTTTTGGTTTCCCTATA
ATTGGAGCTATAGGGCTCTATCCATTTATTCACTCGACCCAACTTGAATTGATTTGACCC
CTTTCCAAGAAAAGAATCAAAACAAGATTTTGTATCGATCCGTTAAGGATGAAGTATTCT
AAGAGTTCTCCATTGATACGACATGCTGTTTTTTCCTTTCATTCCCTTTCAGGATCAGTC
GTGGTCTTACAAACTCCACCAATGGTATGGACGAATCCGTTGCTTCATCAAAATGTGTAA
AAGACCATAGCCGCACTTAAAAGCCGAGTACTCTACCGTTGAGTTAGCAACCCATATAAA
TAGGGTGTGTAGATACGATCGGAATAAAAAATAAATAAAGAGATTCGATTGCCCGACCTC
GTCAAAACATTGAACTAGCAACAGATCAAAAAGAAAGATTTGATGATCAATTGTGAACAT
AAAAATGAACAGAGATCAGATGAAAATACAACAGATTCTGGGATAAATTATAGAGAAAAT
CTAAATAGATGTAAAAATTGATAGACTACCCATACTCTATTTTTTTTTTTTTCATTATA
TTAACTAAGATACTTCTTGTGTCACAACGAAATTGACGAACCCATCGTTTGAGTGAAATA
AAGAAACAAACTTATGAAATGTGGATAAATAGATCTATTTATCCACGATCGAATTATATT
TGTTCGATACACCATTGTCAATATGAATTGAATGTTGAGAAAACCAATTCAATA
AATAAAACAAGGACTTGTGTTGGAGTGACACTACAACATAGATAAGGGATGAAGTATGAG
TGGGGAAATAAATAAGGAATTCCGGTAGGAAAAAAATGTCGGGTTTATTCAATATTTATT
CAATAGAGGTACAATAAGCAAGATTGACCTTTTGTTTGTTGGTGGAGTCCCAACGAAAAC
CATCTGATTGATGGTAATCCCATAATTTCCCAGTTATTTCATCTATTCACTCAATCTTTT
TTCTATCTGTCTCATATCAAGAGAAGATATTTTTTTTTTATAGTTCTATGATACGGGGTC
ATGTGAGAGCAACAATGAATAGAGAATAGAGAAAAAAAATAAGGAATGGTGGAGAAACAA
TTAGACAAAGCTAGATACTGGCCCCCCCCTTTTTTTTTATTTCATTAATTTCATTTGATT
AATTCGAAATTCCTTAAATACCTCCGCCTTCTTTGAAATATCATGAACAGTTCCTGTAGG
TTGAGCGCCTTTTTCAAGGAAATATAGAATAGCGGAAACATTTGAATAAGTTTGGTTCTT
TATCGGATCGTAAAAACCCACTTTACGAAGATCTCTTCCCTCTCTTCGGGATCGAACATC
AATTGCAACGATTCGATAGATGACTCATTGGGATAGACATAAATGAACAACCCCCCCTAG
AAACGTATAAGAGGTTTTCTCCTCGTACGGCTCGAAAAAGAATGATTCGAATTTATGTAT
TGTAGTGGCAAATAGATCCACAAAATCATCAATTAGACTATGATTTGAGTCATTTTTTTT
TGTTCTTCCTTCCTGAAAAAAAAAAAAAAAAGAAATCTCATTCGTACTCATAACTCAAG
TTGGGTAATTCTCAAAGAGCTCGAAGGGAAATCCTTAGACATTTATTGAGCCGTCTCTAA
CCTCTTTTGTTTGTCTCGCCTAGAATCGATTTGATTTCTTCCCCATTCTGATCTAGTTGT
TGAGACAATTGAAAACGGTGTTTCCTTGTTCCGGTATCCTTTATTTTATCTTTGCTTTGA
ATCCTTGGGTTTAGACATTACTTCGGTGATCCTTAATTGTTTCAAAATGGTAGCAACATA
CCTTTTGTTATTTCGTTCTATGGAAACGATTGATTCCCCTGTGATACACTTTTGATCGGA
ATTGGTAAAACTATTTCGACAAATTCATTTTACTTTTTTTTTTTTTACTTGTTCGAACTTG
ATCCTTTCAATTTCTATACCGAAGATATACTTACGAAGTTGTTCCAACTTATTGATTGGC
ATTAACCCTAGATCCTTCTCTCTGCTAAATGAACCAATTCTTTATGCTCGAGCTCCATCA
TGTGCTATATTATAATTATATTATTTTATTACAACCCCAAAATTGGGGTCCTAGTGGAAT
AGAACAAACTATGTCGAGCCGAGAGCATCTTCTTTGATATATAAAATGGTGGGTACAAGA
ATCCACAGCCAATAATGTCCTTCAAGTCGCACGTTGCTTTCTACCACATCGTTTCAAACG
AAGTTTTACCATAACATTCCTCTAATTTTGGACCGGTATGGAATTGATTCAATATGGAAT
CATGAATAGTCATTGGCTCAATCGGTATATAGTATATGAAGTCCTCCATACTTTCATTTT
CATATATGGATCTGGAGAAGTCTCAGCAAGAATATAATTTAACCCCATATTTTATTAGAA
GAATGAAACACATTTATAAAAAACACGAAGAAATGCGTTGCTTAACACTTCTTTACATCT
TCAACAGATTGTTAGGGATGATGAATCATGAAAGATCCAATTCTTTCTCAAACAACTAAA
ACTAAAGAAGGGTCTAGATTACCGATACCGGAACAGAATGAGTCATTAACCAAATAAGCT
ATTCCAATGACCGGGAAAGATCGCAAGTGACTCGATAGGATAGATTCACCAATGTCACAC
TTCTTCGAGGAGAAAGAATTTATAAGGAATCATAAAAAACAATTTCAAGAATTTCCTACT
TCGACATCATTACTGGAAATAAGTTTTCCAGTAAAGACTGAATTGAATCTCTAAATCCAT
CAACAAGTCGGTACAACGAGGATCTAAAAATGTTTAGCAGATATCTGATTAATTAAATCA
GACGCGAATTTGATCATCATAAGAGACCTCTATGTTTCCTTTCCGATTGAATCATCAATA
ATTTAAACCAGATAAATGGGTCAAGAAACAAATCCAATTGTTTTGTTTTCTTGGGGATGG
ATAGAAGGGGCTCATGAAAGAAAAGATTAAGGTCGGTATGGTATTCTTTCATC
TGATTGATAAAATCAGAATCGTAGGAGTCTGATTTTATCGTATTCATCAGATACACCAAA
CAAGTGTTACCGGGGGTAATCGTGGGTATTTAAGGGATCGCAGATCTTTTTCGCCAAAAC
TGAAACACCGGTGTCACTGATCACTGAAATAGAACAATAACAATATATATAGGCATGGTT
CATTTTCTACAGATTTTTCCTTACTTCAGATTCAGGAATCTTTCCATAAAGTAAAGTGAG
TGTATGAATCTCCCCCCTCCCCCAATTGATCGCTACATTGATTTCCAATTATTCATTGGA
GCCAAATCAAATACAAAATAAAAGAAATTAGGTCCAAAGAAAATAATCCGTTCCGTATTG
AATTTTCTTGTTTGTTAATAAGATCCGGATGACAAGGGTCTTGAAATTGATACCTTCCTT
TCCTTTGCGGATTAACTCATATCGACACGAATTCCATGGGGATCATGAATCATACCCAAA
GCCAATTTAAAAGGATCTTCTTATGGGATGCTATCCTGTCTTGTATAAGTACAAAGCAAA
ATGGGTTCATATCAATTCGTTGTTACTATTTTGTTTGATTTTGTCCCACCCCAGTCTCAG
GAGCTTTAATTCCAGCGATGGAATTAATACCAAGAACCCCCCCGTTTTTTAGGATTCAGG

ATCCACATAGAATTAGTCATTTTGTCTACCCTATCTTTATTTATATTTACTTTAGGGAAA
GTAGAGACTTCTCTTTTATTTCGCATTTCGACTCAGAATGCATCATGTGAGAATCCAAAT
ATCATAGATATGGGGCATAAAGTTTATCCAAATGACTAACTAACCTTCAATATGAATATG
GGCGAAGGGGCGAACATACGCTGAATGGCCCACCCAGTTTTTTTTTTTTGAATTTCACTT
TGATCTTTGTTCCCATCCTACCTATATCAAAAAAGATATTTATTTCCATCCACATGTCAT
TGATACTCGATCCGTTTGTTTGTGAGAAACAAAATCGAAAGGGAAGATATGATTCTATAG
AAGAATCATTAGAAATCACAAAGAAAGATTGGATCACATTGTATCCAACATTACACCCTT
AAACAGAAGATTGTTAAAAAGAACAATCTTTGTTATGATAGAATTGGTCTGGGACGGAAG
GATTCGAACCTCCGAGTAACGGGACCAAAACCCGTTGCCTTACCACTTGGCCACGCCCCA
TTTTTATTTCTATTCGGCACCAATAAACACTAATATCGGTATTGGTTGTTCGTCAATTCC
AGCCCCAATATCTATTGAATTGGTTGTTGCTATGATTCTACACATGTAGATGTAGAATCA
AAATGAATTTATTGATCATTACATATAATTCAATTAAGATATTGTATGTAAGGTATGATT
CCTTCTATTCTCATTTGAGAATTGAAGGATTTTTGATTGAGGGAGTTCAAAGAAAAAGAA
AGATTTTGCGGCCTACTTTCCCTTCTTTCTTCATTTTCCCCTTATATCAATAACCCAATA
ATAATGAAATTTTCTCCAAGAACAAAATGTTTGTTATGCTTAATATCTTTAGTTTGATCT
GTCTTAATTCTGCCCTTCATTCGAGTAGCTTTTTCTTCGCCAAATTGCCCGAAGCTTATG
CTTTTTTCAATCCAATCGTAGATGTTATGCCAGTCATACCTGTGCTCTTTTTTCTCTTAG
CCCTTGTTTGGCAAGCTGCTGTAAGTTTTCGATGAGATCTTTAATACTGTCTTAGAAACA
TTCATGATTTATTCGATAAAAAAAAATTCTATTCTTAAGAATTGATAAGATCAGATAATG
AACCCTCGACTCAAACATGGAAATTCTTTTGGATAACCGAGATGAATCGGAATCACCTCA
TTTCTTCATTCCTTCTGGGGGTCGAAGACCCTATGTATGGTCCCTACAATACCTAATTGT
AGGTATGAGAGATCATTTTGTTAACGAAAGAATCAGAATCTTATTACAAATGCATTCCTG
CAAATTTCTTATGTTTTCTAGAAACCGCTTCTTTTCTTGGTGTCAAAACGGAATATGTGG
TACAAAAATGGAGAATCTATTCCCCTATTTCCCCCAAAATGATCTTGGAGATTGTGTAAT
GCTTACTCTCAAACTCTTCGTTTACACAGTAGTGATATTCTTTGTTTCTCTCTTCATCTT
CGGATTCCTATCTAATGATCCAGGACGTAATCCTGGACGTGATGAATAAAAAAATCAGGG
GTTTTTCCTTGCTCGATTTCTGAATTTTCTTAGGATTTTCTTTCTCCATTCCATACATTT
AACTATGAGAAAGGGGGTTAGAGATTTTTTCGAATTCGAAAGGGAAATATCAAGTGATCA
GAAGAAACGGAGAGAGGGGGATTCGAACCCTCGGTACAAATAATTCGTACAACGGATTAG
CAATCCGCCGCTTTAGTCCACTCAGCCATCTCTCCCCATTTTAAATGGATAATTCATATG
TGACGCGTGAAGTAAAGGTTGATAAAAGTTTTTCCTTATCTTTCTTTATTCTATAAATAT
AGACGAATTTGATCAATCATCAATTCCCTTTAGATAATGATTCGAAACAGATATCTCCAA
TAGAAAGAGTACCTCTTTGATTTCGTCCGAAAAGTTCTTTCTTTTATTCCCCCGGCCTGG
CCAGTACCTAGCCAGGCCATTCCTTGTTCCAATGAATCATAGATCAAATGATTTATTTGA
TTTGAAAACGAAAATGCTTGTTATTGAAGCAGCAACAAGGCTATTTCCATTCCTATGATA
GGAGTGTCATTTGTTATTATGTTTTTCCTTTTCTCGATTTACTTAAATGGAATAAAAAAA
ACATATTTTTTTCTATTATAATAGAACTCATATATTTCTTCAAAGAACATGTTTGAACCT
GAACCCTTGAGTCCACAACCAAAACAATAGGATTTTTCACTCGATCCAATCGACCCGAAT
TCATAAGATTTGGCAGTTGAATGAATAGGAAAAGGAGTAGCTTCGAAAAAGAAAAATGGA
GCTCTGGATTCTTGTACAACTCAACTCATTTTTATGTTCCGACTTCAATGGCTCTTTCGG
GCCGGGACTATCAGTAACGGCTCCCCGATAAAAGCTTGTTATTGAAATGAACCTCCTTCT
CCTATTTTATCAAGTCTCCCCGTCAGAGCACAACATGTCAGCACCCCAATTTTCATGATT
CTGATCCTATCTTGATTACGTTTCACGCCCTTGTTCGACAAATGGCCCGCTCGTATACAA
TAATTATATTGTAGCGGGTATAGTTTAGTGGTAAAAGTGTGATTCGTTCTATTAACAAGT
GAAATAGATAAGGGATCTTTCGTTTGATTCCTATTCTGATCAAAAACTTTATTTATTAAA
AGGGCATTAATCCCTTACCTCTCAATGCCACATTTGAGGAAGAATAGACATTCTCGTGAT
TTGTATCCAAAAGTCAAGTCAATTAGAAATTGACTAACAAAATTGGATTATGGAATTGCG
AAGCATAATTTTTTTTTTTTGAAGTTGGATCAACCATTCCAATTGAATGAGTATAAGTAA
GGGATCCATGTATGAAGATACAAAAGTCTATTTCTAATCGTAACTAGATCTTCCATTTTT
TTTTTTTAGGGGGAGATTGAAGCCAAATAGCTATTAAACGATGACTTTGGTTTACTAGAG
CCATCGACATATTGTTTCAGCTCGGTGGAACAAAAAAATTCTTTTCTTCAGGATTCTTGC
AAGTACAAATAGGGAACGAAGTAACTAGAAAGATTTGTGAGAATCCTCCTCTTTCTAGAG
GGATCATCTAAAAAGCAAGTCATTTGGGGTGCATTCAGACGAAAAGGCTGACATAGATGT
TATGGGCCAAATTGATTTCTTTGAATTCAGATTTGCTATGACTCCCTTTTCCCATACATC
GTAAATTTTTTAGTTTTTTTTATGTCTTAGATTTGGGAATCCCATAAAGGAGCCGAATGA
AACCAAAATTTCATGTTCGGTTTTGAATTAGAGACGTTAAAAATGATGAATCGACGTCTA
CTATAACCCCTAGCCTTCCAAGCTAACGATGCGGGTTCGATTCCCGCTATCCGCTTCATA
TTAATTATTATAATACATGCTTTTGATATGTCCCTAAAATCTTTCTTTCACATACAATCC
TATTCCTTTTTTTTATAGGAGATAGGAAAGTCAGAACGTGAAAGAAATCGGAATGAAAAG
CGTCCATTGTCTAATGGATAGGACAGAGGTCTTCTAAACCTTTGGTATAGGTTCAAATCC
TATTGGACGCAATTTATTTCCATCTATTTTTGTAGATTGCTATGTCAAGAAACATATTTT
GAATGATTCGAATCGGGGACATTTCTCAACGATTCGTCTTGTACTTAAGAGTGATCAATT
TCTTTATGTTTGTTCCTGAAGTAGAAACAGTTCTATCTGTTCCGGAATAGCTTCCTTCAA
AAGGGCTTCCGCTTGCGCGGTAAATGTCTTGGTAGAAGATATGATTTCTTGGACCTGAGG
TTTATTTGTTTTTAAGTAGGTACGTAACTGAACGAGAAATTTCTTTACCTGTCCAATTTC
TAACGGATCAAGATACCCATTCGCTCCGGTATAAATAGTCACTATATGTTCTTCCACCGT

GAGAGGGGCTGATTGGGATTGTTTGAGCAACTCACGTAATCGTTGACCTCTTGCCAATTG
ACTCTGAGTGGCTTTATCGAGATCAGAAGCGATTTGTGCAAAGGCTTCTAACTCTGCGAA
TTGAGCCAGTTCCAATTTTGATTTGCCAGCTACTTGTTTCATGGCTTTCATTTGAGCTGC
AGATCCTACTCTCGAGACAGAAATACCCACATTAATGGCAGGACGGATTCCGGCATTGAA
TAGATCAGCGGATAAGAATATTTGTCCATCTGTAATGGAAATCACATTAGTAGGGATATA
AGCCGAAACGTCCCCAGATTGAGTCTCAACTATTGGTAAAGCGGTCATACTTCCTTCACC
TAAACGAGAACTTGATTTAGCGGCTCTTTCCAAAAGGCGTGAATGCAAATAAAAAACATC
TCCTGGATAAGCTTCGCGGCCGGGCGGTCTTCTTAATAGAAGAGACATTTGACGATAAGC
TTGTGCTTGTTTGGAGAGATCATCATAAATTATTGAAGTATGTCGTTCACGGTACATAAA
ATATTCAGCCAGAGCCGCTCCTGTATAAGGAGCGAGGTATTGTAATGTAGCAGGTGAATC
CGCCGTTTCGGCTACCACAATAGTGTATTCCATTGCCCCTCGTTCCTGGAAAGTGGTCAC
TACCTGGGCCACGGAGGATGCTTTTTGACCAATAGCTACATAAACACATATTACATTTTG
CCCCTTTTGGTTGAGAATAGTATCTGTGGCTACTGCTGTTTTTCCGGTCTGTCTGTCCCC
AATAATTAATTCTCGCTGACCGCGTCCTATAGGGATCATCGAATCAATAGCAATAAGTCC
CGTTTGAAGAGGCTCATATACGGAACGTCTCGAAATAATACCTGGAGCAGGAGATTCGAT
TAACCGAGATTCAGAAGCTGAAATTTCACCTCTCCCATCAATAGGTTTAGCCAGAGCATT
TATAACACGACCCAAATAAGCCTCACTCACTGGTATCTGAGCAATTCTTCCTGTTGCTTT
TACAGAACTTCCCTCTTGTATCATCAAACCATCACCCATTAATACAACGCCAACATTATT
TGATTCCAAATTCAGAGCAATGCCTATTGTACCCTCTTCAAATTCTACTAATTCCCCTGC
CATTACTTCATCAAGACCATGAATACGAGCAATGCCGTCGCCTACCTGAAGTACTGTGCC
GGTATTCACAATCTTGACTTCTCTATTATATTGTTCAATACGTTCACGGATAATATTACT
AATTTCGTCGGCTCGAATGGTTACCATTAGTGTCTCTTAATTCTTTTTCGGAAACAAAGA
AAAAAAAAAAAAAAAAAATAATGCCTACAGTAGAAGGGCTAATCAGTTACTTCTTTCATG
GCCCCGAGCATGCCAATATTAGCACCGATGGTGCGTAAATGTAACTCGCTGTTTGAACAA
CTATTCAGAGTTCCTAGAGCTCCTTGTAAGGCTTGTTGGAAAACTCGTTGTCGCACCTGA
TTAATTGCTCTTTGTTGTTCAAAATGAATGGTTTCATTTTTGTAATTTTCTAATCGTTCC
AAATTCTCATAAGTGGCATTAATCAAATTCCATTTTTCTCGTTCTATCTCAGAGTATCCA
TTCACTCGAAACTCATCTGCTTCTATTTCCACTTTCCGTAAGCGAGCCCGGGCTTCTTCG
AGCTGCTCAATGGCTCCTTCACGTAGTTCTTCTGAATTTCGAATAGTACTCAAGATCCTC
TGTTTTCGATTATCTAATAAATCACTTAATGAAAGTAGATTATCTTCCCATTCATTTCAC
AACTTCATTTCCATGATCTCTTCCCGAACCAAACATGAATCTTTCGATTCATTTGGCTCT
CACACTCAGTTACTTAATGGGTCATTCCATCTATTTTAATGTAATGAGCCTACCCTCTCT
TCTCTGTTCGTATTCCAAGATATCAAAACTGATACGAGACCAGAATATTCGGAGGACTCT
TCCGACCCGACAAAAAATCTGTCATTGTCAGCAAAGTTGTTTCTTTTTTTTTTTGTTTTC
TTCAAATCCAAAAAATTCTTCTTATTTTAGACATAGGTCATCGATTCAACATTGGATAAA
AAAGGGCGAGACACCTATTTTTACAGTAAATGGTTCAAATCATTTTATCGATATGAGTGT
TCTATATCGGATAAATTGCCAACTATTCATTTTTTCGAAACCATCTCCGTACTAACGTAG
TGGTAGAAAGAGTACCATGTTGTGCCTGGACTTCAAACGGTTTCGCTTTAACCATGTTAA
AGGTCCCACATTATTGGCTGATAGAGAATCAAAGTTGATTTACCAATAAATTACGAAATG
CTATGGTTCTTACATATGATTTCTTAATTTATTCAGAAGTAATTCGTCGAGATCGTGCAC
CTTTCTTTCCTATTTATAACTTTCCCATTCAAAAAAAAAAAAAAAGGAAAGTGCAGCCGGT
TGGATCCAGCCTATTCTTGAAATACACAACTCGCACACACTCCCTTTCCAAAAAAGATCA
ATACACCAAGCACTACACTTAGATTTATTAGATTTGTTGCTAAAATATCGGTATTAAACC
CGAAACTCCCAGCGGATGGCCAGTGACCAAAGGAAACGAAAGAATCGGTTACATCTCTCA
TATGCTTTTCTCTTATAGATAGGACCAACAAAATGGAACAGAGTTCTTTTTGTATCACTT
CGCCCCCTTTGTTTTGGATTGATTTCTTTTTATTAATTTCCTTATTATAAATATGAATAG
ATTCATTTTAAGAAATATTTTTCTTTATTATTATTTCCATGGAAATTCTCAATAATCTAT
TTATTTAGTCCCAGGTTTCATGTCAATTACGAAATACCTCGTTTGTTGCAACACTTCCTA
AAAGTAAAAAAGAGTTTCCATTAAGAACGGAAGGGAAGAAAGCAAGTGGGTCTGCTATGC
TAATTCCTCATCCTCAAATCACTCCATCCCCGGGGGTATTGTCTCAACGAAGAAGTGATT
GTAGGAGTGAAGTTTGGATATAATTCGGCAAGGCAAGCCCGCGGCAATAAAATAGGAAAA
GAAAATAAGTATTTATTTTTCACATTTATAGGATTAAACAAAAGGATTCGCAAATAAAAG
CGCTAATGCCACGACCAGTCCGTAAATTGTTAAAGCTTCCATAAAAGCCAGACTAAGCAA
TAAAGTACCTCGTATTTTACCCTCTGCTTCTGGCTGTCTCGCGATACCTTCTACGGCTTG
GCCCGCAGCAGTACCTTGACCAACTCCAGGTCCAATAGAAGCAAGTCCCACAGCCAATCC
AGCAGCAATAACGGAAGCGGCAGAAATCAATGGATTCATATTAAGTTCCTCGCACCAAAA
AAAAGAAATGGTTAATGATACAATCAACCGATGAATTATTACTTCATTATTCCATCACTT
AAGATCTATCCGAAAAAAAAAAAAGAACTAAGAACTCTGAATTGAAATAATAATATTACTG
AATCATCAGAGCTACTTCGATATCTCGTTTTTAGTTCCTATCCGTGGAGTCTTTGTAAAT
CTATACGGTTCCAGTTCTTCCATTTCTTTGTTCCGAACCATTCCATTCTTTCAATTCTTC
GCTCTTTCTCTTCTATATGCATGCTTGACTTCATTTGTTTATTCATTCAATCCACAGTCA
CAGATAAAACGGAAGGGCTTGCATTGGAATCCGTCTAAATTCAGTGGGATGGGAAATAAT
ATATATGGATAGCCCATATATAACTAGTGAATATCTAATATCACATATACATTGTTTCTT
TAATAACGTAAACCATCCGTATCTTCTATTGAACCGGATTCTAGAATCATTCTTCGAAAC
ATATACAGGGATTGGCTTAGAGCCCTTACATATACCCAGCTCGACCCCCCTTACTTTTTT
TTAATTCTCTAATATCATACATTTTTTTTTTGCTCCTATTCTAGATCGTATATACCGGTA

TGAGTTGGGATAAGCTTTTTTTTAAGACCATTTCGGAAGCTAGTCAATGATGACCCTCCA
TGGATTCACCTATATAAGCTGCGGCTAAAGTTGCAAAAATAAGAGCCTGAATCCCGCTTG
TGAATAATCCAAGGAACATGACAGGTATAGGAACCACCGAAGGTACTAAAGAAACAAGAA
CAACAACTACTAATTCATCCGCTAATATATTCCCGAAAAGTCGAAAACTAAGTGATAAGG
GTTTTGTGAAATCTTCTAGAATGTTAATTGGTAAAAGTATTGGAGTTGGTTGAATGTATT
TACCGAAATAACCCAATCCTTTTTTGGTAAGACCCGCATAGAAATATGCCACTGACGTAG
GTAAAGCTAAAGCAACAGTAGTATTTATATCATTCGTGGGTGCAGCTAACTCTCCATGCG
GTAACTGTATGATTTTCCGGGGTAAAAGGGCACCTGACCAGTTAGAAACAAAAATAAATA
GGAACATAGTTCCAATAAAGGGAACCCAAGGACCATATTCTTCTCCAATCTGAGTTTTGC
TCAAGTCTCGAATGAATTCGAGGACATATTCGAAGAAATTCTGACCGTCGGTTGGAATGG
TTTGTGGATTCCGAACAGCTATAGTGGCTGAACCTAATAAGATAGCAATTACAACCCAAG
AAGTGATAAGTACTTGGGCATGGACTTGGAAACCCCCTATTTGCCAATAAAAATGTTGGC
CTACTTCCACATCGGATATATCGTATAACGCTTTTAGTGAGTTGATGGAACAGGGTAAAA
CATTCATATTGCCCTCTGACATAAATAGAACTTAAAAAGGAATTATTTTGATTCAGCCAT
CTCGTATCTCTTTCTCAACTCGTCTACTTTGAATCAATCGTATATTTCGGATCCCAAGTG
ATCACATAATATCCCCAGTGATTTTGATCTCTTTTTTGAGACTCATGGATAGTAACCGAT
TCAATCAATTTATGGAGTTTCCAAAGTGATTGACTTATCCTAATCAACAATTTCTTATAT
AGCTAGAACTGCCCTCACAAATTGCGGATACTAATTTGTTAAGAATCCATCGGATTGAAG
CCATAGCGTCATCGTTCGCTGGAATCGAAATATTTGCGAGATCCGGGTCACAATTTGTAT
CGATTAAACAAATTGTTGGAATCCTCAAAGTGAGACATTCTCGAAGAGCCGTATATTCTT
CTTGCTGACCAACGATGATTACAATATCGGGTAACCCCGTCATATATTTGATCCCGCCCA
GATAGGTTTGCAAGTGAGATAATTGCCTCTTCAACATTGCTACATCTCTTTTCGGGAGAC
AGTTGAGTTTCCCCGTATTTTGTTCCGATCTCAAGTTCCTGAACCTATGAAGTCTCATTT
CTGTAGTGGACCAATTCGTTAACATACCACCGAGCCATTTTTTATTAACATAATGACACC
GAGCCCTTATTGCAGCTGATGCTACTAAATTGGCTGCTTTATTTTTGGTACCAACTATTA
AGAAGTGTTTTCCTATACTTGCTGCATCAAAAACTAAATCACAGGCTTCTGACAAAAAAC
GAGCAGTTCGAGTAAGATTTGTAATATGAATACCTTTACGCTTTGAAGAGATGTAAGGTG
CCATTCTAGGATTCCATTTCCTAGTACCATGGCCAAAATGAACTCCTGCTTTCACCATCT
CTTCAAAATTAATGTTCCAATATCTTCTTGTCATTTCTCCCCACATTTTCTCTCTTTTTT
TTTATTTAAGAGGTACCCCTGAAATAAATAATTGTTCCGACGGAACCTTCTACCGGAGAT
TGACCGTTAATACCCAGTCCAAGTCATTAATTCCTTTCTATTCGTTATTATCTTTATTAC
CAAATCAAATGACCAGGACCCTATAGTTAAAAGAAAAGAATGAATCTGTCATTAAATCCC
GTAAATGATCGTTCTGATGTATCAGGGAAATTATTTGGGATGCAAGAACCAAATAATTCT
CTGTGGTGGAACAAAAGATCTCTCATTTCCTCCTCGAATGGATTCTTCTTTTTGATTTCC
CAAGGAATGTTGCTGTGTTGCCTTGAGCGGTGCACTAATCCTTTGAATCCGGTACCAACA
GGCATCATCCCCCCCAGAACAACGTTTTCTTTCAGGCCTTTCAACCAATCAATACGACCT
CGTAGAGCAGCTTTTGCTAAAACTCGAGCGGTTTCTTGAAAACTCGCTTCGGATATGAAA
CTTTGAGTATTCAGAGATGCCCTCGTTATTCCCAATAAGATGGCTCGGTAACAGATCGCT
TCTTCCAAAGCACGCCCTGTTCGTTCCGCTCGCAACAATCCGATTAGTTCTCCAGGTGAA
AAAACATTAGACATTCCATCTTCTGAAACCAACACTTTTGATGTTATTTGACGTACAATA
ATCTCTATATGCCTATTATGGATCTGCACCCCTTGGGATCGATAAACCTTTTGGATCTTA
TTAACCAAAGAGATACGACTTTGCGCTATGGTTAGTTCAGCGCCAATCAAGAATCCCCAA
GGAATTCCAAGGATTCTTGTTATACGTTCGTTCCAACCCTCAACCCTCTTTTCTAAGTTC
ATCGATATTGAATCAATCGAACGCGCTTCTAACACTTGTTCCACTTTTGGAAGACCTTGT
GTTATATCACCCGATCTCGATTTTTCATATATAAATGTAACTAATGTATCTCCTTCGTAA
AGGATTTCTCCACAATGGCCATGAACAGTTGCCCCTGGCGTAGCCAAATGGGGCTTAGCG
GATCTTATGACTAAGGAGTCAACATGAACAATTAGAACTTGACTCGATTTTATGTGTGGT
CCGCATTTGGATATACATACATTTTCACAAATAAACTGTCCAAGGCTAATTATTGTGGAT
GTCTCCTCACAATAATCGTGAGGGAGAAAGCACCAATTCAAATCGAATGGATTAAAAATG
ATGTTACTGCATGAATCGGGATTATAAATTCTCCCATTTTCATCCATTAAATAATATTTA
AGCCCTTGGAAAGTGTGTTTGAAATTGTCAAGTAGAAAATATTTCTTTAACAAGATCTGA
TTATGAGTTATTAAATAGAAATAGTAAAATGAATAAAAATTCGTAATTTGAGGTACAATC
CCTAAAGGACCCAACGAATTCCTAATGGGAATCGCGGGATCCTCTTTAATTAATTTAATT
AATTCTTTGTGAGATTTCGAACCATTGAATAGACCAATTCGAGAACAATCGGATGATAAC
AAAATTAGAAAAGATGGACATTCCTTATTTCTATTCAGCAACGTACGAATAGTCCCTTGA
TGTTGGGTAAATGATTGAATCCTCGCCTTGAAATAAAAAGGATTAATATTGGTGCGATCT
GATCCATTATTGGCAATCAATCCTGAACTTGCCATATCATTCCTTTTTCCGATATAAAAA
ATAGAGGACTTCACTAAATCAATTCTTATGAAATCTCGAATCAGATCATTTGCCCTTACT
TCAACAAAGGAAGCATGAACCTCTTCTATAGAACCATCTCGGTCTTGGTTCCAATTCAAT
ACTAAACAAGTCCGAACTAATTGAAGACTTGTGTGATAAATTCCCCGAATCGGTTTGCCA
TTTCCATAAAGGATATAATTGACAATTCGTAGTTGCACATTATCCCTTTCCTGCAACGGA
TCCTGGGGGAAAAGCGTTGCTAAATTTATCCCATCAGCTATTTCATATGTGACTACGGGT
CGAACTGAAACAAAATACTTTTTCTTGGTAGGTGTAATCCGTTGGACATAAATCCAATTT
TTCAATTTTTTTGATTCCTTAGAATTTTTTTTTCCCGTTCCTGGTGGTATCAAGATGCCG
CAGTGCCGGGATATCTTATCTGTCGCTCCAGGAAAATAGATATCCCCAGAAAAGATTTTG
AGTTCAATCCTTTTTTTTTTTCTCTCCACCCGGACCAATCCGCCTACTTGGCTTCTTGTA

TTTAAAGCGATTCGTGTATCTACTCCAATGATACTGTTGTTCCGTACCATTATGGGCGAA
GAACCGGGTAAGATATGCACTTCTTCGGGAATGAAAAAAAATCGATCTACTTTCATTTGG
TATTTTGGCCTAAATTCTTTTGCTCTTCGATACTCAATCAAATCCTCTTTTTTTACGATT
GAACCCACCCCTATAGTCCCATATTTAGTAATTCCTGAACTGCTTCTTCTGTATAGGGGA
TCGTCGAAATAAGCAAGAATACTATTTCTAGGTAAAACACCATTTATGGGTATTTCAATC
GAGATACCGGAACGGGGCATTAGTTCTTTTTCTCGTTCTTGATCAGATTGGAATGGGATG
ATAAATCTATTTCTTCGCCTCTTAGCCAATAAATCAGAATTCTCGTGGAGAATGGTAGGA
TATATGAAATTCCAATGACCGTTGGATATGATTCGATCAGGTCCTGAATAATCAAGAACC
CCCCCCTTTTTACCGTAAGGATTCGAACTAAACAATTTGTGTCTCACTTGATCATTAGTC
ACGGAGAGGTCAGAAATAGATCTCCGTTCAACAGAATGAACATTCATTTGATCTTGATCC
TTGTGGAGCGAAAAAGGCACTATACTGGATCTGCACAGAGCTCCTGATAATATCCATAAA
TGACTTGTTTTGGGTAAGAGATGAACATTACCATATTTATATTCAGGTGCATGGTACACA
TCGGTACTCCAGTGCATTTCTCCCTCTGAGTCAGAATAAATATGTTTTCGAACTTTCTCT
TTAACTTTATTAAAATTGAAAGTGGATGTTCCAGCGCGAATCTCAGCAATCACTTGTTCT
GATTCTACATATTGATCATTTTGAACTAAAAGAAAACTTTTGGGTGGAATATTCACATTA
TGTAGAATATCGTGACTCTCAATAGTTACATACAGGTCTATATAACATAGAAAAGCAGGA
TGCCCATGACGTGTACGTGTGGGATGAACCAAATCCTCATTGAATTTGATTTTTCCCTTA
AAAGGAGCTCGTACATGTTCTGCAGTACCACCTGTGAATACTCCACCGGTATGAAAAGTT
CTTAATGTTAGTTGAGTCCCCGGTTCGCCGATTGATTGACCCGCAATAATACCTACTGCT
TCTCCTAATTCGACCAGGTCGCCATGAGTGGGACTCTGACCATAACATAATCGACAGATC
CAAGATATACTCCTGCAAAGAAAGGGGGTTCGAATATATATTGGTTGTGTTCGAAAGGTT
ATGAATCGAGTGACAAGTCCAACCCCAATATCTTTATTTTGAGCGGCAATGCAACGTGGA
CCCATATATATATTGTATGCTAATACACGACCAATTAGTGTTTGGACCCAAATTCTTTCC
GTCATCCCATTTCTAGGACTCACGGAAATGCCTCGGGTAGTGCCACAATCTGTTCTACGT
ACAACAATGTGTTGAACTACTTCAACAAGTCTACGCGTGAGGTATCCAGCATCTGATGTT
CGTACAGCAGTATCCACAACTCCTTTGCGGGCTCCGTAGCAGGAAATGATATATTCCGTT
AAAGAAAGTCCTTCGCGTAAATTGCTTTGAATCGGTAAATCAATCATTTGTCCTTGGGGA
TCCGACATTAATCCTCTCATACCTACTAATTGGTGTACCTGAGATGCATTTCCTCTAGCT
CCCGAAAAGGACATTATATGGACTGAATTAGAAGGATCAGTCATCCTAAAATTAGGATGC
ATTTCTTGTCTCAAATATTCACTTGTAGCATACCATATCTCAATGGATTGGCGTAATTTT
TCTACTGTGTGTACATTCCCATAATGATGGTGCTTTTCTAAAATGAAACTTTGTTGTTCA
GCATCTTGGACTAACCACCCCTTAGAAGGTACTGTTAAAAGATCATCAATTCCTAATGAA
ATGGATGTAGCAGTGGCTTGCTGGAAACCCAGAGTCTTTACTTGATCCAGGGTGTGCGAT
GTATATGCCATTCCGAAGTGATCTATTAATCTGCTAATAAGTCGTTTCATGGCAGACCCA
TCTATCGCTTTATTGTAAAAGAACAGATCAGCCCATTCTGCCATAAGTACTTCTCTATTC
CGCTGAGTAGGATTCGCCAATGGGTTTGAGTCAGTGATTCGAAGACCTCCTTTACTGGAT
CTCGATTCATGTAGAAATTAAGGAATTATGATTCCAGTTGAACCGGAGAGATCCAAATTC
CCGCGGTATTACATAATTCCTTAGCTTAGGTACCGTATGAGTAGGCCTGACAAAACCCCT
GTATGGCTTCTTCTATTTCTCGAGAAAAAGAAATAGGACCAACAGTGGTTCGGGTGTATA
TACAAAGGGTTTGTTTTTTTATACGTCTTACTATTAGATAGTGCCCATAAATTTCATGAT
AGGTACCCAAAGATTCATATTGAACTTCGACAGGAACTTCTCTTGAGGCAATGACACGTT
GATCTAGTCGCCACCGAAGCCACAAAGGACTATCTAAATTGATTCGTTTCTGCTGATAAA
CTATAAGTACATCATAGGAACTACAAAAATAGGGCTCTTTCTCTTTCGTAGTATATTTAT
ACTTATAATCATTAACTGTTTCATTTTGATAGTTTATGCGATTCCATGGATTATACCTAT
TTGCACAAATACCTCGACGATTCCCGATCGTTAATACATAGAGTCCAATAAGCATATCTT
GGGTTGGTACCGAAATGGGATCTCCAATAGCCGGAGAAAAGAGATTCATATGAGAAAACA
TAAGTAAACGAGCCTCCGCTTGCGCTTCCAAAGATAAAGGTACATGAACAGCCATTTGAT
CCCCATCAAAGTCTGCATTGAATCCTTTACGAACTAATGGATGTAAACAAATAGCACGTC
CACCCCCTAAAATGGGCTGGAACGCCTGTATGCCTAATCTATGCAGGGTGGGCGCTCTAT
TCAACAATACAGGATGCCCCTGCATAACTTCTTGAAGTATTTCCCATACAATGGGTTCTT
TTTCCCGAATTTGACTTTTAGCAATTCCTATGTTAGAAGCGATATGTCGTCTGATTAAAC
CACGAATGACAAATGTCTGAAAAAGTTCTATTGCTATTTCTCGAGGTAATCCACATCGAT
GTAATGAAAGCGAAGGGCCCACGACAATGACGGAACGCCCCGAATAATCGACCCGTTTAC
CAAGTAGAGTCTCACGAAATCTTCCTTCTTTGCCTTCAATTACATCTGAAAATGACTTGT
AAACTTTATTATGACCGTCTCTCATTGGTTGTCCGCGGATCCCATTATCAAGAAGTGTAT
CCACGGCTTCTTGTACCAATTTCTCCTGACACATTACTAATTCCCCTGGCGTAGATCTAC
TTGTTGTTAATGAATCGGTAAGAGTATTGTTCCGATAGATAACTCTTCGGTAGAGTTCAT
TAATATCCGAACTCATTAGTTTACCCCCATCTATCTGAATGATTGGCCTCAACTCGGGAG
GAAGAACTGGTAATAGGCACAAAACCATCCGTTCTGGTTCTACATTTGTTCGAATAAAAT
GCTTAGCTAATTCCATGCGTCTAACCAAAAAATCCTTTCTTCTTCCAATTTTTCTATCTT
CCCATTCATTCCCAGTGAACCCTTCTTCCCCTAATTCCTTCCATTCTACCGATGAATGAT
CTATAATAATTCGCAAATCCGGATCGGCTAATTGTTCTCTGATAGCACCTGCTCCAGTAG
AGATTTCTCGATTTCGAAATGTATCGAAGCCTGGGGTAGTAAAAAAAAGTGGGATGCTGT
ATTTCCGGGATTGGATTTCAGATTCGAATGAACCTCGTAATCGCAAGAAAGTCGGTTTTT
TAGCTATGGGCCTGGCAAAAGAAAAATTGGGATAGGTTCCTATAGGATCCCCCCTTCAAA
ATCGGACGTGATGGTTTCCTCTCATCCGGCTCAAGTAGTTACACCAAATAAAGAAAGGGG

TTCTCCACTTTCAAATTTTGTTCTAGAAAATCCCAGAAAGATCTACTCCTTACTCAAGTT
CCCAGTGAGGACCAACAAAAATATTTCATGGATTCATCCTTCCTTTGACTTTTCTGAATT
ACTTATTCAATTACGATAAAAATGGAATGTCAAATTATTGAGTAGTCTACTTCCCTTCGA
ATGATAAATCCCCTTAAATGAAAGGAATACCTTGGAATTAATAAGGGATTTACTTGTCTA
TGTATTGTTTCATTCGATCTTTTAGGTCTCCACTTCACCTCGACGGTTATGCCATAATGT
CCCTTGAAGCATATATGCGATGGATGGGCTCCTGTAACCGTGTCATATTTGTTTATTTGA
ACAGAATCTCTTTCTAAAAGAAATAGAACGTCTAATTCCACGAAAGAAGTTTTTTTTTT
TCACGAGGTACAACTGTTATATGTTACGGAATCGACCATGGATCAATTCCCCTTTCATTT
GGAAGTATTGAATACAACCATAATTCTGAGCTTCATATTCCTCCTCCCAAGATACATGTC
AGAGTCAGGGGCATCCCCATCGAATTGAATGGGATGACAGTTTCTCATTCCGAATCTGTA
AAATCCTAATTTCGATCAAATCACACATCGCAGTATACTAGGCCTTCTAATTCCTTAAGG
GGTTTATCTAAAAGATTCGCGATATAACTAGGAAGACGTTTCAAATACCATACATGAGTC
GCTGGACATGCCAGTTTGATGTATCCCATTTGATATCTTCGTATCCGAGAATCAACAAAT
TCAACTCCGCATTGTTCACAAAATTTCGGATCTTCTTTTTCCTCTCCGATCACTCGATAA
TTTCCACAAGCACAAATTCCGCTTTTTATAGGTCCAAAAATTCTTTCACAAAACAATCCA
TCCTTTTCCGGTTTATTGCTTTTGTAATGAAAAGTATAGGGTTTTGTCACCTCTCCAACC
ATCTCTCCATTAGGTAGGATTTTGGTGGCCCAAGCACTTATTTGTTGAGGAGAAACTGAT
CCAACTCGAAGTTGTTGATGTTTATATCGGTCGATCATAGAAGAAAAATTCTGATTCATT
TCGATCAAACTTCCTTCCTGTTAATCTGGAAGTTCTTCTCAGATACAAGGAAATGATTCA
GTTCCAGAGCCAAAGATCGTAGTTCTCGAACGAGCAATCGAAAAGATTCTGGCGCATCCT
CGGGTTTAAGTATTGTTCCGCCAATGATTGTAGTTCCAAGTACTTCCTGACGAGCTCTAA
TATGATCCGATTTATAAGTAAGCATCTCTTGTGAAATATGAGCAACACCAAATCCCTCTA
GAGCCCAAACTTCCATTTCTCCTACTCGTTGTCCCCCTTGCTTGGCCCTTCCCCTAAGGG
GTTGTTGTGTAACAAGTGCATAATGCCCGCTGGAACGTCCATGGATTTTATCATCAACTT
GATGAATTAATTTCAGGATATAGGACTTTCCTATTATAACCGGTTGTTCAAAAGGATCTC
CTGTTCTTCCATCCAATATTCTGCTTTTTCCCGGATACTCGGGTTCAAATACCCACGGAT
TTGCTGTTTGCTTACTGGCTGAATATAATTCAGGAAACACTAGCTTTCTCGAAGCCCCCT
GCTCATATCTCTCATCAAAGGGTGCTATTCTATAATGTCTGTCCAACAGGTACCCCGCTA
AACCGAGCGAACATTCAAATATTTGTCCCACATTCATTCGCGAAGGTACTCCTAATGGGT
TGAAGACCATATCGACCGGTGTTCCAGCTTGCAAATAAGGCATATCCTGTCTAGGCAAAA
TTTTGGAAACGATACCCTTATTCCCATGTCTTCCAGCTATTTTATCACCTACTTTGATTT
CACGTTTCTGTAAAATATATACACGAATCGTTTCTGGATTATAACTGGAACCCCCCTTTT
TCTGGATCCATCTCACATCAATAACTCGACCCCTTCCGCCTATAGGTAGTTTTAGACAAG
TTTCCTTTGCGGTGGATACCTGAATGCCAAGTATGGCTCGTAATAATCTATCTTCCGGGG
CATACGATGATTCTTTCGCCGCCTGAGGCGTTAATTTACCCACTAAAATATCACCTCTTT
CTATCCAAGATCCCAGCATTACAATTCCATTTTTGTCTAAATTGCGGAGTAAATGGGCTT
CTAAATGCGGTATTTTATTAGTGATTCTTTCGGGGCCTTGGCTTGTCACATGAGTCTGAA
TTTCATATTTCCGTATGTGAAAAGACGTATAAATATCTCCATATACCAGACGTTCGCTAA
TGAGTACCGCGTCTTCAGAATTGTAGCCTTCCCATGACATATGAGCTACTAATACATTTT
TTCCCAAAGTAAGTTCACCGCCAACTGTAGCCGCGCCGTCCGCTAAAATTTGTCCCTTTT
TAATGCATTTACCCCGCGGAGCCTGAGGTTTTTGATGCATACAAGTATTTTTGTTGGAAC
GTTGATACATAACCAACGGAATGCTTATAGTGTCTCCATTACCCGATAAAACGATCTTGT
CCGTATCGGTATAAATGATCTTTCCCTCGTGTTCGGCTATAGCCGAAACCCCCGAATCTA
GAGCCGCTTGGCATTCCAACCCAGTTCCAACAATGCACTTCTCGGATCGAGAAAGCGGAA
CTGCTTGACGCTGCATATTAGAACTCATTAAAGCCCGATTTGCATCATTATGCTCGATAA
AGGGAATGAGGGAAGCTCCAATAGAAAAATATTGGAAGGGAAAAATGCTTCGAAGATGAA
TCTGTTCCCATGCAATAGTCAAGAATTCTTGACGGTATCCGGCTGGAACAACCTGTTCTT
CCTGAATACCCCGATTCAAAGCCAAAGAATTTCCTGCCGCTACCATATAGTATTCATCTC
TACTTGGTGATAAATAAACCATCTTTTTTGATCTCTCAGATATTTCATAAAACGGACTCT
CTATAGACCCCCAATGACCAATCCTCGCATGAATAGCTAAGGATCCAATAAGTCCAACAT
TGATTCCTTCGGACGTGTCAATTGGGCAAATACGTCCATAGTGACTAGGATGGATATCTC
GTATCCGAAAACTAGCAGTTCGCCCTGTTAATCCTCCAGGACCCAAATAACTCAATTTTC
GCCCATGAACTATTTGTGTCAATGGATTAGTTCGATCCAAAACTTGAGATAAAGGGTGTA
GGCCGAAAAACGATTCATAAGTGGTTGTTAATGGAGTTGGAGTTACCAAATTATGAGGAG
TCGGTATCAATTTATGCCTGATTGCTCCACATATAGTTCCTCGAACCGCATTTTCTAAAC
GAACCAGAGCCAATCCGAATTGATCCTGTAACAGATCTGCTACAGAACGAATACGTTTAT
TTTTCAAGTGATTCATGTCGTCAAGTGTGCCCATTCCAAATTTCATTCCGATCAAATGAT
CCACAGCAGCCAATACGTCTTGTGGTAACAAGAATGTATTGTTCGGAGGTATATCAAGAT
TCAGTCTCCGGTTCATATTTCGTCGACCAATCCTTCCTAATTCACATCTTTGTTGAAAAA
ATTTCTTTTGTAATTCCTTACATAAGGACTCAGAAAATACCGGATCCCCGCCTACACAAG
CAAATTGTTGATAAAACTCCAAAATGGCATTTTCCCTTGACCCAATCTTTTTTTTCTCCT
TATGATTCGGGAAAGACAAGAAAATTTCAGGGTAGCAAACATTATCTAGAATTTCTCTTA
GATTCGAACCCATAGCCGATGATGGAACTAGGATAGATATTTTTTGTTTCCTACTCACAC
GGGCCCATATCCTTGCTTTTCTATCAATCTCTAATTCTGATCTTCCCCCCCAATCTGATA
TTATGGTGCCGGTATAGACAGAAATTCCGCTATGGTCCAATTCTGAACGGTAATAAATAC
CGGGGCTTTGCAATATTTGATTGATTACAATTCTGTATATTCCACTTACTAGAGAGGTTC

CCAGGGAATTCATTAGAGGAATATTTCCAATAAATACGGTTTGTTCTTGCATATCTCTAC
CGGTTTTCCAAATTAATCCCGCGGATACATATAATTCAGAAGAGTATGTGAGTGATTCAT
ACACAGCATCTCTTTCTTTTATCAAGGGCTCTGCCAATTGATATGTTGCCACAAATAATT
GAAATTCAATTTCTTGGTCTGTATCTTCAATTTTTGGAAACTTATGAAGTTCTTCCATCA
AGCCTTGATCAATGAACCTACAAAATCCGTCAAATTGTATCTGACTAAACCCTGGTATTG
TATACATTCCCTCATTTCCATCCCGGAACATCTTAAGTTTTCCGTTTATCGAAAAAATCC
AACTATTGGCTCACTCTTCGTTGAACCATATAGATTGATCTAGCAACGATGGAATGTATA
TTTTGCTCATTTGAACAACATGAAATTTTATCCAACCCCATATACATATATACATGTACT
AAATACGTATGAACGGAGGAATAAAAAAAAATGTGACTCAAATTCGAATTTGCGACAGAT
ACAAATGGAAATGAATTGATAAAACATTCCTGGAAACAAAATTCTGCCACTTAGACTTAT
GGAGTCTTGTATAGAATATCAAAATAGATCCAATTTCTACCTTATGATATTACGATCAGA
TTGGGTACCATAAATGGATTCGGAATTGAATCTGTTCTCTATGAGTGAGATAAAGACAGA
ATAATCAGGAACCGTCTAGAGTTGACTTTGATTTTAGCACTTAATTTATTTCATCGATTC
CGTTGTTCAAAAAATGATTCGCAGAGAGAAAAGATATTTCTACCCATTTGTTAATTAGTA
GAATACGATTGAAGTGCGTAAGAGAAGTCATATTTATTAAGTACATGCAGATATAACTAT
CTAGCTATCCGTATATCCCATCTTTTATCGAAGTTCCCTTGAGGCAACATAGGTCGTGCT
ATATCCAAATTTCTATTTTATATTCAATATATTCAATGAAAAATGCAAGCACGACGATTT
CCTAATAGGAATATGTAGATAAGATACCTGACTAGGTATCCGTGTAAGAATTTCTGTTCT
GGGGTTTACATATACACATAATTGTTGTTATAATTGAAATTGAAAAGGATTAATTATGGA
AAAGAATTGAGACTGATTAGTCGTATATCAATTTGATCTCCTTATGTCATTAAGGAAACC
AAATTGGAGATCAAAACCCAAGAACCATTCATGAATTCACAGTCATTAATGCTTCCAATT
TGCTCTGAATTTTGGATTCTGTGACTGGAAATCCATTTTTCTCTTTCAATAGAAAAAGGG
GGGAAAGCTTTTATTTAGGTGTTGTGTGTTTTGAAATACAATCAATCTAAGAGAACAACA
GGATCCAATCAAAAAAAAGAAATGGTTCAGCAATTCCCCAGAATATTTCCATCTATATCT
ATTTTGTATCGTTTTGGCGGCATGGCCGAGTGGTAAGGCGGGGGACTGCAAATCCTTTCT
CCCCAGTTCAAATCCGGGTGTCGCCTGATTAACAAAAGACTCGGAATTTCTTACCCTACT
AAAAGAACTCACAAAATTCTTGCCTGGCAGAAGCAGAGGTAAGGGGCGGGGACTG
TCGATACCCAATTTTAAGAATCGGGGGGTTGACTTTCAATTATTTCTTCAAAAATCGGGG
TGTGACCCAAACCTGTACCATACCAATATGAATTACCAATATAAATAAAGAAATACTCAC
TTAATTACGGATTGCTGATGCGTTCAGGCCATTGATTTGATTTATCAATCGAATCAAATC
CATAGTAAGATTCAATTGTGAGATTCAGAACTACGAAAGTCAGGGACCGTAAATCCGTGT
ATCCAAAGGAAGGTTCCTAAGAGACTGTAAATCCTTGCTCCTAGGATCCAAGAAGGGGTT
CTAGGAAGGACTATAAATACTTCCTAATTACCTTACCCTACTAGTGTTTACTGACTGAGT
CTTGAAGTAGATTGGGTAGGCTGGTGGGGAATCCAATTAGGAGTTGTGGAAAGAACTGAA
GATACTTTGTATCCATACAAACTCATGAGAGTCTCTGAGTGCTCAGGTTTTCAATTAATA
TTGTATGGGTGATTGGCTTTTATAGAATAAAAGTGGAAAAAAGTGCTTTCGTTGGGGTAA
CCCCGCCAAGAATGTAATAGGGTGTCTTCCAATTGTTTCACATTTCACAGAAGTAGAGAC
AGTAAGGCTTAGATGGGAAATTAGGAGTATGTGATAGATAGTTATATATCTTGATGGTAT
ATTTTTTTTTTCTGCTTTTTGTTTATGAAAGGCAACAATAGGTCTTACTATTCCTACATA
TTCCATTAGTCACATTCCCTTGAGACTTCCAAGGGGCAACTGTGTATCTTGCTTGTACTT
AGTGCTTTCCGATTCCACCAGAAATCATATAGGGACTTGTTACGGGTGTATTCATTGGAT
TGGTTCATCAAAAACGTTAGGTCAAAATCCCATTTTGACTCTGCACCATTGATTCCACTA
TTATTAGTGATCAAGAATGGAATAATTCCTTCATATTCATAGAGATAGGGGACATGATTC
ACATGGATATAGTCAGTCTCGCTTGGGCTGCTTTAATGGTAGTCTTTACATTTTCCCTTT
CACTCGTAGTATGGGGAAGAAGTGGACTCTAGGGGTACTACTAATTGAGTTGAGTCATCG
AATTGTATCAATTGTTTATTAGATCGTTCTGCGAATCGCTTTTAAAAAAAAATCTCTCCA
ATTTAGAAATTCCACAGGAATCCAATATGAATAAGAACCTTTGGATCAAACAAATATTTC
AACGACTTGATTCCCGTGTTCGTATTTCGAAACTCAAAGGGATACACATGATGGGAAATT
TTTTCCAACCGAATTCTTTCTAAATATTCTATTTCGACAAATCGGCCCTTACTAGAATTA
TGCATATTACAATGAGGAGCAACCAACCCCTATTTTTTTTTTGATTTGTTTCCCTTTTCT
CTTTGCTGTTCAAAGAGGGAACCGTTCTTCTATTACGTACGTGGATATGTACTTTCTACT
GAGGCGACATAGACATAGTCGTTGTCCAAAGAGGTACTACGCCTAATAAGATCTTACTTT
CGTTGGGTATGCGTACTTACCTTTTTATACTCCTAGGAATCTTATTTATGCTTTATCGAC
TCGTCTCATGTCATGGTTCAAGCATGAAAAATCGGTGGGGTCTACTACATCCTTTTCAAA
ATCCGAAGAAGTTACTATAGAACTTTTTGGATCATCCGTTAACGGATCAATCAATTACTT
CTTCGTAATGCTAAAAAAAGGCTTGGTTTTCTTTTATATAATATATGCCCATACTAGTCT
TTCTCCATTGATTCTTTCAATGGATCCCCGGATCCATATTGAAAATAATCAGAAACCCAG
GAATTAGAAAAGTTGACGTTCGATTATTTCAGATTGATCGGGATCAATACAAATTGACGT
AACAAAGAAATAGAATTGGAGTGCTATTTACATGTACATATATAAATGTGGGTACATATT
GTGGATTGATCTATATCAAGCTCATACCTTTCTACAATAATAGATAGTGTGGTAGAAAGA
ACTATAATGAACCTTTCTACCATACTATCTATTATACTGCTGACTCCAACCCGACCATTT
AAGACTTGGAATTTGAATCCCTTTCTTTCATTTCTTCAATCGTTGATAAGAACTAATAAG
TCAAGTTTCAGTCAAATTAATCACTTTGACTGACTGTTTTTACGTAGATTATAAGTAAAA
AAGCAGTAGGAACTAGAATGAACAACGCAATAGCAATAAATGCTAGAATATTGACTTCCA
TAATCTCATCGTTTTTTTTTGCTTCGCAATAACTCGGGATCTAATCCCATAGAGATGATA
AGTCTTTCTCCTGTAAATTCAATAGGATAGATTGTATCCTGATGATACTTAATCGTATCA

ATATCATGAATAACAATATCTGATCTATCAAATCGATTCATCGTCGAGAATTGAATAGTA
TAACATAGGAAGATCTTTTATCCATACCGAATCCAAAATTGGATTCCTGGTCCAATCAAG
AATCCCATTGAATTTCTCATTTCCACTCTTTCTTTTTTATAACCTGCCGTCTTCCTTATA
CAATCATCTGGCCGGCGTTCCATTGGTCACAAACCCAAACGGTAGGGATGAAATGGAAAA
AGGAATGAGTTAAGTTCTAAACGAAGTTTTTGTGAAGATCTACTCTTTTTGGAAGACAGA
GAAGTGTGATAAAGATTGGTCCGGTAGAAAAGATCTAACAGAATATTCTATTCTGACAAA
TTCATTTATTTATTGATTTTGTTTTTTCTTCGATGGGGCCATTAAAATAGGAAGAAAAAA
AAAAGGGGGGGGGGTAGGTTTCATCTGAAAAGTACTCTGTCGCTGTCGGGGTAACTAGTA
ACTATACTATATTACTATATTAATTAAATTAATTGCGTATCGTACAATAAACG
AATACAATTTGTGTATGTGCTCCCGGGAAACGTATGAGTACTCTATTACATGGACCAGGA
GCAATCGAAAAAGACAGGCCCGTAGGGTCTCTCTTGAAATCTGAATAGGGCGATACCGCC
GATCAATCTACATATGTCTCTCCCCATCAATCGGTACTAGTTGAAGTAATTGAAAGTCCC
ATATTTGTACGATGAGAAATGCGAAACGAAAAACACGAAAGAAATAAGGATCCCCCGGGG
ATTAAATCCTGCTCCTTGTCCCCCCTCTTCGCAGAAAATGGGGAGATGAGTTGATGGATT
CATCGGATTCTAGGTCGGGACTGACGGGGCTCGAACCCGCAGCTTCCGCCTTGACAGGGC
GGTGCTCTGACCGATTGAACTACAATCCCGGGAAATGGGGTGTACAGCATACATACATAT
TCTTATAATTTCATTCGAACCCTTTCTTTCTATATTAGATTGAAAATCGACATCT
TTCTGTTACAAGAAAGACGAGTGATATACTGATATACACATGGATATGGACTATAGTGGG
AGTGACACGGATTACTAGTAATCCTGTGGTTATTTTATTACCCAATCAATTGATAATCCA
TTTTTCAATGAAAAAAAAAAAAAAGGACTCTTTATTTTTATCTATCAGGCATTTCATTTAT
AGAGGACAAACTGGTTATATCATCCTCATGGATCGGCGAATTGTTGGGCCGAGCTGGATT
TGAACCAGCGTAGACATATCGCCAACGAATTTACAGTCCGTCCCCATTAACCGCTCGGGC
ATCAACCCAGGAAGAATCAATTCTAGGCTTATTGATAATCCATGATCAACCCCCTTTCGT
CTTACCCCCAGGGGAAGTCGAATCCCCGCTGCCTCCTTGAAAGAGAGATGTCCTGAACCA
CTAGACGATAGGGGCATACCCGCCCGATCGCCATCATACTATCTATGCTCATAGTATGAG
CAGTTTTTTGAAATTGTCAATATACAATATATATGACTAGATCCGAAGAATCTTTCTTGC
TTACAAGATTCCATAGAATGGAATTTTGGGATTGTTGATTCATGAACCATCCTATATATA
AGAGAGGATAGGATCCTTCAGGGAGTGATTTGTCCGACAGAAAAAGGGCAAACCCCATTC
CATTTCTTTCATTTTCACTCGTTGATTCGTTCGTCGTTAAGGTGAGATATGCCTATCTCA
CACTAACACTAAACTAAGCCAGGAAATTCAGAAACGATAGAATTTCTTTTTTTGAGGATC
GACGAATAATCGAAAAGATTCTTTTTTTTTTTTCTAATAATTTAATTTAGGGTACGAGTC
GAATCCCTTCATCACATGATTCGATGAAATATCTTGGATCTATATCGAATTGGTACATGT
ATCAATCAACCAAGTGAATCTCGTCCGGATGAGTCAATAAAAGCAAAGCAATTAGGAGCG
GCCCTGAAACAATTCATTGCATTGATATTTCTCAAATATAAATAACTAAAACTTCCTAGG
TAAATAAAATTTCTTGTTCCTGAATGAGCCCCTATGTATACATGTACATTATATACATAT
ACATTGTAGTACATACATAGATACATGTAGTAGACTCTATAGTAGCTAGTGGTTAATTCA
TTTTTTGAAGAAAATGGGCCCTTTTAACTCAGTGGTAGAGTAACGCCATGGTAAGGCGTA
AGTCATCGGTTCAAATCCGATAAAGGGCTTTTTCTACGAAGCTCCAGTCTTAGTCTTCAT
TTTTCATCGGAGAATAGAGATATTGTTGATATTTGTAATAAAAGTCACCCATAATGAGTT
ATCATTCTAATGAGTTATAGGTATAAAGTGAAACAGTTGTTTATTATGATTATGATAAGT
AATCGCACTTAGTAGGAGGACTACTATGTAATTCACTACAGGCTATACCCCTCCTCATAT
TATTCCTATTTTTGGTCCTGGGACATAGATATTCTAGATACCCAATCCAAATTGTGAATC
GCCAAACCAAAGTATTCCCATTTCTCTATTGTTCCAATCAAATCCCTCGGAAAAATTAGA
AATCAAGAAAATCAAAAAGTAAGTGGACCTGAGCCATTGAATCATGACTATATCAGCTAT
TCTGATATTCAAATTCGATAGAGATGAAATTGTAGAAGCGAACTTTTTCTTTCCTTGGAC
CACGCAATAATTTGTCGATATTTCAGATTGAATCTTCTTGTTCCTGGATGCTCCATAGGA
ATAAATTGCTCTTCCTTTCCTCCACAGAGATACGTTTATTCCAAGTCACAAGAGCAATCT
CTTTTTCAATACCTTTCTTTGATTCCAGAAAAAAAGAAGTTTCTATCTATATAGGATTTA
GATATAGATATCAAATCATGGCTTCAGGTACAAAATATTTCCATATTGATGCATCAGATA
TTTTTGTTCCTCCAATGCAACGGAAAACGAGTGCGATAAAGGAGGGATTTTGATTTCCAG
TCTCCCTATTTAATTTAGGGGGCAGGGACAAAAAATAGGGTCCTTTTTTTTCTGCCGGAT
ATAGGGTAATAAAAAAAAGTAAAGAGGGAAATATTCGAAGTTTATTTTTTTGGTTCGAC
CCGCGAAAAGATATACTCTGGAATTTTAGATTCATTCGAAGGAAATATAACAAAGAAGAC
AATAACAAACAAAAAGCAATCAAAAAAGGAAGGAGTAAGAAATTATATATATATATAGGA
TACTGTAGTAGTTTAGTATACACATAAATTACGAGAATCCATAAAGATATTTATTGATCT
TTTCTCAATAAGATCCAAGAACACGAATACGATTAGCTTATGGAATGGCGAGCTAGATCT
GGGGAGCAACTGATAACGAGAGAAAGGATCGCTTGTTTCCTCACAGTTATTTCAAAAAAT
GGATCTGATTGATGGGTCATAAGACAATTCAGGGTTCGGATGGTTATTAAGAATAAGAAG
GAATAAGGAATAGGAAGGAATAATTGAATCGAACTCATGGATTTACCTAGGTTGGTTTCT
GGCCCAATAGAAAGGAAGGATTTGTATCTTCGAAACCCATTGGATGGAAGGCGCAGTGGA
CGAGGAATCGTTCATAGATGACCGAACCATCGTATGCCCTGAGAATGATATGAGGTGTTC
GGAAATGGTTGAAGTAGTTGAATAGGAGGATCGATATGACTATAGCCCTTGGCAGATTTA
CCAAAGAAGAAAATGATTTATTTGATATTATGGATGACTGGTTAAGGAGGGACCGTTTCG
TTTTTGTAGGTTGGTCTGGTCTATTACTCTTTCCTTGTGCTTATTTCGCTTTAGGCGGTT
GGTTCACAGGTACAACCTTTGTAACTTCATGGTATACCCATGGATTGGCCAGTTCCTATT
TGGAAGGCTGCAATTTCTTAACCGCTGCAGTTTCTACTCCTGCTAATAGTTTAGCACATT

CTTTGTTGTTACTATGGGGACCTGAAGCACAAGGAGATTTTACTCGTTGGTGTCAATTAG
GCGGTCTGTGGACTTTTGTTGCTCTCCATGGAGCTTTCGGGCTAATAGGTTTCATGTTAC
GTCAATTTGAACTTGCTCGATCTGTTCAATTGCGACCTTATAATGCAATCGCATTTTCTG
CTCCAATTGCTGTTTTTGTTTCTGTATTCTTGATTTATCCACTAGGTCAGTCTGGTTGGT
TCTTCGCGCCTAGTTTTGGTGTAGCAGCTATATTTCGATTCATCCTCTTCTTCCAAGGGT
TTCATAATTGGACGTTGAACCCATTTCATATGATGGGGGTTGCTGGAGTATTGGGCGCTG
CCCTGCTATGCGCTATTCATGGTGCTACTGTAGAAAACACTTTATTCGAGGATGGTGACG
GTGCAAATACATTCCGTGCTTTTAACCCAACTCAAGCTGAAGAGACTTATTCGATGGTCA
CTGCTAACCGCTTTTGGTCTCAAATCTTTGGGGTTGCTTTTTCCAATAAACGTTGGTTAC
ATTTCTTTATGTTATTTGTACCAGTAACCGGTTTATGGATGAGTGCTCTTGGAGTAGTCG
GTCTAGCTCTGAACCTACGTGCCTATGGCTTCGTTTCCCAGGAAATCCGTGCAGCGGAAG
ATCCTGAATTTCAGACTTTCTACACCAAAAATATTCTCTTAAACGAAGGTATTCGTGCTT
GGATGGCGGCTCAGGATCAGCCTCATGAAAATCTTATATTCCCTGAGGAGGTTCTACCCC
GTGGAAACGCTCTTTAATGGAACTTTAGCTTTAGCCGGTCGTGACCAAGAAACCACTGGG
TTCGCTTGGTGGGCCGGGAATGCACGACTTATCAATTTGTCCGGTAAACTACTCGGGGCT
CACGTAGCCCATGCCGGATTAATTGTATTCTGGGCCGGAGCAATGAACCTATTCGAAGTG
GCTCATTTCGTACCAGAGAAACCTATGTATGAACAAGGATTGATTTTACTTCCCCATCTA
GCTACTCTAGGTTGGGGAGTAGGTCCGGGTGGGGAAGTTATAGACACCTTTCCATACTTT
GTATCTGGAGTACTTCACTTAATTTCCTCTGCAGTCTTAGGCTTTGGCGGCATTTATCAT
GCACTTCTAGGACCTGAGACTCTCGAAGAATCCTTTCCATTCTTCGGTTATGTATGGAAA
GATAGAAAGAAAATGACCACAATTTTGGGTATTCACTTAATCTTGTTAGGTATAGGTGCT
TTTCTCCTAGTGCTCAAGGCTCTTTATTTTGGAGGCGTATATGATACCTGGGCTCCCGTG
GGGGGAGATGTCAGAAAAATGACCAACTTGACCCTTAGCCCAAGTGTTATATTTGGTTAT
TCACTAAAATCCCCCTTTCCCTTGGGGGAGAAGGATGGATTGTTAGTGTGGACGATTTAT
AAGATATAATTGGAGGACATGTATGGTTAGGTTCCATTTGTATACTTGGTGGAACCTGGC
ATATCTTAACCAAACCCTTTGTTTGCATGGGCTCCCCGCGCATTTGTATGGTCTGGAGAA
GCTTACTTGTCTTATAGTTCAAAGGCTTTATCTGTCTTTGGTTTCATCGCTTGTTGTTTC
GTCTGGTTCAATAACGCTTATCCTAGTGAGTTTGACGGGCCCACTGGCCCAGAAGCT
TCTCAAGCTCAAGCATTGACTTTTCTAGTGAGAGACCAACGTCTTGGGGCTAACGTGGGA
TCCGCTCAAGGACCCACTGGTTTAGGTAAATATCTAATGCGTTCCCCGACCGGAGAGGTC
ATTTTTGGAGGAGAAACTATGCGTTTTTGGGATCTCCGTGCTCCCTGGTTGGAACCTCTA
AGAGGTCCCAATGGTTTGGACTTGAGTAGGCTGAAAAAAGACATACAACCTTGGCAAGAA
CGACGTTCGGCAGAATATATGACTCATGCCCCTTTAGGTTCTTTAAATTCCGTGGGTGGC
GTAGCTACCGAGATCAATGCAGTTAATTATGTCTCTCCTAGAAGTTGGTTAGCTACCTCT
CATTTTGTTCTAGGATTCTTCCTATTCGTGGGTCATTTGTGGCATGCGGGAAGGGCCCGT
GCAGCTGCAGCAGGATTTGAAAAAGGAATCGATCGTGATTTTGAACCTGTTCTTTCCATG
ACCCCTCTTAATTGAGACAGGAGATCAAATGCATGAAGTAGGAATCCATTTGATTCCATT
ATACATATTAGGTTAAGATCAGGTCATATTTAAAAAGTATTCCTTGTTTTTTTCTTTTCA
TTCTATCTATTTTTTTCTGGCTCGGCTATCCCACCTAGCCGAGCCATTTCCCTTTATGAC
ACCGGGCCAGGCCATACCAATAAAGAAACAAATCGATTCAACGAGCAAAAGGAGAGAGAG
GGATTCGAACCCTCGATAGTTCTTTGTTCGGAACTATACCGGTTTTCAAGACCAGAGCTA
TCAACCACTCAGCCATCTCTCCAAGAGACAATCTCCATTTTATTCCTCCGAATAGAACAT
GGCCATATGGGTTGATACTCTAACTATCTGTAGAAACATCCCAAGTGCGAATCTATATTT
CGACATATCTATCTGTCTATAGATGCATGATCCAACATGCCCATTTGGGAAGTCAAAAAA
AAATTCCCTGATTCCATGTCCGAATAAAATAAAGTGGGACTAAGTTCGAAAGGATCAATA
AATTCATGGTCAAATCCCGTCATGATGCATTATTTCAATTTTGACTCTGAGAGAGGGATC
AAATGGTATAGTTCATTTGTTGGTAGCTTGGAGGATTACAAGCATGACTATTGCTTTCCA
ATTGGCTGTTTTTGCATTAATTGCTACTTCATCAATCTTACTGATTAGTGTACCCGTTGT
ATTTGCTTCTTCTGATGGTTGGTCAAGTAACAAAAATGTTGTATTTTCTGGTACATCGTT
ATGGATTGGATTAGTCTTTCTGGTAGCTATTCTTAATTCTCTCATCTCTTGAACCTATTC
GGTCTTTCCCGGATCAAAAAACTGACCCCTCCCCAAATTCTTTCGGATTGTAAGACACAT
TAAAATGAAATATGAGTCCAAAAATAAAAATAAAAAAATTGGAGGGAGGGGTCAAAAATC
ACTTCTTGAATAAAAAAAACGAAGAATCTAATAATAATTGGAATCTTCCTAAGTATCTGA
CCCTGTCTGTACAAATGGGATCCAGACACATATATGATATATCATATATGTGTGGACATA
TACGTGTGTATCAGGAACGAAGAAAGTGCGGATATGGTCGAATGGTAAAATTTCTCTTTG
CCAAGGAGAAGATGCGGGTTCGATCCCCGCTATCCGCCCATGGTAAAGTAAGGTAATATG
ATAAATGATTTAGGTATAGTTGACCACGATAGGGGAGTGGTTCTATTCTTCCCATCCCAA
AACCAAAATAGCCATTGGTTACTAGGTAACGGAATCGCACCTAAAAATGTTTTTTTGAAA
AAAAAAAAAAAAAGAGATGTTGCGGAGACAGGATTTGAACCCGTGACCTCAAGGTTATGA
GCCTTGCGAGCTACCAAACTGCTCTACCCCGCGCTGAAAACTAATGGACGAACAAGAATT
GGATGTGCCCCCATACCATATTCTATATAAATAGAATAGCCCATTTATACAGAATGGTAA
AGGGGGCCCCTCTATGATCATAGCTCATAGAGATAAATAGAAATAGGAAGAAGGGGTATT
TTTATCCTTACCAACTTGATCTTGTGGCCCCCGGCAACAAACATGCCTGAACCTTTTCGT
GAAGTATGTGTCCGGATAGCCGAAAGTCTCGATAGCTAGCTCTAGGTCTTCCGGTCAAAA
AACAACGTCGATGAAGACGTGTAGGTGTACTATTACGTGGTGGGGATTGCAATTTTCCAT
GAATTTCCCATTTGTCACTCAACGATGAAACTTTGCCTATTTCTTTTTTTGAGGATCGAC

GAATCAAATGATATTTCTGTTCCAATTTCTGCTTCTTTCTCTCCCTCTGAATCAAACTTT
TCCTTGCCATAAAGGTTCAGTTCCTATTATTATCAATGATACGGGTCGGATCCTAGATGT
AGAAATAGAAGAAGGTGGATTCTCCCTTCTCCATCGAATCAAATGAAATTGTCGATGATA
CAGCACATTAAAAAAAAAAAATTAACCAAATTTGCCTGATGTAGAGGCAATCAAGAAAGCT
GCATAAGTGAATATATAACCTACAGAAAAGTGGGCTAATCCAACCAATCTTGCTTGCACA
ATGGAAAGAGCCACTGGTTTATCCCTCCATCGAATCAAATTAGCCAAAGGCGTGCGTTCA
TGAGCCCATGCTAAGGTTTCAATCAATTCCTGCCAATATCCACGCCAGGAAATTAAGAAC
ATAAATCCAATAGCCCAAACAAGATGCCCAAATAGGAACATCCATGCCCAGACCGATAAA
CTATTCATACCAAAGGGGTTATAGCCATTGATAAGTTGTGAAGAGTTTAACCATAGATAA
TCTCTTAACCATCCCATCAAATAAGTGGAGGATTCGTTAAATTGTGAAACGTTACCCTGC
CATAATGTGATGTGCTTCCAATGCCAATAAAAAGTAACCCATCCGATAGTATTTAACATC
CAGAAAACCGCCAAATAAAATGCGTCCCAGGCCGAAATATCACAAGTACCGCCTCGTCCC
GGACCGTCGCAAGGAAAACTATAACCGAAATCTTTTTTATCTGGCATTAACTTGGAACCA
CGTGCATCTAAAGCACCTTTTACTAAGATCAATGTAGTTGTATGCAAACCTAGAGCAATA
GCATGATGAACCAAGAAGTCTCCAGGACCTATTGTTAAGAATAGTGAATTACTATTCTCA
TTAACGGCATTCAACCAGCCCGGTAACCATATACTTCGACCAGCATTGAATGCCGGGCCA
TTCGTTGAAGATAAAAGTACATCGAACCCATATGAAGTCTTACCGTGAGCGGATTGTATC
CATTGGGCAAATATGGGTTCGATCAAGATTTGTTTCTCCGGAGTACCAAAAGCGAGCATG
ACGTCGTTATGAACATAGAGTCCTAAGGTATGGAACCCTAGAAAGAGACTGGCCCAACTT
AAATGAGATTTGATAGCTTCTTTATGGTCTAACATTCTTGCCAATACATTATCCTCATTC
TGTTCCGGATTGTAATCTCGAATGAAGAATATAGCTCCATGAGCAAAGGCCCCTGTCATG
ATGAATCCTGCGATGTATTGGTGATGAGTATATAACGCAGCTTGAGTAGTAAAGTCTTGT
GCTATGAATGCATAAGCAGGTAAAGAGTACATGTGTTGAGCTACCAAGGAAGTAATAACC
CCTAAAGAGGCTAGAGCAAGGCCTAATTGAAAATGAATCGAATTATTGATTGTGTCATAA
AGACCCTTATGCCCACGTCCCAATCGACCCCCCGGAGGAATATGTGCTTCTAAAAGATCT
TTCATACTGTGCCCAATCCCGAAGTTAGTTCTATACATATGACCAGCAACGAGAAAAATA
AATGCAATAGCTAAATGATGATGAGCAATATCGGTCAGCCATAAACTTTGCGTTTGTGGA
TGGAATCCCCCGAGAAGGGTTAGAATGGCAGTTCCCGCCCCTTGGGAGGTACCAAATAAA
TGACGACTGGAATCGGGGTTTTGGGCATAAAGATTCCACTGACCTGTAAAAAGTGGGCCC
AACCCTTGAGGATGGGGTAATACATCTAAGAAATTATTCCATCTGACGTACTGCCCCCTT
GATCCTGGAATAGCGACATGAACTAAATGCCCTGTCCAAGCCAAGGAACTGACTCCGAAG
AGTCCTGACAAATGATGATTGAGACGAGATTCGGCATTTTTGAACCACGAAACGCTTGGT
TTCCATTTGGGTTGTAGATGTAACCAACCCGCAATTAAAGATATGGCAGAAAGAAATAAT
AGAAAAAGAGCTCCAGTATAAAGATCTTCATTGGTGCGTAAGCCAATTGTGTACCACCAC
TGATAAACACCGGAGTAAGCGATATTCACTGGGCCGAGAGCCCCTCCTCGAGTAAAGGCT
TCTACAGCCGGTTGACCAAAATGGGGATCCCAAATTGCATGAGCAATAGGTCTTACATGT
AAAGGGTCCTGTACCCATGACTCAAAATTTCCTTGCCAAGCTACATGAAACAGATTTCCG
GAAGTCCACAGAAAGATTATTGCTAACTGCCCGAAGTGAGAAGCAAAAATGTTCTGATAA
AGACGTTCCTCAGTAATATCATCATGACTCTCAAAATCATGTGCGGTAGCAATACCAAAC
CAAATACGACGAGTAGTGGGGTCCTGAGCTAAGCCTTGGCTAAACTTTGGAAATCTTAAT
GCCATAATGCCTTTCAAATCCTCCTAGCCATTATCCTACTGCAATAATTCTTGCTAAGAA
GAATGCCCATGTTGTGGCAATTCCACCCAGAAGGTAATGGGTTACTCCTACAGCGCGTCC
TTGTACAATGCTCAAGGCTCTAGGCTGAGTAGCAGGAGCAACTTTTAATTTGTTATGAGC
CCAAACGATGGATTCAATGAGTTCTTGCCAATAACCACGGCCGCTGAATAGAAACATTAA
ACTGAAAGCCCAGACAAAATGAGCACCTAGGAAAAAAAGGCCATATGCCGATAATGAAGA
ACCATAAGACTGAATTACCTGAGATGCCTGTGCCCATAAAAAATCCCGGAGCCACCCATT
AATAGTAATGGAACTCTGTGCAAAGTTTCCTCCCGTAATATGAGTTACCACCCCTTGATC
ACTTATACTACCCCAAACATCTGACTGCATTTTCCAACTGAAATGGAATATTACTACCGA
AATAGCATTGTACATCCAGAATAGACCTAAGAAGACATGATCCCAGGCAGATACTTGACA
TGTCCCCCCTCTTCCAGGTCCATCACAAGGGAAACGAAAACCAAGATTTGCTTTATCAGG
TATCAAACGTGAGCTACGAGCAAATAGAACACCTTTCAGTAGTATCAATACGGTCACATG
GATCGTAAATGCATGAATGTGATGTACCAAAAAATCTGCGGTTCCTAATGGAATAGGTAA
CAAAGCGACTTTACCGCCTACTGCTACTAAATCACCACCCCCCCAAGTCAAGCTGGTGCT
TGTTGTTGCACCAGGAGCTGTTGCACCAGGTGCTAAAGCGTGGGTGTTTTGTACCCATTG
AGCAAAGATGGGTTGTAATTGTATAGCGGTATCTGAAAACATATCTCGGGGACGCCCTAA
AGCGCTCATGGTATCATTATGAATATACAAGCCAAAACTGTGAAAGCCTAGAAATATACA
TACCCAGTTGAGATGTGATATGATTGCATCTCGGTGTCTAAGGACACGATCTAATAGATC
GTTGTATCGAGTAGTTGGATCATAGTCTCTTACCATAAAAATGGCTGCATGTGCAGCAGC
ACCAACTATGAGAAATCCACCGATCCACATGTGATGTGTGAACAACGAAAGTTGTGTACC
ATAGTCAATAGCTAGGTATGGATAGGGGGGCATGGAATACATATGGTGAGCTACAACAAT
GGTTGAAGAGCCTAACATAGCTAGATTAAGAGATAATTGAGCATGCCATGACGTTGTTAG
GATCTCATAGAGGCCTTTATGACCCTGGCCCGTAAATGGGCCTTTATGCGCCTCTAAAAT
ATCTTTCAGGCCATGACCAATGCCCCAGTTGGTCCTATACATGTGACCAGCTACCAGGAA
AAGAATTGCAATAGCTAAATGATGGTGTGCAATATCTGTCAGCCATAGACCCCCTGTTAC
TGGATCTAATCCTCCACGAAAACTAAGAAATTCCGCGTATTTTGACCAATTCAAGGTGAA
AAATGGGGTTGATCCCTCGGCAAAACTGGGATAAAGTTGAGCCAAAAGATCCCGATTCAA

GATAAATTCATGAGGAAGTGGTATCTCTTTAGGATCCACTCCAGCGTCTAGAAATTGGTT
AATCGGTAAAGATACATGTACTTGGTGTCCCGCCCAAGAAAGAGACCCAAGCCCTAGTAA
CCCCGCTAAGTGGTGATTCAACATGGATTCCACATCTTGGAACCAAGCCAATTTTGGGGC
AGCTTTGTGATAATGGAACCAACCGGCAAAAAGCATTAAGGCTGCAAAGACCAATGCACC
GATTGCGGTACAATAGAGTTGTAATTCATTAGTTATTCCAGATGCTCGCCAAAGCTGAAA
AAAACCAGAGGTTATTTGTATTCCTCGGAAACCCCCGCCCACATCACCATTCAATATTTC
TTGACCTACTATTGGCCAAACAACCTGGGCGCTGGGTCCAATGTGAGTAGGATCACTTAG
CCATGCTTCATAATTAGAAAAACGGGCGCCATGGAAATACATACCACTCAGCCAAAGAAA
AATGATGGAGAGTTGACCGAAATGAGCACTAAATACTTTTCGAGAGATCTCCTCCAAATC
ACTGGTATGGCTATCGAAATCGTGAGCATCAGCATGAAGGTTCCAGATCCAAGTGGTAGT
ATCAGGGCCCTTAGCTATTGTTCTTGAGAAATGGCCGGGTCTGGCCCATTCCTCGAAAGA
AGTTTTTATGGGATCCCTATCCACCAAAATCTTCACTTCTGGTTCCGGCGAACGAATAAT
CATTGAGTCCTCCTCTTTCCGGACAACACATACAAAGAAACCCGCCAACAGTCAAGTAAT
TAGTGAACGATGGGTATTTATGATTAGTTCCTTATCTTTCCTATCCCCCATCTATCTTTT
TTTTTTTTAGTTATTTACTAGAGCAATTATGATATGGAAGTCGATCCGGGGCAAGTGTTC
GGATCTATTATGACATAACCATGGGGCGCTCAACGGACCTTTATAATATTTTATAACCCC
CTCCGGCGTGACACAAAAACGGATTTTTTGATACAAGCTAGTTAGTGTATTCATATCTCA
ATGTATAGTATCTAGATGGATCTACTTCATATCTTACACGGAACATATTACTTACAATAC
AAATCAAAGGATCATTCATTAGTCATTAATAAGAGACATCTTGATATCTATATTTAGTCA
TTCGAGGTCTGTCTTTTTTACTGGCTTAGCTTTATTAGCATAGCAGAAGGGAATATTTTC
TGTACTGTATCCGTATCGTTTATCCCTATGACAGACGAAATAGAACAATCTTAGACTTAG
AAGGGATATAATGAAATTCCCTGATTGGCTCTTCCTAGAGGAACGATCTATTTTATTTGA
TTGATGGATCCCATATTATAATGAATTCAAAAAGAGAATGTTCTTATTCAAACCTCCTTG
CGATCTTCAACCAATTATGTGCTTCAATATAATTCCCCGGAGTAAGCGCTATAGCTTGTT
TCCAATATTCAGCAGCTTGATCGGACCAAGCCTCTGCAATTTCAGAATCTCCTTGTCGAA
TGGCCTGTTCTCCCCGGTCGGAATAGGTGGGTCAATTCCTTCCCTTAGAACCGTACTTGA
GAGTTTCCTACCTCATACGGCTCGACATTCTTTTGGTGTTCCATCTTAATCTACCATATC
TAACTGAATGAGATTTCTCATAAATCTATCCCATTTTTTTTTTTCGGGTTAACCAGAAG
AGGTTAATTACACGAGTTTCAAACTCTAATTTTGATCAATAATCAGTTTTCTCTTTTCTC
CCACCTTCAGAAGAACCAAGCATAGGTATTTTCCTCTATCGTTCGAATTTTCTGAAAGGT
AACTATCTCGGTTTCATATAGAAATTCATATAGAATCTTTGAAAAAGACTTTCCTCCATA
AGAAAGAAAGGACTTACTATCTTTGGGATCTGATGCTACACCGCTGCTCAATACCTTAGT
AGATCGACTCTATTACATAAGTTGATTCCTAACTTTTATCTCATATCATGACATTAAGTA
AGCAGTCCTTATTGTATCGGTCCCCGAACCTCACTAATTGATCTTTACGGTGCTTCCTCT
ATCAATTAGATCCTTTATCCATAGAATAAAGTATATAGGCCATACCTATTTCTTCATATT
TCGGCTCTTATGAAGTCTCTTTCTTTGCTACAGCTGATAAAAATCGTTGCTTTGGACGAT
GCATATGTAGAAAGCCTATTTTGTTTCTAGTATTGACTAGCGGATTTGGTCTTTCCTTCC
TTCTTTCTATAGTGTAGATAGTCGCACGTAATGACAGATCACGGCCATATTATTAAAAGC
TTGTGGTAAGAATGGATTTCGTTCTATTGCCCGGAAATAATATTCCAAAGCCTTCGTGTG
TTCTCCGTTACTTGTGTGGATAAGGCCTATGTTATAGAGTATATAACTTCGATCATAGGG
ATCAATTTCTAGTCGCGTAGCTTCATAATAATTTTGTAAAGCTTCCGCATAATTTCCTTC
GGATTGAGCTGACATCCGTTACGGTCGTTCATTCTATTCAAAGAATCTCCGTTCCAGAAC
CGTACGTGAGATTTTCATCTCATACGGCTCCTCCCTTCTGTGCATAGTAATAAGGGGAAT
AATCCATGGAATCAAAAAAGATTGAAATATTCTCATTATGAACTGACAGGGGCTGGTGTT
TTTACAAGAAATCTCTAGCCAGCCTTCCTGCAAGAGGTCTGTCTTTTCTTAACACCAAGC
GCGTTTGTGCTAGATAGAAATGGTAACTCCAACAATTTCTTTGTCCTCAACGCCCCCTGT
TTCCAGGAATTAGTCACTTCAACGACCTTTGATGGTTATACGGGTATCCAAAGTACGAAC
GAGATGGATGTTTGTTGTCCCAACCATTCTTGTTAGTTCCGATCCCGATAAGGAAAAGGG
TTAATTTATAACAAAGTTTTCGTGTTGTTGATTTCTAGATGTAGTGCTTCTTCCCCTATG
CGGCCTATTGGTACTAGTGGAGTAGGATTGACCCGCAATACAGAACCTATAGGTGTAACC
TTTCGCTCAATACTAGAATCGACAGTTGAAGCATCTAAGGCTGCATCAATCGGGGATACA
CGACAGAAGGAATTGTTCTATCTCCAAACTTCACCTTCATCAAGCGTAGGTTTATTTCAA
GAATCTTTTTTCTTTGTATCCCGAATCATGTCTCTTTCTCGTAAGACTGAGGGCGGTAAA
TAAATAAATTCAAAAAAAAAAGCAAATCGCACCATCTCTGTAATAGGTAAATGCCTCTTT
TTCTCCTGAGGTTGTCGGAATTATTCGTAATAAGATATTGGCTACAATTGAAGAGGTCTT
ATCAATAAAATTTCCATTTATCCGAGATCTAGGCATAGTTAACAATCCATTCTAGAATTC
TTCTCATTACCCCTCAGGGGAAAATGATTCCACAAACAAAGGAATTGTACAGTACGAAAT
CACATAAAAACAGACTCATTCTAAAAAAAAAATGTGGACCTTCCACTCAAATTATCCCT
TTTTGAGAGGTATAGATAGGAAATATTTGAATCGGATTGGATTTCATTGAAATTGAGTAG
TATACCAATGAATGGAACTCTTTTATTTTATCGAAGTTAAGAAATCCAGGAATTTTTACT
ACCGATTCTTATAATTTAATTCGATAAATTTGGATTTGATTATGATCCAAAGAGGAAAAA
GAATCAAATAATCATTCCATGATGAAAATAGAATAACCATCCATTTTGTGTGCATAGTGT
GGATACACCATCCAATCGAAAGATAAAAATCTATAGAACGATTCATGAATTTGTAATAGA
TCTATGGAGTAGCTCATGAGAGGAGTTGTTGTTGAGAAATCTGAAACTGGAAGGGGGGAA
TTTTGTAATTCCTATGGAATCGTAGTTTAAATATAAACATAGTCTAAAATAGGGTCAGTT
GACTCGTTCCAATTCATTGGCTTAATCCGAAATATTAGAATAAGATAGGATCGTCGTATT

GACAAACAAGACATTTTTGTTTTTAACAAGAAAAAAGTGTGTTTTTTTTTATCCCTCGAGC
CTCGAAGGAAAATCGTTCTTTAGCGAAAAGTTTTCTATTTCTAATAGATTGGTCGTACCT
GTATTGCAATAATATGAATGACTCGCTATTTACTCGGTTTCTGGGGCATAATAATAAGAT
TATGTAGGAGAGATGGCCGAGTGGTTCAAGGCGTAGCATTGGAACTGCTATGTAGACTTT
TGTTTACCGAGGGTTCGAATCCCTCTCTTTCCGTACCTTCATCTAATTCACCAACCGACC
ACAATGTATCAAATCAAATAACAATTGATACCATTATTCCAACAGTAAGACCCTTATTTG
ATAGAGATTCTCTATTCCTAATTACTGCGGTACGGAAAATACCGGAAAGAGTGGAAAGG
AATGAAAATCTCACTGCTGATCCATTTGTGATACGTGAATGGGAGAAAAATCCGGATCAA
ACCCCTTCTTCGGTGAAAAAAAAAAAAAGAGGGGGGGGGCAAAATGGTCCGAAGCTTTGTT
ATTTTAGTTAGGTTCAAGTCTGACGGGAATAATATTCTACGACTAGAAACTCATTGATTT
TCAAACCGATCCATTTAATATCTATTATTTGATTTACTAATCCTTTATATTGGGATGAGT
CAAAAGTCAAATGTTTTGCCAAATCCTCGCGGGGCGATGAATCAAGATAATTTTGAATCA
GAGCTCTGGATCTTTGTTCATCCCTTGCAGTAATAATATCTCGGGGTTTGCAGCGATAAC
TTGGGATATCTACTACACGACCATTAACTAAAATATGTCTATGGTTAACTAATTGCCTGG
CTCCAGGAATGGTCGAAGCCATACCTAATCGAAAAAGGATGTTATCCAAACGCATCTCAA
GTAGTTGTAGTAAAACCTGACCTGTTGACCCTTTGGCTTTTCCGGCAATACGAACATATC
TAAGCAATTGTCGCTCTGTCAGACCATAATGAAAACGCAATTTTTGTTTTTCTTCTAGAC
GAATACGATATTGAGATCTTTTCCCGAAACGTGATTGGTTTCTAAGATCACTTCCGGATC
TAGGTCTTTTACTAGTTAGTCCCGGTAAAGCCCCCAGACAGCGTATTTTTTTGAAACGAG
GCCCTCGGTAACGAGACATAAAGACTCCTTGTTAAAATTGTATTTTACAGAATAAACTTA
AATTAAGACTGAACTAAACGATAAACGAAACTAAATCTATTGAAGTACTACAAAAGAAGA
CTACAAAAGAAGAATGAGATGAATTGTATCAATATCCGGATTATTTTGTATATATAGGAA
GTGAAGGACCCCTTTCTTGATTTGTTCTGTAGTGTAGAGATTTAACTGCTCCAATCAAAT
AAGTTTTTTATTCATAGTTGGAAGTTGCTACGACATAATAGATCGGTGACCCGACATTTT
TAAAAGAAAAAAAGAGAGGAGTCTTTTCAATATTCCTTGATCTCAAGGAATATTGAAAAG
CCGGCTATCGGAATCGAACCGATGACCATCGCATTACAAATGCGATGCTCTAACCTCTGA
GCTAAGCGGGCTCACATAACAGAAATAAGTGCAATAGAACTAACTAACTATATCTATATA
GAATGTTTTTTTTAATTCTTAATTTATATATTATACTTAATTTATAGCAGTTAGTTATAG
CAGATTAGATTAATCATATTAGAGCAGATCGGTACTAAGGAAAGGATAAGATAAGGATGC
AATCCAGATCATAATGAGACATTTCGCCGGTTTCATTCAGAAAGGGGGGAGGTAGAACGA
AAAAAAAAAATGAATATCGACCGTTCCAGTATTAAAAATCGCGCGGGAAAAATGAGAGGG
GGGGAGGGTATGTATATGTGGGATATCTCTATCCATATTGAATTGCAGATACATCAATGA
TAGAATCATTTCTGATGGGACCAAATACGGGTCTTCCGATAGAGAATATGGACAAGAAAT
CAAAATAAAATAAATAAAATAATAAAATAGGAGTAGACTTTTTTTCGATATTAGGAATCA
GTATCTAATGAATTCAACGGTTCCGACATAAATAAATGAAAGAGGGGGATGGGATCACAA
TGAGATCTCGGTCTCATAAGGGGATATGGCGAAATTGGTAGACGCTACGGACTTGGTTGG
ATTGAGCCTTGGTATGGAAACCTACTAAGTGATAACTTCCAAATTCAGAGAAACCCTGGA
ATTAAAAATGGGCAATCCTGAGCCAAATCCTGTTTTCAGAAAACAAGGGTTCAGAAAGCG
AGAACCAAAAAAAGGATAGGTGCAGAGACTCAAAGGAAGCTGTTCTAACGAATGGAGTTG
ATTAACATTGGTATAGGAATCCTTCTATCGAAATTCCAGAAAGGATGACCCTATCCTATA
TACGTACTGAAATATCAAACAATTAATCACGATCCGATTCCGTATTTTTTTTATATGAAA
AATGGAAGAATTCTTGTGAATCGATTCCAAATTGAAGGAAGAATCGAATATTCAGTGATC
AAATCATTCACTCCTCGGATAGATCTTTTGAAGAACTGATTAATCGGACGAGAATAAAGA
TAGAGTCCATTCTACATGTCAATACCGACAACAATGAAATTTATAGTAAGGGGAAAATCC
GTCGACTTTAGAAATCGTGAGGGTTCAAGTCCCTCTATCCCCAATAAAAAGAAAAGAGCC
CGTTTTACTACCTAACCTCTTTATTTCGTCATCGGTTCCAAATTAGTTATGTTTCTTATT
CACTCTACTCTTTCACAAACGGATCCGGACAGAAACCTTTCTCTCTTATCACAAGTCTAT
AGATACGATATACTTACAAATGAACATATATAGGCAAGGAATTTCCATTATTAAATAATT
CACAGTCCATATCATTACTCTTACACTGACAAAGTCTTCTTTTTGAAGATCCAAGAAACT
CCAAGGCCTAGGTAAGATTTTGTAAGACTTTTTGGGTTTCTTTAATTGACATAGACCCCA
GTCCTCTAATAGGGCGATGCATCCGGAATGGTCGGGATAGCTCAGCTGGTAGAGCAGAGG
ACTGAAAATCCTCGTGTCACCAGTTCAAATCTGGTTCCTGACACGCGGTTAATGTATCGA
ATGGATACTCATCCAAATGAATGGGTAAAGGAAAGAAGTAGATTTTGTTCCTTTTTTTTA
TACTGTACCCCCTCTCGCTCAAAAAGAATGTTAATACTTCATACATATCCAAAGTTAGGT
GGCTGAAACCAAAAAGTCTAGCCTAGGGGAGTTGAAGGATAGGAATAGACAGGATTCATT
TCAGATACAGTACAAAGAAAATACGATCCCTTTTCATTTCTGAATTTCATATTTTCTTGC
GTATTCTATTTCCTCACTCCCTCTTACGCGACTTCCAGGAGCCCATCCAAGTGATATGCG
CGGTACAAAGTTCATGGTACAGAACTCTTTTGATTCATACTATTGGCTTTACTCATCCGA
AATAGATATATTTAAAATTGGGGAATATCAACGAAGCCTATTTATTAGCTCATCCATAAT
ACGAATTAGAGCCCAGTTACTCTGTTTCATCTAGAACGTAAAAAGATTCCTTGAATATCT
GGAGTCGTAGAAGTGAAGATTAGTTTCTTATCATTCAATGAGCATCTTGTATTTCATAGA
AATTGGGGGCAATATAATCCTTACGTAGGGGCCATCCCACCCAACTTTCGGGCATCAAGA
TACGTTTCAGGCGTGGATGATTTTCATAAGAGATCCCCAACATATCATAAGATTCCCGTT
CTTGAAAATCAGCACTTTTCCAAATCCAGAAAACAGACGGGATTCTAGGATTCCTCCTTG
GAACAAATACTTTTATGCACACCTCTTCGGGTTGATCCACCCCATACTGTATTCTCGTAA
GATGATACACACTAGCTAAAAATCCGCCAGGTGCTACATCATAGGCACACTGGAAACGTA

GATAATTGTAACCATATACATATGAAATAACAGCAATGGAGTACCAATCCTCGGGCTTTA
TTTGTAAAGTCTCTCTTCCTTGGTAATCGAAGCCCAAAGATCTATGAACTAGCTCATGCT
TGACTAGCCAAGCAGATGAACGACCCTGCATCTTCTTGATCTCTCCCACATTTGTATGAA
TATTTTACATTTACGATGAATTTTATGAAAATTGACTCGCCGTTTGTTATTCCGCACAAA
AACACCCTGCCTAATTCATTAATTTGGGGGAAGATACTGAACTTTTGTATTTGAAAAATG
TTTCAGAAGGTATCTCTGAAGTAGATGGAGATTGGTAGAGTAATCCTTGATCGTAATTTC
CAGTATGAGTACTGTGTCCAACATGAAACTTGTGATTGGTAGTAAAATATCGATTTTCCT
GTTGAGACCCAATTCTATCTTCATAGATTTCTCGAGAGACTTTCTTACGAAGTTTCGTTA
TAGCATCTATAACTGCCTCTGGTTTAGGGGGGCAGCCCGGCAAATAGACATCCACAGGAA
TTAGCTTATCGACTCCCCGAACAGTACTATAAGAATCGGTACTGAACATCCCTCCTGTAA
TAGTACAGGCTCCCATAGCAATGACATATTTTGGTTCGGGCATTTGTTCATATAATCTCA
CTAACGAAGGAGCCATTTTCATTGTTACTGTGCCGGCTGTTAAAATTAGGTCCGCTTGCC
TAGGACTTGATCTTGGTACCAGTCCATAACGATCAAAATCGAATCGCGAGCCTATTAATG
AAGCAAACTCAATGAAGCAACAACTGGTACCATAAAGAAGCGGCCATAAACTAGAGAGTC
TTGACCAATTCGAAAGATCATTCGATGTAGTTGAAATAACTGAATTTTGGGCGGTTCGGT
CAAGTAACGGAAACTCAATAGAATTCATAACTGTTTCAATGTAATCTTTTCCTTCTTTTT
GATTTTGATTGTCTGAATATTCAGGAGCTAAGACCATTCCAATGCTCCTTTTCGCCATGC
ATAAACTGAACCAACAATTGGGATAAGCACGAAAATTAAAGCTTCTATAAACACAGATAC
ACCCAATACATCGAAACTCATTGCCCATGGATAAAGAAAGACTGTTTCAACATCAAAAAC
AACAAAAACTAGAGCAAACATGTAATAGCGGATTCGGAATTGTAACCAAGCATCCCCCAT
CGGTTCTATGCCCGATTCATAACTAGAGAGCTTCTCTGGTCCTTCACTAATCGGGGCCAA
AACTCCGGAAATTAGAAATGCCAAAATAGGAATAACACTTGATATTATTAGAAATGCCCA
GAAAATATCATATTCGTGAAGCAGAAACATAGAAGCACTCCTATTAATGTGGAATATACC
GAATTAGTTGATTCAAATTGGAATTCTCAATTCATCCATAACTGCATTAGTCGAAACAAC
AATTTTGATCAAACCACATAGTTTCGTTTGTTTACTTGTTGTGGGTCATGTATCGTCTCA
AGATTCATCCAACGGAATCCCACTTACACTTACTTCGATTCTATTTAGATATGGTGTAGA
CATATAATGCTATTATACAAATCAAACTCTCTCCTACCTTGCCTCGGGTTTTCTATCAAA
CAAAAAAAGGAATTAAGGAATTTTTTTTAAAGAATATTTTAAATAATATGAATTGAAATT
GAAATAATATTCAAACAATATTATTCAAATAAGATTAAATGAAATATTAAACATAAATAA
TTTCAATATTCTATTAATATAATAGAGCCAAAGAGGAGGTCTGGCCCATTTTTTCTCTCT
CTCTCTTTTTTTTTTTTTTCTTAGTGATTTAGAATATAGTCAGTAGTCAGATGTAATAGA
ATTTCTAGGAATTTCCATCTCGGGATTTATGGTATATTCTACGTGGCTGTGTTGGTGTAT
TCTTTTCATTAAGATCCGGATAGAGGATTATTGTTTCTATTGATTTGATACGGATACGAA
AACGGAATGCATCGACCGATTCGATTCTCTCTCCCTGTCCCAGATTTATACTTAATTGAT
TTGATTCAATCCATTGAATTGTGAGGAACCCTTACATATAAAAACTCATGGGTTCCTATA
CCCAAATTAGGAAACTTTGGACCTGTACTACCCCGGCCCCGGCTTTACCCCCGAGTTAGA
AGTCTTGAAAGAATCATTTCAGACCCATTTCTAGGACTAAAGATCGTGATTTGGAATGAC
TCGAAATACTTATTTATTTAATGTAATAATAACACCTAGCAGGACCAACCAACGAGTTAG
GTTTCGTGACAACAAAAAACGTTTCTTTTGAAGCAAACCTACAAAATGGGGCATAGTTTA
ATGGTAGAGTCGGCTGAATCGTAAATGATTTACAGAAGATACTTCGAATGGAATCATGCG
TTGTCGAACGATTCGATAGACAAAATCTCCCCATCCCAAAACCAACTCATAGAACATAGA
AATAGAAGAGGGTGCGTTGATACATTTAGGACCAATTCATTGTCTCTAAAACAATGAATT
GGGATTGTTGTTCTGCCAAAAGGCGATACGTCGGGGGATTCCTAACTCATCCAAGTTCAA
ATGGGCCCTAATCTTTTTAGATAAAGTCTGCATTGGTAGAATTGGAATGATGAACAAATT
GGATTGGGTAGATTGGAATAAAAAAAAGAAGTTATTAAGTATTGTACAGAAAAATGACTA
CTTGCTTTGCTAAGCCGGTTATACGAAGAAAAGCCTATTGTACAATGAAACTTACCAAAG
AGCTTCGTTTTTGAAACTCTGGCTTTTCTACAAATACAAGAACAAGAATAGGTTCTAGAT
AATGTGACTTACTATTAGATTGAATTTGGATTTGATTTGGTTGGGTCAGGTTGGAGTTTT
TCTTGAGCCAGGCTCATGTTATGATTTTGACTTCATAAATTGGCTTGGGTATACCAAAGC
AAAGGTGTATCACTAAATCTTGGATCATGGACAAATAAAAGAGGAAAAAGGCCGTATGTC
ATTCACAGACGAAGATTAATGAAGAAGAATGGGTTTGTTTATCCGAGATTTGAAAATACC
GATCCGATTGGATCCATTGGAATAAATTATTGTTTTCAAGCCCCGAGGGATCTCCGTGAT
CCTGTGGGAATGATTCCATTTCTATGGAACAATCAACCGGCCGGTCACACGCACTAATTA
GGAAATGAATACAAAAATGTATAGGGCTATACGGACTCGAACCGTAGACCTTCTCGGTAA
AACAGATCAAACTTATTATTATCGAAATGATTCGAACTGTTTCAAAGACCCAACATGCGT
TTTTTTTTTTGCATTGGGCTCTTTCATTAACTGATAAAAAGATCGGCTAGTCCACCATAT
TTTTTCTTGACAGGAAGATAACGAGATGGCTCCATGCGCTCGGATTCATTATTTGAATTC
TGATCCGGGAGCAATACCAAAGTGTTTCAAAGAAGGGTTACCCTGACGTAGGTCTGCCTC
CGGCCTAGATCAACCTAAGTTAAATGGAGTCTCTATCAATCCGCCCCAAGAGTCAAATAT
GATACTTAATACACCTTAAAGTTCATAGGACGAAAAGAGGTTATTTTGAGGTCCTTATCC
TCATTATGCCTAGCATTGAAGGGACTGGGTATTCACCTTATCAATGATCAAACCAATGAT
GGGTTCTATTTGGTACCTGAATTGGCACCTGAATCGGACCGAACAAAATATTTGTCAGGC
TATTGTTCTCTTGTTCCCTCGAATCCATGGAGTAAGACATCGATTTCTCAATAAGATCAA
TTCTGTTGATTGCATGATGGACTCCTCTGAAAAAGCATTGGCGCGCGTGTAAACGAGGTG
CTCTACCTAACTGAGCTATAGCCCTTGTCATAGACATATTAACATCTAGATAATTTCTTG
TCAAGATGGATATTCCATAATCCCACATGATAACTCTCCGATCCGTTTCCTGCCAAGGAT

TGGTATTGCTGAGAAGTAATATTCCGTCTATAATCCCCGATGTGATGGGTCCCATTTTTT
CTTTCTCTTTGTGATGATAAATGACCTACTTAACCCAGTGGTTAGAGTATTGCTTTCATA
CGGCGGGAGTCATTGGTTCAAATCCAATAGTAGGTAGAACTTATTAGATACCGGAGTCGA
TGGTATCTAATAAGTTTTTCTACCCACCTTCTTCTCTTTTTTTTTTATGGATTTTGTACC
CTTTCCCTATTATACCCCCACTACTCATATTTGTATTTGTTTTTTTTTTGTTCGTTACAT
CAGATTACAATTGATTGTATCCAATTGGCGGAATCCAAATATGGTGTATAAACAGAACTT
CTTTTGATTATTCTGATACATTGACTAGTACGAAATAACATTGATAGCCTCTACTCGTGT
CCTAGCTCGTCTAAGAGCTAGATTCGCCTCAATTGCTTGTCTCTTGCCTTCAGCTCTACT
CAAGTTAGCTTCAGCTATTTCAAGAGTTCGCTGAGCTTCTTGTGGATCAATGTCACTACC
CTTCTCTGCATCATTTACTAAAATGGTGATCTCATTATTGCCTATTCTAGCGAAACCGCC
CATCACAGCCATCGTTAACCATTGGTCGTTGAGGCGTATTCTCAAAATACCTATATCTAC
GGCTGTGGCAATAGGGGCGTGATTTGGTAATACGCCAATTTGGCCACTATTAGTAGATAA
AATGATTTCTTTCACTTCCGAATCCCAAATAATTCGATTCGGAGTCAGTACACAAAGATT
TAAGGTCATTTCTTCAATTTGCTCTCCACTTCTAAGTTTATAGCCTTCGCAGTAGCTTCA
TCAATGTTACCTACCAAATAAAAGGCCTGCTCGGGAAGACCATCTAATTCTCCGGAAAGG
ATCAGTTGAAACCCCCTAATTGTTTCTGTAAGACCAACATATTTCCCTGGAGAACCAGTA
AATACTTCTGCTACGAAGAAGGGTTGTGATAAGAAACGTTCAATTTTTCGTGCTCTTGCT
ACGGTTAAACGATCCTCTTCAGATAATTCGTCCAACCCAAGGATAGCTATAATGTCCTGA
AGTTCTTTGTAACGTTGTGAAGTTTGCTTAACTCTTTGCGCAGTTTCATAATGTTCCTCA
CCAACGATCCTAGGTTGTAGCATAGTTGACGTTGAATCTAACGGATCTACTGCTGGATAG
ATACCTTTGGCAGCTAATCCTCTTGATAGTACGGTAGTAGCATCTAAGTGCGCAAATGTC
GTAGCAGGAGCAGGGTCGGTCAAATCGTCCGCAGGTACATAAACTGCTTGAATGGAAGTT
ATAGACCCCTCTTTGGTAGAAGTAATTCTTTCTTGCAAAGAACCCATTTCTGTACTAAGG
GTAGGTTGATAACCCACAGCGGAAGGCATTCTACCTAATAAGGCGGATACTTCTGATCCT
GCTTGGACGAAACGGAAAATATTGTCGATAAATAGAAGTACGTCTTGTTCATTAACATCC
CGAAAATATTCCGCCATGGTTAGGGCAGTCAAACCAACTCTCATACGAGCTCCCGGCGGT
TCATTCATCTGTCCATGGACTAGAGCTACTTTGGATTCTGCAATATTTTGTTCATTAATC
ACTCCGGATTCTTTCATTTCCATGTAAAGATCATTTCCTTCACGAGTACGTTCGCCTACT
CCGCCAAATACAGATACACCTCCATGAGCTTTGGCAATGTTGTTGATCAATTCCATGATG
AGTACTGTTTTACCCACCCCAGCTCCCCCGAATAGTCCGATTTTTCCTCCACGACGATAA
GGGGCTAAAAGATCTACCACTTTAATCCCTGTTTCAAAGATTGATAATTTGGTATCTAAC
TGGATAAAAGCAGGCGCAGATCTATGAATAGGAGATGTTGTGCGAGTATCTACAGGACCT
AAATTATCAACAGGCTCTCCAAGAACGTTGAAAATTCGTCCTAGAGTAGCTCCACCCACT
GGAACGCTTAGAGGAGCTCCCGTGTCAATCACCTCCATTCCTCTCATCAGACCATCTGTA
GCACTCATAGCTACAGCTCTAACTCGATTATTTCCTAATAATTGCTGGACCTCACAAGTC
ACATTAATTTGCTGACCGACAGTATCTCGACCCTTAACTACCAAAGCGTTGTAAATATTA
GGCATCTTGCCGGGGGGAAAAGCTACATCCAGTACCGGACCAATGATTTGAGCAATACGC
CCCAGGTTTTTTTCTTCAAGTGTGGAAACCCCAGGCCCAGAATTAGTAGGATTGATTCTC
ATAATAATGAAAGTGAAATATGTCAAAATTTTTTGCGAATATTACCGAATCGAAAATAAA
TGTCCGATAGCAAGTTGATCGGTTAATTCAATAAATAAGAAATGGGAGTTAGCGCTTGAT
TTCGTTGGTACCATTCAACTGAATCCAACTCAATCGTTTACTCATTCACTAAATGAATTT
TCAAGTTCAACCAACCCTTTTTCAAAATATCTATCAAATCAAGTAGATGAATAAGAATCA
TGGGGAAGTCTTTCATTTTTCTATCATTATAGACAATCCCATCCATATTATCTATGGAAC
TCGAACCTGAACTTTATTTATGATTCAGAATTTCTATCTTATTGGCCGTTGTTCCTTATT
TCAGCATATTAGTTTCCGCCTATTCTTGTTTTTATTTTTTATACCCTTTCATGGATGAAT
TCTGCCTATTTTCACATCTAGGATTTACATATACAACATATATCACTGTCAAGGGTGAAT
TTCTTATTATTTAGATTCAAAAAAAAAGAAGGAGATCCAAACTTGCAAAACAAGGATTGG
GTTGCGCCATACATATGAAAGGGTATACAATAATGATGTATTTGGATGTATTTGGCAAAT
CAAATACCATGATAACGAACCATTCTAATTAGTTGATAATATTAGTTGAGAATTTTGTGA
AAGATTCCTGTGAAAGGTTTCATTCATTACTAATCCATGTCGAGTAGACCTTGTTGTTGT
GAGAATTCTTAATTCATGAGTTGTAGGGAGGGACTTATGTCACCAAAAACAGAGACTAAA
GCAAGTGTTGGATTCAAAGCTGGTGTTAAAGATTACAAATTGACTTATTATACTCCTGAC
TATGAAACCAAAGATACTGATATTTTGGCAGCATTTCGAGTAACTCCTCAACCCGGAGTT
CCACCTGAGGAAGCAGGGGCTGCGGTAGCTGCCGAATCTTCTACTGGTACATGGACAACT
GTGTGGACCGATGGACTTACCAGCCTTGATCGTTACAAAGGACGATGCTACCACATCGAG
CCCGTTCCTGGGGAGGAAACTCAATTTATTGCCTATGTAGCTTACCCCTTAGACCTTTTT
GAAGAAGGTTCTGTTACGAACATGTTTACTTCTATTGTGGGTAATGTATTTGGGTTCAAA
GCTCTACGAGCTCTACGTCTGGAGGATCTGCGAATTCCTCCTGCTTATTCCAAAACTTTC
CAAGGCCCGCCCCATGGCATCCAAGTTGAGAGAGATAAATTGAACAAGTATGGTCGTCCC
CTATTGGGATGTACTATTAAACCAAAATTGGGGTTATCCGCCAAGAACTACGGTAGAGCG
GTTTATGAATGTCTCCGTGGTGGACTTGATTTTACCAAGGATGATGAGAACGTGAACTCC
CAACCATTTATGCGTTGGAGAGACCGTTTCGTATTTTGTGCCGAAGCAATTTATAAAGCG
CAGGCCGAAACAGGTGAAATCAAAGGACATTACTTGAATGCTACTGCAGGTACATGCGAA
GAAATGATCAAAAGGGCCGTATTTGCCAGAGAATTGGGAGTTCCTATCGTAATGCATGAC
TATTTAACGGGGGGATTCACTGCAAATACTACCTTGGCTCATTATTGCCGGGACAACGGC
CTACTTCTTCACATCCATCGCGCAATGCATGCAGTTATTGATAGACAGAAGAATCATGGT

ATGCACTTTCGCGTACTGGCTAAAGCGTTACGTATGTCTGGTGGAGATCATGTTCACGCT
GGTACCGTAGTAGGTAAACTAGAAGGGGAACGGGACATCACTTTGGGTTTTGTTGATTTA
CTACGTGATGATTTTATTGAAAAAGACCGAAGTCGCGGTATTTATTTCACTCAAGATTGG
GTCTCTATGCCAGGTGTTCTGCCCGTGGCTTCAGGGGGTATTCACGTTTGGCATATGCCT
GCCCTGACCGAGATCTTTGGGGATGATTCCGTACTACAGTTCGGTGGAGGAACTTTAGGA
CACCCTTGGGGAAACGCACCTGGTGCAGTAGCTAATCGGGTGGCTGTAGAAGCGTGTGTA
CAAGCTCGTAATGAGGGACGTGATCTTGCTCGTGAAGGTAATGAAATTATCCGTGAAGCT
GCCAAATGGAGCCCTGAGCTAGCTGCCGCTTGTGAGGTATGGAAGGAGATCAAATTCGAA
TTCGCCGCAATGGATACCTTGTAATCCAGTAATTCCCGTTCGTTCCCCTAATTGTAATTA
AACTCGGCCCAATCTTTTACTAAAAGGATTGAGCCGAATTAAAGAATGAGGATCCTATGT
ATATGGATAGATATAGATCTTGTATCTATCAATATGTGCCTACTTTACCTAGATATACAA
GATCTAAATACAAGATAAGATCTAAGACTAAACAACTCAATGCTTCTATTGTTGGATCCA
TAATTAATCCTATGGATCCTTAGGATTGGTGGATCCTTTTCTATCCCGTTGTTTCGGACC
ATAGATCGAGCCAAGGGTCACAACTTCTTCTACTCATCCTGTATATTGTCCTTTTCATTC
CGTGTTGCATTAGAAACTTATTATTATACGAGATTATACGAAAATGAATCCTTCCTAGGA
GGGAACAAATATTTCTCTTTTCGATGAGAGTTTGTACACAACATGGGAGAAACCTATCTT
CTATTTATAATAATTGAAGAAAAGGTTCCATCATATCATATATAGTGAATTGATACTCCC
GATTCCCACAAAATCATTTCTTTCGTTCAATAGTTACTCGTTATTAGTTAATAATCCTAG
TGATTGGATCTATATGCGTATTCCGATAGGAAATGAAATAGTAAAATGATTTTTCGTCGA
ATGACTATTCATTTATTGTATTTTCAAATAGGGGGCAGGAAGGATCTATGGGAAAATGGT
GGTTCAATTCAATGTTGTCTAACGAGGAGTTAGAACACAGGTGTGGGCTAGGTAAATCAA
TGGACAGTCTTGGTCGTCCTGTTGGAAATACCAGTGGAAGTGAAGATCCCATTCTAAATG
ATACGAATAAAAACAATCATAATCATGGTTGGCGCGAAAGTAATAGTTGCAGTAATGTTG
ATCATTTTTTCGGTGTCAGAGACATTTGGAGTTTCATCTCTGATGACACTTTTTTAGTTA
GGGATAGTAATGGTAACAGTTATTCCGTATATTTTGATATTGAAAATCGGGTTTTTGAGA
TTGACAATGATAGTTCTTTTCTGAGTGAACTAGAAACTGCTTTTTCTAGTTATCTGAATA
GCGGGTCTAAGAGTGACAATCGCTACTATGATCATTATATGTATGATACTACGTATAGTT
GGAATAATCACATTAATAGTTGCATTGATAGTTATCTTCGTTCTGAAATCAGTATTAATA
AGTACATTTCGAGTGGTAGCGACAATCCCATTTACAGTTATATTTATAGTTACATTTGTA
GTGGTGAAAGTGTAAGTGATAGTGACAGGGGGAGTTCTAGTATAAGAACTGGCGGTAATG
GCAGTGATTTCAATATAAGAGGAAGATCTAATGATTTCGATGGAAATAAAAAATACAGAC
ATTTATGGGTTCAATGCGAAAATTGTTATGGATTAAATTATAAGAAATTTTTTAGGTCAA
AAATGAATATTTGTGAACAATGTGGATATCATTTGAAAATGGGTAGTTCAGATAGAATCG
AACTTTCGGTTGATTCGGGCACTTGGGATCCTATGGACGAAGACATGGTCTCTATTGACC
CCATTGAATTTCACTCGGAAGAGGAACCTTATAGAGATCGTATCAATTCGTATCAAAGAA
AGACAGGTTTAACTGAGGCTGTTCAAACAGGCATAGGTCAACTAAATGGGATTCCCATAG
CCATTGGGGTTATGGATTTTCAGTTCATGGGGGGTAGTATGGGATCCGTAGTAGGCGAGA
AAATCACCCGGTTGATCGAATATGCTGCTAATAGATCTCTACCTGTTATTATGGTGTGTG
CTTCTGGAGGAGCACGCATGCAAGAAGGAAGTTTGAGTTTGATGCAAATGGCTAAAATAT
CTTCCGCTTTATATGATTATCAATTCAATAAAAAGTTATTCTATGTATCAATCCTTACAT
CTCCTACAACCGGCGGAGTAACGGCCAGTTTTGGTATGTTGGGAGATATTATTATTGCTG
AACCCAATGCCTACATTGCATTTGCGGGGAAAAGAGTAATTGAACAAACATTGAATAAGA
CAGTACCTGACGGTTCACAAGCAGCTGAGTATTTATTCCATAAGGGCTTATTTGATCCAA
TCGTACCACGTAATCCTTTAAAAGGTGTTCTGAGTGAATTATTTCAGCTACACGGTTTCT
TTCCCTTGAATCAAAATTCAAGTAGAGCGCTAGGCTCAGTTATTTGTAGCGAACTTTAGT
TCATCGGAATCAAAGTCAAAATAAGAAGAGTGGAGTTTTCTTTGGTAACATAACTTCTAT
AGGAAGTTTCGGATAATTACTTTTTTTGATGCAGATTTTTTATCCTACCCCTATTCATGA
TTAGTAATCAGGAACCCCCTATCAGGAGGAAAAGAGTGAATTCTTCCTTCCGCGGAATGG
AATTGGGAAAAAAAATCAAAAGAATTTCATGTTCCCTTCTTTCATATTAATATATATCGT
ATTAATATATATAGAATAATTCAAATCTATAAGGGAAGTGTTGCATATTTTTATATCTCC
CGAGGACCTACACTTTTGACTATGAATTCCTGTTGGATCGGATTCTAACAAATCCTTAGG
AAACTCGTAAGAAACTCTTTATTAGAAGATAAGGGCGGTAGAACAAGAATAATAAAGCGG
ATTATCATCCATCTATTTCTTGTAGAAAGGTGAATAGATACACTTATTTAGCTCTACATT
CCTTGCACTTATTATATACTTAATAATACTTATAATAGATATACTTATCATAAGATAAGA
TATCTTTATAACAGGTACAAATATTAAATCGAGGCACCCATTCTATGACAGATTTCAATT
TACCCTCTATTTTTGTGCCTTTAGTGGGCTTAGTGTTTCCAGCAATTGCAATGGCTTCTT
TATCTCTTCATGTTCAAAAAAACAAGATTGTTTAGACCTGATAGGACAAAATTTCATCAA
TTTATTTCAACACTTGGACTTGGATCATAATAGGGATATCCATTTAGTGGAATATGATAC
GACATGTGGCCCCCTCCGGGCACAAATAAAAAAGTGATTATACATGCGGATACATTATAT
ATGGATAAATGCATGTATATGGGGGGATAGCTGTTTTAAAATGGATCAGAGCGGATATCT
GAAATAGAAAGTTAACGTATCTATATTATGTAGATATACATAGTGGTGGTATTATACGAA
GGGGATGTTATTATTTTATATCTAACCAATTCGATGAATTACTCCTAATAGTTCGCGTCA
TAATAGTGCTAGTTGATGAGAGTTACTTCGGGAGCAAAATAAAAAAAGTAAAATCAAATT
CATTTGGCTTATTCTCTTCTCTCAATTCCAATAGGATGCAACTGAATCTAGTATGAACTA
TCGATCAGAACGTATATGGATAGAACTTATAACGGGGTCTCGAAAAACCAGTAATTTCTG
CTGGGCCTGTATCCTTTTTTTAGGTTCACTAGGATTCTTATTGGTTGGAACTTCCAGTTA

TCTTGGTAGGAATCTGATATCTTTATTCCCGTCTCAGCAAATCATTTTTTTTCCCCAAGG
AATCGTGATGTCTTTCTATGGGATCGCAGGTCTGTTCATTAGTTCCTATTTGTGGTGCAC
AATTTCGTGGAATGTAGGTAGCGGTTATGATCGATTCGATAGAAAAGAAGGAATAGTGTG
TATTTTTCGTTGGGGATTTCCTGGAATAAATCGTCGCATCTTCCTTCGATTCCTTATGAG
AGAGATTCAATCGATCAGAATGGAAGTTAAAGAGGGTCTTTATCCTCGACGTGTCCTTTA
TATGGAAATCAGAGGCCAGGGCGCCATTCCCTTGACCCGTACTGACGAAAATTTGACTCC
ACGAGAAATTGAACAAAAAGCTGCTGAATTGGCCTATTTCTTGCGCGTGCCAATTGAAGT
ATTTTGAAATGAAGGGAATGAATGCTTTCTCAGCATGAGGGAAGGGACCCAGGAACCCCC
TTTTAAATATAACTGAAGCTTCTTCGGAACGTTCATTCGAGCAAAACATGTTAGATTCTA
TTTCCCCCGTTCCGTTGGTAATCCTTCTGTGGCCCATAGAATAAAGCAGGCGGACGTATA
CGGAACAACCATAATAAGAAGCAATTTTGATCGACCAAAACTCCTTTTTCTGCATATAGA
ACTCAATTCTACTAGTAACAAGTCTAATAAGTATGTATTCATCACACATATCAGAGCATT
TCGGAATACATAATTTCTCTTTTTAGGACCAATACTTTGGATTAATACATTAGATACAGA
TGTATCATATCCCGTTAATTATCTTTCTTTTGTCAATCGATGTTTCTTTTTTGATCCTTT
CTTTAGCTCCTGGATAACCAAACGTTTAGATCTCTCATAACTATCCAATTTCTCTCTCGT
TTTGTCACCTATTTCGGATTTCATCATTAATATTTTTCAGAAATATCCCCTCAATTATTC
CCGGGTCGTGGGTAGCCAGTGAAAATTTCGAAAAAATAATTGAGGGGAGTTCTTTCGTCT
CGAAATCAAAATAATATTCATTATTTCAAGCGGTTCTTTTGGGCATTCATCGAAAGAACA
AATGAAGATAGAATTGGTTCGAATTTGACCAACTGAGATATCTGGGAAAAGTATTTGATT
ATTTCTTCATTCGAAACGGGCCCTTATTCTATTTCTATTTCTATATTCTTTCTAGATCCA
AGGACTAAACAATTCAAAAAAAAAAGGAATAGATCCATAGGTTCCATACCTTGTTATAGA
ACTCATGCTTCATAGAAATATCGGATCAGATAGAGTCGGCGAATGAAGCGGGTTCATTAA
CAATTCACAGATGAAAAGTGTCAAAAAAGAAAGCATTGACTCCCCTCCCGTATCTTGCAT
CTATAGTCTTTTTGCCCTGGTGGATCTCTCTCTCATTTAATAAAAGTCTGGAACCTTGGG
TTACTAATTGGTGGAATACCGGACAATCCGAAACTTTTTTGAATGATATTCAAGAAAAGA
ACGTTCTAGAAAGATTCGTAGAATTAGAACAACTATTCCTGTTGGATGAAATGATAAAAG
AGTACCCGGAGACACAGATACAAAAGCTTCGTATAGGAATCCACAAAGAGACGATGCAAT
TGGTCAAAATGCACAACGAAGATCATATCCATATCATTTTGGATTTCTCGACAAATATAA
TCTGTTTTGCTATTCTAAGTGGTTATTCTATTCTGGGTAATGAAGAACTTGTCATTCTGA
ATTCTTGGGTTCAGGAATTCCTCTATAACTTAAGCGACACAATAAAGGCTTTTTCTATTC
TTTTATTAACTGATTTATGTATCGGATTCCACTCACCCCGGGGGTGGGAACTAATGATTG
GTTCGGTCTACAAAGATTTTGGATTTGCTCATAACGATCAAATTATATCTGGTCTTGTTT
CCACTTTTCCAGTCATTCTAGATACAATTTTGAAATATTGGATCTTCCATTATTTAAATC
GTGTATCTCCTTCACTTGTAGTGATTTATCATTCAATGAATGAATGAAGAACTCATTTGA
TCTGCTGATATCAATCAAATCATGATGCTACTTCGTACATAAACAAACTGTTTTGAAGCT
TACTCACTCTTTATACTTCTACCCGCCCAGGGGGTTCCTACTATACTTCAGTACAATTAT
TCCAGTACAATGGCAGAATCATGGATAGGGAACTATGCTAGCTACCTACCTAATTTATTG
TAGAAATTTCCGGGATCAATTATTGGACCATGCAAAATAGAAATACCTTTTCCTGGGTAA
AGAAAGAGATGACTCGATTCATTTCCGTATTGATCATGATATATGTAATAACTCGGACAT
CTATTTCAAATGCATATCCTATTTTTGCGCAGCAGGGTTATGAAAATCCACGAGAAGCAA
CTGGTCGTATTGTATGTGCCAATTGCCATTTAGCTAATAAGCCCGTGGATATTGAGGTTC
CACAAGCTGTGCTTCCTGATACTGTATTTGAAGCAGTTGTTAGAATCCCTTATGATATGC
AAATGAAACAAGTTCTTGCTAATGGTAAAAAGGGGGCTTTGAATGTGGGGGCTGTCCTTA
TTTTACCCGAGGGATTCGAATTAGCTCCCCCCGATCGTATTTCTCCCGAGCTGAAAGAAA
AGATGGGCAATCTGTCTTTTCAGAGCTATCGCCCCACTAAAAGAAATATTCTTGTAGTGG
GTCCTGTTCCTGGTCAGAAATATAGTGAAATCGTCTTTCCCATTCTTTCTCCGGACCCTT
CTACTAAGAAAGACGTTCACTTCTTAAAATATCCCATATACGTAGGCGGGAACAGGGGAA
GGGGTCAGATTTATCCCGACGGGAGCAAAAGTAACAATACAGTCTATAATGCTACAGCAG
CAGGTATAGTAAGCAGAATAGTACGTAAAGAAAAAGGGGGATATGAAATAAGCATAGCCG
ATGCATCGGATGGACACCAAGTGGTTGATATTATACCTCCAGGACCAGAACTTCTTGTTT
CAGAGGGTGAATCCATCAAGCTTGATCAGCCATTAACGAGTAATCCCAATGTGGGTGGAT
TTGGTCAGGGAGATGCAGAAATAGTACTTCAAGATCCATTACGTGTCCAAGGTTTGTTGT
TCTTCTTGGCATCTGTTATCCTAGCACAAATCTTTTTGGTTCTCAAAAAGAAACAGTTTG
AGAAGGTTCAATTGTCCGAAATGAATTTCTAGATCCACGGATTCATCAAGTTGATAAAAA
GGGCCGAATTATTGTTGATCAATGCAATTATGTATGATCCAAAAAAATATGGAAAGCCCC
TTGTCTTGCTTTGTTTATCCTGCTTTTCTGCGAGATGCCGGGAATTGCTTGTATCCCATT
CCCAGTAATAGTATGTATATTGCGAAGAAGACTACTTGACCCTCCCCCTTTTTTTTTTTT
CAATCCAAATTGGAGCGGTGTGACGCTTCTTATTGCCAGATTGTAAATGCCACGGAATGC
ATCAATAGTTTTTTCTATCTAATAGAATCGAATTCTAATAGACAATAGGCGGCATAACAT
TAAGTAGGAAGAGAATACGCGGCAAGGGATAAATGAAAGAATGATTCTGGGAGGGATTAC
TTGTCTTCCTAATTTTCGACACAAGAAAAGGGCGGAAAATTCCTTTTCTTGTGTCGAAAT
AATAATGATTCTTGATCTTGTTCGTCAAAGATTACTGTTTTCTTTTCCAGGTCTATCGGA
ACCTCTTTCTTTAGATTCATAAGAAGTGGCGGACAAACAAAAAAGGGGGATGGCTTAGTA
AACAAATAGAACTTCTTCAACGAACTTATAAAATTTCAAGTAAAAAAAAAAATAAAATTTT
AAGATGAGATAATAAATAAGGATTTGGATATGTGCAAAAATCCAAGATTTTCCCCTTTCA
ACCGGAACATTAAGAGTCTTTTTTTACTTGATGAAATTTTATTTTTATAGA

ATAGAGTAGAGTAAGGTTCAATTAAATTAGTATAGAAATGGTTTGCAGGATGTCTCATCT
GTAGAAATCCTGTGTCATCCAAAAAATAAATTGATTCCTTCTTTCTTCTTGTTTCGGAAG
GGGCCCTCATACTATGGCGGAACAGATACTATGAATCAATCAAGGGATTCCATTTTTCAA
AAACATCATCAGAAACAAAGCATCCTTTTATCATTTCTATGAATCTAATATTATGATTAT
GTTGACTGGATGAATTTCCAACTTTTTTGATGTTATGGAATAGATCAAACAAAACCTTAC
CCGAAGAGTAAGAACTCAATAGGACCTTACCCCTCTTTGTCTGATTCGGGGGGTAAGGTC
CTATTGAGTTCTTACTTTTTCATGTCTACAATCCGGCTCATCCGATTACTATAGGGATGA
TCCCAATCCGGAATATGAGCCGTAAAAGAAAATACCTATTGAACCGATCACAGGAATACC
AGTTACAGTACCTATAAGCCAAAGAGGAATCCTTCCAGTAGTATCGGCCATTTACCCACT
TCCCTCCACATTTCATCAAGTGGTCGTGCTAGAGACATAAACAGTCATGGATAATTATGA
GGATGATATCCTTCCGAATGGGATAAGAGAATTCCTACTCTTTTTCTTTATTTTTTCTCA
ATTGAAGAAATAATTGGAAAATAAAACAGCAAGTACAAAAATGAGTAATAACCCCCAGTA
GAGACTGGTACGATTCAATTCAACATTTTGTTCGTTCGGGTTTGATTGTGTCGTAGCTCT
ATAATTCGGATTAGGTTTATCGTTGGATGAACTGCATTGCTGATATTGACCCCAAAAAAG
AAACAGTAGGTACAGCTAGTCCGTGAACAGCCAACCATCGCACTGTAAAAATTGGATAGG
TTCGATCTATGGTCATTGAGGCCTCCTAAAAGGATCTACTAAATTCATCGAGTTGTGCCA
AAGGATCAAAACGACCAGTTATTAATGGAATTCCTTGCCGGCTCTCTGTGAAATATTCGT
TTGGCCGAGGGCTTCCAAACACATCGTAAGCTAAACCTGTGCTGACGAATAACCAACCCG
CAATGAATAGGGAAGGTATAGTAATACTATGAATGACCCAGTATCGAATACTGGTAATAA
TATCAGCAAAAGAACGTTCTCCCGTGCTTCCAGACATGCTGAGCTCCACATATTCTTGTA
CAGTCAAACAGAGGATCGATTCCGTGAAAGATGGGATCAGTAAATGGAAAACTACTGATA
TTTCATCCTTGTGAGATCGTCAATAGTGTACCGAAGGTGTATTTAGAGTATACCGAATCA
GTATAGCTATCCTTCCTCTGACACAGCAATGCATTTTCAATCAGTATCGAAAAGAAATGG
AATTCCTTTCTTCCTTTCTTGTTCCTTGTCTATGCAAAACCGCGTGTCATTCAATAGAAA
ATTCTTAAATACCTGTAATATAGGGTTTCCTTTACTGGCTTCGGAATAGAAACTGAAGAT
CTTGGTAAAGTATGAGTCGACGGGTTCTAATAATTCATGATTATTATATTTCCACAATTC
AATTAGATGCAAAATTTAGAAACCCCTTTTCATGGTTGTCGAAAAGGTATTTTTTTTTTC
TAATCCTTTCATTTAAAGTAATTGGTTGGTCGTACAGTAGTAGACAGTAGTAGGTAGTAG
TAGATGGAAAAAACAGAACAAACAGTAGTTGGAACAATTATCAATATTTGTGATGCAACC
ATTGCTGCATTAGACCCAAAGGTTCCTTCTTAACCCAGCTACAAGGATGGGACTGAACTT
TATGATATGGAAAGACAGAGTGCGAAACCTAAACTAAAAGGATAATAGCAATTGCTAGTT
TTAGAATGAAGTTGGGCTCGAAATAAAGGTTTTATTTCTTCGAGAAATCATGGGATACTT
TTCATTTTTCTTCTCGTTCGAAATATTATGTGCAATTAACCAACCTACTACTGAATACAA
TTAAGTTAAAAAGTCAAGTAAAAGCCGTTATCCGGCTGTTTGTTCCAAAATGGATTAGAT
AAATTGAAAAAGAAACGAAATGATCAAAAAAATGGAATTTTAAAATCCAATTCTTTATAT
TCTGATAGTTACTCAAAGAGAATTTCATTTTTGAATTGAATGAAGTTACAAGACACAGTT
CTTATTATTAGTACTTTACTCACGGGTTGCTCACTGAATCTGTTAATTCGGAATCATGGG
ATCTGTAGATGTTACAGGCGACGAATCCATCTTTTTTTTCTACCCCTCTTACTCTCTCTT
TGTTAGTGCCGTCTATAATGGATGATGAATCAAGAGCTTTCAATTGGAACTGATTCTGTC
AATTGGTATTTTTTCTTGTCATTGTATCTCGCAAAAATGGAAACTTAGGTAAGTGCTTTA
GAACCCTATGTATGAAAAAGAAGATATCTCATTTAGCTCCTCCATGACTACTATAACTAG
TTATTTCGGTTTTCTACTGGCTGCTTCAACTATAACCCCAGCTCTATTGATTAGTCTGAG
CAAGATACGACTTATTTGAAATTAATTGAATGAACAATTCATAAAAATGAATATTTCTGT
GAGATTCCCGGTATTCTATAGTTCCTTCCCGCGTTAATTGCCAATTCTTGGTTATTGAGA
TTCATGGGCGATTCGGATTAATATTTAGGGACAGATATTACCTCTCTTTTTCTATTCTTT
CAAAGAAATTGAAATGATTGAAGTTTTTCTATTTGGAATTGTGTTAGGTCTAATTCCCAT
TACTTTGGCCGGATTATTCGTAACTGCATATTTACAATACAGACGCGGCGATCAGTTGGA
CCTTTAATTGAGTAACATCTCTTTTTTTTAATTGACCTCCTCCTTAATCTCCAGGAGGTC
AAATTCAAGTTGCAGTTCAAGTTAGTGAAGTTATTTTATTGTGATTCAACATTAAAAGAA
CAGAATCACGCTCTGTAGGATTTGAACCTACGACATCGGGTTTTGGAGACCCACGTTCTA
CCGAACTGAACTAAGAGCGCTTTATTATGATGGGAGATACGGATGTCAAGAAAAGGATTC
TTTTTGTACCCCCAATACATCTTGTATGTATAGTATCATAAAATGGTAGATTGTGTCCAA
TTTTAATCGATCTCAATTGACCCCTCGTTACTGTCCATAGGAGAAGTAATAGGTAGGGAT
GACAGGATTTGAACCCGTGACATTTTGTACCCAAAACAAACGCGCTACCAAGCTGCGCTA
CATCCCTTTCATTTGTTGTACAGTGCCATTGTAGGGAATCCATGTTTTGTTTTCCACA
TCATAATTTCCTCTATCTAAATAGAATTTCTTTTGCCATTTCTTCTTTTTGGTTTTTTGG
TTTCATTCTCATAAAGAATTATATACATACCTAACGTATAAACGTATAAAGGAATGAAT
ATTTATCAGTAGTGCTCAGGAAGGAGGGTTCATCTTTTTCTGTTTTAGGGACAGGTAGAT
TTCATCTACCGGATCGTTGTATATATCCATTTTGGTTAGAGATTCCCCGTACAAATGATC
TTTAACTACATATGCATCTGATCATATATGTATTACAATATACAATAAAGTCAATAAAGT
GAACTTTAAAGAAGGAGGATTTTCAATGCGAGATATAAAAACATATCTCTCCACGGCACC
TGTGCTAACTACTCTATGGTTCGGGTCTTTGGCGGGTCTATTGATAGAGATCAATCGTTT
ATTCCCAGATGCGTTGACATTCCCCTTTTTTTCATTCTAGTTATTGACATGGGAAGGGAT
CAAGAAGATTAGAGATACAATAAATTCTCTGTGACTAACCCCCCCCTTTTTCAGTTCTT
TAGGATAGGAAAGAAAGAGTAAAGAATAAAAGTGGATTGAATCTCATCGAAACTCGGGTT
CGGGTTAATATAGGGAGAACAGAAATGGAAATGTGGGTCGAGGGCAGGCTGTTCAAGATC

ATACAAGATACTAAATGAAATACTGGGATTGGGAATAATTGATAGTTAGAAATATTTGTA
TTACTTAATAATTTGATTACTCTATTGATTGCAACGAAATCTTTCATAATTGAATTGGAT
TTCGAGTTAGCAACCTCTCGTCTATTTATTTTTCATTCCTTTCTTCTTCGCTTCGGTTCG
AATCGAAAATAGAAGAATTGAGTGAATTCAAAATCCAAAGGAGGTTCATGGCTAAGGGTA
AAGATGTCAGAGTAGTAGTTATTTTGGAATGTACCAGTTGTGTCCGAAATGGTTTGAATA
AAGAATCGCGGGGCATTTCCAGATATATTACTCAAAAGAATCGACACAATACACCTAGTC
AATTGGACTTGAAAAAATTCTGCCCTTATTGTTACAAACATACGATTCATGGGGAGATAA
AGAAATAAATCGAACGGAACGCGTGTGCCACTCTTCCAAGGAAGAGGAAGAAATTACATA
TATATATATATATATATACAAATCCAGTCCTATTTTGGTCGGATCCGAGATGAATGAA
GAAATAGGATTTTAGAAATAAGAAATAAACCATGGATAAATCCAAGCGGCCCTTTCATAA
ATCCAAGCGATCTTTTCATAGGCGTTTGCCCCCAATTGGATCGGGGGATCGAATTGATTA
TAGAAACATGAGTTTAATTAATCAATTTATTAGTGAACAAGGAAAAATATTATCTAGACG
AGTGAATAGATTAACCTTGAAACAACAACGATTAATTACTATTGCTATAAAACAAGCTCG
TATTTTATCTTCGTTACCTTTTCTTAATAATGAGAAACAGTTTGAGAGAACCGGGTCGAT
CCCTAGAACTACTGGTCCTAGAACCAGAAATAAATAAGCCTATTCCTCTCAAACGAATCA
AAACTCTAATTCGAACTCAGATTGAAGTTTTGTTCGAAAAAGCCGAGAGATTGTCGCGCC
GTAATGTAATAATAAAAAAAAAGAATGGGGGAAGAATAAATCTTTTTTTTTTTATTGAAAC
GTGTTCGTTCATTCCTACTACTTATCTTATCATACGAATTTCTACTATACCCTCCCGGAG
TTCATTCTCCGGGGAACTCCGTTTAAAGTATTCCAGTGAATTCCTTCCAATCTCCTTATT
TGATGATCGCATTGGAAATCGTGTCAAGACAATTCCTATTTGATATGGCTATTTGTGCAG
GTATTTTACGATTAAGAAGCAACTGCCTCTTGTACAGATCAAGTATTAATCGACTATAAC
TATGGGATCCCCTATTCTCACGAGTTACTGCATTTATCCGAGTGATCCACAAACGACGAA
AACTTCTCTTTCGCCTGCCTCTATCCCGATGAGTGGAAACCAAAGCTCTCATTTTCTGTT
GAGTAGTAGTTCGAGTAAGTCTTGAATGAGCCCCTCGAAAGGTTGCTGCAAATAAACGAA
TTTTTGTTCTACGTCTCCGAGCTATATATCTTCGTCTAACTCTGGTCATTGAATCAAAAG
AAACTTTGATGAATAACTAATTGATTTCTTTTCTTTCAGTCATTCTTTTCCCCTCTCCTG
GTTTATTAATAACAAAACGGATTCTTCCGATGTATAAAATTAAAATACAAATTCCAATGG
CTTTTGCTACTATAACCTTCCCAACCACGATTTTTTTATTTCTTCCAGGCATTTCACCTC
AAAAAAAAAAAAAGAAATTGTACCGATATTAGGTATAAAATAAATCGTAAATGGACA
AATAGTGGCTTCCATCGTTTCTATGGTTACTTCTTAAACGGCGAGGTCCTCTCTATACAC
CGGAGCCCCTTTCCTCATTTAATCAATGTTATTGGTAACTTGTACAGTTCACGCTCTTTG
GCTCTACCGATGAATTATCGAGTAATAGGTCTTTTTTCAATGGGATCTATCCATACAGTG
ACGGCATTTAATTATGAAGGTTGAATAGGTAGCTGACCCTGTTAGTCCGTTCTTGCAAGA
ATAGGAGCATAATCTTTCTGCTTCTTAAATATCATTCCCCCGCTTAATGGATAACCATTT
GCTACCAATGGGAATTGCTTTTCATCTCAAATCGAGGTGATTGGATTTGCACCAATGGAA
ACCATAAATTCCATACAAATAGAGGTATACGAGAGATCTTTATTTTTCGATAGTGAATGG
AGTTCTTCCATTCTATTCATTGGTACGAGTCATTGATACTGTAAAAGTCGTCTCATTTGT
TCTAGCTCATGATCTGAACGAGTCGCACATACACCCTAGTACATGTTCCTCGACGCTGAG
GACATCCTCGAAGAGCGGGGGATTTCGTGACATTTCTGATTGGCTGTCTTGTGTTTCTAA
TAAGTTGTTTAATAGTTGGCATGCTGAATCATATACAGAATGGGCTGGTTTAGATCGATC
CTAACCGGATGATTATGAATTACTTCTTTACCCAGGTAAGAAGATAAAAGATCAAATAAG
GGTTCATTCAAATTATGATTCGAAATGGAATCAAAGATTTATGCGAAATCCCCGGTATTT
TCGATCGCTACAAGATCAACAATGCCATAATCTTGGGCTTCTGTTGCTGACATAAAAACA
TCCCTTTCCATGTCTTCGGATACAACCCATAAAGGATTGCCCGTTCTTTGTACATAAACC
CTTGTGAGGGTTTCGCGGAGTTTCAGTAGTTCTTCCGCTTCCAGGATAAATTCTCCTGTG
GGTGCCTCATAAAAAGAACTAGCAGGTTGATGGATCATAACCCTGATGATATAACATAAT
GAACGATTCCTCTATCTCGCATGATTGGGCGAAGGGAAGGGATAAAGAATAACAAGCAGG
GAGAAAAAGATAGAATTGAACAACCGTACAGGCATCTTTTGTGCATACGGCTCTGTAATG
GAATTTTTTTTCTCTTTTTTCATCGAAGAAAGAGACAAGTCGAATCTATCAGACCCAGA
TCGTTGAATGATCCATTTACCATCCTTCCTTTCGGAATAATCAAAAAATACTATGATGGT
TCCGTTGCTTTATATATTTATCTCGTCTGTGATTCAGCAATCCCAAAGTTTCTTTCTGAT
CCGATCAAATAAAAATAAGTAAAAAATGATCTTTTTTTTTTTTTTATTTTCACACTCTT
TCATAACATAAATATTGGAAAGAGACTTCTGATGTGGAAGCAAAAAGGTTTGTGACGCTG
AAATGGACCCCGATACATAAGATCAAGTCGGAAATAACCTTTCTTTCATACTACTATCTC
GATACATAATCTCATATTATGAAAAAATAATAATAGTTTGCTCATATCGAACTTGAAATG
CCATGCTATTATTACTTAATATTATTATTATTTTATTCATATTCCATATGACGAAGGCAT
AGTCTTTTTTTCTCTCAAATAAAAAAACTCATTGGCGCCAAGCGTGAGGGAATGCTAGAC
GTTTGGTAATTTCTCCTCCAACCAGGATGAAAGATCCCATTGAAGCGGCTAATCCCATGC
ATATTGTATGCACATCTGGTGGCACAAATTGCATAGTATCATAAATAGCTATTCCTGGGA
TTACCCATCCGCCGGGAGAATTTATAAACAAATACAGATCCCTGGTATCATCCTCTATAC
TGAGATATACCATGAGACCAACAAGTTGATTCGAGATCTCGCTATCAACTTCTTGGCCTA
AAAAAGTAATCTTTCTCGATGAAGTCGGTTGATTAGGACAAAATTCTATTCCTTAGGAA
CCGTACACGCACCTTTGGGTGCATACGGTTCAAAAAATCAAAATTGAGAAAAAAAAAA
AGAAATTGTCGATTCCAGCCCTATTTCTTTTTTCGTAGCGGGCTTTTTCTTCCATTTTTT
AAGAAACATGAGTTTTGACTTGCTTCCCTATAAAATCAAAAAAGAATTCACTGAACTTAT
CGAGCTAACCCCTCATTGATGTATTGTTTCATCGAGATCTAAATCACGATGTAATTTTCT

TGTTCCCGAATGGGCCTCTTCCACTCTTTTAGGTTTATGCTCTACTCCGGGTAAAGATCT
GCCCGAATTCGATTTGCACATATAGGACAAATGATCCCAGTACCACTTCTTTTTGCTATG
ACTTCTTTTTTTTTTTTTCAATTTGTTTCATTTTCATGCCTTCCACAAAATATTCGATGT
ATTCATCATATTATTCCATTAATTGGCAATTTGGGATCACTCATATGGTATAAAGGAATC
ATTTCTGATAGGGTGGTAATCATACATGGATTACCTTGGTATTTTCTGAACGGAGCCTGT
ATACTTCATTTTATTGGTCCAAGCCAACCATAAATTCTTTTAATTGAGAATATTGATCCT
CCAACCAAATAAATTGATCTAATTGCACTTCACGCTTCGAATTATTGATGGTTCAATCAA
TCTTTCTTGGGCGAAACAGAGGATATCTCGATCGGGGGAGAGAACGGGGAAATCCCATAT
GACCCAATATGTCTGACAAGTCACACTATACGTCAACCCAAACTGCATCTTCCTCTCCAG
GACTCCGAAAAGGTACTTTTGGAACACCAATGGGCATTAAATGAAAGAAAAATGAAGTAT
TCTATTTCACTTTGATGTGGAAACGTAACAAACAATGGTTTATTGTCTTCATAATATTGT
CGTTTATCGTATTTTATCGATAGATTGGAAGATTCATAGAGGAAGACGGAATAAAGGAAA
ATTCTTACGAACGGATCGTTCGAATGAGAAACAAGTATCTATACATTCGCTCACAAAAAA
TAGGATTAATCCCCCCATTGCGTATTGGTACTTATTGGGTATAGAATAGATCTGCTTCTC
TTTGTTCCTACGAACAGAATTGTTCAATTATTACTAACGGAACAGAATAAATATTAACCC
TTGTTTCGAGATAATCCAATGAAAAGGTGAGGTCCATAGCATAGTTATTTCCAATGTGAT
AAAGTTACATAGTATCTATTTTATCTTTGAGAAAGGGGTATTTCCATGGGTTTGCCTTGG
TATCGTGTTCATACCGTCGTATTGAATGATCCCGGTCGGCTGCTTTCTGTCCATATAATG
CATACAGCTCTAGTTTCCGGTTGGGCCGGTTCGATGGCTCTATACGAATTAGCAGTTTTT
GATCCTTCTGACCCCGTTCTTGATCCAATGTGGAGACAGGGTATGTTCGTTATACCCTTC
ATGACTCGTTTAGGAATAAACAATTCATGGGGCGGTTGGAGTATTACAGGAGGAACTATA
ACGAATCCGGGTATTTGGAGTTACGAAGGTGTGGCCGGGGCACATATTGTGTTTTCTGGC
TTGTGCTTCTTAGCAGCTATCTGGCATTGGGTGTATTGGGACCTAGAAATATTCTGTGAT
GAACGTACGGGAAAACCCTCCTTGGATTTGCCCAAGATTTTTGGAATTCATTTATTTCTC
TCAGGGGTGGCTTGCTTTGGGTTTGGCGCATTTCATGTAACAGGCTTGTATGGTCCTGGA
ATATGGGTATCCGATCCTTATGGCCTAACCGGAAAAGTACAATCTGTAAATCCAGCGTGG
GGTGCGGAAGGTTTTGATCCCTTTGTTCCGGGAGGAATAGCCTCTCATCATATTGCAGCA
GGTACATTGGGTATATTAGCAGGTCTATTCCATCTTAGTGTCCGCCCACCCCAACGTCTA
TACAAAGGATTACGTATGGGCAATATTGAAACTGTCCTTTCCAGTAGTATCGCTGCTGTC
TTTTTTGCAGCTTTCGTTGTTGCTGGAACTATGTGGTATGGTTCAGCAACTACCCCGATC
GAATTATTTGGTCCCACTCGTTATCAGTGGGATCAGGGATACTTCCAGCAAGAAATATAT
CGAAGAGTTGGCGCCAGTCTAGCCGAAAATCTGAGTTTATCGGAAGCTTGGTCTAAAATT
CCCGAAAAATTAGCTTTTTATGATTACATCGGTAATAATCCGGCGAAAGGTGGATTATTC
CGGGCAGGCTCAATGGACAACGGGGATGGGATAGCTGTTGGATGGTTAGGACACCCTATC
TTTAGAGATAAAGAAGGGCATGAACTTTTTGTACGCCGTATGCCTACTTTTTTTGAAACA
TTTCCAGTAGTTTTGGTGGACGGAGACGGAATTGTGAGAGCCGATGTTCCTTTTAGAAGG
GCAGAATCGAAGTATAGTGTCGAACAAGTGGGTGTAACTGTTGAGTTCTATGGTGGCGAA
CTCAATGGAGTCAGCTATAGCGATCCTGCTACTGTGAAAAAATATGCTAGACGTGCCCAA
TTGGGTGAAATTTTTGAATTAGATCGTGCTACTTTGAAATCCGATGGTGTTTTTCGGAGC
AGTCCAAGGGGTTGGTTCACTTTTGGACATGCTACGTTTGCTTTGCTCTTCTTTTTCGGA
CACATTTGGCATGGCGCTCGAACCTTGTTCAGAGATGTTTTTGCTGGGATTGACCCAGAT
TTGGATGCTCAAGTGGAATTTGGAACATTCCAAAAACTTGGAGATCCAACTACAAGGAGA
CAGGTAGTCTGATACAACATTGCTCCGGTATCTTTCGCCTCTATATTTGATTTTTTTGAT
TTGACATAAGGTACCGTAGAAATATTGATTTGAATCATCGCCTTTCTTTGCTCTTGTCCT
TTCTTTATCTGGGAAATAATCCTAAATGAACAGGTGTGGAAGCTATAATTGTAAACAACG
ATCGAATCTATGGAAGCATTGGTTTATACATTCCTCTTAGTCTCGACTTTAGGGATAATC
TTTTTCGCTATATTTTTTCGAGACCCGCCTAAGGTCCCGACTAAAAAGACGAAATGATTT
TTCATTATCTTAATTGAAGTAATGAGTCCCCCATATGGGGGACTCATTACTTCAATTAGT
CTCCGTGTTCCTCGAATGGATCTCTTAGTTGTTGAGAGGGTTGCCCAAAAGCGGTATATA
AGGCGTACCCTGTAAAGCTTACAAGTGAACCAGATATGGAGATGGCGACTAAGGTTGCTG
TTTCCATTATTAGAGAATTTCAAGACCACGATGGATCTATGCTACGATAAGATCGTTTAT
TTACAACGGAATAGTATACAAAGTCAACAGATCTCAACCAATGCAATAGTATTTATGGCT
ACACAAACCGTTGAGGGTAGTGCTAGATCTGGGCCAAGACGAACTATTACAGGGGATTTA
TTGAAACCATTGAATTCAGAATATGGTAAAGTGGCTCCTGGATGGGGAACCACCCCATTT
ATGGGTGTCGCAATGGCTCTATTTGCGATATTCCTATCTATTATTTTAGAGATTTATAAT
TCTTCCGTTTTACTGGATGGAATTTCAATGAATTAGGTCCATAAGAACCAGAAGCCCTAG
CTTTTCAATCAAAAATGAATCACTTAGGACTCAGATTTATAGTCCATTCTGGTAGTTTGA
CCGTGGAATTCCGTTGTTTCGGTATTTCCGGAATATGAGTGTGCGACTTGTTATAATTGA
TCCTATTGATAGTACAGAGAATGGGTCTGTCATCTCGACAGAGATGGTTCTGCCTCGTCG
GATATTCATCCTAGTATCTGGAGCACGGAATATATGGAATAGATCAAGAAATATTTGAAC
TATGATTCATACCTACTATTCAGACCTCGTGACTGGACTTCAAAAAATTTTCAAACAAAG
AGGTATTTGATAAATTGAACGATTTTTCTTCCTTTAGAATCATGCTTATTTTGACCGAAG
GACAAATCTTTCTCTGGATTTTTAGTCATTACATCTATGAATAAGTGATGATCAAATAGT
TCTTACTCATAGAACCCTTGGTCTTAGTTTTTGGGTTTTATTGAATCATCGTGGTTCTAG
TATGAATCTGAGGTTTCAATCGATTCATAGGGTCTCAACAAGAGAATTCCTATCAAAAA
AAAATAGTAAACAATAGTCAATCTGCATTACGCACAAACAAAAACAACAAATCAAATAAC

AAATAAATAGGGGAATAGAAGATTCAAGAGGCCTGTAACGATCAACATAAAGACAGATGA
GCTAACTTGATATTTTGGCATTCTCATCACAACAAAGAAGAGAGTTCGGATTTTGGTTCC
TTCGTATCTTCAGAGACGATTGAATCAAGTGGATAAATAAGAAATTTCAAATTTTCTATT
ACATATCCATTGTAATCAGTATTTGGGTGTTTCTGCTTGAGCCGTACGAGATGAAATTCT
CATATACGGTTCTCAGAGGGGGAGTCCCCTTGGTTTACCTATCTCAATAAAGTATATGAT
TGGTTCGAGGAGCGTCTCGAGATTCAGGCGATTGCAGATGATATAACTAGTAAATATGTT
CCTCCTCATGTCAATATATTTTATTGTCTAGGAGGGATCACACTTACTTGTTTTTTAGTA
CAAGTAGCTACGGGTTTTGCTATGACTTTTTACTATCGTCCGACCGTTACGGAGGCTTTT
GCCTCTGTTCAATACATAATGACTGAAGCCAACTTTGGTTGGTTAATCCGATCAGTTCAT
CGATGGTCAGCAAGTATGATGGTCCTAATGATGATCCTACACGTATTTCGTGTGTATCTC
ACGGGCGGATTTAAAAAACCTCGCGAATTGACTTGGGTTACGGGTGTGGTTCTGGCTGTA
TTGACTGCATCGTTTGGTGTAACTGGTTATTCCTTACCCCGGGACCAAATCGGTTATTGG
GCAGTAAAAATCGTGACAGGCGTACCTGAAGCTATTCCCGTAATAGGATCACCTTTAGTA
GAGTTATTGCGTGGAAGTGCTAGTGTGGGTCAATCTACTTTGACCCGTTTTTATAGTTTA
CACACTTTTGTATTACCTCTTCTTACTGCCGTATTTATGTTAATGCATTTTCCAATGATA
CGTAAGCAAGGTATTTCAGGTCCTTTATAGAGAAGACAGATCATAGATATTTGTAATCGA
TCATATATAATTTCGGGGAGGAACAATAGTGTTTTATTGCTACAAATATGGATTATTGAA
AAGAATAAGACATCTTTTTGGATATTTCTCTTCAACTAACTACGAAGTATTGTATTCTTT
ATTTGATACGAATAGTTGAAGTACATTCTCCGAAGAGAAGATGGATTATGGGAGTGTGTG
ACTTGAACTATTGATTGGGCCGTGCAGATATATGATTTTATCCGCCACATTGGAATTCAC
AACCAAATGTGTCTCTGTTCCAACCACCGCGTAGGTCCCCCTACAGAGGATAGGCTGGTT
CGCTTTAGGAGAATCTTTTCTATGATCAGACCAAATCATGTTATGTTGTGCATGAACGGG
CTCCGTAAGATCCAATAGAATAAAATAAAATGAAGTAATGTGGCATGATCCAGATTATGT
TTTATCTATTTACTTAAAGTATGGAAATGCATTCATTTCCTCTGCATCGATCCCAATCTA
TGATACTATCGGAGTGAAACAAGGGATCTAAGGAAGAACATAGGCTAGACTTTATTAGTA
ACAAGGAAATCCTTTGTATTAAGAAGACTCGAGATATTGTGGGGATAAACACTAATCACA
AAGCATGAGACCATCCAAAAAGCATTTGATCATGATCAAATTTGGAAGCCTACTTGGGTA
TTGAGCATTTACTTGTAAGAACTGAATTCCTTGCAATGGGTAGTTGCAACCCCGTAAAAT
TGAATCCGGTAAATCTTTTCTTACATAGAGTCATATATGTGTGGATGATATATCTATTTT
ATATGGACCCGTTTTATTCTTTTGATTCTTGCTCGAGCCGGATGATAAAAAATTATCATG
TCCGGTTCCTTCGGGGGATGGATCTATAAGAAAGAATTCACCTATCCCAATAACAAAGAA
ACCTGACTTGAATGATCCTGTATTAAGAGCTAAATTGGCTAAAGGGATGGGGCATAATTA
TTACGGAGAACCCGCATGGCCCAATGATCTTTTATATATTTTTCCAGTAGTCATTCTAGG
CACTATTGCGTGTAACGTAGGTCTAGCGGTTCTAGAACCGTCAATGATTGGTGAACCAGC
GGATCCATTTGCAACTCCTTTGGAAATATTACCCGAATGGTACTTCTTTCCCGTATTTCA
AATCCTCCGTACAGTACCCAATAAGTTATTGGGTGTTCTCTTAATGGTTTTAGTACCAAC
GGGATTATTGACAGTACCGTTTTTGGAGAATGTTAATAAATTCCAAAATCCATTTCGTCG
TCCAGTAGCTACAACAGTTTTTTTGATCGGTACCGCGGCAGCCCTTTGGTTAGGTATTGG
GGCAACATTACCTATTGATAAATCTCTAACTTTAGGCCTTTTTTAAGTTGATTTAACCGT
GAAATACTACGCGTATGTATCTAGGGAATAGTCACTTCTAAAGTGAATTCTCCCTAGATA
CATCTCTTAAATTTCATTATCAATCCATTCTGGATATAGAGATGATACTAAGGATTCAAA
AGCCATTTTCTTCTTTTCTTTCTTTCAAAAAAGATGAAATAATACCAATGGATTTAAAAC
TTATTCTTAGGTAAATAAATTGCAAAATGCTTCTGTAGAATGTCCAATATCTGTTTTACA
TCTTCTATGCGAAGATGTTCAATTCTCATAAGATCTTCTTGACTGTTATTCAAAAGGTCC
AATAATGTATGTATATTGGACCTTTTGAGACAATTATAGGTCCTGGAGGGCAATTCTGAT
TGGTCAATAAAAATACATTTCAGTGCAATTCCTTTTTTGTTTTTCCTTATATTAGCCAAT
CTATCATGAAAAGTAAAAAACGATACAGTAAACCTGTTTTGATTGTCTTCTAAATAAAAA
TGAATGTCCTCTTCCTCCGCATGTAGAAAAGGAATAAATAAGTCAATCAAAGTACGGGAA
GCTTCGCGAAGTGCTTCTTTAGGAGTTAAACTTCCATTCGTCCATATTTCAAGAAAAAGT
ATCTCTTGTTTCTCATTCTCACTCCCATAAGAATGAATACTATGATTCGCATTTCGAACA
GGCATGGATACAGCATCTATAGGATAACTTCCATCTTGATAGTTATTGGGGGATTTCATA
CGATATCCGCGATCCCTCTCGATTTTGAATTCAATACACAAATGAATTGGCTCCGTCAGA
TTAGCTATATGCTGTGTAGTATCGACTAGTTCCACAGAAGGCGGTGAGATGATATCTTGA
GCAGTTACGTATTTAGGACCCCTGACGCAAATGGATGCGTCACGAGTTCCATACAGATTA
CTTCTCAATACAATTTCTTTCAAATTCATTAAAATTTCATGTACTGATTCTTCAATACCA
ACTATCGTAGAATATTCATGTGATACCTTCTCAGATTTTGCACGTGTGATACATGTTCCC
TCTATTTCTCCGAGTAAAGCCTTTCGCATCGCGATACCTATCGTATCTGCTTGACCTTTC
ATAAGCGGGGACAGAACGAAACGGCCATAATAAAGACGCTTACTGTCTGTTCTTGATTCA
ACACACTTCCACTGTAGTGTTCGAGTGGATACTGCTACTTCTTCTCGAACCATACTAATA
TTATTGTTTGATCAGATCATTGAATCATTTATTTCTATTGCAATCCATTCAATTTTTATT
TCTACACACGTCTTTTTTTAGGAGGCCTACATCCATTATGTGGCATAGGGGTTACGTCAC
GTACGAAACTTAATAGTATACCACTTCTACGAATGGCTCGTAATGCTGCATCTCTTCCGA
GACCAGGGCCTTTTATCATGACTTCTGCTCGTTGCAGACCCTGATCCACTGCCTTACGAA
TAGCATTTCCTGCTGCGGTTTGAGCAGCAAATGGTGTCCCTCTTCTTGTGCCTCTGAATC
CACAAGTACCAGCGGAGGACCAAGAAACCACCCGACCTATTACATCTGTAACAGTCACAA
TGGTATTGTTGAAACTCGCTTGAACATGAATAACTCCTTTTTGTATTCTACGTCCATTCT

TACGTGAACCAATTCTTGGTATAGCTTTTGTCATATTTTATCATCTCATAAATATGAGTC
AGAGATATACGGATATATCCATTTCATGTCAAAACAGATCCTTTATTTGTACATCGGACC
GTTTAGAAAGTCCCTTGTTAGAAAGATTACCCCTGTCTCTGTTTATGTTTCGGATTGGAA
CAAATTACTATAATTCGTCCCCGCCTACGGATCAGTCGACATTTTTCACAAATTTTACGA
ATGGAAGCCCTTATTTTCATATTTGTTATTCCTTAATTCCAAATATACTCCTTGGAAGAA
AATAAGTCTCTTGAAATTTTGAACCTCGAATTGTATTCCCATGAAAGGAATGTTTAAATT
CAAATAAAAAGCCGCCTAATCATTCGACTCTTTGTTGCGAAGTCTATAAATTATACGTCC
CCTAGTTGAATCATAACGACTTACTTCGATTTTGACTCTATCTCCTGGTAGTATCCGGAT
AAAACTCCGTCGGATCCTCCCCGAAACATAACCTAGAATCAGATCTTCATTGTCTAAACG
AACTCGGAACATACCATTGGGAAGTGATTCAGTAATTAAACCTTCGTGAATCAATTTTTG
TTCTTTCATTCCAGGTAACCCCCTTGAAGTATCAACTAATGGAGGAGGAGTAATAGTAGA
CAATTCGTCTTTCCTCTCTTTTTCCCAAATAGCAAGTTACGGATCAAATTCGGATACCAG
AAGGATCACCAGATATAATACAAAATTTCTCCCCCAATTCTTTCTAGTCGAGCTTCTCGA
TCTGTCATTATACCTCGAGAAGTAGAAAGAATTACGACCCCCATTCCACCTAAAATCCTA
GGAATTCGTTGATGGTTGGAATAGATTCGTAGACCGGGACGACTGATACGCTTTAAAATA
TTTCTATATGTTCCTTTCCTATTCCTTCTATGTCGCAGGGTTGAAACCAAGAAATATTTG
TTGTTTTCCCTATGTTTCCTAACATTTTCAATAAACCCTTCTTGTAGAAGTATTTTAACA
ATGTTTTCGGCGATATTAGTAGATGCTACTCGAACCGTTCCTTTTTTATCCATGTTAGCA
TTTCTTATAGAAGTTATTATATCGGCAATAGTGTCCCTACCCATGACGAACTAAATTTAT
GGGTGCCTTCCAGTTTTGATATAATCAACATGTTCCTTTTTTTTTTTCATTTTTTCTTAT
TTATTTATGAATTATTAAAGGTATATGCGTGAGACACAATCTACTAACGTGATCTATTTC
AGAGACCTGACTATACTCTATCACGGTCTCATCTACTAGTATTTATAAGACTTCAGGAGC
TAATGAGACTATTTTAGTGAAATTCAACTGTCTCAATTCCCGCGCGATCGCTCCAAAAAC
TCGAGTTCCTTTTGGATTTCCTTCTTGATCAATGACAACTGCTGCATTGTCATCATATCG
TATTATCATACCGTTGTCGCGTTTGAGTTCTTTACATGTACGTACAATTACAGCTCTGAT
CACTTCTGATCTTTCGAGAGGCATATTGGGCACTGCTTCTTTGATTACAGCAACAATAAC
GTCACCAATATGAGCATATCGTTGATTACTAGCTCCTATGATTCGAATACACATCAATTC
TCGAGCCCCGCTGTTGTCCGCTACATTCAAATGAGTCTGAGGTTGAATCATATCATTTTT
TTTTTTTTTGAATCTGCTCTTTCAATGCAAAAGGCAAAGGAAAAAGAGAGAAATATTGT
CTGCCCAGAAATCCAAAAATCTGCGATTGTATTTTTCATCACAAATACCCTTCACATACC
TATCACGCGATAATGAATTGAGTTCGTATAGGCATTTTGCACGCAGCTATTGAAATAGCT
GCTCTGGCTACAGTTTCTGATACTCCGCCCATTTCATAAAGTATTCGACCGGGTTTAACG
ACAGATACCCAATATTCGGGAGATCCTTTCCCCGAACCCATACGTGTTTCGGTAGGTCTT
ACTGTAACGGGTTTGTCGGGAAATATACGTACCCATATTTTTCCACCACGGCGTGCATAT
CGTGTCATTGCTCGTCGTCCTGCTTCTATTTGTCTAGATGTGATCCAAGCGGGTTCAAGT
GCCTGAAGAGCGTATCTGCCGAAACAAATATGATTGCCTCGATAAGATATTCCCTTCATT
CTTCCTCTATGTTGTTTACGGAATCTGGTTCTTTTGGGGTTATAGTTGATGGTTGTTTCT
CAATCCCATCTCTACTGCAGAACCGGACATGAGAGTTTCTTCTCATCCAGCTCCTCGCGA
ATGAAACGATTCAATAAGATTACGTATATGTATTTATTGAATGAATAATACACTGAATCA
TGGAATTTCTTGATATTTAATCTGTCACACGGGAAGCCGTATAGTATATAGTATATACGG
CTAGACGGATATTTCTATTTTATTTATATGGGATAATGCCTTTCTTTTGAAAATGAATCC
TTGACCTTTACCGAATCTGTCAAAATACTACAATCCAATAATGGTTTCGCGGGCGAATAT
TGACTCTTTCCATATTTGCTTCATTCGTAGGGTGAACCCATGACCTATCAGAAGAAATTA
ATTGGTTCCTGGTTGATTCCGCCATCCCACCCAATGAATCATTAGGATTCGTTTTCAATA
GAATCTTCCGCAGTCACAGGTTTCGTCGTTCCCATAGCTTTTCCATTAATGGCTAGGCCT
GAACTATGCAATGGAGCTCCTAATTAAATTCGTTCCCGAGCCAATCTCCTCAGTCTCTAT
TGACTCGGGGCTCTTTATTATTTGTATTTTTCTTATGAACCGTATTCATCTAATTATGGA
CGAATCAGTATTGATGCTTTATCACACTGCCTTTTATGATATGATGTGATTGATAGACCA
TACATATTGGAATCATATATCATGGAGATTCTCCTTCTCTCTTTCTCTCGCCCTTCCAGT
TACCCACATCCCTCTATTTTTCTTTCCAACCTATAAATGGATTTTTCCTTTATGGAAAA
AAAAGATTTCAGTTGCTACAACTATATGATCGATACATCATATGGCGACTGCTTCCTTGG
ATCTCGATAATACAAAGCAATGAGTTGGTTACTAGTTCTTATAGTTATTAGTTAGGGGCT
GGTCTGTTTTTTGAATCCCAACTTTAAATAAAAAACCAACGAGTCACACACTAAGCATAG
CAGTTCCACCAAAAGGTCAATCGAATTTTTATTCAACCTTATAGAATTAGAATTGCTCAT
TTTTCATTTTTTTTTTTTATTGAAGTGAAAAGGAATAGTTTGTAGTTTTTGTTCTATCAC
TGAATAGAATGGCAAGCAAAGGAAGGGTCCATTATTGCTCGTCTACAAATATCCAAATTT
TGATGCCCAATGCCCCATAGGCAGTTCGAACTGTATAGGAACAATGATCAATTTTAGCTC
GAATGGTTTGGAGGGGAACCCTACCTTCTCTGATCCATTCGACACGTGCAATTTCTTTTC
CGTCGATACGCCCTGCAATTTCCACTTGAATTCCTTTTGTGTCTGCTTGTTCAGTTAATT
CAATAGCTTTTTTCATTGCCTTTCGAAACGAAACCCTATTCTTTAATTGTAGAGCTATAT
ATTCTGCAAGAATATTAGGTTGTCCATAAGGTTTTGCAACTCTTGTAATAGCAATGTTAA
GTCTCCGATTCACAGAATGAAGCCCCTTTTGTACATTGATCTGCAATTCTTCGATTCCTC
GTGTTTGGCCTTCTATTAACAAATTTGGGAATCCAATATAGATTATGACCTGGATCAAGT
CCATTTTTTTTTGAATCTCTATATGTGCAATTCCAATTCCTTCGAAACTTGAAGATACTC
TTATATTTTTTTGTACATATATCTTGATCCAATCCCGTATTTTTTCATCTTCCTGGAGAC
CTATGTAATAACTTTTTGGTTGTGCGAACCAAAGGGAACGATGACTTTGGTTTTCGCCAA

GGCGGAAACCAAGTGGATTTATTTTTTGACCCATCTTTTTTTTCTCTCTCTCTCTCCTTC
TCTATATATCTTTCTATTATAGGATCTCTCCATACATTTTTTGTTTCTAAAAATATATCC
TGATCTGTTTTCTTTTTAGAGCTATCCTTCAATACAATAATTATATGACAGGCGGGTCTT
TCTATCGGATAACTACGTCCTCTAGCCCTGGGTTTTAACTTTTTCACGATAGTACCCCCA
TTGACTTCGGCTTTACTAATGACCGAATCAGCTTCGTTGAAACTTTTATTGTGACTAGCA
TTTGCTGCTGCAGAATAAACCAGTTTAAAAATGGGATAAAATGCTCGATAAGGCATGAGT
TCCAGTAACATAAGTGTTTTCTCATAGGAATGCCCACGAATCTGATCAATGACTCTTCGT
GCTTTGTGAGCAGACATACATATACGTTGAGCTAAAGCTTGTACTTGTGTACTCGAGCTC
CTCTTCATCTTCTGCTTTTTCAAGGTCTTCTTCCACTTTCTCCACTTTGACATAAGATAA
GGTTCGCCTCCCGCCAATGAACGATGAGCACCTATTTCACTTTATTTTATTAACGGAGAG
ATCTAGTATCGTTTCTCGCGTGTCCCTGGAAAGTAAGAGTAGGTGCAAATTCTCCTAATT
TTTGACCCACCATACGATCCGTTATATAAATAGGTAAATGCGCCTTTCCATTATGAATGG
CAATTGTATTGCCGATCATTGTGGGTATAATGGTAGATGCCCGGGACCAAGTTACTATTA
TCTCTTTTTCCTCTCTCATGTTAAGCTTCTTTATTTTTTCCAATAAATGATTAGCTACAA
AAGGATTTTTTTTTAGTGAACGTGTCACGGCCTATTATTCCCCCCCCTTTTTTTTTTT
GAAAAGACGAGTAAAAAACAATATTTATTTGATTTTGAATATTCCTATTTACGGCGACGA
ATAATAAAACTATTACTATATTTATTCCTTTTCCTACTTTTTCTTCCAAGCGCAGGATAA
CCCCAAGGGGTTGTGGGTTTTTTTCTACCAATCGGGGCCCTCCCTTCACCACCCCCGTGG
GGATGGTCTACAGGGTTCATAACTACCCCTCTTACTACAGGACGCCTACCTAGCCAACAT
TTAGATCCGGCTCTACCCAAACTTTTCTGGTTCGCCCCAACATTACCCACTTGTCCGACT
GTTGCTGAGCAGTTTTTGGATATCAAACGGACCTCCCCAGATGGAAATCTTAATGTGACC
GATTTACCCTCTTTTGCAATCAGTTTCGCTACAGCACCTGCTGCTCTAGTTAATTGTCCA
CCCTTTCCAAGTGTGATTTCTATGTTATGTACGGCCGTGCCTAAGGGCATATGGGTTGAA
GTAGATTTTTCTTTTTGATCAATAAAAACCCCTTCCCAAACCGTACAAGCTTCTTCCAAA
GCATACGGCTTTCCGGATGTATATATATATTCTATGATGATATCTAGACAGATGGATTTT
ATATGAATCGTGTGATGAAGTACCACATGAGTGGATATATAGGAATACAAATCTGCCAAA
TCACTCATGTTATGATCTTATACATCCTAGGTCTCTCCGTTTCGTCATCTGGCTTATGTT
CTTCATGTAGCATTCAGACCGAATGACTCTATGAAATTACGTCGCTACTTCCACATATTA
CGGGTAACGTAGGAGACATCTCTATTTTTCCCCGGGGGAATTTTTAGAATTACCACCACT
TAGCTTTCAATTCACCTCTGACCATCAAATGAAATGTGAATAACCCATCCTCTTCTCTTT
GAAACAAGGGTCGCTTCCGCTTCTGTCCGTGCTTCAAACAATTTTGTCTTCTCCATATTA
CCATATCTGGAGTGTCAATAGTTTTCTATGAGGAACTACTGAACTCAATCACTTGCTGCC
GTTACTCTTCAGTTTTCTGTTGAGGTCTATCCCGTAGAGGTACTCAAATTGGATCAGTGA
TCAATTTCTAGGTTTCGTCGTAAACCTAATTGGTTACTTCCAATTACGTAAATCCATAGT
TCAAACCGCACTCAAAGGTAGGGCATTTCCCATTGATATAGGAACTTCTGTACCGGAAAC
AATGGTATCTCCAATTATAGCCCCTCTGGGATGTAAAATATATCTTTTCTCACCATCTCC
ATAGTGTATGAGACAAATGTATGCATTTCGATTAGGGTCATATTCTATGGTTACGATCCT
AGCAGATATGTCTTTTTCATTCCGTCGAAAATCGATTTTACGGTATAGACGCTTATGGCC
TCCTCCTCTATGCCCTGCGGTAATGATTCCCCTGGAATTACGACCTTTACCGCAGCGATG
CTGTCCATAGATCAAATTATTTCGCGGATTGGATTTCGCTTGACTGTCTATGGATCCTTT
GCGTATGCTCGGGGTAGAAGTTTTGTATAAATGTATCGCCGTGTTATTTAGTATTTTTT
TTTCTTTTTTTTTTTTACTTAAATTCTTTTCTCTTCTCTATAAGAGGTAAAATAGAATAA
CCCGGTTGAAGCGTAATGATCATACGTCTGTAATGCATTGTATGTCCCATAATGGGTCCC
GTTCTTCTACCCTTTCCCGGGAGTTGATGACTATTTATAGCTATTACTTTGACGCCAAAG
AAGAGTTCGACCCAATGCTTTATTTCTGTCCTAGTTGATCCTGATTCGACTTTCTTCCCC
ACGAGTTCCAGTATCGATAAGAATTCTAGTTCTTACTCTTCATATGTTATGGTTGGTATG
AATATACCATACCAATTCGTTATGTATGGATGATGAGATTCCATTGATACGGAGCCAGTG
GAATTAGTCTTATTGAATGTCCCCGTTGGCCTGCATCCAGCAGGAATTGAACCTACGAAT
TCGCCAATTATGAGTTGGGTGCTTTAACCATTCAGCCATGGATGCTTCACTGGGGTCATT
GTACATCGCGAGTGACCCAAATTCAATTCACTTAGATTCTTTTGGATTCTTTAGGAGGAA
TCAATGAAATGAGAGGACATCAATTCAAATCCTGGATCTTCGAATTGAGAGAAATCAAGA
ATTCTCACGATTTCTTGGATTCATGGATCCAACCCGATTCGGTGAAATCTTTCACTTCCT
TTTTTTTCCACCAAGAGCGCTTTATGAAACTTTTTGATTCCCGAATTTGGAGTGTCCTAA
TTTCACGTGATTCACAGGGTTCAATTCGTCGACATTGCATGATCAAAGGTGTAGTACTGC
TTGTACTTGTAGTAGCGGTCCTTATATACAATCGAAATAGGGTCGAAAGAAAAAATATCT
ATTTGATGGGGCTTCTTCCTAAACCTCTGCGTTCCATTGGACCCCCCAATTATACATTGA
AAGAATCCTTTTGGTCTTCCAATCTCAATAGGTTGATTGTTTCGCTCCTGTATCTTCCAA
AAGGGAAAAATATCTATGAGAGTTGTTTCATGGATCCGAAAGAAAGTACTTGGGTTCTTC
CAATAACTAAAAAGTGTATCATGTCTGAATCTAACTGGGGTTCGCAGCGATGGAGGAATG
CGATCGTAAAAAAGAGGAATTCCAGCTGTAAGATATCGAATGAAATTGCAGCTGGAATTG
AGATCTCATTCAAAGAGAAAGATATCAAATATCTGGAGTTTTTTTTTGTATCCTATACGA
ATGATCCGATCCGCAAGGACCATGATTGGAAATTATTTGACCGCCTTTCTCCGAGTAAGA
AGCGAAACATAATCAACTTGAATTCGGGACAGCTATTCGAAATTTTAGTGAAACATTTGA
TTTGTTATCTCATGTCTGCTTTTCGTGAAAAAAGACCAATTGATGAGGGGGGTTTCTTCA
AACAACAAGGAGCTGAGGCGACTATTCAATCAAACGAGATTGAACATGTTTCCCATCTCC
TCTCGAGAAACAAGGGGGGTATTTTTTTGAAAAATTGCGCTCAATTTCATATGTGGCAAT

TCCGCCAAGATCTCTTCGTTATTGGGGGGAAGAATCGGCACAAATCGGATTTTTTGAGGA
ACGTCTCGAGAGAGAATTTGATTTGGTTAGACAATGCGTGGTTGGTAAACAGGAATCGGG
TTTTTAGCAAGGTACGGAATGTATCGTCAAATATTCAATATGATTCCATAAGATCCATTT
TCTTTCAAGTAACGGATTCTAGCCAATCGAAAGGATTTTCTGATCAATCCATAGATCCTT
TCAATTCCATTAGTAATGAGGGTTCGGAATATCACACATTGATCAATCAAACGGAGATTC
AGCAACTAAAAAAAAGATCAATTCTTTTAGATACTTCCTTTCTTCAAACGGAACGAACAG
AGATAAAATCAGATCGATTCTCAAAATACCTTTCCGGATATTCCTCAATGGCTCGGCTAT
TCCCGGAACGTGAGAAGCAGATGAATAATCATCTGCTTCCAGAAGAAATAGAAGAATTTC
TTGGGAATCCTACAAGATCAATTCGTTCTTTTTTCTCTGATAGATGGTCAGAACTTCATC
TGGGTTTGAATCCTACCGAGAGGTCGACTATAGATCAGAAATTGTTGAAGAAACAACAAG
GTGTTTCTTTTGTCCCTTCGAGGCGATCGGAAAATAAAGAAATAGTTGATATATTCAAGA
TAATTACGTATTTACAAAATACCTCCTCAGTTCATTCGATTGCAGCAGATCCGGGATGGG
ATATGGTTCCGAAGGATGAACCGGATATGGACAGTTCCAATAAGATTTCATTCTTGAACG
AAAATGCATTTTTTGATTTATTTCATCTATTCCATGATCGGAACAAGGGGGGATACAGGT
TGCACCACGAGTTTGAATTAGAAGAGACATTTCAAGAAATGGCAGATCTATTCACTCTAT
CAATAACCGAGCCGGGTTTAGCCTATCATAATAAGGAATTTGGCTTGTCTATTGATTCCT
ACGGAAAATTATTGAATGAGGTATTCAACTCCGGGGATGAATCGAAAAAGAAATCTTTAT
TGGTTCTACCTTCCATTTTTTATGATTTATTTTTATTGGTTCTATCTTCTATTTTTTATG
ATTTATTTTTATTGGTTCTACTTTCTATTTTTTATGATTTATTTTTATTGGTTCTACTTT
CTATTTTTTATGAAGAGAATGAATCTTTTTATCGAAAGATAAAAAAAAAATCGGTCCGGA
TCTCCTGCGGGAATGATTTGGAAGATCCAAAACCAAAAATAGCGGTATTTGCTCACAACA
ACATAATGGAGGCGATCCATCAATATAGATTGATCCGAAATCAGATTCAAATCCAATATA
GTACCTATGGGTACATAAGAAATGTATTGAATCGATTCTTTTTAATGAATCGACCCGATT
GCAACTTCGCATATGGAATTCAAAAGCATCCCATAGGAATTCAAAAGCACCCAATAGGAA
ATGATATTCTGAATCATCTAACTATAATAATAGATAAGATCAACCAACATTTATCCAATT
TGAAAAAGATTAAGAAGAAGTGGTTCGATCCTCTTATTTCTCGAACCGAGAGATCCACGA
ATCTGGATCCTAATGTATATAGATACAAATGTTCCAATGGAAGCAAGAATTTCCAGGAAC
ATTTGGAGCATTTCGTTTCTGAGCAGAAACACCGTTTTCAAGTAATGTTCGATCGATTAC
GTATTAATCAATATTCGATTGATTGGTCCGAGGTTATCGACAAACAAGATTTGTCCAAGT
CACTTCGTTTCTTTTTGTCCAAGTCACTTCTCCTTTTGTCCAAGTCGCTTCTCTTTTTAT
CTAAGTCACTTCCTTTTTTCGTTGTGAGTCTCGGGAATATCTCCATTCATAGGGCCGAAA
TCCACATCTATGAATTGAAAGGTCTGAATGATCAACCCGGCAATCAGTTGTTAGAATCAA
TAGGTGTTCAAATCGTTTATTTGAATAAATTGAAACCCTTCTTATTGTATGATCATGATA
CTTCCCAAAGATCGAAATTTTTAATCAATACAGGAACAATATTACCTTTTTTGTTCAACA
AGATACAAAAGTGCATGATTGACTCATTCCGTACTAGAAAAAATCGCAAGAAATCCTTTG
AGAACACGGATTCCTATTTCTCAATGATATCCCACGATCGAAACAATTGGTTGAATCCTC
AGAAAAGTTCATTGATATCTTCTTTTTATAGAGCAAACAGACTTCAATTCTTGAATCATC
CCCATTGCTTCTGGTTCTATTGTAACAAAGGATTCCATTTTTATGGGGAAAAGACCCGTA
TCCATAATTATGATTTTACATATGCACAATTCCCCAATATCTTGTGCATTCGCAACAAAA
AATTTTCTTTGTGTTTCGGTAAAAAAAAACATGTTTTGGGAGAGAGAGAGACTATTTCCC
CAATTGAGTCACAGGTATCTGGCATATTCATACCTAACAATGTTTCACAAAGTGGTAACA
AAACGTATAACTTGTACAAATCTTTCCATTTTTCAATTCGATCTGATCCATCCGTTCCTA
TTTACTCGATTGCAGACATTTCGGGAACACCTGTAATAGAGGAACAAATAGTCAATTTTG
AAAGAACTTATTGTCAGCTTCTTTCAGATATGAATCTATCTGATTCAGAAGGGAAAAACT
TGCATCACTATCTCCGTTTCAATTCAAACATGGGTTTGATTCACACTCCATGTTTTGAGA
AATATGTGCCGTCCGGAAAGAGGAAAGAACTGAGTCTATGTCTAAAGAAAAACGTTGAGA
AGGGGGAAGTAGGTAGAACCCTTCAACGAGATAGTGCTTTTTCAAATCTCTCAAAATGGA
ATTTGTTCCAAACCTATATGCCATGGTTCCTTACTTGGACGGGGTGTAAATATCTTTATT
TCACCTTAAAAAACAATATTTATTTGATATTGAATATTCCCTTTCAATATTCCCTAAGTG
GCAGTCAAAATTTTGTGTCCGTTTTTCATGATATTATGCATGGATCAGATATATCATGGC
CAATTCCTCAGAAAAAGTGGTGGTCGATTCTTCCACAACGGAATCTGATAAGTGAGAGTT
CGAGTAAGTGTTTACAGAATCTTCTTCTGTCCGAAGAAATGATTCATCGAAATAATGAGT
CACCCATTCCATTGATATGGACACATCTGAGATCACCAAATGCTTGGGAGTTCCTCTATT
CAATTCTTTTCCTTCTTCTTGTTGCTGGATATCTCGTTCGTACACATCTTCTCTTTGTTT
TCCGAGCCTCTAGTGAGTTACAGACAGAGTTAGAAAAGATCAAATCTTTGATGATTCCAT
CATACATGATTGAGTTGCGAAAACTTCTGGATAGGTATCCTACATCTGAACTGAATTCTT
TCTGGTTAAAGAATCTCTTTCTAGTTGCTCTGGAACAATTAGGAGATTCTCTGGAAGAAA
TACGGGATTCTGCTTCTGGCGGCAACATGCTATTGGGTGGTGGTCCCGCTTATGGGGTCA
AATCAATACGTTCTAAGAAGAAATATTTGAATATCAATCTCATCGATCTCATCAGTATCA
TACCAAATCCCATCAATCGAATCACTTTTTCGAGAAATACGAGACATCTAAGTCGTACAA
GTAAAGAGATCTATTCATTGATAAGAAAAAGAAAAAACGTGAACGGTGATTGGATTGATG
ATAAAATAGAATCCTGGGTCGCGAACAGTGATTCGATTGATGATGAAGAAAGAGAATTCT
TGGTTCAGTTCTCCACCTTAACGACGGAAAAAAGGATTGATCAAATTCTATTGAGTCTGA
CTCATAGTGATCGTTTATCAAAGAATGACTCTGGTTATCAAATGATTGAACAACCGGGAT
CCATTTACTTACGATACTTAGTTGACATTCATAAAAAGTATCTAATGAATTATGAGTTCA
ATAGATCCTGTTTAGCAGAAAGACGGATATTCCTTGCTCATTATCAGACAATCACTTATT

CACAAACCTCGTGTGGGGCTAATAGTTCTCATTTCCCATCTCATGGAAAACCCTTTTCGC
TCCGCTTAGCCCTATCCCCTTCTAGGGGTATTTTAGTGATAGGTTCTATAGGAACTGGAC
GATCCTATTTGGTCAAATACCTAGCGACAAACTCCTATGTTCCTTTCATTACGGTATTTC
CGAACAAGTTCCTGGATGACAAGCCTAAAGGTTATCTTATTGACGATATCGATATTGATG
ATAGTGACGATATCGATATTGATGATAGTGACGATATTGATGATGACCTTGATACGGAGC
TGCTAACTATGACGAATGTGCTAACTATGTATATGACGCCGAAAATAGACCGATTTGATA
CCACCCTTCAATTAGAATTAGCAAAAGCAATGTCCCCTTGCATAATATGGATTCCAAACA
TTCATGATCTGTATGTGAATGAGTCGAATTACTTATCCCTCGGTCTATTAGTGAACTATC
TCTCCAGAGATAGTGAAAGATGTTCCACTAGAAATATTCTTGTTATTGCTTCGACTCATA
TTCCCCAAAAAGTGGATCCCACTCTAATAGCTCCGAATAAATTAAATACATGCATTAAGA
TACGAAGGCTTCTTATTCCACAACAACGAAAGCACTTTTTCATTCTTTCATATACTAGGG
GATTTCACTTGGAAAAGAAAATGTTCCATACTAACAGTAACAGATTCGGGTCCATAACCA
TGGGTTCCAATGCACGAGATCTTGTAGCACTTACCAATGAGGCCCTATCAATTAGTATTA
CACAGAAGAAATCAATTATAGACACTAATACAATTAGATCAGCTCTTCATAGACAAACTT
GGGATTTGCGATCCCAGGTAAGATCGGTTCAGGATCATGGGATCCTTTTCTATCAGATAG
GAAGGGCTGTTGCACAAAATGTACTTCTAAGTAATTGCCCCATAGATCCTATATCTATCT
ATATGAAGAAGAAATCATGTAAGGAAGGGGATTCTTATTTGTACAAATGGTACTTCGAAC
TTGGAACGAGCATGAAGAAATTAACGATACTTCTTTATCTTTTGAGTTGTTCTGCCGGAT
CGGTCGCTCAAGATCTTTGGTCTCCACCCGGACCCGATGAAAAAAATTGGATCACTTCTT
ATGGATTCGTTGAGAATGATTCTGATCTAGTTCATGGCCTATTAGAAGTCGAAGGCGCTC
TGTTGGGATCCTCACGGACAGAAAAAGATTGCAGTCAGTTTGATAATGATCGAGTGACAT
TGCTTCTTCGGTCCGAACCAAGGAATCAGTTAGATATGATGCAAAACGGATCTTGTTCTA
TCGTTGATCAGAGATTTCTATATGAAAAATACGAATCGGAGTTTGAAGAAGGGGAAAGAG
AAGGAGCCCTCGACCCGCAACAGATAGAGGAGGATTTATTCAATCACATAGTTTGGGCTC
CTAGAATATGGCGCCCTTGTGGCAATCTATTTGATTGTATCGAAAGGACCAATGAATTGG
GATTTCCCTATTGGGCCAGGTCATTTCGGGGCAAGCGGATCATTTATCATAAAGAGGATG
AGCTTCAAGAGAATGATTCGGAGTTCTTGCAGAGTGGAACCATGCAGTACCAGACACGAG
ATAGATCTTCCAAAGAACAAGGCTTTTTTCGAATAAGCCAATTCATTTGGGACCCTGCAG
ATCCATTCTTTTTCCTATTCAAAGATCAGCCCTTTGTCTCTGTGTTTTCACGCCGAGAAT
TCTTTGCAGATGAAGAGATGTCAAAAGGGCTTATTACTTCCCAAACAAATCCTCCTACAT
CTATATATAAACGCTGGTTCATCAAGAATACGCAAGAAAAGCACTTCGAATTGTTGATTC
ATCGCCAGAGATGGCTTCGAACCAATAGTTCATTATCTAATGGATCTTTCCGTTCTAATA
CTCCATCCGAGAGTTATCAGTATTTATCAAATCTGTTCCTATCTAACGGAACGCTATTGG
ATCAAATGACAAAGGCATTGTTGAGAAAGAGATGGCTTTTCCCGGATGAAATGAAACATT
TGATTCATGTAACAGGCTAAAACGGACTATGTACTTTATCTGTTGGGTTACGGGCGGGCA
TTTTACCAGAGGTTTCTATTGTATCAATTTACCCTTGTGTGATTCCTGTTGAAGCATATA
CTCGGGGGGTGGGTGCAGGGCGGACGATTTCAAAACGGACTCCTCATTCATTAGATAGAG
AAGATCGCCAAGATTTCGTGATCCGCTGCCGAACCTATTCCAATTCCAACAGCCCGGACT
CGGATCGTGGGGATCGATGGAATACTTCGTATCAACAGATACTTGGTATATGTATATCAA
TATTGATTAGATCCGAGATCTGTTATTGAATTGCTCATTCAATGAGCATTTCAATATTAT
GCCTTGAAGAGGACTCGAACCTCCACGCTCTTTAGCACGAGATTTTGAGTCTCGCGTGTC
TACCATTTCACCATCAAGGCATCTTGAAAGTGAATCGTATTCCATGAATATGATATCTAT
CTAGTGTGATATATGGAATATATGACAAAGGTGGAGTTTTGGAGTATTTCTATCGATCGG
TCATGTCATATAGGCCCGAGTCGGACATCAAATTGCTTCGATTTGAATTATCCGGAGGAT
ACCTTATATATATCAAAAAGATGTACAATCAAACCTATTTCTCGATTCAATCGAAGCCCA
AAGAAGTTAATATGGTACCCAAATAACGATAGATATGTAAAAAGCAGGTCCGATTACGCC
TATTCCTAATCCTAAATGGAATGTAACGACGTAGGGATCCATATGTAAACATAGTCTCTA
TTTACATATGCTCGAATGACCCCTTCTCATAATGAGAATGTACATAACCCTATTCCGGTC
TGGTCCGGTATGGAATGAACTTATAATCTGATGATCGAGTCGATTCCATGATTATAAGTT
CATAACCCCAGCCCATTCCCATTTTGGGCGGAACAGATCTACTAATTCTTTTATTCCAGT
TAGTAAAAGGGATCTTGAACTAAGAAATAGACCCTAGAAGCTAAAAGAGGGTATCCTGAG
CAATTGCAATAATTGGATTCATTGATATTCCTGGTATAGTAGATGCTATCACACATACAA
TCATACTCAATTCGATGGAATTGTTTGATCTTAAGGGGGATCTTCTATAATTTCGCACGT
GAGGGGTTATTTCTTGGTTTCGTCCAGTCATTAATAACTTGATTATTTTTAGATAATAGT
AGATAGAAACAACGCTCGTAAGGAGTCCTATTGAAACCAAGAAATATAGGCCTGCCTGCC
ATCCACACCAGAATAGATGGAGTTTTCCGAAAAAACCTGCTAGTGGAGGAAGACCTCCTA
GGGATAAGAGACATAGGGCTGAAGAGAGAGCCAAAAAAGGATCTTTCGTGTATAATCCTG
CATAATCTCGAATGTTATCAGTTCCGGTACGTAGACCAAATGAGACAATGCGAGCAAAAG
TTCCTAGATTCATGGAGATATAGAACAGCATATAAGTTATCATGCTTGCATATCCATCAT
TTGAGTCTCCAACAATTATTCCAATAATTACATATCCGATTTGACCTATGGACGAATATG
CAAGCATACGTTTCATGCTTGTTTGAGTAATAGCAATGAGATTCCCCAATATCATGCTAA
GAATAGCTAGGATTTCCAGAAGAAGATGCCATTCGTTTGATGAGAAATAAAAAGGAATAT
CGAAAATTCGAGTGGCTGAAGCTGAAGCAGCTACTTTCGAAGTAACAGAAAGAAAAGCAA
CGACTGGAGTGGGAGAGTCAGAGTCGAAAAGAGGATTCCTCACTTCTTTCTCTCATTCAA
AACCGTGCATGAGACTTTCATCTCGCACGGCTCCTAAGTGATAAAAGAAAGAAGAACTCA
CCTTCTTTCTTTTTTGATTACCTTCCTCGCGTATGTATAAGACCGAATCCATTCGATTTC

TAAAAAGGATTACTAATCCTTAACTTTTCGAGGAATCCTTCATCAGTGGTTGTGAATGAC
TGACTTTTTCAATCTTTTCGACCCCGGTTCCGTAGGAGCACAAGTCAGAAAGATTGAGAA
ATAGAACCATCTGATTTGATTCGTTCTCAATAGCCATGAGATGATCATCTTAGGGTGATC
CTTTTGTCGACGGATGCTCCTATTACACTCGTAGTCTCTGAAGGATGAGAACCAACTATG
TAGCATCTACATCGATAATTCAAGTATTGTATACGTCATTAGTCCGATCCTTTGTAGGAA
CTACCCGTAATAACGAACTTGCAAAATGGATCTGTTTATCATAAAGAGATTCGTTGTTCC
TGACCCTGCTTCACCTTAATTGTTATTTGAACAAAAAGATCACAATAAACTTTTGGTAAA
AGTTATGTCTTGGTCCGAGTGGGGATAGCATTTCTCTTCTGCATGTCCATGGAGTTTTGA
AAAATCCAAACATCTCAGAGATAGATATAGAGGTAGGAATTTGTCGAACGAACCGCACTC
CTTCGTATACGTCAGGAGTCCATTGATGAGAAGGGGCTGGGGAAAGCTTGAACCCAATTC
CTACAGTGATGGATATAAGCGCAATGGAAATTCCTGGGGAGTTATACATTTGTGTATTGA
TAAGACCATTCACTATTTCTTGAAGCTCGATCTCTCCCCCGGATGAACCATATAGCCAAG
AGAAACCATGAACCAGAATAGAAGAGCTTGCCCCACCCATGAGTAAATATTTCGTAGTAG
CCTCATTAGACCGTACATCTCTCTTGGTATATCCAGATAATAGGTAGGAGCATAAACTGA
AAGATTCTGGAGCTACAAAGATAGTTATTAAATCGTTAGCACCACATAAAAACATTCCTC
CTAGAGTAGCTGTTAATACGAATAACAAAAACTCTGTTATAGCCATTTCTGTACATTCAA
TGTACTCTACGGATAGAGGAATACATAGAGTTGAACATAGTAAAATAAGAAATTGAAAGA
TTTCGTTGAAATTGTTCGTTTGGAAATTTCCCGAAAAGCTAATCATAGGTTCTTCTCTCC
ATCGGAACAATAGGGCCGTTATGCTCATTACTAAACTTGTTGAAGAGATGAAATATAACC
AAGGTATATCTTTTTGATCAGAGGTTGAATCGATCATCAGAAGAAGAATTAGGCCAAAAA
TGAGGATACATTCTGGGAAAATGAAACTTCCATGGAAGAGAAGCAAATGAAACGCTTTCA
TAAAAATTCTCGTAGAATCGAGAATGAAGTTTTCATTCTGTACATGCCAGATCCTGAATT
AGTAACTGCATCCAATCTCCGAAAAAGTCCCAATTGTTTCGAACTTTCGATTTTTGGAAT
GGGATATTTACGGAATCCCCATGAACAGGATCAAACCTTATTCCATGGTATTTCCATGAG
ATTCCTCCTTCTTATTCTTAAGAAAGCCCCCGAGAGGGCTTAGTTGATCCATGATTTATG
TTTCATCTTTCTTTTCCTTTTCGTTTGTTTCGAGAAAGATATCGATCCATTCCAATTCTT
TCTTTTTCTATTGATTCTTTTCCGATCGAGATGTATGGATCCACGGATCTATGTGTCTAT
ATAGATCCTGTTCATGGATTAACGAAAATGTGCAAAAGCTCTATTTGCCTCTGCCATTCT
ATGAGTCTCTTCCTTTTTGCGTATGGCATCGCCACTCCCTTTGGCAGCATCCACTAATTC
GGAACTTAATTTGAAAGCCATATTTCGACCCGGACGTTTTCGGGATGCCCCTAATAACCA
ACGAATGGCAAGTGCTTTTCCTTGTGTAGATTCTATTTCAATAGGAACTTGATGAGTCGA
TCCACCTACACGTCTTGCTTTGACTGCTATATCGGGAGTTACTCCACGTATTGCTTGACG
TAAAACAGATAGTGGATTTGTTTCTGTCTTTTGTTGAATCTTTTTCACGGCTTGATAGAT
AATTTGATAAGCCAATGATTTTTTTCCGTGTTTCAGAATACGGTTAACCAACATGTTAAC
TAATCGATTACGATAAATTGGATCGGATTTTGCAGTTTTTTCTTCTGCAGTACCTCGACG
TGACATGAGCGTGAAAGAGGTTCAAGAATCCGTTTTCTTTTTATACGGGCTAAAAACGAA
TCACTTATTTTGGCTTTTTGACCCCATATTGTAGGGTGGATCTCGAAAGATATGAAAGAT
CTCCCTCCAAGCCGTACATACGACTTTCATCGAATACGGCTTTCCACAGAATTTGATATG
TATCTATGAAATCGAGTATGGAATTCTGTTTACTCACTTTAAATTGAGTAGCCGTTTCCC
TCCTTTTCCTGCTAGGATTGGAAATCCTGTATTTTACATATCCATACGATCGAGTCCTTG
GGTTTCCGAAATAGTGTAAAAAGAAGTGCTTCGAATCATTGCTATTTGACTCGGACCTGT
TCTGAAAAAGTCGAGGTATTTCGAATTGTTTGTTGACACGGACAAAGTAAGGGAAAACCT
CTGAAATGATTTCAATATTGAACCTTGGACATATAATAGTTCCGAATCGAATCTCTTTAG
AAAGAAGATCTTTTGTCTCATGGTAGCCTGCTCCAGTCCCCTTACGAAACTTTCGTGATT
GGGTTAGCCATACACTTCACATGTTTCTAGCGATTCACATGGCATCATCCAATGATACAA
GTCTTGGATAAGAATCTACAACGCACTAGAACGCCCTTGTTGACGATCCTTTACTCCGAC
AGCATCTAAGGTTCCTCGAACAATGTGATATCTCACACCGGGTAAATCCTTAACCCTTCC
TCCTCTTACTAAGACTACAGAATGTTCTTGTGAATTATGGCCAATACCAGGTATATAAGC
AGTGATTTCAAATCCAGAGGTTAATCGTACTCTGGCAACTTTACGTAAGGCAGAGTTTGG
TTTTTTGGGGGTGATAGTGGAAAAGTTGACAGATAAGTCACCCTTACTGTCACTCTACAG
AACCGTACATGAGATTTTCACCTCATACGGCTCCTCGTTCAATTCTTTCGAAGTAATTGG
GTCCTTTTCCTCGTTCGAGAATCTCCTCCCTTCTTCCACTCCGTCCCGAAGAGTAACTAG
GACCAATTCAGTCACGTTCTCATGTTCCAATTGAACACTTTCCATTTTTGATTATGATCA
AAGGAGAAGATTATTCTTTTTACCAAAATATGCGGATCAAATCACGATCTTATAATAAGA
ACAAGAGATCTTTCTCGATCAATCCCTTTGCCCTCATTCTTCGAGAATCAGAAAGATCCT
TTTTGAGTTTGAATTTGTTCATTTGGAATCTTTATTTATTTTTGTATTTTTTATTTATTT
ATTTTCTTTGATTCTTTATTTCGATTTTCTTTCCCTCTCTTTTCTTTTTATTCCCTTCCA
TCATTCCTTAAGTCCCATAGGTTTGATCCTGTAGAATCTGACCCATTTTCTCATCGAACG
AGGGGTACGAAATAAATCCGATTGATTTTTCGATCAAAAGTACTATGTGAAATCTTCGGT
TTTTTCCTCTTCCTCTATCCCATAGGTACAGCGTTTGAATCAATAGAGAACCTTTTCTTC
TGTATGAATCGATATTATTACATTCCAATTCCTTCCCGATACCTCCCAAGGAAAATCCCG
AATTGGATCCCAAATTGACGGGTTGGTGTAAGCTTATCCATGCGGTTATGCACTCTTCGA
ATAGGAATCCATTTTCTGAAAGATCCTGGCTTTCGTGCTTTGGCGGGTCGTCCGAGATCC
TTTCGATGACCTATGTTGTGTTGAAGGGATATCTATATGATCCGATCGATTGCGTAAGGC
CCGCGGTAGCAACGGAGCCGGGAAAGTATACAGAAAAGACAGTTCTTTTCTATTATATTA
GTATTAGTTAGTGATCCCGGCTCGGTGAGTCCTTTCTTCCGTGATGAACTGTTGGCACCA

GTCCTACATTTTGTCTCTGTGGACCGAGGAGAAAGGGGGGCTCAGCGGGAAGAGGATTGT
ACCATGAGAGAAGCAAGGAGGTCAACCTGTTTCAAATATACAAATGGATTCTGGCAATGC
AATGTAGTTGGACCCTCATGTCGATCCGAATGAATCAGTCTTTCCACGGAGGTCAATCTT
TGCCTGCTAGGCAAGAGGATAGCAAGTTACAAATTCTGTCTCGGTAGGACATGTATTTCT
ATTACTATTAAATTCAGAAATGAAGTAGTTAATGTTGGGGTTACCATTATCCTTTTTGTA
GTGACGAATCTTGTATGTGTTCCTAAGAAAAGGAATTTGTCCCTTTTTCGAG
GTCTCAAAAGGGCGTGGAAACACATAAGAACTCTTGAATGGAAATTGAAAAGAGATGTAG
CTCCAGTTCCTTCGGAAATGGTAAGATCTTTGGCGCAAGAAGAAGGGGTTGATCCGTATC
ATCTTGACTTGGTTCTGCTTCCTCTCTTTTTTTAACAATACCGAGTCGGGTTCTTCTCCT
ACCAGTATCGAATAGAACATGCTGAACAAAATCTTCTTCCTGTAAAACCTGCTCGATTTA
GATCGGGAAAATCGTACGGATTTTATGAAACCATGTGCTATGGCTCGAATCCGTAGTCAA
TCCTATTTCCGATAGGAGCAGTTGACAATTGAATCCAATTTTTCCATTCTTTTCGTATCC
GTAATAGTGCGAAAAGAAGTCCCGGCTCCGAGTTGTTCAGGAAGAGTGGCGTTGAGTTTC
TCGACCCTTTGCCTTAGGATTAGTCAGTTCTATTTCTCGATGGGGGCAGGGAAGGGATAT
AACTCAGCGGTAGAGTGTCACCTTGACGTGGTGGAAGTCATCAGTTCGAGCCTGATTATC
CCTAAACCCAACGCAATGTGAGTTTTTCTATTTTGACTTGCTCCCCCGCCGTGATCGAAT
GAGAATGGATAAGAGGCTCGTGGGATTGACGTGAGAGGGTAGGGATGGCTATATTGCTGG
GAGCGAACTCCAGGCTAATATGAAGCGCATGGATACAAGCCTTGGAATGAAAGACAATTC
CGAATCAGCTTTGTCTACGAACCGGAAGATTGGTAAGTAATGCAACTATGAATCTCATGG
AGAGTTCGATCCCGGCTCAGGATGAACGCTGGCGGCATGCCTAACACATGCAAGTCGGAC
GGGAAGTGGTGTTTCCAGTGGCGGACGGGTGAGTAACGCGTAAGAACCTGCCCTTGGGAG
GGGAACAACAACTGGAAACGGCTGCTAATACCCCGTAGGCTGAGGAGCAAAAGGAGGAAT
CTGCCCGAGGAGGGGCTTGCGTCTGATTAGCTAGTTGGTGAGGCAATAGCTTACCAAGGC
GATGATCAGTAGCTGGTCCGAGAGGATGATCAGCCACACTGGGACTGAGACACGGCCCAG
ACTCCTACGGGAGGCAGCAGTGGGGAATTTTCCGCAATGGGCGAAAGCCTGACGGAGCAA
TGCCGCGTGGAGGTAGAAGGCCCACGGGTCGTGAACTTCTTTTCCCGGAGAAGAAGCAAT
GACGGTATCTGAGGAATAAGCATCGGCTAACTCTGTGCCAGCAGCCGCGGTAAGACAGAG
GATGCAAGCGTTATCTGGAATGATTGGGCGTAAGGCGTCTGTAGGTGGCTTTTCAAGTCC
GCCGTCCAATCCCAGGGCTCAACCCTGGACAGGCGGTGGAAACTACCAAGCTGGAGTACG
GTAGGGGCAGAGGGAATTTCCGGTGGAGCGGTGAAATGCGTAGAGATCGGAAAGAACACC
AACGGCGAAAGCACCCTGCTGGGCCGACACTGACACTGAGAGACGAAAGCTAGGGGAGCG
AATGGGATTAGATACCCCAGTAGTCCTAGCCGTAAACGATGGATACTAGGCGCTGTGCGT
ATCGACCCGTGCAGTGCTGTAGCTAACGCGTTAAGTATCCCGCCTGGGGAGTACGTTCGC
AAGAATGAAACTCAAAGGAATTGACGGGGGCCCGCACAAGCGGTGGAGCATGTGGTTTAA
TTCGATGCAAAGCGAAGAACCTTACCAGGGCTTGACATGCCGTGAATCCTCTTGAAAGAG
AGGGGTGCCTTCGGGAACGCGGACACAGGTGGTGCATGGCTGTCGTCAGCTCGTGCCGTA
AGGTGTTGGGTTAAGTCCCGCAACGAGCGCAACCCTCGTGTTTAGTTGCCACCATTGAGT
TTGGAACCCTGAACAGACCGCCGGTGATAAGCCGGAGGAAGGTGAGGATGACGTCAAGTC
ATCATGCCCCCTATGCCCTGGGCGACACACGTGTTACAATGGCCGGGACAAAGGGTCGCG
ATCCCGCCAGGGTGAGCTAACTCCAAAAACCCGTCCTAAGTTCGGATTGCAGGCTGCAAC
TCGCCTGCATGAAGCCGGAATCGCTAGTAATCGCCGGTCAGCCATACGGCGGTGAATTCG
TTCCCGGGCCTTGTACACACCGCCCGTCACACTGTGGGAGCTGGCTATGCCCGAAGTCGT
TACCTTAACCGCAAGGAGGGGGATGCCGAAGGCGGGGCTAGTGACTGGAGTGAAGTCGTA
ACAAGGTAGCCGTACTGGAAGGTGCGGCTGGATCACCTCCTTTTCAGGGAGAGCTAAAGA
AGCGAGCTACGTCTGAGCTAAGCTTGGAGATGGAAGTCTTCTTTCGTTTCTCGACGGTGA
AGTAAGACAAGCTCATGAGCTTATTATCCTAGGTCGGAACAAGTTGATAGGATCCCCTTT
TTTACGTCCCCATGCCCCTCCCGTGTGGCGACATGGGGGCGCAAAAAGGAAAGAGAGGGA
TGGGCTTTCTCTCGCTTTTGGCATAGCGGGCCTCCCCGTGGGGGGCCCGCAAGGGCTATT
AGCTCAGTGGTAGAGCGCGCCCCTGATAATTGCGTCGTTGTGCCTGGGCTGTGAGGGCTC
TCAGCCACATGGATAGTTCAATGTGCTCATCAGCGCCTGACCCGGGGATGTGGATCATCC
AAGGCACATTAGCATGGCGTACTCCTCCTGTTCGAACCGGAGTTTGAAACCAAACTTCTC
CTCAGGAGGATAGATGGGGCGATTCAGGTGAGATCCAATGTAGATCCAACTTTCTATTCA
CTCGTGGGATCCGGGCGGTCCGGGGGGGACCACCATGGCTCCTCTCTTCTCGAGAATCCA
TACATCCCTTATCAGTGTATGGACAGCTATCTCTCGAGCACAGGTTTAGGTTCGGCCTCA
ATGGGAAAATGGAGCACCTAACAACGCATCTTCACAGACCAAGAACTACGAGATCACCCC
TTTCATTCTGGGGTGACGGAGGGATCGTACCATTCGAGCCTTTTTTTTTCATGCTTTTCC
CGCGGAGGTCTGGAGAAAGCAGCAATCAATAGGATTTCCCTAATCCTCCCTTCCCGAAAG
GAAGAACGTGAAATTCTTTTTCCTTTCCGCAGGGACCAGGAGATTGGATCTAGCCATAAG
AAGAATGCTTGGTATAAATAACTCACTTCTTGGTCTTCGACCCCCTCAGTCACTACGAAC
GCCCCCCGATCAGTGCAATGGGATGTGTCTATTTATCTATCTCTTGACTCAAAATGGGAG
CAGGTTTGAAAAAGGATCTTAGAGTGTCTAGGGTTGGGCCAGGAGGGTCTCTTAACGCCT
TCTTTTTCTTCTCATCGGAGTTATTTCACAAATACTTGCCATGGTAAGGAAGAAGGTGGG
AACAAGCACACTTGGAGAGCGCAGTACAACGGAGAGTTGTATGCTGCGTTCGGGAAGGAT
GAATCGCCCCCGAAAAAGAATCTATTGATTCTCTCCCAATTGGTTGGATCGTAGGTGCGA
TGATTTACTTCACGGGCGAGGTCTCTGGTTCAAGTCCAGGATGGCCCAGCTGCGCCAGGG
AAAAGAATCGAAGAAGCATCTGACTCCTTCATGCATGCTCCACTGGGCTCGGGGGGATAT

AGCTCAGTTGGTAGAGCTCCGCTCTTGCAATTGGGTCGTTGCGATTACGGGTTGGATGTC
TAATTGTCCAGGCGGTAATGATAGTATCTTGTACCTGAACCGGTGGCTCACTTTTTCTAA
GTAATGGGGAAGAGGACCGAAACATGCCACTGAAAGACTCTACTGAGACAAAGATGGGCT
GTCAAGAACGTAGAGGAGGTAGGATGGGCAGTTGGTCAGATCTAGTATGGATCGTACATG
GACGATAGTTGGAGTCGGCGGCTCTCCTAGGGTTCCCTCATCTGGATCCCTGGGGAAGAG
GATCAAGTTGGCCCTTGCGAACAGCTTGATGCACTATCTCCCTTCAACCCTTTGAGCGAA
ATGTGGCAAAAGGAAGGAAAATCCATGGACCGACCCCATCGTCTCCACCCCGTAGGAACT
ACGAGATCACCCCAAGGACGCCTTCGGCATCCAGGGGTCACAGACCGACCATAGACCCTG
TTCAATAAGTGGAACGCATTAGCTGTCCGCTCTCCGGTTGGGCAGTAAGGGTCGGAGAAG
GGCAATCACTCATTCTTAAAACCAGCATTCTTAAGACCAAAGAGTCGGGCGGAAAAAGGG
GAAGAGCTCCCCGTTCCTGGTTCTCGTGTAGCTGGATCCTCCGGAACCACAAGAATCCTT
AGAATGGGATTCCAACTCAGCACCCTTTGAGATTTTGAGAAGAGTTGCTCTTTGGAGAGC
ACAGTACGATGAAAGTTGTAAGCTGTGTTCGGGGGGGAGTTATTGTCTATCGTTGGCCTC
TATGATAGAATCAGTCGGGGAGGCCCGAGAGGCGGTGGTTTACCCTGTGGCGGATGTCAG
CGGTTCGAGTCCGCTTATCTCCAGCTCGTGACCTTAGCCGATGCAAAGGTATATGATAGC
ACCCAATTTTTCCGATTCGGCAGTTCGATCTATGATTTCTCATTCATGGACGTTGATAAG
ATCCTTCCATTTAGCAGCACCTTAGGATGGCATAGCCAACACATTAATGGCGAGGTTCAA
ACGAGGAAAGGCTTACGGTGGATACCTAGGCACCCAGAGAGGAGGAAGGGCGTAGTAAGC
GACGAAATGCTTCGGGAAGTTGAAAATAAGCATAGATCCGGAGATTCCCGAATAGGTCAA
CCTTTCGAACTGCTGCTGAATCCATGGGCAGGCAAGAGACAACCTGGCGAACTGAAACAT
CTTAGTAGCCAGAGGAAAAGAAAGCAAAAGCGATTTCCGTAGTAGCGGCGAGCGAAATGG
GAGCAGCCTAAACCGTGAAAACGAGGTTGTGGGAGAGCAATACAAGCGTCGTGCTGCTAG
GCGAAGCGGTGGAGTGCTGCACCCTAGATGGATAGAGTCCAGTAGCCGAAAGCATCACTA
GCTTACGCTCTGACCCGAGTAGCATGGGGCACGTGGAATCCCGTGTGAATCAGCAAGGAC
CCCCTTGCAAGGCTAAATACTCCTGGGTGACCGATAGCGAAGTAGTACCGTGAGGGAAAG
GTGAAAAGAACCCCCATCGGGGAGTGAAATAGAACATGAAACCGTGAGCTCCCAAGCAGT
GGGAGGAGAAATCTCTGACCGCGTGCCTGTTGAAGAATGAGCCGGCGACTCTTAGGCAGT
GGCTTGGTTAAGGGAACCCACCGGAGCCGTAGCGAAAGCGCGTCTTCATAGGGTAATTGT
CACTGCTTATGGACCCGAACCTGGGTGATCTATCCATGACCAGGATGAAGCTTGGGTGAA
ACTAAGTGGAGGTCCGAACCGACTGATGTTGAAGAATCAGCGGATGAGTTGTGGTTAGGG
GTGAAATGCCACTCGAACCCAGAGCTAGCTGGTTCTCCCCGAAATGCGTTGAGGCGCAGC
AGTTGACTGGACATCTAGGGGTAAAGCGCTGTTTCGGTGCGGGCCGCGAGAGCGGTACCA
AATCGAGGCAAACTCTGAATACTAGATATGACCCCAAAATAACAGGGGTCAAGTTCGGAC
AGTGAGACGATGGGGGATAAGCTTCATCGTCGAGAGGGAAACAGCCCGGATCACCAGCTA
AGGCCCCTAAATGACCGCTCAGTAATAAAGGAGGTAGGGGTGCAGAGACAGCCAGGAGGT
TTGCCTAGAAGCAGCCACCCTTGAAAGAGTGCGTAATAGCTCACTGATCGAGTGCTCTTG
CGCCGAAGATGAACGGGGCTAAGCGATCTGCCGAAGCTGTGGGATGTAAAAATGCATCGG
TAGGGGAGCGTTCCGCTTAGAGGGAAGCTCCCGCGCGAGCAGGTGTGGACGAAGCGGAAG
CGAGAATGTCGGCTTGAGTAACGCAAACATTGGTGAGAATCCAATGCCCCGAAAACCTAA
GGGTTCCTCCGCAAGGTTCGTCCACGGAGGGTGAGTCAGGGCCTAAGATCAGGCCGAAAG
GCGTAGTCGATGGACAACAGGTGAATATTCCTGTACTACCCCTTGTTGGTCCCGAGGTAC
GGAGGAGGCTAGGTTAGCCGAAAGATGGTTATCGGTTCAAGGACGCAAGGTCACCTTGCT
TTTTTAGGGCAGGGTAAGAAGGGGTAGAGGAAATGCCCCGAGCCAATGTCCGAGTACCAG
GCGCTACGGCGCTGAAGTAACTCATGCCATACTCCCAGGAAAAGCTCGAAGGACCTTCAA
CAAAAGGGTACCTGTACCCGAAACCGACACAGGTGGGTAGGTAGAGAATACCTAGGGGCA
CGAGACAACTCTCTCTAAGGAACTCGGCAAAATAGCCCCGTAACTTCGGGAGAAGGGGTG
CCTCCTCACAAAGGAGGTCGCAGTGACCAGGCCCGGGCGACTGTTTACCAAAAACACAGG
TCTCCGCAAAGTCGTAAGACCATGTATGGGGGCTGACGCCTGCCCAGTGCCGGAAGGTTA
AGGAAGTTGGTGACCTGATGACAGGGGAGCCGGCGACCGAAGCCCCGGTGAACGGCGGCC
GTAACTATAACGGTCCTAAGGTAGCGAAATTCCTTGTCGGGTAAGTTCCGACCCGCACGA
AAGGCGTAACGATCTGGGCACTGTCTCGGAGAGAGGCTCGGTGAAATAGACATGTCTGTG
AAGATGCGGACTACCTGCACCTGGACAGAAAGACCCTATGAAGCTTTACTGTTCCCTGGG
ATTGGCTTTGGGCCTTTCCTGCGCAGCTTAGGTGGAGGGCGAAGAAGGCCTCCTTCCGGG
GGGGCCCGAGCCATCAGTGAGATACCACTCTGGAAGAGCTAGAATTCTAACCTTGTGTCA
GGACCTACGGGCCAAGGGACAGTCTCAGGTAGACAGTTTCTATGGGGCGTAGGCCTCCCA
AAAGGTAACGGAGGCGTGCAAAAGTTTCCTCGGGCCGGACGGAGATTGGCCCTCGAGTGC
AAAGGCAGAAGGGAGCTTGACTGCAAGACCCGCCCGTCGAGCAGGGACGAAAGTCGGCCT
TAGTGATCCGACGGTGTCGAGTGGAAGGGCCGTCGCTCAACGGATAAAAGTTACTCTAGG
GATAACAGGCTGATCTTCCCCAAGAGTTCACATCGACGGGAAGGTTTGGCACCTCGATGT
CGGCTCTTCGCCACCTGGGGCTGTAGTATGTTCCAAGGGTTGGGCTGTTCGCCCATTAAA
GCGGTACGTGAGCTGGGTTCAGAACGTCGTGAGACAGTTCGGTCCATATCCGGTGTGGGC
GTTAGGGCATTGAGAGGACCTTTCCCTAGTACGAGAGGACCGGGAAGGACGCACCTCTGG
TGTACCAGTTATCGTGCCCACGGTAAACGCTGGGTAGCCAAGTGCGGAGCGGATAACTGC
TGAAAGCATCTAAGTAGTAAGCCCACCCCAAGATGAGTGCTCTCCTATTCCGACTTCCCC
AGAGCCTCCGGTAGCACAGCCGAGACAGCGACGGGTTCTCTGCCCCTGCGGGGATGGAGC
GACAGAAGTCTTGAGAATCCAAGATAAGGTCACGGCGAGACGAGCCGTTTATCATTACGA

TAGGTGTCAAGCGGAAGTGCAGTGATGTATGCAGCTGAGGCATCCTAACAGACCGAGAGA
TTTGAACCTTGTTCCTACATGACCCGATCAATTCGATCAGGCACTCGCCATCTATTTTCA
TTGTTCAACTGTTTGACAACATGAAAAAACCAAAAGCTCTGCCCTCCCTCTCTATCTATC
CAAGGGATGGAAGGGCAGAGGCCTTTGGTGTCCCTTCCAGTCAAGAATTTGGGCTTCACA
ATCACTAGCCAATATTTCTCTCATACCTTTCTTCGTTCATGGTTCGATATTCTGGTGTCC
TAGGCGTAGAGGAACCACACCAATCCATCCCGGAACTTGGTGGTTAAACTCTACTGCGGT
GACGATACTGTAGGGGAGGTCCTGCGGAAAAATAGCTCGACGCCAGGATGATAAAAAGCT
TAACACCTCTTATTCTTATTACTCAAAAAGAAAAAAATGAAAAGGTCGTCTTATTCAAAA
CCCAATTATGACATCCCTTCTCTCCCACTTCACACCTCGGAACGCGCTGTTCTTATAGAG
AGAAAGGCGCTTTCACGTCTTCTTAACCCGAAATGGCTGAGGGGAGAAAAGGTTCCTTTT
TGAGGGTACTCCAGGGAACAGATCCAGTGGAGACGGGGTGGGGCCTGTAGCTCAGAGGAT
TAGAGCACGTGGCTACGAACCACGGTGTCGGGGGTTCGAATCCCTCCTCGCCCACAACCG
GCCCAAAAGGGAAGGACCTTTCCTTTACCTCTGGGGGTAGGAAAATCATGATCGGGATAG
CGGACGCAAAGCTATGGAACTGGGGTGTGGGTCTTTTGTCGAAATGGCCTTATTCTTTTT
ATTTATCGTGAAGGAAAAAATCGATACATATAGTATGCCTGGCCCGAATCAGCATATTTG
TGTTTTACTCCCCGTAACTCTTCCTCAGCCAGGCTTGGGAAGAATAGCAGAGCAAATACA
AGTATTAGTAGCATAGCAAAAATGCGTTCCTCGTCATTAATATGTTTGCTCGCGGTAATT
GTGGCCTCTCGGGAGAATCGATGACTGCATCTTTGATGCACTGCTAGTACATCATCTGAG
AATTATGAATTGGCTAGTTGTAAATAGCCCCAGGGCTATGGAACAAAGGATTATCCCGGA
TCTACACCGAGGTATTGACGGTGATTCTCAAATATCGCAGAACAGAATGCGATGAGATAG
AGTGCAATAGAAACAAAGACAGGGAACGGGTTACCTACTCCTAACGGTCAAAGCGAGCCC
TTTAATTCTGTAATTCTGAATTCTTTAATTAAGAATTCATCAAATCTCCCCAAGTAGGAT
TCGAACCTACGACCAGTCAGTTAACAGCCAACCGCTCTACCACTGAGCTACTGAGGAATA
ACGGGAGATTCGATCTCATAGAGTTCAACTCCCGCTCTCAACCCATGACCAATATGAGCC
CGAAGCTTCCTTCGTAACTCCCGGAACTTCTTCGTAGTGGCTCCGTTCCATGCCTCATTT
CATAGGGAACCTCAAAGTGGCTCTATTTCATTATATTCCATCCATATCCCAATATTCCAT
CCATATCCCAATTCCATTCATTTAATATCCCTTTGGTGTCATTGACATAAGAGATGTCAT
TCAATCGAAGAAGAGGGTATCATTGACATAAAAGATGTCATTTCTAGTCTATCTGTTTCT
ATCTATGGAAAGTGAAGAAATCATCATATAGTAATCGAGAAATTGCAATAGAAAAGAAAA
AAGGGAGGTTTGTGATGATTTTGAAATCTTTTCTACTAGGTAATCTATTATCCTTATGCA
TGAAGATAATAAATTCGGTCGTTGTGGTCGGACTCTATTATGGATTTCTGACCACATTCT
CCATAGGGCCCTCTTATCTTTTCCTTCTCCGAGCTAGGATTATGGAAGAAGGAACCGAGA
AGGAGGTATCAGCAACAACTGGTTTTATTACGGGACAGCTCATGATGTTCATATCGATCT
ATTATGCGCCTCTGCATCTAGCATTGGGTAGACCTCATACAATAACTGTCCTAGTTCTAC
CGTATCTTTTGTTTCATTTCTTCTGGAACAATCACAAACACTTTTTGGATTATGGATCTA
CTACCAGAAATTCAATGCGTAATCTCAGCATTCAATGTGTATTCCTGAATAATCTCATTT
TTCAATTATTCAACCATTTCATTTTACCAAGTTCAACGTTAGTCAGATTAGTCAACATTT
ATATGTTTCGATGCAACAACAAGATGTTATTTGTAACAAGTAGTTTTGTTGGTTGGTTAA
TTGGTCACATTTTCTTCATGAAATGGGTTGGATTGGTATTATTCTGGATACGGCAAAATC
ATTCTATTAGATCGAATGTACTTATTCGATCTAATAAGTACCTTGTGTCAGAATTGAGAA
ATTCTATGGCTCGAATCTTTACTATTCTCTTATTTATCACCTGTGTCTACTATTTAGGCA
GAATACCGTCGCCTATTGTCACTAAGAAACTGAAAGAAACCTCAAAAACGGAAGAAAGGG
GGGAAAGTGAGGAAGAAACAGATGTAGAAATAGAAAAAACTTCCGAAACGAAGGGGACTA
AACAGGAACAAGAGGGATCCACCGAAGAAGACCCTTCCCTTTGTTCGGAAGAAAGGGAGG
ATCCAAAAAAACTACATGAAAAAAAAAAGAGGCAAGAAATTTTGAAGTTAGAAATACTTA
AAGAGAAAGAAGATAAAGACCTCTTCTGGTTTGAAAAACCTCTTGTGAATCTTCTTTTCG
ACTATAAACGATGTAATCGTCCATTGAGATATATAAAAAAAAATTTATTTCAAAATGCTG
TAAGAAATGAAATGTCACAATATTTTTTTCACGTATGTCCAGTTGATGGAAAACAAATAA
TATCTTTTACATATCCACCCAGTTTATCGATTTTTTTGGAAATGATGCAAAGAAAGATGT
CTTTGTGTACGACCGAAAAACTATCCCCCGAAGATCTGTATAATCATTGGGTTTATACCA
ATGAACAAAAAAGGTACAGCTTGAGCAATGAATTCATAAACCGAATAGAAGTTCTAAACA
AGGGATCTCTTACTATGGATGTGCTTGAAAAAAGGACCAGATTGTATAATGATAAAAATA
ACCAAGTCTAGTCACTTCTTATTCTATTCAAAACAAAAAGAATAGATAATAGATATAGTA
AATTAATAAAAAGCCGGATACATATGATAAATATACAAAGAGATAAGAAGAGATTCGCCC
TCCCCCCACATATTTGATCCCTTCTCCTACAAAGAAACTTGCAACACCAACACCATTGGT
AATTCCGTCAATTACCCGTCTATCTAAAAAATGAGTTACTTCAGCTAATCCTCTTATACT
TGTAGTTAAGCATGTTGCATAAAAAAGATCTATGTAACCACGATTATATGACCAATTGTA
TATCACATTTATTATTCGGTCCAATAAAATTTTCTTAGAGCCTGTTTTTTTAACAAATGA
ATTGATTAAGTCCAAATTCTGAAAAGATGAATTAACAGACCCATATAAAATAGACGCTAT
GAATATTCCAAAACAGGCTATACTGACTGAATAAATTGCATTTGTCACAAATTCATACCA
ATCCACAGAATAGTTCGAATTTTGATGTAAAAGGTTTATTGATGGAGTTAACCATTTCGA
TAATATATCAAAATCGATTACTCCTTGATCGAAAGGAATTCCTATGGATCCAACGAACAA
AGTAAATAGGACCAATATAAGTAGAGGCAAAAGCATAGTATTGTCTGATTCATGGGGATA
CGTTGAAGTGTCTTTATTTTCAAAATAAATCCTACAGGAGCGTATCAGATTTCTTACATT
TCCGTTCATTTTGTATATCTTCTTCGAAAAAAAGGAAACCTTTTCATTATTATTCATTGT
TGATAAAAACAAATTTCTGTTAACTGGTTTGGTTCCTTCTTTCCCCCATATAGATATTGA

ATAGAACGAGCTATTTTGAGTGCCACTGTAATTTTGAAAATGAGCATGTAAATGACCATC
AAAAGTAAGTAAATACATCCGAAACATATAAAATGCAGTTAACCCCGCCGAGAAACAAGC
TATTATCGCGAAAATAGGTGAATACAACCAACTATCATTAAGAATTTCATCTTTAGACCA
AAAACAAGCAAGAGGTGGAATTCCACAAAGAGAAAGTGTACCTAATAAAAAAGTATTTTT
TGTAATTGGCACATATTTTGTTAAACCACCCATAAGAACCATGTTCTGACTTTTATCTGG
AGAATATCCAACAATGGGTTCCATTGAATGAATAATTGATCCGGATCCTAAAAACAATAA
TGCTTTCGAATAGGCATGAGTGATCAAATGGAATAAAGCAGCTCGATAAGAGCCTATCCC
TGGGGCTAACATAATATAACCCAATTGAGACATTGTAGAATAGGCTAAACTTCTCTTAAT
GTCTCTTTGAGCAAGAGCTAAAGTAGCTCCTAATAGTACCGTTATTACACCTATCAAAGA
AATGAGATTCATTATGTAAGGTATGACTGTGAAAAGCGGAAGAAATCGAGCGACAAGAAA
AATGCCTGCTGCTACCATAGTAGCAGCATGGATAAGAGCTGAAATAGGAGTAGGCCCCTC
CATGGCATCAGGTAACCATACATGAAGGGGAAATTGTGCGGATTTAGCAACTGCACCGAC
GAATAATAGGGAGGCACACAGAGTAGCAAATAAAGAGTTGACCCCATTATTACGGATCAG
GTTATTGAAGATTTCGAACAAATCTCGAAATTCGAAACTCCCTGTTATCCAATAAAAACC
TAAGATTCCTAATAATAACCCAAAATCCCCTACACGATTAGTTACAAACGCTTTTTGACA
AGCATTTGCGGCAGCCGGTCGTGTGAACCAAAAACCTATTAATAAATACGAACACATTCC
CACTAGTTCCCAAAAAATATGAATTTGTATCAAATTGGAACTAGTAACTAATCCCAACAT
GGAAGTATTGGAAAAACTCATATAAGCAAAAAATCTCAAATATCCTTGATCATGAGACAT
ATAATTGTCACTATAAATAAGAACCATGATTCCAACCGTAGTGATTAGTATTGACATAAT
AGAAGTAAGTGGATCGATCAAGTAGCCGAACTCTAAGGAAAAATCAGTATTGATGGTCCA
AGACCATAGATGTTGATAGATAGAACTGCCATTTATCTGTTGAATAGACAGATCGGACGA
AAAAACCATAACTATACTTAGCAATGAAACACTAGGAAAAGTCCACATACGACGCAGATT
TTTTGTTGCGGTCGGAACAAGCAGAAGTCCCAACCCTATTGACATAGTAACTGGAAGTAG
AGCGAAAGGTATGATCCATGCATATTGATATGTATGTTCCATAAGAAAATCAAACTTTCT
TTTTTTATTCTTATTAATTGTTTCCCATTCACCAGCCCTTATTTCTTCCGGAAGGAGCAA
TAATCAAATAAATAAAAAAAAAAATCAAGATATACAAGATATAAAAGAACTCAAATATGAT
TTTTCATTCTTAATTATTCTGATTCTTTCCAAACTATTGAAAAAAAAAAAAAAAACAAAAA
ATTCAAATAATCAAGTTACTAGTTAAAAGCTACTACCTAGTTATTAACGAAGATATTTAT
TAAGATAAAAAATACGAGATTTTCAATTATTCTACTATATACATACGATACGGTAATTTC
TATCCATATTTATATCAATATAGTAAGGAAAAAGAATGATACCCAAATTTCAAGTACTTT
ATTCTGATATGTATCGTAGGATCTGCCAATAACTCGATCCAATAAACCTAGTTTTTTTTT
AGTTTCTTCGGAGTCATTAACTAAAACAAATTCTGGAATTGATTGGCTTCTAGTCCAATA
AAACAAACTTATGTTTATGTGTTTATGAAAAATGCTAGAATACTTGTCACTTCGCATAGA
ACTAAAAAGAGAAATTACTCAAATTCCCTTTATTTTATCAAGTAATGTATTGTTTAACGG
ATTAACTACTTTAGATTTAATTTCAATTTAAATTATGTGGACTCTATCTCCGAGCTTGAT
CAATTAATAGAAAAAGGTTACATTCATTATTGGTTTCCTAAATTAGGAAAGGGTTCTTAA
GCTTTTCGATTTATTAAAAATAACATTTATAATATATATATATATTATCTAAATAGACTG
AGATATGAATTGGAACCTTTTTAATTCTTATCAATGGCATCCGATTCAACGGTAGTAGAT
CCCGCCCGTAGACATAGATTGAATAAAGATATGAAGCCCCCAATCAGTACAAATTATTCT
TTCTTCGAACATTGGATGTAATGGAAATGTGAAAAAAAAAAAAAATTCCCTTGAAAATCA
CATCGTTTTTTCTTTTTTCTTCTATTTCATTTCTTTTTTTTATCCTTAATGATACCATATG
CGATGCTGATCTATCTGTATCCATACTCTTCTATGTTATGAAGTAAGCATAAGTATCGGC
TATGGAATAAAGATAGATAATGAATAGTTAATAGAAAGAACAATCTATTTTGAATTGAAT
AATAAATGTCTTTCACGTCCAACTATAAAAATGAGTGACCCTTTTATTTTGAAATGGCGG
TTCCAAAGAAACGTACTTCTCTGTCAAAAAAGCATATTCGTAGAAATATTTGGAAAGGAA
GGGGATATCAGGCCGCGGCAAAAGCTTTATCTTTAGCTAAATCTATTTCTACCGGGCATT
CAAAAAGTTTTTTTGTGCGACAAACAAGTAATAAAGCCTTGGAATGATCTGAATCTGCAT
CAGCATGACTCGATGAATTGGATGATTAAATTTATAAGATGAAGAATTCACATTAACTAA
TGTTTTGAACTCATCAATATGAGATAGAACTAGCTGTTGTGGTATGTAGAATATGAAGTA
TTCTGTATACTAGGTTCTAAAAATGATTTCTTTTTTTGTTTGAAAAAAAAAAAAAAAGAAA
GAAAAAGAAGTAGTTAGACACAAAATACAAGGTTTCACCTTTCATTGATTGGTTTTTATA
ATTCGCGAGATGGGGATTGTAACTTTCCCCATCGATTCATTTTTCACAATAACAAAACTC
TTCCTTTTTTTTCTTAGGAAGGGATTGCCTTATTGGATTATGTATCTCCAATAGTTATACA
TCATTAAGATTTTTGGAAAATCTGAAACAAGTATGAACGAGGTTAGAACCCCCTTTTTTG
TTCCAAAGTAAGGCGGGGGTAAGATTCATAGTCAAAGTCAATGGATTATTGAAACTAATT
GATTAAAATATACATTTTAAAACTTTGATTTGTAGCTCTCTGAATCACTACATATCTACA
AGGACTCCCTCTTGTTTCGAACAGATAAAAAAAAAAAACACAAAATAATAAAACTGCCAG
GATCCCATTTAGATCGATATTTATGTACTAAATAATGAGTCAATCTTACGTTACGTAATC
CAACCCCAATGGATCCTATCCCATGTTTGAGCTGGAGGATCGATCAATCGATATATCTAG
ATCCAATATCTAAATTTTTTTATCTATTAATATTAGATTTCAAATCTATATATATAAAGAGA
TCTTATCAGTTTAAATTTGATTTTCTAAGTTTTCTAAGTTAAAGTACATACAGTAGAATT
CCGATAAAGATTGAAACTCTTTTGTTATACGATATGCCCGGTCCAATACCTAATGGGTTT
GGTAAATCCTCACACAAATTATGTTCTGTATACAATGAAGATCCCAATCATTAATACATC
TTTGATACCAAACTATCCAAATTTTCATAGAAATCCTTTGGATGTAGTGATGAAGTTGTG
ATATATAAAAAATCTTGTTTTATTTTGTTCATTGAAAAATGAATCTTTTTCATTTCGGAT
CTATCAAATCAGATAAATGAGAAATGAATCGTCAAAAAATGAGAAAAAAAATTCTTAGTTC

TATACCTGGACTCAGGGCATAACCGTCTAGTTCCAACTATTCCAGAAGAACTTATAATAC
GGAAAAGCCCGCTGTTTAAAACGACCCCCTGCCACGATACTAAGGATTATTGAGCGTTTA
AATATTTATTTGAACGGGTCTTTATTCATCGATTCTAATATTTCCTAAAATAGATTGCAT
TAAATCCCTCAATCTCGACGATTGAACATCATATGGACTATGATTATTTCGATGATGCCG
CCATGGTGAAATTGGTAGACACGCTGCTCTTAGGAAGCAGTGCTAGAGCATCTCGGTTCG
AGTCCGAGTGGCGGCATCCTCTAAAAAAGGCACAATAGATCCTATAATGAATTCAATTCC
GGATTTCCATTCCGTAATTGGGGATACCCCCCTAAAAAAAATTATGATATTTGCCACTTT
AGAACATATATTAACTCACATCTCTTTTTCTATCATTTCAATTGTTATTACGATTCATTT
GATGACCTTATTAATCCATGAAACCGTAGTACTATTTGATTTGTCAGAAAAAGCCATGAT
GGCTACCTTTTTCTGTATAACAGGATTATTAGTTACTCGTTGGATTTATTCGAGACATTT
GCCGTTAAGCGATTTATATGAATCTTTAATGTTTCTTTCATGGAGTTTCTCCATTATTCA
TATGTTTCCTAAAAGACGGAACCAGAAAAGTTATTTAAGCGCAATAACCGCGCCAAGTGC
TATTTTTACCCAAGGCTTTGCCACTTCGGGTCTTTCAACCGAAATGCATCAATCTGCAAT
ATTAGTACCTGCTCTACAATCCCAGTGGTTAATGATGCACGTAAGTATGATGTTATTGAG
TTATGCAGCTCTTTTATGTGGATCGTTATTATCCATAGCTCTTTTAGTCATTACATTTCG
AAAAAACCTAGATATTCCTCGCAAAAGCAATCATTCATTAATTGGGTCATTTTCCTTTGT
GAATGAAAAAAGAAGCGTTTTACAAAACACTTCTTTTCTTTCATTTAGAAATTATCACAG
GTATCAATTAACTCAACAATTAGATCAGTGTAGTTATCGTGTCATTAGTCTAGGGTTTAC
TTTTTTAACCATAGGTATTCTTTCGGGAGCAGTATGGGCTAATGAGGCATGGGGGTCTTA
TTGGAATTGGGACCCCAAGGAAACTTGGGCATTTATTACTTGGACCATATTCGCGATTTA
TTTACACAGTAGAACAAATCAGAGCTTTCAGGGTGTGGATTCGGCAATTGTGGCTTCTAT
AGGATTTCTTATAATTTGGATATGCTATTTTGGGGTCAATCTATTAGGAATAGGACTACA
TAGTTATGGTTCATTCACATTAACAACTAATTGAAGAAAGGGCCTGAAGAATACATAGGA
ACATCGTCCCGTATATTATATAAAGAAGGTTTGTGCAAGTTTTTTCGAACCATTTGAATC
AAGTAGTGATTCAAATGGTTCGAAAAAACTTTAGATGTATCTGATTATAATTCACTTCAT
TTTTTTTTTCTTTCATTGAACGCTTTTAAAAATCACACAGTTCTTTTACATTAATGTAAT
GTCTTATTGATGAAAACTATTTATGAAAATAAATAGATAGAATAGCTTCTATCCTGTCAA
TTGATAGCGAGAGAACGAAATCAGGATAAATACCAATACCTATTACAGGTAGAAGGATAC
AGACCGAAACAAATAGTTCTCGTGGTCCAGAATCAAAAAAATAAGAGTTTGGAACGTTGA
ATAGCTTGTAGCCATAGAACATCCGACGTGACATAGATAATGAATAAATAGGAGTTAATA
TCATTCCAATTGCCATTACGAAAGTTATTAGTATTTTTGGCATTAAAAGATATTTCGGGC
TAGTAATTATTCCAAAAAATACTACTGATTCCGCAACAAAACCACTCATTCCTGGCAATG
CAAGAGAAGCCATCGAGAAGCTACTGAACATGGTAAATATTTTTGGCATTGGGATAGCTA
TTCCTCCCATTTCGTCGAGATAAACAAGACGTATTCTATCGTAGCTCGTTCCTGCCAAGA
AAAAAAGTGCAGCACCAATAAATCCATGAGAGATTATTTGTAAAATGGCTCCATTGATTC
CCGTATCGGTTATGGAACCAATTCCTATAAGTGTGAAACCCATATGAGATACGGAAGAAT
AGGCTATTCTCTTTTTTAAATTGCGTTGACCGAAAGAAGTTGAAGCTGCATAGATTATTT
GAATCGCTCCTACTATCATCAACCAGGGAGAAAATATAGAATGAGCATGGGGTAATAATT
CCATATTGATCCGAACCAATCCATACGCTCCCATTTTTAATAAGATTCCCGCTAGAAGCA
TACATGTACTGTAATGTGCTTCTCCATGGGTATCTGGTAACCATGTATGTAGGGGTATAA
TCGGCGATTTGACAGCATAAGCAATAAGGAAGCCAATATAGAATATTATTTCCAATCCCA
AAGGATACGATTGATTAGCTAATGTTTCAAAATTTAATGTTGGCTCATTGGAGCCATATA
AACCCATACCCGGAACTCCCATTAAGAGAAAAATAGAACCCCCCGCTGTGTACAAAATAA
ACTTTGTAGCTGAGTACAGACGTTTCTTCCCTCCCCATATGGATACAAGTAGGTAAACAG
GAATTAATTCTAATTCCCACATGAGGAAAAAAAGTAAAAGGTCTCGAGAGGAAAATGATC
CTATTTGACCACTATACATTGCTAACATCAGGAAATGGAACAATCGCGAATCTCTAGTAA
CTGGCCGAGCCGCTAAAGTAGCTAAAGTAGTGATGAATCCCGTCAGTAAAATGGGTCCTA
TGGAAAGTCCATCGATTCCCGGTCTCCAGTGAAAATCAAAAGTATTTATCCATTTATAAG
CCTCTTCTAATTGGATTAATGGATCGTCCAATTGGAAATGATAACAGAACGCATAGGTCG
TTAGAAGGAGTTCTAATAAGCATATACAAATAGTATACCACCGAACCACCTTATTTTTAT
TCCCTCTACGAGGGAGAAAGAAAATTGACGAACCCGCGGATATCGGCAAAACAACAATTA
TTGTTAACCAAGGAAAATAACTCGTGGTAAAGACAAGATAGACTTTGACCAGAAAAACCC
GCGCTCGGAATAATTTCTCGAGTACGGGTTTTTGTCGGTAAAGAGGAATCAAATGGATTC
AAGTGGATTTTTCTAGAACGTATCAATAAGCTAGACCCATGCTGCGAGTTGTCTCATGCC
ATAAGTAAACCCGAACACTCAAGAAATCCGTTGGACAAGCGGATTCACATCTCTTACAAC
CTACACAGTCCTCTGTTCTTGGAGCAGAAGCAATTTGTTTAGCTTTACATCCGTCCCAAG
GTATCATTTCCAATACATCTGTGGGGCAGGCTCGTACACATTGAGTACACCCTATACATG
TATCATAAATCTTTACTGAATGCGACATTGGATCTATAAATTTTTTGAACTTTATTAATT
TTCGATCTGGCTTCATTAGTAATTGAATTATATATATTATGTTGTAGACGCCAGACGAAT
CAGTGGTTTACCAGAACTTTTTTGATAATAAATCTATTTCTGGATCTGTTCATGAGCAAG
GGCCAAGATACTTTGATTTCTTACGTTTCAACAAGCATGGTCATACGAATCATACATGCT
AGTTCTAAATTAGTGAATATTTTGTGGATATCTGTCTTTATTTATATCATTAATACTATT
TATTCAACAAATTCGATTGATTGATACGAGTTGATTTTCTGTTACGATGGATCGATGAAA
CAATAGCCGGCCCAATAGCTGCTTCAGCGGCTGCAATAGCTATAACAAAAATCGAGAAAA
TATCTCCTTTTAATTGACGACTATCAAACAAATCAGAAAATGTTACGAGATTTATATTAA
CCGCATTCAGTATAAGTTCAAGACACATAAGTGCTCTAACCATGTTTCGACTTGTGATCA

ATCCATAAATACCGATAGAAAATAAATAGGCACTCAAAATAAGTACATGTTCGGTCATCA
TTGACCAACCCCTCATCAATTTTGATTCATTTCAATATGAACAACAATTTCACCGATTTG
ATTAGAATATAAATGAAGTACGAAACAAAGTAAGGTATTAGTAATGGATCGAACTAAACC
CCTTTCAATTTCATATATGAACAATAGAATATGAAAATGGAACTGAAGGAAAATGGATGT
GATAACATAGAACAAAACAAGACTTTATTTATTTTATTTTGGATCTAAGTATTTA
TTACTTAATTACTTTACTGCCGGGCCATAGCAATTGCACCTATCAAAGCAACTAAAAGAA
TTATAGAAATGAGTTCAAATGGAAGGTAAAAATCTGTTGATAAATGAATCCCAATTTGTT
GAACGTTACTTGTTAGGTCCTGCTCTATAATCTGATTTGATCTTGTAGTCCAAACAATCC
CGTACCACGACGTATCCGAGATAGTAGTAATTAGTGAAAAAAGAATACTTGTACAAACCA
GTGAAGTGACCCCATCCCCAACGGTCCAAAGATAGAAATCTTTGTAATATTCTGAACCAT
TCATGAACATCACAGCAAATAGGATTAAAACATTTACAGCTCCTACGTAAATAAGGAGCT
GTGCAGCAGCTACAAAATAGGAGTTAGATGGAATATGGAATAAGGATATACAAACAAGAA
CCAGTCCCAATGAAAAAGCAGAATAAATTGGGTTGGTAAGTAAGACCACCCCCAGACCCC
CTAATATAAGACCTGATCCCAGAAATACTAAAAGAATATCATGTATTGGTCCAGGTAAAT
CCATTATGTGTAAAAAAAGAGATAAATAAATCGAAATATTTCATAAACTTACTAACCGAC
CCAAGAAAAAAGAGGTTACCTTATTTTTTTCTTGTATATGATACGTTCCTAATTGAATCC
TAAAGGGGTGTAGATTTATATAGATACAGTTATTGGATCAATCCCGCTACTGTATCTGAA
AGAGTAGTTCGAAATTGTATATTTTGAAATCATCACAGATCTATGAATCCATTCTAAATC
AAAATCAAGCCTTGATTCTCACCAATCAATAGTTATTTACTGACCTAGCAAAATGAAAGA
AGCCTCACTCCTTTACTTTAAATCAAAACGGAATCTTAGTAATTGGTAATCGTTTTTGAA
TCAAGAGGTTTACCCCTGATTATTTTGATTGGAGTCGAATTCGTAATTGTTCGAATTGTG
TAATCTCCAATTACTGACATTGGTAACCGGCCCAAAGCAATTTGATTATAATTCAATTCG
TGACGATCATAAGTAGAAAGTTCATATTCTTCAGTCATTGATAAACAGTTTGTTGGACAA
TACTCGACACAATTACCACAAAATATACAGATTCCAAAATCAATACTATAATTAAGCAAT
CGTTTCTTTCTAATATCCGTTTCCAATCTCCAATGAACAACGGGTAGATCTATGGGGCAT
ACCCGAACACATACTTCACAAGCAATGCATTTATCAAATTCAAAATGGATTCGACCACGA
AAACGCTCCGATGTGATCGACTTTTCATAAGGATATTGAATAGTCACAGGTAAACGATTC
ACGTGAGATAAGGTAATCATGAAACTTTGACCAATATACCTTGCAGCTCGTATTGTCTGT
TGACCATAATTCATGAACCCAGTCACCATAGGGAACATATCGTGGATATCCATGAATAGT
TTGATGTTTCTTTCTCTTGCTCTAGATAGGTTATGAATCTAGAATATCATATTTTGTTAT
AGCGAAACGAGTTGGGAAGAAGTTGTTAATAATAGATTACCTAGAGAAATAGGTAAAAGA
AATTTCCACCCAAGGTTTAATAGCTGGTCCATTCTCATCCTAGGTAAAGTCCATCTTGTT
GTGATAGGAATGAACAGGAACAAATAAGCTTTAGCTAATGTAATAAGGATACCAATTGTC
ATTCCAAAGACTCCACCTGTTTTATTTATTCCAAAAGGTTCAGAAATGAATATGTACGGA
ATAGAGAAATTCCACCCGCCCAAGTAAAGAACTGTTACAAATAATGAAGAAACTAGTAGA
TTTAGGTAAGAAGCAACGTAAAATAAACCAGATTTAATACCTGAATATTCGGTTTGATAA
CCTGCTACTAATTCCTCCTCTGCTTCTGGTAAATCAAAAGGTAATCTTTCACATTCCGCT
AGGGAAGAAATTAGAAAAACTATAAACCCTATAGGTTGACGCCACAGATTCCACCCCCAA
AACCCATATTTTGACTGTGCCTCAACTATATCAACTGTACTTGAACTGTTAGATAATCAT
AGTCGATGATAACATCACTATTCCCATCGCTATTCCAGAACCGCACATGAGACCTCAGCT
TCATACGGCTCCTCGATGGCCACAAATAAATCTAAGGACTGGTTCATTATTACCTCGGCA
TGTTTGTAGTGTAGATTAGAGTAAAGATGTATCGAAGAGTCCCGAATTAGACCAATGGAA
TTCCGTCCGCCATAATAATAAAAAAAAAAAAAAAGCGCTTCCGAATTGATCTCATCCTTTA
TTTTCTAATTAGACTTAGAATTCTTTTAGTTCAGTAATAACTTAATCCTTCAATAAAATA
CTCGTTGTTTCAATAGATATTTCGACCCATACTTTTCGTACGAAAAAGAATTAGACACTA
ATTCATGAGTTATAGTTGATAAATCATTATAAGAATTCTATCCGTATAAATATGGATAGG
ATTGAGGAAAGAAAGAATAAACAAAGATCTCTTATTATTCAGTTTTCCTATTGCATTCCA
TTTCTTTCGTTCCTGTTCTTCTTTCTCACTCAGAAGGGGGTTTCTAAAAAATCAAATCAA
AGGATTACTTCGTTCTCGACAGTCATTTATTTAATCAGTGGATAGAAGCATACTCTGGAT
CGGAATCGTGAGGAAGTACTGCTTGATCATTTCTACGAACTTAAAGCCCTCATTATGATT
CCTTTTATGTTATGCAGAAATATCCCTTTGGATACCTTACATTATCTTCATCACTAATCC
TTTGTGTACCTTTGTGTTCCTAACCGTCCACTCATTTTTGATTGATCCCCGGTATGGTAA
TACATACAACATACACAACATACAACAACATACTATACAATAGTAGAAACTCCATACAGT
TGATCTTTTGCACCCGCTTCAAGTCATGATGACTAATCAACCAATCTTGGGGTAAACAGT
TTCGACCGCTTATGTTTACTTCCACTTTACTCTCGTACATAGGGAATGAGAATCAATCTT
CTTACTGCAAATTCATAAGCTGTTTTGTTTCACTCATATAACTATCTGGTTTAACTCATC
GACCCGAATGATGAATAAAAAGAGAAAGGTATCTATTCAACACATCCAACCCCTTTCCTT
TATTTCCAGGAAAGGGGTAAAGGTTCATGTTCCAACGAATCACACGTAGAGATATTGATA
ACACACACGGAGTTAATGGTATTTCATAACTAATAGATTGAGCAGCAGCTCGTAGACCAC
CTGAAAAGGAATATTTATTATTCGATCCATATCCTGACATAAGAAGTCCAATAGGAGCAA
TACTGGAAATAGCGATCCATAAAAAAACGCCTATACTGAGATCGGCTAGAACAAGGCGAT
AGCCAAAAGGAATTACTAAATAGCTTAGTAGAATTGATATGACCGCTATAGATGGCCCCA
TACTGAATAAACGAACATCTCCTCTAGATGGGAGAAGATCCTCTTTCAAAAGTAGTTTGG
TCCCATCTGCTAGAGCTTGAAGAATTCCCAAGGGGCCGGCATATTCAGGCCCGATACGTT
GTTGTATCCCTGCAGATATTTCTCTTTCTAACCACACAATCACGAGTACGCCTATTGTGA
TTCCCGATACAGGAGTAAAAATAGGGACAAGCAGCCATAAGAGGTCATAGACCTCTTTTA

AGGATTCCGATCTGGAAAAAGAATTGATAGCTTGTACTTCTGTCGTATCAATTATCATTT
CAACGATCAACTTCTCCCATAATGATATCTATACTACCTAGTATCGTCATGATATCCGCC
AATTTCATTCTTTTAACTAGCTGAGGAAGAATTTGCAAATTGATGAAACCAGGTGGACGA
ATTTTCCATCTCCAGGGAAAAACACTATTATCCCCTATCAGAAAAATTCCCAATTCTCCC
TTTGGGGCTTCGACTCTCACATAAAGTTCTTGTTTCGACAATTCAAAAGTGGGAGAAGGC
TTTTTACTAATGAATCGATATTCAAAACCATTCCATTCGGAATCACTTGCTCTATCAAAG
CGTCGAACTTCTAAGTTCTCATAGGGCCCCCCGGAATTCCTTCTAGAGCCTGTTGAATA
ATTTTTATGGATTCCGTCATTTCATTGATTCGTACTAAATAACGAGCTAATGAGTCTCCT
TCTTTTTGCCACTGGACTTCCCAATCGAATTCATCGTAACACTCATAATGATCAACTTTA
CGAAGATCCCATTGGATTCCGGAAGCTCGTAGCATTGGTCCCGATAAACCCCAATTTATT
GCTTCCTCCCCACCAATAATGCCCACCCCTTCAACTCGTTCCAAAAAAATGGGATTTTGC
GTAATAAGCTTTTGATATTCAACAATTCCTGTTAAAGAATAATCGCAGAAATCCAAACAT
TTATCTATCCAGCCATGAGGTAGATCAGCAGCGACTCCCCCGATACGGAAATAATTATGC
ATCATTCGCATACCTGTGGCAGCTTCGAATAGGTCATATAGCAATTCCCTTTCTCTGAAA
ATATAGAAGAAGGGAGTCTGTGAACCGATATCTGCCATAAAAGGTCCAAGCCATAATAAA
TGAGAAGCTATACGACTCAGCTCCAGCATAATTACTCTGATATAGCTGGCTCTTTTGGGT
ACTTGAATATTTCCTAATTGTTCTGGTGCATTTACCGTTATTGCCTCTGTGAACATAGTA
GCTAAATAATCCCAACGTGTTACATAAGGAAGATATTGTATAATTGTTCGGTTTTCCGCA
ATTTTTTCCATCCCTCTGTGTAAATAACCCAATACGGGTTCGCAGTCAATAACATCTTCA
CCATCTAGAGTAACGATAAGTCGAAGAACACCATGCATTGATGGGTGGTGAGGACCCATA
TTAACTATCATGAGGTCTTTTCTTGTAGCCGGTACATTCATATGTTTTCTTCTCCGATCC
ATTATTCTATGAATTGCCGAAAAGGAAATAAATGAGGTTCATCAAAATTCAAGATCTAAT
AAACCAAAGAATTCAAATAATCTTCAAATTAACGAGTTTTTGGTTCCCGAATATCTAATT
GATCGATTAATTTTTTATAACGCACTCTATTTTTCTTTGACAAATAAGCCAGCAGTCGTT
GACGTTTTCCCAGAATTTTCCGCAAACCTATCTGAGATAAATAATCTTTTCTGTGCAATT
CAAAATGTGAAGTAAGTCTCTGTATCTTATTGGTGAAACTGATTACTTGAAATTCAACAG
ACCCTTTGTTTTTTTCTTCTTTCGGAATAACTGAGATGAATGAATTTTTTACCATAACCA
TAAAAATAAAATTTCTCTCTCTTTTTTTTTTTTTTTCCACAGATATGGATTTTACCAATC
AGGAATAATAATAATGCCAGTTATTTTGATCTGATACACCCAATAATCTGGATTTATTCA
TATATTACTTTATTCTATATTCTATCAAATCAAATCAAAATTAAATTCAAATGAATTGTA
AGTACAATTTGTAATTTGATTCGATAGAAGGGCAATTTTTGTACCCACAAAAGAAAATTA
TTTTGACCTAAGAAGATTCTGCCAAATCATTTCTAGATTCAATCCAAATCCGACACCTGA
TATATAGGAAATGTACCAACCATCAACTAATTCCGAATCGTGGATACATATGTATCCTTG
ACATACTGAAACGGCTGCCATTATTGGTATCAAACCAGTAGCGATTCATACAAGCTAAAT
CCTCTAATCGATAATTGGGCCAAAGAAACAATTTGAATTGAATGAATTTATTTATATCTG
TATCAATATGCTTGTCCTCATCCAAAAATTGTCCCCAGTTCCTTACATTGTTGTCATTGA
AAAATACCGGATTTCTATCCATAGCATTCCTATTTCCGGAATTGAAACAAATTAGAATTC
TCAATTCTCTACGACGCCTAGGGGATAGAATATTTTCAGGAACAAGCAAATCATAATGAT
TTTCGTCTCTATTCACAAGCATCTTTTTATGTTGTACAATGGATCCATTGAAATAATTCT
CATCAACATATCTTTTTTTCTCGATATCTTCCATTAGTTTGGCATTTATTATTATGGACCA
ATGAGATACCTATGGTTTGATACATAATAAATTGTCTATCCCATTTTATAGACAGACGTA
CTGGTTCAAGAATCAATATTCCCTTTTTTTATCAATTCTGGAAGAGTTGGATTCGTCTGAA
TTAACATTACATCCAGGTGCATTTCTCCTCCTTGAATAGAGGATATAGCAATTTCCTTTG
CATTTATTAGTCTAAGCAGGAAACAATATACCTTAACATTATTGAAAATTCTGTTATTCA
AAAGATCATCCCATCTCAACTGAAAAAGCAAATATTTTTTCAGGAATAACTCTTGTTTTG
CTTTCTTGTTACTCTCGGGTTGTCCTTTCTTTTCACGTTTTTGAATGTCCGATTCCGTGT
AATCCTTTTCAAAATCTTTTTGTCGTTTTCGTGCGTCTGAGCCAATATTTCCGTGGCCGA
GCTGTTCTTTTTCTTCTTGATTTCGATTCTTTAATTCAAGATATTCTTTTTGATTAGATG
ATATACGAAGATCCTTTTTTTGATTTTCATTAATGTTTTTGTTTTCACTAATGTTTTCAT
TTCCATTAAAAATCAAAAGAAGTAATTTGATTGGTATAATCCACGGTTTGATCTTATATG
CATCATAAAGTGGCACAAATTCTGAGAAGAACCAAGATTTCGTATTTAATATGGGGCGAT
ATAGCATTTCTTTATTCATTCCCATCAAAAAAAACTTTTTTTTTTGATTGGGTGGGTTGA
TTTCTTGATGAATCGTGAGATAGAAAAGATCTTTCTTATCAATCATTTGATAATAATAAG
TCTCGGTCTTAGTCATTTTATTAATGTTGGTCCCGGTATCCGTATCGGTCCAGGACTCAA
TATCGATATTCTTTCTAAGAAATAAATCGAAAATTTTCCACTCCAAATATTTTCTATCCG
TACTTTTACCCGTACCAATAATAGACTCTTCTCCTAGATAATCACTAATACATATATCCC
CCAGTACATAAAATGGTTCAATTTTAGGTGTGTTGTAATTATATGGAATCTCTCGGTCCT
CGTTTACTTGTAATGAGGATGGATAAATATATGAGTCTTTCCTATCCCCATAATTAATAT
ATTTATATGAAAAAAGATCATATCTGTAGTTTTTTTTTAATTTTGCTTTTTTTTATCGTCA
ATGAATTCACTTCATAATCATTTTTTTTTCTCGTAATGAATGAATTGGGCTTTTTCATATG
AATTCTTTTTTGAGTCTTTATTTTGAATCGTACGACGCCGATTGACCCTAGTTCGCCATT
TTTGCGGTACTAATTTAGACCACCTAGCCTGAGATAAATTGTATTGATAATGACCCCTTA
ACCAGTTTTTCCACTCATTCATTCCAGAATTCGGAAGTTTTTTATGCCTTGATTTGGAAT
CTAATATTCTTCGTGTTCCAAAAAAATTCCGGATTCTATCCTTAAGAATAAGATATGCTT
CGCGATATTGAAGTAGAGATCTCAAATGATACTTCTTATTAATAGCTTGGATTTGTGATA
ATTTGTAAAATACAGATGCTTGTGAAAAGGAATATAGGTCACCAGAAATCTTTGATTTAT

TTTTACTAATATTAGAAATATACTTTTTTATAGTCGAAATAAAGTGCATTTTTTTTTGAT
TTATTTCATCAATCCCTTCTTTGTGAATGTATTTATCGATAATCTTTTTTGTTGATTCAA
GGAAAAGTTGTACGTTGACTCTAGAAACATTAACAGTATATAGAAAGATATGTATGTATA
TTCTTTCGTCGAGAAATGTCAAAAAATAGTGCCATTTGCGTATGAATATGAATCTGTTAC
TTTTTCCTTTTGATATCTGCCAAATATGTTTCTGCGATTCCGATCTTTTATCACCACAAC
TTGTATCGTTAGGACTAATATTTATATCCGGAGTTAGAAATATTTTTCTTTTGTCTTTTG
CACCCCTTTCTATTTGATTTCTGATTAGGGTTGTTCTATCGGACAGATCTTTCTTTTTTT
TTTCTGTCAGTGAATAATTTGCCCAATCCATGAATCTAATTCGAATGGTCGATGTCGATT
TAGGAACAATTTTATTACTGCTTATGATTATGGAATCTTTTCCATTTTCATTCGGTTCAT
ATACTTTCTTCAATACAAATAAGAAAATTGGATTTACGTTTGCAAACTCTTTTATTTTTC
TTTTTAAAAATAGAATCGTTTTGATAATCCATCTTGTTTTTTCTTTTGAGACCTTTCTAA
ACTGTTTTGTTTTTTTTTTGAAAACTCTTAGAACTAGAAAACATTTTTTTTTCACTTTTA
TCTTTTTTTTTTCGAATTCATTATAAATAGGTTCAAAAAAGGAAGGTTGTTGTCGGGCAG
AACCAAAAGGTAGTTCGGTTTCCTTTCCCCAGATTGTTAAAAAACAAAAATTTTCTGTTT
TCCCTTTCTTTTGGATTGGATCTCTATGATGGGATCGTAGTTTGGATTTGCGCCAGGGTT
TCAGACAGAAAGGAAATAGGATTTTTATCTGAATACCATCTGTTAACCAGTTTTTCGGAA
ATTCTGTTTCTGATAATTGAACACCATTATAGGTGCATTTAACATGCATTTCTCTATTCC
ACTCCTTCAAATCCTCGTACCACTCGGGGAATTGAAATAAGAGCATACGGCCGAGGTTTT
TAGCTATTATCAATGAAGGCAATATGATGTATTTTCTAAGAATCGATTGGGTTACTAACA
TAGTACCTCTTATTGCTTGAGCAAATATAAGAGTATCCCATGTTTCTGCTATTGCTATCC
GTTCATTCTCGTTTTTTTTATCTTCAATTTTTTTTGCCTCTTCTTTTGCCTCTTCCTCCT
CAGAATCCGAAGTTTTGAGTTTCGGTCCTGTATCTATCCAATTTCTAAAAATTAGATTCA
TTGTTCGGGAGATATCAAAAGAAAAAAAAAAAGTTTTGTCTATTCTGTCCAAAAAAAGCA
GGGAATGTGCATTCGCTTGAAACATTTCCCAAATAACCATTTTACGTCTTTGAGCGCGTA
TGGATCCTTTTACTATATCCCGACGAAAATCTGATTGTTGCGAGTAACGTACCAAAGCCA
ACTCTTCTGCTTGATCACTATTAGTACTGGTACTAGTATGAGTATTGGTATTCTGATCCG
GATCCGCCTTATCAGTATAAATTACCACACGTTTGGCTTTTCTTGAACGAATCCCATGAT
TTTCTGTTGATTCTTCTGATTCTTCCTCATTTTCCTCCTCCCGCTCTTCAAAAGAATTGA
TCAATTTGTATGATCGTCGAGGAATCTTTTTACCGATTTCTTCTATTCCAATAGATTTAT
TTTGAATTGTTTGATTATTTTGATCAGTTGTAATTACATCGAATAGAAATTTCAAACATT
TTTTTTGATTTTCTGAATCAAATCTTCTTTGTTCGGATATTAAAACAAGCTTTTTAAAAT
TCAGACTAAAACCAGTTCTTGATTTCCTAGCTAATTCACTGATAGAGGTCAAGAAAGGAC
CAATGTCTGTCGATAATGGTTCTCCATTAAAAGTATCCGTTTTAGGTTCAAGTTTTTGGT
AATCAGTAGGAAGCCTATCATGAATCTTATTTATCCAAACCATTCCATAG
TCCCTATAGAATCTTCTGTCCCTATAGAATCTTCTGTCGAAGTGATTGAGTCATCCACCA
TTGAACGTGAATACACTTTTTTTATTGTTCCACGATATGGTCCGTTTAAAAAAGGATCAT
ACACTCTAGGCAAGCATTCTTGGTTATTCTTATCTTCTTGGTTATTCTTATCTTCTTGGT
TATTCTTATCTTCTTGGTTATTCTTATCCTCTTGGTTATTTTTATCATTATACAATCTGG
TCCTTTTTTCAAGCACATCCATAGTAAGAGATCCCTTGTTTAGAACTTCTATTCGGTTTA
TGAATTCATTGCTCAAGCTGTACCTTTTTTGTTCATTGGTATAAACCCAATGATTATACA
GATCTTCGGGGGATAGTTTTTCGGTCGTACACAAAGACATCTTTCTTTGCATCATTTCCA
AAAAAATCGATAAACTGGGTGGATATGTAAAAGATATTATTTGTTTTCCATCAACTGGAC
ATACGTGAAAAAAATATTGTGACATTTCATTTCTTACAGCATTTTGAAATAAATTTTTTT
TTATATATCTCAATGGACGATTACATCGTTTATAGTCGAAAAGAAGATTCACAAGAGGTT
TTTCAAACCAGAAGAGGTCTTTATCTTCTTTCTCTTTAAGTATTTCTAACTTCAAAATTT
CTTGCCTCTTTTTTTTTTCATGTAGTTTTTTTGGATCCTCCCTTTCTTCCGAACAAAGGG
AAGGGTCTTCTTCGGTGGATCCCTCTTGTTCCTGTTTAGTCCCCTTCGTTTCGGAAGTTT
TTTCTATTTCTACATCTGTTTCTTCCTCACTTTCCCCCCTTTCTTCCGTTTTTGAGGTTT
CTTTCAGTTTCTTAGTGACAATAGGCGACGGTATTCTGCCTAAATAGTAGACACAGGTGA
TAAATAAGAGAATAGTAAAGATTCGAGCCATAGAATTTCTCAATTCTGACACAAGGTACT
TATTAGATCGAATAAGTACATTCGATCTAATAGAATGATTTTGCCGTATCCAGAATAATA
CCAATCCAACCCATTTCATGAAGAAAATGTGACCAATTAACCAACCAACAAAACTACTTG
TTACAAATAACATCTTGTTGTTGCATCGAAACATATAAATGTTGACTAATCTGACTAACG
TTGAACTTGGTAAAATGAAATGGTTGAATAATTGAAAAATGAGATTATTCAGGAATACAC
ATTGAATGCTGAGATTACGCATTGAATTTCTGGTAGTAGATCCATAATCCAAAAAGTGTT
TGTGATTGTTCCAGAAGAAATGAAACAAAAGATACGGTAGAACTAGGACAGTTATTGTAT
GAGGTCTACCCAATGCTAGATGCAGAGGCGCATAATAGATCGATATGAACATCATGAGCT
GTCCCGTAATAAAACCAGTTGTTGCTGATACCTCCTTCTCGGTTCCTTCTTCCATAATCC
TAGCTCGGAGAAGGAAAAGATAAGAGGGCCCTATGGAGAATGTGGTCAGAAATCCATAAT
AGAGTCCGACCACAACGACCGAATTTATTATCTTCATGCATAAGGATAATAGATTACCTA
GTAGAAAAGATTTCAAAATCATCACAAACCTCCCTTTTTTCTTTTCTATTGCAATTTCTC
GATTACTATATGATGATTTCTTCACTTTCCATAGATAGAAACAGATAGACTAGAAATGAC
ATCTTTTATGTCAATGATACCCTCTTCTTCGATTGAATGACATCTCTTATGTCAATGACA
CCAAAGGGATATTAAATGAATGGAATTGGGATATGGATGGAATATTGGGATATGGATGGA
ATATAATGAAATAGAGCCACTTTGAGGTTCCCTATGAAATGAGGCATGGAACGGAGCCAC
TACGAAGAAGTTCCGGGAGTTACGAAGGAAGCTTCGGGCTCATATTGGTCATGGGTTGAG

AGCGGGAGTTGAACTCTATGAGATCGAATCTCCCGTTATTCCTCAGTAGCTCAGTGGTAG
AGCGGTTGGCTGTTAACTGACTGGTCGTAGGTTCGAATCCTACTTGGGGAGATTTGATGA
ATTCTTAATTAAAGAATTCAGAATTACAGAATTAAAGGGCTCGCTTTGACCGTTAGGAGT
AGGTAACCCGTTCCCTGTCTTTGTTTCTATTGCACTCTATCTCATCGCATTCTGTTCTGC
GATATTTGAGAATCACCGTCAATACCTCGGTGTAGATCCGGGATAATCCTTTGTTCCATA
GCCCTGGGGCTATTTACAACTAGCCAATTCATAATTCTCAGATGATGTACTAGCAGTGCA
TCAAAGATGCAGTCATCGATTCTCCCGAGAGGCCACAATTACCGCGAGCAAACATATTAA
TGACGAGGAACGCATTTTTGCTATGCTACTAATACTTGTATTTGCTCTGCTATTCTTCCC
AAGCCTGGCTGAGGAAGAGTTACGGGGAGTAAAACACAAATATGCTGATTCGGGCCAGGC
ATACTATATGTATCGATTTTTTCCTTCACGATAAATAAAAAGAATAAGGCCATTTCGACA
AAAGACCCACACCCCAGTTCCATAGCTTTGCGTCCGCTATCCCGATCATGATTTTCCTAC
CCCCAGAGGTAAAGGAAAGGTCCTTCCCTTTTGGGCCGGTTGTGGGCGAGGAGGGATTCG
AACCCCCGACACCGTGGTTCGTAGCCACGTGCTCTAATCCTCTGAGCTACAGGCCCCACC
CCGTCTCCACTGGATCTGTTCCCTGGAGTACCCTCAAAAAGGAACCTTTTCTCCCCTCAG
CCATTTCGGGTTAAGAAGACGTGAAAGCGCCTTTCTCTCTATAAGAACAGCGCGTTCCGA
GGTGTGAAGTGGGAGAGAAGGGATGTCATAATTGGGTTTTGAATAAGACGACCTTTTCAT
TTTTTTCTTTTTGAGTAATAAGAATAAGAGGTGTTAAGCTTTTTATCATCCTGGCGTCGA
GCTATTTTTCCGCAGGACCTCCCCTACAGTATCGTCACCGCAGTAGAGTTTAACCACCAA
GTTCCGGGATGGATTGGTGTGGTTCCTCTACGCCTAGGACACCAGAATATCGAACCATGA
ACGAAGAAAGGTATGAGAGAAATATTGGCTAGTGATTGTGAAGCCCAAATTCTTGACTGG
AAGGGACACCAAAGGCCTCTGCCCTTCCATCCCTTGGATAGATAGAGAGGGAGGGCAGAG
CTTTTGGTTTTTTCATGTTGTCAAACAGTTGAACAATGAAAATAGATGGCGAGTGCCTGA
TCGAATTGATCGGGTCATGTAGGAACAAGGTTCAAATCTCTCGGTCTGTTAGGATGCCTC
AGCTGCATACATCACTGCACTTCCGCTTGACACCTATCGTAATGATAAACGGCTCGTCTC
GCCGTGACCTTATCTTGGATTCTCAAGACTTCTGTCGCTCCATCCCCGCAGGGGCAGAGA
ACCCGTCGCTGTCTCGGCTGTGCTACCGGAGGCTCTGGGGAAGTCGGAATAGGAGAGCAC
TCATCTTGGGGTGGGCTTACTACTTAGATGCTTTCAGCAGTTATCCGCTCCGCACTTGGC
TACCCAGCGTTTACCGTGGGCACGATAACTGGTACACCAGAGGTGCGTCCTTCCCGGTCC
TCTCGTACTAGGGAAAGGTCCTCTCAATGCCCTAACGCCCACACCGGATATGGACCGAAC
TGTCTCACGACGTTCTGAACCCAGCTCACGTACCGCTTTAATGGGCGAACAGCCCAACCC
TTGGAACATACTACAGCCCCAGGTGGCGAAGAGCCGACATCGAGGTGCCAAACCTTCCCG
TCGATGTGAACTCTTGGGGAAGATCAGCCTGTTATCCCTAGAGTAACTTTTATCCGTTGA
GCGACGGCCCTTCCACTCGACACCGTCGGATCACTAAGGCCGACTTTCGTCCCTGCTCGA
CGGGCGGGTCTTGCAGTCAAGCTCCCTTCTGCCTTTGCACTCGAGGGCCAATCTCCGTCC
GGCCCGAGGAAACTTTTGCACGCCTCCGTTACCTTTTGGGAGGCCTACGCCCCATAGAAA
CTGTCTACCTGAGACTGTCCCTTGGCCCGTAGGTCCTGACACAAGGTTAGAATTCTAGCT
CTTCCAGAGTGGTATCTCACTGATGGCTCGGGCCCCCCCGGAAGGAGGCCTTCTTCGCCC
TCCACCTAAGCTGCGCAGGAAAGGCCCAAAGCCAATCCCAGGGAACAGTAAAGCTTCATA
GGGTCTTTCTGTCCAGGTGCAGGTAGTCCGCATCTTCACAGACATGTCTATTTCACCGAG
CCTCTCTCCGAGACAGTGCCCAGATCGTTACGCCTTTCGTGCGGGTCGGAACTTACCCGA
CAAGGAATTTCGCTACCTTAGGACCGTTATAGTTACGGCCGCCGTTCACCGGGGCTTCGG
TCGCCGGCTCCCCTGTCATCAGGTCACCAACTTCCTTAACCTTCCGGCACTGGGCAGGCG
TCAGCCCCCATACATGGTCTTACGACTTTGCGGAGACCTGTGTTTTTGGTAAACAGTCGC
CCGGGCCTGGTCACTGCGACCTCCTTTGTGAGGAGGCACCCCTTCTCCCGAAGTTACGGG
GCTATTTTGCCGAGTTCCTTAGAGAGAGTTGTCTCGTGCCCCTAGGTATTCTCTACCTAC
CCACCTGTGTCGGTTTCGGGTACAGGTACCCTTTTGTTGAAGGTCCTTCGAGCTTTTCCT
GGGAGTATGGCATGAGTTACTTCAGCGCCGTAGCGCCTGGTACTCGGACATTGGCTCGGG
GCATTTCCTCTACCCCTTCTTACCCTGCCCTAAAAAAGCAAGGTGACCTTGCGTCCTTGA
ACCGATAACCATCTTTCGGCTAACCTAGCCTCCTCCGTACCTCGGGACCAACAAGGGGTA
GTACAGGAATATTCACCTGTTGTCCATCGACTACGCCTTTCGGCCTGATCTTAGGCCCTG
ACTCACCCTCCGTGGACGAACCTTGCGGAGGAACCCTTAGGTTTTCGGGGCATTGGATTC
TCACCAATGTTTGCGTTACTCAAGCCGACATTCTCGCTTCCGCTTCGTCCACACCTGCTC
GCGCGGGAGCTTCCCTCTAAGCGGAACGCTCCCCTACCGATGCATTTTTACATCCCACAG
CTTCGGCAGATCGCTTAGCCCCGTTCATCTTCGGCGCAAGAGCACTCGATCAGTGAGCTA
TTACGCACTCTTTCAAGGGTGGCTGCTTCTAGGCAAACCTCCTGGCTGTCTCTGCACCCC
TACCTCCTTTATTACTGAGCGGTCATTTAGGGGCCTTAGCTGGTGATCCGGGCTGTTTCC
CTCTCGACGATGAAGCTTATCCCCCATCGTCTCACTGTCCGAACTTGACCCCTGTTATTT
TGGGGTCATATCTAGTATTCAGAGTTTGCCTCGATTTGGTACCGCTCTCGCGGCCCGCAC
CGAAACAGCGCTTTACCCCTAGATGTCCAGTCAACTGCTGCGCCTCAACGCATTTCGGGG
AGAACCAGCTAGCTCTGGGTTCGAGTGGCATTTCACCCCTAACCACAACTCATCCGCTGA
TTCTTCAACATCAGTCGGTTCGGACCTCCACTTAGTTTCACCCAAGCTTCATCCTGGTCA
TGGATAGATCACCCAGGTTCGGGTCCATAAGCAGTGACAATTACCCTATGAAGACGCGCT
TTCGCTACGGCTCCGGTGGGTTCCCTTAACCAAGCCACTGCCTAAGAGTCGCCGGCTCAT
TCTTCAACAGGCACGCGGTCAGAGATTTCTCCTCCCACTGCTTGGGAGCTCACGGTTTCA
TGTTCTATTTCACTCCCCGATGGGGGTTCTTTTCACCTTTCCCTCACGGTACTACTTCGC
TATCGGTCACCCAGGAGTATTTAGCCTTGCAAGGGGGTCCTTGCTGATTCACACGGGATT

CCACGTGCCCCATGCTACTCGGGTCAGAGCGTAAGCTAGTGATGCTTTCGGCTACTGGAC
TCTATCCATCTAGGGTGCAGCACTCCACCGCTTCGCCTAGCAGCACGACGCTTGTATTGC
TCTCCCACAACCTCGTTTTCACGGTTTAGGCTGCTCCCATTTCGCTCGCCGCTACTACGG
AAATCGCTTTTGCTTTCTTTTCCTCTGGCTACTAAGATGTTTCAGTTCGCCAGGTTGTCT
CTTGCCTGCCCATGGATTCAGCAGCAGTTCGAAAGGTTGACCTATTCGGGAATCTCCGGA
TCTATGCTTATTTTCAACTTCCCGAAGCATTTCGTCGCTTACTACGCCCTTCCTCCTCTC
TGGGTGCCTAGGTATCCACCGTAAGCCTTTCCTCGTTTGAACCTCGCCATTAATGTGTTG
GCTATGCCATCCTAAGGTGCTGCTAAATGGAAGGATCTTATCAACGTCCATGAATGAGAA
ATCATAGATCGAACTGCCGAATCGGAAAAATTGGGTGCTATCATATACCTTTGCATCGGC
TAAGGTCACGAGCTGGAGATAAGCGGACTCGAACCGCTGACATCCGCCACAGGGTAAACC
ACCGCCTCTCGGGCCTCCCCGACTGATTCTATCATAGAGGCCAACGATAGACAATAACTC
CCCCCCGAACACAGCTTACAACTTTCATCGTACTGTGCTCTCCAAAGAGCAACTCTTCTC
AAAATCTCAAAGGGTGCTGAGTTGGAATCCCATTCTAAGGATTCTTGTGGTTCCGGAGGA
TCCAGCTACACGAGAACCAGGAACGGGGAGCTCTTCCCCTTTTTCCGCCCGACTCTTTGG
TCTTAAGAATGCTGGTTTTAAGAATGAGTGATTGCCCTTCTCCGACCCTTACTGCCCAAC
CGGAGAGCGGACAGCTAATGCGTTCCACTTATTGAACAGGGTCTATGGTCGGTCTGTGAC
CCCTGGATGCCGAAGGCGTCCTTGGGGTGATCTCGTAGTTCCTACGGGGTGGAGACGATG
GGGTCGGTCCATGGATTTTCCTTCCTTTTGCCACATTTCGCTCAAAGGGTTGAAGGGAGA
TAGTGCATCAAGCTGTTCGCAAGGGCCAACTTGATCCTCTTCCCCAGGGATCCAGATGAG
GGAACCCTAGGAGAGCCGCCGACTCCAACTATCGTCCATGTACGATCCATACTAGATCTG
ACCAACTGCCCATCCTACCTCCTCTACGTTCTTGACAGCCCATCTTTGTCTCAGTAGAGT
CTTTCAGTGGCATGTTTCGGTCCTCTTCCCCATTACTTAGAAAAAGTGAGCCACCGGTTC
AGGTACAAGATACTATCATTACCGCCTGGACAATTAGACATCCAACCCGTAATCGCAACG
ACCCAATTGCAAGAGCGGAGCTCTACCAACTGAGCTATATCCCCCCGAGCCCAGTGGAGC
ATGCATGAAGGAGTCAGATGCTTCTTCGATTCTTTTCCCTGGCGCAGCTGGGCCATCCTG
GACTTGAACCAGAGACCTCGCCCGTGAAGTAAATCATCGCACCTACGATCCAACCAATTG
GGAGAGAATCAATAGATTCTTTTTCGGGGGCGATTCATCCTTCCCGAACGCAGCATACAA
CTCTCCGTTGTACTGCGCTCTCCAAGTGTGCTTGTTCCCACCTTCTTCCTTACCATGGCA
AGTATTTGTGAAATAACTCCGATGAGAAGAAAAAGAAGGCGTTAAGAGACCCTCCTGGCC
CAACCCTAGACACTCTAAGATCCTTTTTCAAACCTGCTCCCATTTTGAGTCAAGAGATAG
ATAAATAGACACATCCCATTGCACTGATCGGGGGGCGTTCGTAGTGACTGAGGGGGTCGA
AGACCAAGAAGTGAGTTATTTATACCAAGCATTCTTCTTATGGCTAGATCCAATCTCCTG
GTCCCTGCGGAAAGGAAAAAGAATTTCACGTTCTTCCTTTCGGGAAGGGAGGATTAGGGA
AATCCTATTGATTGCTGCTTTCTCCAGACCTCCGCGGGAAAAGCATGAAAAAAAAAGGCT
CGAATGGTACGATCCCTCCGTCACCCCAGAATGAAAGGGGTGATCTCGTAGTTCTTGGTC
TGTGAAGATGCGTTGTTAGGTGCTCCATTTTCCCATTGAGGCCGAACCTAAACCTGTGCT
CGAGAGATAGCTGTCCATACACTGATAAGGGATGTATGGATTCTCGAGAAGAGAGGAGCC
ATGGTGGTCCCCCCCGGACCGCCCGGATCCCACGAGTGAATAGAAAGTTGGATCTACATT
GGATCTCACCTGAATCGCCCCATCTATCCTCCTGAGGAGAAGTTTGGTTTCAAACTCCGG
TTCGAACAGGAGGAGTACGCCATGCTAATGTGCCTTGGATGATCCACATCCCCGGGTCAG
GCGCTGATGAGCACATTGAACTATCCATGTGGCTGAGAGCCCTCACAGCCCAGGCACAAC
GACGCAATTATCAGGGGCGCGCTCTACCACTGAGCTAATAGCCCTTGCGGGCCCCCCACG
GGGAGGCCCGCTATGCCAAAAGCGAGAGAAAGCCCATCCCTCTCTTTCCTTTTTGCGCCC
CCATGTCGCCACACGGGAGGGGCATGGGGACGTAAAAAAGGGGATCCTATCAACTTGTTC
CGACCTAGGATAATAAGCTCATGAGCTTGTCTTACTTCACCGTCGAGAAACGAAAGAAGA
CTTCCATCTCCAAGCTTAGCTCAGACGTAGCTCGCTTCTTTAGCTCTCCCTGAAAAGGAG
GTGATCCAGCCGCACCTTCCAGTACGGCTACCTTGTTACGACTTCACTCCAGTCACTAGC
CCCGCCTTCGGCATCCCCCTCCTTGCGGTTAAGGTAACGACTTCGGGCATAGCCAGCTCC
CACAGTGTGACGGGCGGTGTGTACAAGGCCCGGGAACGAATTCACCGCCGTATGGCTGAC
CGGCGATTACTAGCGATTCCGGCTTCATGCAGGCGAGTTGCAGCCTGCAATCCGAACTTA
GGACGGGTTTTTGGAGTTAGCTCACCCTGGCGGGATCGCGACCCTTTGTCCCGGCCATTG
TAACACGTGTGTCGCCCAGGGCATAGGGGGCATGATGACTTGACGTCATCCTCACCTTCC
TCCGGCTTATCACCGGCGGTCTGTTCAGGGTTCCAAACTCAATGGTGGCAACTAAACACG
AGGGTTGCGCTCGTTGCGGGACTTAACCCAACACCTTACGGCACGAGCTGACGACAGCCA
TGCACCACCTGTGTCCGCGTTCCCGAAGGCACCCCTCTCTTTCAAGAGGATTCACGGCAT
GTCAAGCCCTGGTAAGGTTCTTCGCTTTGCATCGAATTAAACCACATGCTCCACCGCTTG
TGCGGGCCCCCGTCAATTCCTTTGAGTTTCATTCTTGCGAACGTACTCCCCAGGCGGGAT
ACTTAACGCGTTAGCTACAGCACTGCACGGGTCGATACGCACAGCGCCTAGTATCCATCG
TTTACGGCTAGGACTACTGGGGTATCTAATCCCATTCGCTCCCCTAGCTTTCGTCTCTCA
GTGTCAGTGTCGGCCCAGCAGGGTGCTTTCGCCGTTGGTGTTCTTTCCGATCTCTACGCA
TTTCACCGCTCCACCGGAAATTCCCTCTGCCCCTACCGTACTCCAGCTTGGTAGTTTCCA
CCGCCTGTCCAGGGTTGAGCCCTGGGATTGGACGGCGGACTTGAAAAGCCACCTACAGAC
GCCTTACGCCCAATCATTCCAGATAACGCTTGCATCCTCTGTCTTACCGCGGCTGCTGGC
ACAGAGTTAGCCGATGCTTATTCCTCAGATACCGTCATTGCTTCTTCTCCGGGAAAAGAA
GTTCACGACCCGTGGGCCTTCTACCTCCACGCGGCATTGCTCCGTCAGGCTTTCGCCCAT
TGCGGAAAATTCCCCACTGCTGCCTCCCGTAGGAGTCTGGGCCGTGTCTCAGTCCCAGTG

TGGCTGATCATCCTCTCGGACCAGCTACTGATCATCGCCTTGGTAAGCTATTGCCTCACC
AACTAGCTAATCAGACGCAAGCCCCTCCTCGGGCAGATTCCTCCTTTTGCTCCTCAGCCT
ACGGGGTATTAGCAGCCGTTTCCAGTTGTTGTTCCCCTCCCAAGGGCAGGTTCTTACGCG
TTACTCACCCGTCCGCCACTGGAAACACCACTTCCCGTCCGACTTGCATGTGTTAGGCAT
GCCGCCAGCGTTCATCCTGAGCCGGGATCGAACTCTCCATGAGATTCATAGTTGCATTAC
TTACCAATCTTCCGGTTCGTAGACAAAGCTGATTCGGAATTGTCTTTCATTCCAAGGCTT
GTATCCATGCGCTTCATATTAGCCTGGAGTTCGCTCCCAGCAATATAGCCATCCCTACCC
TCTCACGTCAATCCCACGAGCCTCTTATCCATTCTCATTCGATCACGGCGGGGGAGCAAG
TCAAAATAGAAAAACTCACATTGCGTTGGGTTTAGGGATAATCAGGCTCGAACTGATGAC
TTCCACCACGTCAAGGTGACACTCTACCGCTGAGTTATATCCCTTCCCTGCCCCCATCGA
GAAATAGAACTGACTAATCCTAAGGCAAAGGGTCGAGAAACTCAACGCCACTCTTCCTGA
ACAACTCGGAGCCGGGACTTCTTTTCGCACTATTACGGATACGAAAAGAATGGAAAAATT
GGATTCAATTGTCAACTGCTCCTATCGGAAATAGGATTGACTACGGATTCGAGCCATAGC
ACATGGTTTCATAAAATCCGTACGATTTTCCCGATCTAAATCGAGCAGGTTTTACAGGAA
GAAGATTTTGTTCAGCATGTTCTATTCGATACTGGTAGGAGAAGAACCCGACTCGGTATT
GTTAAAAAAAGAGAGGAAGCAGAACCAAGTCAAGATGATACGGATCAACCCCTTCTTCTT
GCGCCAAAGATCTTACCATTTCCGAAGGAACTGGAGCTACATCTCTTTTCAATTTCCATT
CAAGAGTTCTTATGTGTTTCCACGCCCTTTTGAGACCTCGAAAAAGGGACAAATTCCTTT
TCTTAGGAACACATACAAGATTCGTCACTACAAAAAGGATAATGGTAACCCC
AACATTAACTACTTCATTTCTGAATTTAATAGTAATAGAAATACATGTCCTACCGAGACA
GAATTTGTAACTTGCTATCCTCTTGCCTAGCAGGCAAAGATTGACCTCCGTGGAAAGACT
GATTCATTCGGATCGACATGAGGGTCCAACTACATTGCATTGCCAGAATCCATTTGTATA
TTTGAAACAGGTTGACCTCCTTGCTTCTCTCATGGTACAATCCTCTTCCCGCTGAGCCCC
CCTTTCTCCTCGGTCCACAGAGACAAAATGTAGGACTGGTGCCAACAGTTCATCACGGAA
GAAAGGACTCACCGAGCCGGGATCACTAACTAATACTAATATAATAGAAAAGAACTGTCT
TTTCTGTATACTTTCCCGGCTCCGTTGCTACCGCGGGCCTTACGCAATCGATCGGATCAT
ATAGATATCCCTTCAACACAACATAGGTCATCGAAAGGATCTCGGACGACCCGCCAAAGC
ACGAAAGCCAGGATCTTTCAGAAAATGGATTCCTATTCGAAGAGTGCATAACCGCATGGA
TAAGCTTACACCAACCCGTCAATTTGGGATCCAATTCGGGATTTTCCTTGGGAGGTATCG
GGAAGGAATTGGAATGTAATAATATCGATTCATACAGAAGAAAAGGTTCTCTATTGATTC
AAACGCTGTACCTATGGGATAGAGGAAGAGGAAAAAACCGAAGATTTCACATAGTACTTT
TGATCGAAAAATCAATCGGATTTATTTCGTACCCCTCGTTCGATGAGAAAATGGGTCAGA
TTCTACAGGATCAAACCTATGGGACTTAAGGAATGATGGAAGGGAATAAAAAGAAAAGAG
AGGGAAAGAAAATCGAAATAAAGAATCAAAGAAAATAAATAAATAAAAAATACAAAAATA
AATAAAGATTCCAAATGAACAAATTCAAACTCAAAAAGGATCTTTCTGATTCTCGAAGAA
TGAGGGCAAAGGGATTGATCGAGAAAGATCTCTTGTTCTTATTATAAGATCGTGATTTGA
TCCGCATATTTTGGTAAAAAGAATAATCTTCTCCTTTGATCATAATCAAAAATGGAAAGT
GTTCAATTGGAACATGAGAACGTGACTGAATTGGTCCTAGTTACTCTTCGGGACGGAGTG
GAAGAAGGGAGGAGATTCTCGAACGAGGAAAAGGACCCAATTACTTCGAAAGAATTGAAC
GAGGAGCCGTATGAGGTGAAAATCTCATGTACGGTTCTGTAGAGTGACAGTAAGGGTGAC
TTATCTGTCAACTTTTCCACTATCACCCCCAAAAAACCAAACTCTGCCTTACGTAAAGTT
GCCAGAGTACGATTAACCTCTGGATTTGAAATCACTGCTTATATACCTGGTATTGGCCAT
AATTCACAAGAACATTCTGTAGTCTTAGTAAGAGGAGGAAGGGTTAAGGATTTACCCGGT
GTGAGATATCACATTGTTCGAGGAACCTTAGATGCTGTCGGAGTAAAGGATCGTCAACAA
GGGCGTTCTAGTGCGTTGTAGATTCTTATCCAAGACTTGTATCATTGGATGATGCCATGT
GAATCGCTAGAAACATGTGAAGTGTATGGCTAACCCAATCACGAAAGTTTCGTAAGGGGA
CTGGAGCAGGCTACCATGAGACAAAAGATCTTCTTTCTAAAGAGATTCGATTCGGAACTA
TTATATGTCCAAGGTTCAATATTGAAATCATTTCAGAGGTTTTCCCTTACTTTGTCCGTG
TCAACAAACAATTCGAAATACCTCGACTTTTTCAGAACAGGTCCGAGTCAAATAGCAATG
ATTCGAAGCACTTCTTTTTACACTATTTCGGAAACCCAAGGACTCGATCGTATGGATATG
TAAAATACAGGATTTCCAATCCTAGCAGGAAAAGGAGGGAAACGGCTACTCAATTTAAAG
TGAGTAAACAGAATTCCATACTCGATTTCATAGATACATATCAAATTCTGTGGAAAGCCG
TATTCGATGAAAGTCGTATGTACGGCTTGGAGGGAGATCTTTCATATCTTTCGAGATCCA
CCCTACAATATGGGGTCAAAAAGCCAAAATAAGTGATTCGTTTTTAGCCCGTATAAAAAG
AAAACGGATTCTTGAACCTCTTTCACGCTCATGTCACGTCGAGGTACTGCAGAAGAAAAA
ACTGCAAAATCCGATCCAATTTATCGTAATCGATTAGTTAACATGTTGGTTAACCGTATT
CTGAAACACGGAAAAAAATCATTGGCTTATCAAATTATCTATCAAGCCGTGAAAAAGATT
CAACAAAAGACAGAAACAAATCCACTATCTGTTTTACGTCAAGCAATACGTGGAGTAACT
CCCGATATAGCAGTCAAAGCAAGACGTGTAGGTGGATCGACTCATCAAGTTCCTATTGAA
ATAGAATCTACACAAGGAAAAGCACTTGCCATTCGTTGGTTATTAGGGGCATCCCGAAAA
CGTCCGGGTCGAAATATGGCTTTCAAATTAAGTTCCGAATTAGTGGATGCTGCCAAAGGG
AGTGGCGATGCCATACGCAAAAAGGAAGAGACTCATAGAATGGCAGAGGCAAATAGAGCT
TTTGCACATTTTCGTTAATCCATGAACAGGATCTATATAGACACATAGATCCGTGGATCC
ATACATCTCGATCGGAAAAGAATCAATAGAAAAAGAAAGAATTGGAATGGATCGATATCT
TTCTCGAAACAAACGAAAAGGAAAAGAAAGATGAAACATAAATCATGGATCAACTAAGCC
CTCTCGGGGGCTTTCTTAAGAATAAGAAGGAGGAATCTCATGGAAATACCATGGAATAAG

GTTTGATCCTGTTCATGGGGATTCCGTAAATATCCCATTCCAAAAATCGAAAGTTCGAAA
CAATTGGGACTTTTTCGGAGATTGGATGCAGTTACTAATTCAGGATCTGGCATGTACAGA
ATGAAAACTTCATTCTCGATTCTACGAGAATTTTTATGAAAGCGTTTCATTTGCTTCTCT
TCCATGGAAGTTTCATTTTCCCAGAATGTATCCTCATTTTTGGCCTAATTCTTCTTCTGA
TGATCGATTCAACCTCTGATCAAAAAGATATACCTTGGTTATATTTCATCTCTTCAACAA
GTTTAGTAATGAGCATAACGGCCCTATTGTTCCGATGGAGAGAAGAACCTATGATTAGCT
TTTCGGGAAATTTCCAAACGAACAATTTCAACGAAATCTTTCAATTTCTTATTTTACTAT
GTTCAACTCTATGTATTCCTCTATCCGTAGAGTACATTGAATGTACAGAAATGGCTATAA
CAGAGTTTTTGTTATTCGTATTAACAGCTACTCTAGGAGGAATGTTTTTATGTGGTGCTA
ACGATTTAATAACTATCTTTGTAGCTCCAGAATCTTTCAGTTTATGCTCCTACCTATTAT
CTGGATATACCAAGAGAGATGTACGGTCTAATGAGGCTACTACGAAATATTTACTCATGG
GTGGGGCAAGCTCTTCTATTCTGGTTCATGGTTTCTCTTGGCTATATGGTTCATCCGGGG
GAGAGATCGAGCTTCAAGAAATAGTGAATGGTCTTATCAATACACAAATGTATAACTCCC
CAGGAATTTCCATTGCGCTTATATCCATCACTGTAGGAATTGGGTTCAAGCTTTCCCCAG
CCCCTTCTCATCAATGGACTCCTGACGTATACGAAGGAGTGCGGTTCGTTCGACAAATTC
CTACCTCTATATCTATCTCTGAGATGTTTGGATTTTTCAAAACTCCATGGACATGCAGAA
GAGAAATGCTATCCCCACTCGGACCAAGACATAACTTTTACCAAAAGTTTATTGTGATCT
TTTTGTTCAAATAACAATTAAGGTGAAGCAGGGTCAGGAACAACGAATCTCTTTATGATA
AACAGATCCATTTTGCAAGTTCGTTATTACGGGTAGTTCCTACAAAGGATCGGACTAATG
ACGTATACAATACTTGAATTATCGATGTAGATGCTACATAGTTGGTTCTCATCCTTCAGA
GACTACGAGTGTAATAGGAGCATCCGTCGACAAAAGGATCACCCTAAGATGATCATCTCA
TGGCTATTGAGAACGAATCAAATCAGATGGTTCTATTTCTCAATCTTTCTGACTTGTGCT
CCTACGGAACCGGGGTCGAAAAGATTGAAAAAGTCAGTCATTCACAACCACTGATGAAGG
ATTCCTCGAAAAGTTAAGGATTAGTAATCCTTTTTAGAAATCGAATGGATTCGGTCTTAT
ACATACGCGAGGAAGGTAATCAAAAAAGAAAGAAGGTGAGTTCTTCTTTCTTTTATCACT
TAGGAGCCGTGCGAGATGAAAGTCTCATGCACGGTTTTGAATGAGAGAAAGAAGTGAGGA
ATCCTCTTTTCGACTCTGACTCTCCCACTCCAGTCGTTGCTTTTCTTTCTGTTACTTCGA
AAGTAGCTGCTTCAGCTTCAGCCACTCGAATTTTCGATATTCCTTTTTATTTCTCATCAA
ACGAATGGCATCTTCTTCTGGAAATCCTAGCTATTCTTAGCATGATATTGGGGAATCTCA
TTGCTATTACTCAAACAAGCATGAAACGTATGCTTGCATATTCGTCCATAGGTCAAATCG
GATATGTAATTATTGGAATAATTGTTGGAGACTCAAATGATGGATATGCAAGCATGATAA
CTTATATGCTGTTCTATATCTCCATGAATCTAGGAACTTTTGCTCGCATTGTCTCATTTG
GTCTACGTACCGGAACTGATAACATTCGAGATTATGCAGGATTATACACGAAAGATCCTT
TTTTGGCTCTCTCTTCAGCCCTATGTCTCTTATCCCTAGGAGGTCTTCCTCCACTAGCAG
GTTTTTTCGGAAAACTCCATCTATTCTGGTGTGGATGGCAGGCAGGCCTATATTTCTTGG
TTTCAATAGGACTCCTTACGAGCGTTGTTTCTATCTACTATTATCTAAAAATAATCAAGT
TATTAATGACTGGACGAAACCAAGAAATAACCCCTCACGTGCGAAATTATAGAAGATCCC
CCTTAAGATCAAACAATTCCATCGAATTGAGTATGATTGTATGTGTGATAGCATCTACTA
TACCAGGAATATCAATGAATCCAATTATTGCAATTGCTCAGGATACCCTCTTTTAGCTTC
TAGGGTCTATTTCTTAGTTCAAGATCCCTTTTACTAACTGGAATAAAAGAATTAGTAGAT
CTGTTCCGCCCAAAATGGGAATGGGCTGGGGTTATGAACTTATAATCATGGAATCGACTC
GATCATCAGATTATAAGTTCATTCCATACCGGACCAGACCGGAATAGGGTTATGTACATT
CTCATTATGAGAAGGGGTCATTCGAGCATATGTAAATAGAGACTATGTTTACATATGGAT
CCCTACGTCGTTACATTCCATTTAGGATTAGGAATAGGCGTAATCGGACCTGCTTTTTAC
ATATCTATCGTTATTTGGGTACCATATTAACTTCTTTGGGCTTCGATTGAATCGAGAAAT
AGGTTTGATTGTACATCTTTTTGATATATATAAGGTATCCTCCGGATAATTCAAATCGAA
GCAATTTGATGTCCGACTCGGGCCTATATGACATGACCGATCGATAGAAATACTCCAAAA
CTCCACCTTTGTCATATATTCCATATATCACACTAGATAGATATCATATTCATGGAATAC
GATTCACTTTCAAGATGCCTTGATGGTGAAATGGTAGACACGCGAGACTCAAAATCTCGT
GCTAAAGAGCGTGGAGGTTCGAGTCCTCTTCAAGGCATAATATTGAAATGCTCATTGAAT
GAGCAATTCAATAACAGATCTCGGATCTAATCAATATTGATATACATATACCAAGTATCT
GTTGATACGAAGTATTCCATCGATCCCCACGATCCGAGTCCGGGCTGTTGGAATTGGAAT
AGGTTCGGCAGCGGATCACGAAATCTTGGCGATCTTCTCTATCTAATGAATGAGGAGTCC
GTTTTGAAATCGTCCGCCCTGCACCCACCCCCCGAGTATATGCTTCAACAGGAATCACAC
AAGGGTAAATTGATACAATAGAAACCTCTGGTAAAATGCCCGCCCGTAACCCAACAGATA
AAGTACATAGTCCGTTTTAGCCTGTTACATGAATCAAATGTTTCATTTCATCCGGGAAAA
GCCATCTCTTTCTCAACAATGCCTTTGTCATTTGATCCAATAGCGTTCCGTTAGATAGGA
ACAGATTTGATAAATACTGATAACTCTCGGATGGAGTATTAGAACGGAAAGATCCATTAG
ATAATGAACTATTGGTTCGAAGCCATCTCTGGCGATGAATCAACAATTCGAAGTGCTTTT
CTTGCGTATTCTTGATGAACCAGCGTTTATATATAGATGTAGGAGGATTTGTTTGGGAAG
TAATAAGCCCTTTTGACATCTCTTCATCTGCAAAGAATTCTCGGCGTGAAAACACAGAGA
CAAAGGGCTGATCTTTGAATAGGAAAAAGAATGGATCTGCAGGGTCCCAAATGAATTGGC
TTATTCGAAAAAAGCCTTGTTCTTTGGAAGATCTATCTCGTGTCTGGTACTGCATGGTTC
CACTCTGCAAGAACTCCGAATCATTCTCTTGAAGCTCATCCTCTTTATGATAAATGATCC
GCTTGCCCCGAAATGACCTGGCCCAATAGGGAAATCCCAATTCATTGGTCCTTTCGATAC
AATCAAATAGATTGCCACAAGGGCGCCATATTCTAGGAGCCCAAACTATGTGATTGAATA

AATCCTCCTCTATCTGTTGCGGGTCGAGGGCTCCTTCTCTTTCCCCTTCTTCAAACTCCG
ATTCGTATTTTTCATATAGAAATCTCTGATCAACGATAGAACAAGATCCGTTTTGCATCA
TATCTAACTGATTCCTTGGTTCGGACCGAAGAAGCAATGTCACTCGATCATTATCAAACT
GACTGCAATCTTTTTCTGTCCGTGAGGATCCCAACAGAGCGCCTTCGACTTCTAATAGGC
CATGAACTAGATCAGAATCATTCTCAACGAATCCATAAGAAGTGATCCAATTTTTTTCAT
CGGGTCCGGGTGGAGACCAAAGATCTTGAGCGACCGATCCGGCAGAACAACTCAAAAGAT
AAAGAAGTATCGTTAATTTCTTCATGCTCGTTCCAAGTTCGAAGTACCATTTGTACAAAT
AAGAATCCCCTTCCTTACATGATTTCTTCTTCATATAGATAGATATAGGATCTATGGGGC
AATTACTTAGAAGTACATTTTGTGCAACAGCCCTTCCTATCTGATAGAAAAGGATCCCAT
GATCCTGAACCGATCTTACCTGGGATCGCAAATCCCAAGTTTGTCTATGAAGAGCTGATC
TAATTGTATTAGTGTCTATAATTGATTTCTTCTGTGTAATACTAATTGATAGGGCCTCAT
TGGTAAGTGCTACAAGATCTCGTGCATTGGAACCCATGGTTATGGACCCGAATCTGTTAC
TGTTAGTATGGAACATTTTCTTTTCCAAGTGAAATCCCCTAGTATATGAAAGAATGAAAA
AGTGCTTTCGTTGTTGTGGAATAAGAAGCCTTCGTATCTTAATGCATGTATTTAATTTAT
TCGGAGCTATTAGAGTGGGATCCACTTTTTGGGGAATATGAGTCGAAGCAATAACAAGAA
TATTTCTAGTGGAACATCTTTCACTATCTCTGGAGAGATAGTTCACTAATAGACCGAGGG
ATAAGTAATTCGACTCATTCACATACAGATCATGAATGTTTGGAATCCATATTATGCAAG
GGGACATTGCTTTTGCTAATTCTAATTGAAGGGTGGTATCAAATCGGTCTATTTTCGGCG
TCATATACATAGTTAGCACATTCGTCATAGTTAGCAGCTCCGTATCAAGGTCATCATCAA
TATCGTCACTATCATCAATATCGATATCGTCACTATCATCAATATCGATATCGTCAATAA
GATAACCTTTAGGCTTGTCATCCAGGAACTTGTTCGGAAATACCGTAATGAAAGGAACAT
AGGAGTTTGTCGCTAGGTATTTGACCAAATAGGATCGTCCAGTTCCTATAGAACCTATCA
CTAAAATACCCCTAGAAGGGGATAGGGCTAAGCGGAGCGAAAAGGGTTTTCCATGAGATG
GGAAATGAGAACTATTAGCCCCACACGAGGTTTGTGAATAAGTGATTGTCTGATAATGAG
CAAGGAATATCCGTCTTTCTGCTAAACAGGATCTATTGAACTCATAATTCATTAGATACT
TTTTATGAATGTCAACTAAGTATCGTAAGTAAATGGATCCCGGTTGTTCAATCATTTGAT
AACCAGAGTCATTCTTTGATAAACGATCACTATGAGTCAGACTCAATAGAATTTGATCAA
TCCTTTTTTCCGTCGTTAAGGTGGAGAACTGAACCAAGAATTCTCTTTCTTCATCATCAA
TCGAATCACTGTTCGCGACCCAGGATTCTATTTTATCATCAATCCAATCACCGTTCACGT
TTTTTCTTTTTCTTATCAATGAATAGATCTCTTTACTTGTACGACTTAGATGTCTCGTAT
TTCTCGAAAAAGTGATTCGATTGATGGGATTTGGTATGATACTGATGAGATCGATGAGAT
TGATATTCAAATATTTCTTCTTAGAACGTATTGATTTGACCCCATAAGCGGGACCACCAC
CCAATAGCATGTTGCCGCCAGAAGCAGAATCCCGTATTTCTTCCAGAGAATCTCCTAATT
GTTCCAGAGCAACTAGAAAGAGATTCTTTAACCAGAAAGAATTCAGTTCAGATGTAGGAT
ACCTATCCAGAAGTTTTCGCAACTCAATCATGTATGATGGAATCATCAAAGATTTGATCT
TTTCTAACTCTGTCTGTAACTCACTAGAGGCTCGGAAAACAAAGAGAAGATGTGTACGAA
CGAGATATCCAGCAACAAGAAGAAGGAAAAGAATTGAATAGAGGAACTCCCAAGCATTTG
GTGATCTCAGATGTGTCCATATCAATGGAATGGGTGACTCATTATTTCGATGAATCATTT
CTTCGGACAGAAGAAGATTCTGTAAACACTTACTCGAACTCTCACTTATCAGATTCCGTT
GTGGAAGAATCGACCACCACTTTTTCTGAGGAATTGGCCATGATATATCTGATCCATGCA
TAATATCATGAAAAACGGACACAAAATTTTGACTGCCACTTAGGGAATATTGAAAGGGAA
TATTCAATATCAAATAAATATTGTTTTTTAAGGTGAAATAAAGATATTTACACCCCGTCC
AAGT

Supplementary Table S6. Cinnamomum primers

```
Cinna_140_cp1F:  GGAAACCAAATTGGAGATCAA
Cinna_140_cp1R:  AAAGCTTTCCCCTATTTTTCTATTG

Cincas_290_cp1F: CGGATCAAGATACCCATTCG
Cincas_290_cp1R: GGAATCCGTCCTGCCATTA

Cincas_216_cp2F: TCCCTTCATCACATGATTCG
Cincas_216_cp2R: ACATAGGGGCTCATTCAGGA

Cinbur_158_cp1F: TCACAGTCATTAATGCTTCCAA
Cinbur_158_cp1R: TTGATTGGATCCTGTTGTTCTC

Cinbur_129_cp2F: CCCCAAGAGTTCACATCGAC
Cinbur_129_cp2R: TTCTGAACCCAGCTCACGTA
```

Supplementary Table S7. Amplicon sequences for Cincas_216
>CB1_Cinnamomum-burmannii_Cincas-216
atgaaataccttggatctatatcggattggtacatgtatcaatcaaccaagcgaatctcgtccggatgaa
tcaataaaagcaaagcaattaggcgcgtccctgaaacaattcattgcattgatatttctcaaatatcaat
aactaaaacttcctaggtaaatcaaatttattgt
>CB2_Cinnamomum-burmannii_Cincas-216
atgaaataccttggatctatatcggattggtacatgtatcaatcaaccaagcgaatctcgtccggatgaa
tcaataaaagcaaagcaattaggcgcgtccctgaaacaattcattgcattgatatttctcaaatatcaat
aactaaaacttcctaggtaaatcaaatttattgt
>CB3_Cinnamomum-burmannii_Cincas-216
atgaaataccttggatctatatcggattggtacatgtatcaatcaaccaagcgaatctcgtccggatgaa
tcaataaaagcaaagcaattaggcgcgtccctgaaacaattcattgcattgatatttctcaaatatcaat
aactaaaacttcctaggtaaatcaaatttattgt
>CB4_Cinnamomum-burmannii_Cincas-216
atgaaataccttggatctatatcggattggtacatgtatcaatcaaccaagcgaatctcgtccggatgaa
tcaataaaagcaaagcaattaggcgcgtccctgaaacaattcattgcattgatatttctcaaatatcaat
aactaaaacttcctaggtaaatcaaatttattgt
>FC96_Cinnamomum-burmannii_Cincas-216
atgaaataycttggatctatatcgrattggtacatgtatcaatcaaccaagygaatctcgtccggatgar
tcaataaaagcaaagcaattaggmgcgkccctgaaacaattcattgcattgatatttctcaaatatmaat
aactaaaacttcctaggtaaatmaaatttmttgt
>FC111_Cinnamomum-burmannii_Cincas-216
atgaaataycttggatctatatcgrattggtacatgtatcaatcaaccaagygaatctcgtccggatgar
tcaataaaagcaaagcaattaggmgcgkccctgaaacaattcattgcattgatatttctcaaatatmaat
aactaaaacttcctaggtaaatmaaatttmttgt
>FC144_Cinnamomum-burmannii_Cincas-216
atgaaataycttggatctatatcgrattggtacatgtatcaatcaaccaagygaatctcgtccggatgar
tcaataaaagcaaagcaattaggmgcgkccctgaaacaattcattgcattgatatttctcaaatatmaat
aactaaaacttcctaggtaaatmaaatttmttgt
>CC1_Cinnamomum-cassia_Cincas-216
atgaaataccttggatctatatcggattggtacatgtatcaatcaaccaagcgaatctcgtccggatgaa
tcaataaaagcaaagcaattaggagcgtccctgaaacaattcattgcattgatatttctcaaatatcaat
aactaaaacttcctaggtaaataaaatttattgt
>CC2_Cinnamomum-cassia_Cincas-216
atgaaataccttggatctatatcggattggtacatgtatcaatcaaccaagcgaatctcgtccggatgaa
tcaataaaagcaaagcaattaggagcgtccctgaaacaattcattgcattgatatttctcaaatatcaat
aactaaaacttcctaggtaaataaaatttattgt
>CC3_Cinnamomum-cassia_Cincas-216
atgaaataccttggatctatatcggattggtacatgtatcaatcaaccaagcgaatctcgtccggatgaa
tcaataaaagcaaagcaattaggagcgtccctgaaacaattcattgcattgatatttctcaaatatcaat
aactaaaacttcctaggtaaataaaatttattgt
>CC4_Cinnamomum-cassia_Cincas-216
atgaaataccttggatctatatcggattggtacatgtatcaatcaaccaagcgaatctcgtccggatgaa
tcaataaaagcaaagcaattaggagcgtccctgaaacaattcattgcattgatatttctcaaatatcaat
aactaaaacttcctaggtaaataaaatttattgt
>379NW_Cinnamomum-cassia_Cincas-216

atgaaataycttggatctatatcgrattggtacatgtatcaatcaaccaagygaatctcgtccggatgaa
tcaataaaagcaaagcaattaggagcgtccctgaaacaattcattgcattgatatttctcaaatatcaat
aactaaaacttcctaggtaaataaaatttattgt
>CV1_Cinnamomum-verum_Cincas-216
atgaaataccttggatctatatcggattggtacatgtatcaatcaaccaagcgaatctcgtccggatgaa
tcaataaaagcaaagcaattaggagcgtccctgaaacaattcattgcattgatatttctcaaatatcaat
aactaaaacttcctaggtaaatcaaatttattgt
>CV2_Cinnamomum-verum_Cincas-216
atgaaataycttggatctatatcggattggtacatgtatcaatcaaccaagcgaatctcgtccggatgaa
tcaataaaagcaaagcaattaggagcgtccctgaaacaattcattgcattgatatttctcaaatatcaat
aactaaaacttcctaggtaaatcaaatttattgt
>CV3_Cinnamomum-verum_Cincas-216
atgaaataycttggatctatatcggattggtacatgtatcaatcaaccaagcgaatctcgtccggatgaa
tcaataaaagcaaagcaattaggagcgtccctgaaacaattcattgcattgatatttctcaaatatcaat
aactaaaacttcctaggtaaatcaaatttmttgt
>CV17_Cinnamomum-verum_Cincas-216
atgaaataccttggatctatatcggattggtacatgtatcaatcaaccaagcgaatctcgtccggatgaa
tcaataaaagcaaagcaattaggagcgtccctgaaacaattcattgcattgatatttctcaaatatcaat
aactaaaacttcctaggtaaatcaaatttattgt
>CV46_Cinnamomum-verum_Cincas-216
atgaaatacctgggatctatatcggattggtacatgtatcaatcaaccaagcgaatctcgtccggatgaa
tcaataaaagcaaagcaattaggagcgtccctgaaacaattcattgcattgatatttctcaaatatcaat
aactaaaacttcctaggtaaatcaaatttattgt
>2USP_Cinnamomum-verum_Cincas-216
atgaaataycttggatctatatcggattggtacatgtatcaatcaaccaagcgaatctcgtccggatgaa
tcaataaaagcaaagcaattaggagcgtccctgaaacaattcattgcattgatatttctcaaatatcaat
aactaaaacttcctaggtaaatcaaatttattgt
>10USP_Cinnamomum-verum_Cincas-216
atgaaataccttggatctatatcggattggtacatgtatcaatcaaccaagcgaatctcgtccggatgaa
tcaataaaagcaaagcaattaggagcgtccctgaaacaattcattgcattgatatttctcaaatatcaat
aactaaaacttcctaggtaaatcaaatttattgt
>3ING_Cinnamomum-verum_Cincas-216
atgaaataccttggatctatatcggattggtacatgtatcaatcaaccaagcgaatctcgtccggatgaa
tcaataaaagcaaagcaattaggagcgtccctgaaacaattcattgcattgatatttctcaaatatcaat
aactaaaacttcctaggtaaatcaaatttattgt
>VS3_Cinnamomum-verum_Cincas-216
atgaaataycttggatctatatcggattggtacatgtatcaatcaaccaagygaatctcgtccggatgaa
tcaataaaagcaaagcaattaggagcgkccctgaaacaattcattgcattgatatttctcaaatatmaat
aactaaaacttcctaggtaaatcaaatttmttgt
>VS23_Cinnamomum-verum_Cincas-216
atgaaataycttggatctatatcgrattggtacatgtatcaatcaaccaagygaatctcgtccggatgaa
tcaataaaagcaaagcaattaggagcgkccctgaaacaattcattgcattgatatttctcaaatatmaat
aactaaaacttcctaggtaaatcaaatttmttgt

Supplementary Table S8. Amplicon sequences for Cincas_290

```
>CB1_Cinnamomum-burmannii_Cincas-290
ctccagtataaatagtaactatctgttcttccaccgtgagaggggctgattgggattgtttgagcaactc
gcgcaatcgttgacctcttgccaattgattctgagtggctttatcgagatcagaagcgaattgtgcaaag
gcttctaactctgcgaattgagccagttccagtttcgatttgccggctacttgtttcatggctttaattt
gagctgcag
>CB2_Cinnamomum-burmannii_Cincas-290
ctccagtataaatagtaactatctgttcttccaccgtgagaggggctgattgggattgtttgagcaactc
gcgcaatcgttgacctcttgccaattgattctgagtggctttatcgagatcagaagcgaattgtgcaaag
gcttctaactctgcgaattgagccagttccagtttcgatttgccggctacttgtttcatggctttaattt
gagctgcagatc
>CB3_Cinnamomum-burmannii_Cincas-290
ctccagtataaatagtaactatctgttcttccaccgtgagaggggctgattgggattgtttgagcaactc
gcgcaatcgttgacctcttgccaattgattctgagtggctttatcgagatcagaagcgaattgtgcaaag
gcttctaactctgcgaattgagccagttccagtttcgatttgccggctacttgtttcatggctttaattt
gagctgcagatcctactctcgagacagaaatacccacat
>CB4_Cinnamomum-burmannii_Cincas-290
ctccagtataaatagtaactatctgttcttccaccgtgagaggggctgattgggattgtttgagcaactc
gcgcaatcgttgacctcttgccaattgattctgagtggctttatcgagatcagaagcgaattgtgcaaag
gcttctaactctgcgaattgagccagttccagtttcgatttgccggctacttgtttcatggctttaattt
gagctgcagatcctactctcgagacagaaatacccacat
>FC96_Cinnamomum-burmannii_Cincas-290
ctccrgyataaatagtmactatmtgttcttccaccgtgaraggggctgattgggattgtttgagcaactc
rcgcaatcgttgacctcttgccaattgattctgagtggctttatcgagatcagaagcgaattgtgcaaag
gcttctaactctgcgaattgagccagttccagtttsgatttgccrgctacttgtttcatggctttmattt
gagctgcagatcctactctcgaracagaaatacccamwt
>FC111_Cinnamomum-burmannii_Cincas-290
ctccagtataaatagtmactatmtgttcttccaccgtgaraggggctgattgggattgtttgagcaactc
rcgyaatcgttgacctcttgccaattgattctgagtggctttatcgagatcagaagcgaattgtgcaaag
gcttctaactctgcgaattgagccagttccartttsgatttgccggctacttgtttcatggctttmattt
gagctgcagatcctactctcgaracagaaatacccacat
>FC144_Cinnamomum-burmannii_Cincas-290
ctccrgyataaatagtmactatmtgttcttccaccgtgaraggggctgattgggattgtttgagcaactc
rcgcaatcgttgacctcttgccaattgattctgagtggctttatcgagatcagaagcgaattgtgcaaag
gcttctaactctgcgaattgagccagttccartttsgatttgccrgctacttgtttcatggctttaattt
gagcygcagatcctactctcgaracagaaatacccacat
>CC1_Cinnamomum-cassia_Cinnamomum-cassia_Cincas-290
ctccagtataaatagtcactatctgttcttccaccgtgagaggggctgattgggattgtttgagcaactc
gcgcaatcgttgacctcttgccaattgattctgagtggctttatcgagatcagaagcgaattgtgcaaag
gcttctaactctgcgaattgagccagttccagtttcgatttgccggctacttgtttcatggctttaattt
gagctgcagatcctactctcgagacagaaatacccacat
>CC2_Cinnamomum-cassia_Cincas-290
ctccagtataaatagtcactatctgttcttccaccgtgagaggggctgattgggattgtttgagcaactc
gcgcaatcgttgacctcttgccaattgattctgagtggctttatcgagatcagaagcgaattgtgcaaag
gcttctaactctgcgaattgagccagttccagtttcgatttgccggctacttgtttcatggctttaattt
gagctgcagatcctactctcgagacagaaatacccacat
>CC3_Cinnamomum-cassia_Cincas-290
```

ctccagtataaatagtcactatctgttcttccaccgtgagaggggctgattgggattgtttgagcaactc
gcgcaatcgttgacctcttgccaattgattctgagtggctttatcgagatcagaagcgaattgtgcaaag
gcttctaactctgcgaattgagccagttccagtttcgatttgccggctacttgtttcatggctttaattt
gagctgcagatcctactctcgagacagaaatacccacat
>CC4_Cinnamomum-cassia_Cincas-290
ctccagtataaatagtcactatctgttcttccaccgtgagaggggctgattgggattgtttgagcaactc
gcgcaatcgttgacctcttgccaattgattctgagtggctttatcgagatcagaagcgaattgtgcaaag
gcttctaactctgcgaattgagccagttccagtttcgatttgccggctacttgtttcatggctttaattt
gagctgcagatcctactctcgagacagaaatacccacat
>379NW_Cinnamomum-cassia_Cincas-290
ctccagtataaatagtmactatctgttcttccaccgtgagaggggctgattgggattgtttgagcaactc
gcgcaatcgttgacctcttgccaattgattctgagtggctttatcgagatcagaagcgaattgtgcaaag
gcttctaactctgcgaattgagccagttccagtttcgatttgccggctacttgtttcatggctttaattt
gagctgcagatcctactctcgagacagaaatacccacat
>CV1_Cinnamomum-verum_Cincas-290
ctccagtataaatagtaactatctgttcttccaccgtgagaggggctgattgggattgtttgagcaactc
gcgcaatcgttgacctcttgccaattgattctgagtggctttatcgagatcagaagcgaattgtgcaaag
gcttctaactctgcgaattgagccagttccagtttcgatttgccggctacttgtttcatggctttaattt
gagctgcagatcctactctcgagacagaaatacccacat
>CV2_Cinnamomum-verum_Cincas-290
ctccagtataaatagtaactatctgttcttccaccgtgagaggggctgattgggattgtttgagcaactc
gcgcaatcgttgacctcttgccaattgattctgagtggctttatcgagatcagaagcgaattgtgcaaag
gcttctaactctgcgaattgagccagttccagtttsgatttgccggctacttgtttcatggctttaattt
gagctgcagatcctactctcgagacagaaatacccacat
>CV3_Cinnamomum-verum_Cincas-290
ctccagtataaatagtaactatctgttcttccaccgtgagaggggctgattgggattgtttgagcaactc
gcgcaatcgttgacctcttgccaattgattctgagtggctttatcgagatcagaagcgaattgtgcaaag
gcttctaactctgcgaattgagccagttccagtttsgatttgccggctacttgtttcatggctttaattt
gagctgcagatcctactctcgagacagaaatacccacat
>CV17_Cinnamomum-verum_Cincas-290
ctccagtataaatagtaactatctgttcttccaccgtgagaggggctgattgggattgtttgagcaactc
gcgcaatcgttgacctcttgccaattgattctgagtggctttatcgagatcagaagcgaattgtgcaaag
gcttctaactctgcgaattgagccagttccagtttcgatttgccggctacttgtttcatggctttaattt
gagctgcagatcctactctcgagacagaaatacccacat
>CV46_Cinnamomum-verum_Cincas-290
ctccagtataaatagtaactatctgttcttccaccgtgagaggggctgattgggattgtttgagcaactc
gcgcaatcgttgacctcttgccaattgattctgagtggctttatcgagatcagaagcgaattgtgcaaag
gcttctaactctgcgaattgagccagttccagtttcgatttgccggctacttgtttcatggctttaattt
gagctgcagatcctactctcgagacagaaatacccacat
>2USP_Cinnamomum-verum_Cincas-290
ctccagtataaatagtaactatctgttcttccaccgtgagaggggctgattgggattgtttgagcaactc
gcgcaatcgttgacctcttgccaattgattctgagtggctttatcgagatcagaagcgaattgtgcaaag
gcttctaactctgcgaattgagccagttccagtttcgatttgccggctacttgtttcatggctttaattt
gagctgcagatcctactctcgagacagaaatacccacat
>10USP_Cinnamomum-verum_Cincas-290
ctccagtataaatagtaactatctgttcttccaccgtgagaggggctgattgggattgtttgagcaactc
gcgcaatcgttgacctcttgccaattgattctgagtggctttatcgagatcagaagcgaattgtgcaaag

gcttctaactctgcgaattgagccagttccagtttcgatttgccggctacttgtttcatggctttaattt
gagctgcagatcctactctcgagacagaaatacccacat
>3ING_Cinnamomum-verum_Cincas-290
ctccagtataaatagtaactatctgttcttccaccgtgagaggggctgattgggattgtttgagcaactc
gcgcaatcgttgacctcttgccaattgattctgagtggctttatcgagatcagaagcgaattgtgcaaag
gcttctaactctgcgaattgagccagttccagtttcgatttgccggctacttgtttcatggctttaattt
gagctgcagatcctactctcgagacagaaatacccacat
>VS3_Cinnamomum-verum_Cincas-290
ctccagtataaatagtaactatctgttcttccaccgtgagaggggctgattgggattgtttgagcaactc
gcgcaatcgttgacctcttgccaattgattctgagtggctttatcgagatcagaagcgaattgtgcaaag
gcttctaactctgcgaattgagccagttccagtttcgatttgccggctacttgtttcatggctttaattt
gagctgcagatcctactctcgagacagaaatacccacat
>VS23_Cinnamomum-verum_Cincas-290
ctccagtataaatagtaactatctgttcttccaccgtgaraggggctgattgggattgtttgagcaactc
gcgcaatcgttgacctcttgccaattgattctgagtggctttatcgagatcagaagcgaattgtgcaaag
gcttctaactctgcgaattgagccagttccagtttsgatttgccggctacttgtttcatggctttaattt
gagctgcagatcctactctsgagacagaaatacccacat

Supplementary Table S9 Genbank accession numbers

	**Species**	**Sample Accession**	**GenBank Accessions**
1	*Cinnamomum burmannii* (Nees & T.Nees) Blume	BRM1531	In progress
2	*Cinnamomum burmannii* (Nees & T.Nees) Blume	BRM1532	In progress
3	*Cinnamomum burmannii* (Nees & T.Nees) Blume	BRM1533	In progress
4	*Cinnamomum burmannii* (Nees & T.Nees) Blume	BRM1534	In progress
5	*Cinnamomum burmannii* (Nees & T.Nees) Blume	FC96	In progress
6	*Cinnamomum burmannii* (Nees & T.Nees) Blume	FC111	In progress
7	*Cinnamomum burmannii* (Nees & T.Nees) Blume	FC144	In progress
8	*Cinnamomum cassia* (L.) J. Presl	BRM1535	In progress
9	*Cinnamomum cassia* (L.) J. Presl	BRM1536	In progress
10	*Cinnamomum cassia* (L.) J. Presl	BRM1537	In progress
11	*Cinnamomum cassia* (L.) J. Presl	BRM1538	In progress
12	*Cinnamomum cassia* (L.) J. Presl	379NW	In progress
13	*Cinnamomum verum* J. Presl	BRM1547	In progress
14	*Cinnamomum verum* J. Presl	BRM1548	In progress
15	*Cinnamomum verum* J. Presl	BRM1549	In progress
16	*Cinnamomum verum* J. Presl	BRM1550	In progress
17	*Cinnamomum verum* J. Presl	BRM1551	In progress
18	*Cinnamomum verum* J. Presl	VS3	In progress
19	*Cinnamomum verum* J. Presl	VS23	In progress
20	*Cinnamomum verum* J. Presl	2USP	In progress
21	*Cinnamomum verum* J. Presl	10USP	In progress
22	*Cinnamomum verum* J. Presl	3ING	In progress

Supplemental Table S10: Chemical shift details for the 16 bioactive molecules assigned using structural elucidation of proton structure within cinnamon NMR spectra.

SNO	Metabolite	1H Multiplicity
1	Cinnamaldehyde	9.66 (d), 7.46 (d), 6.80-6.74 (dd)
2	Coumarin	6.46 (d)
3	Methoxy cinnamaldehyde	9.62(d), 7.66 (d), 3.83 (s)
4	Cinnamic Acid	7.95 (t), 7.58 (t), 6.50-6.51 (dd)
5	Benzoic Acid	8.12 (dd), 7.66 (t), 7.44 (d)
6	Methyl Salicylate	7.58 (d), 7.08 (d), 3.93 (d), 3.79 (d)
7	Quinic Acid	4.06-4.03 (m)
8	Eugenol	5.88-5.98 (m)
9	α-glucose	5.12 (d)
10	β-glucose	4.49 (d)
11	Fructose	4.0 (d)
12	Formic acid	8.23 (s)
13	Choline	3.22 (s)
14	Shikimic Acid	2.79 (t)
15	Succinic acid	2.15 (s)
16	Alanine	1.61 (d)
